# Convergent Synthesis of the C1–C29 Framework of Amphidinolide F

**DOI:** 10.1021/acs.joc.2c00850

**Published:** 2022-06-08

**Authors:** Filippo Romiti, Ludovic Decultot, J. Stephen Clark

**Affiliations:** School of Chemistry, University of Glasgow, Joseph Black Building, University Avenue, Glasgow G12 8QQ, U.K.

## Abstract

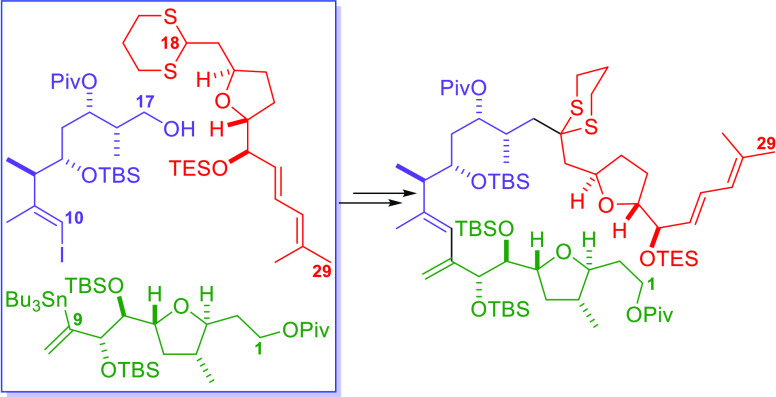

The complete carbon framework of the macrocyclic marine natural product amphidinolide F has been prepared by a convergent synthetic route in which three fragments of similar size and complexity have been coupled. Key features of the syntheses of the fragments include the stereoselective construction of the tetrahydrofuran in the C1–C9 fragment by oxonium ylide (free or metal-bound) formation and rearrangement triggered by the direct generation of a rhodium carbenoid from 1-sulfonyl-1,2,3-triazole, the highly diastereoselective aldol reaction between a boron enolate and an aldehyde with 1,4-control to prepare the C10–C17 fragment, and the formation of the tetrahydrofuran in the C18–C29 fragment by intramolecular nucleophilic ring opening of an epoxide with a hydroxyl group under acidic conditions.

## Introduction

**Figure 1:**
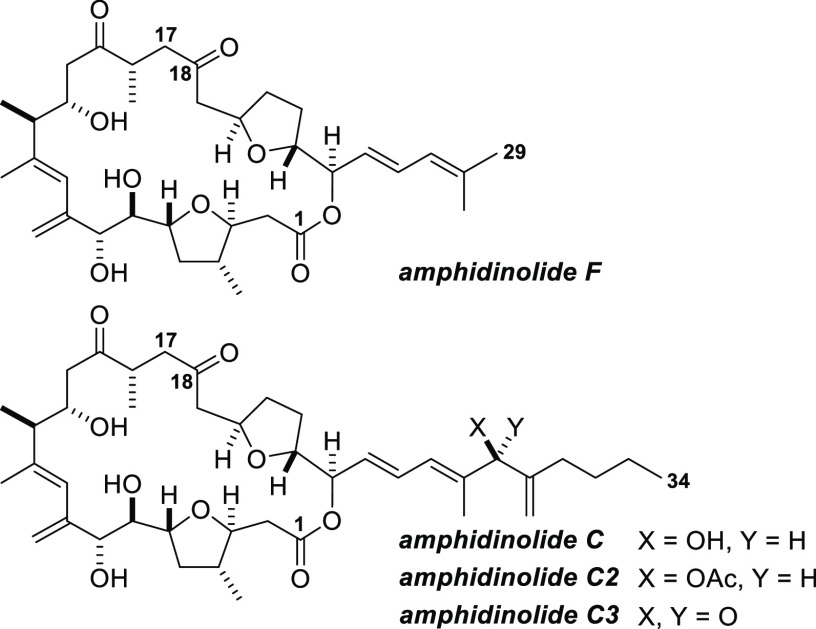
Amphidinolides F, C, C2, and C3.

The cytotoxic marine natural product amphidinolide F was isolated from a dinoflagellate associated with the Okinawan flatworm *Amphiscolops magniviridis* and its structure reported by Kobayashi and co-workers in 1991 (Figure 1).^1^ Amphidinolide F contains a macrolactone that is identical to the core of amphidinolide C,^2^ a natural product isolated by the Kobayashi group and reported in 1988, but it bears a truncated side chain (C25–C29). The natural products amphidinolide C2 and C3 share the same macrolactone core structure but, in common with amphidinolide C, have longer and more elaborate side chains than amphidinolide F (Figure 1).

Amphidinolides F, C, C2, and C3 are cytotoxic agents, but amphidinolide C displays significantly higher activity against certain cancer cell lines (e.g., L1210 murine lymphoma and KB epidermoid carcinoma cells) than any of the other three.^3^ This observation suggests that the hydroxyl group in the side chain of amphidinolide C confers enhanced cytotoxic activity by either hydrogen bonding or covalent binding to its biological target at this site.

The size, stereochemical complexity, and biological activities of amphidinolides F, C, C2, and C3, have made them attractive targets for total synthesis and stimulated the development of new strategies and synthetic methods that permit rapid access to key subunits found in these natural products. Over the past two decades, substantial portions of all four compounds have been synthesized by the groups of Kobayashi (C1–C10; C17–C29),^4^ Armstrong (C18–C29),^5^ Carter (C7–C20),^6^ Dai (C18–C26),^7^ Ferrié (C1–C9),^8^ Forsyth (C1–C9; C11–C25; C1–C14; C15–C25),^9^ Mohapatra (C1–C9; C19–C34),^10^ Morken (C1–C15),^11^ Pagenkopf (C1–C9; C18–C34),^12^ Roush (C1–C9; C11–C29),^13^ Spilling (C1–C9; C18–C29; C18–C34),^14^ and Williams (C10–C25).^15^ These meticulous and extensive synthetic studies have culminated in the recent total syntheses of amphidinolides F and C by the groups of Fürstner and Carter^16,17^ and the total syntheses of amphidinolides F and C2 by the group of Ferrié.^18^

In previous publications, we have reported the synthesis of the C1–C17 fragment of amphidinolides F, C, C2, and C3 and the C18–C34 fragment of amphidinolides of C, C2, and C3.^19^ Our expectation was that the entire carbon skeleton of each natural product would be obtained by the union of two fragments of similar size and complexity through construction of the bond between C17 and C18 (Figure 1). Although our original strategy was both convergent and logical, we were concerned about the number of steps required to prepare each fragment and the somewhat limited options that would be available for fragment coupling to complete the entire carbon framework. We now report the design and implementation of a convergent second-generation synthetic route to the entire carbon framework of amphidinolide F. The new synthetic route is based on the concise and efficient synthesis of three fragments of similar size and complexity and was designed to provide greater flexibility in the final coupling sequence.

**Figure 2:**
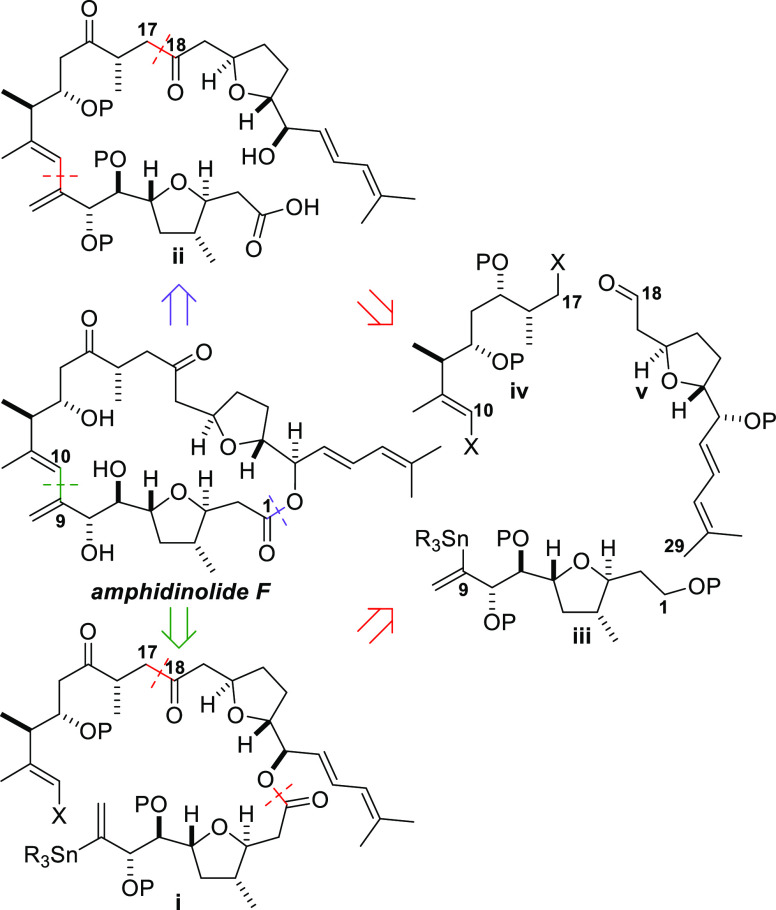
Disconnection of amphidinolide F into the C1–C9, C10–C17, and C18–C29 fragments (**iii**–**v**).

The retrosynthetic analysis of amphidinolide F on which our second-generation synthesis is based is shown in Figure 2. Two primary disconnections by scission of the bond between C9 and C10 (green) or the lactone C–O bond (purple) lead to intermediates (**i** and **ii**, respectively) in which the macrocycle has been opened. Further disconnection of **i** through the ester C–O bond and the C17–C18 bond generates the three key fragments **iii**, **iv**, and **v**. Disconnection of the bond between C9 and C10 and the bond between C17 and C18 in carboxylic acid **ii** leads to the same fragments (**iii**–**v**). This analysis provides flexibility in fragment coupling in the forward direction, with the formation of the macrocycle being accomplished by either a standard macrolactonization reaction or an intramolecular palladium-catalyzed Stille coupling reaction (Figure 2).

## Results and Discussion

Synthetic studies commenced with the construction of the C1–C9 fragment of amphidinolide F. In our previous work, the tandem sequence of copper-catalyzed carbenoid generation, oxonium ylide formation, and rearrangement was used to synthesize an intermediate common to both tetrahydrofuran-containing segments (C1–C7 and C18–C24).^19,20^ However, subsequent elaboration of the C1–C7 unit to give the C1–C9 fragment with the required level of stereocontrol at C7 and C8 proved to be rather inefficient.^19a^ Thus, for the second-generation approach, a highly functionalized chiral pool starting material was selected and the pivotal catalytic carbenoid generation, oxonium ylide formation, and rearrangement reaction was modified so that the substituents at C7 and C8 were present prior to construction of the tetrahydrofuran in the C1–C9 fragment.

**Scheme 1:**
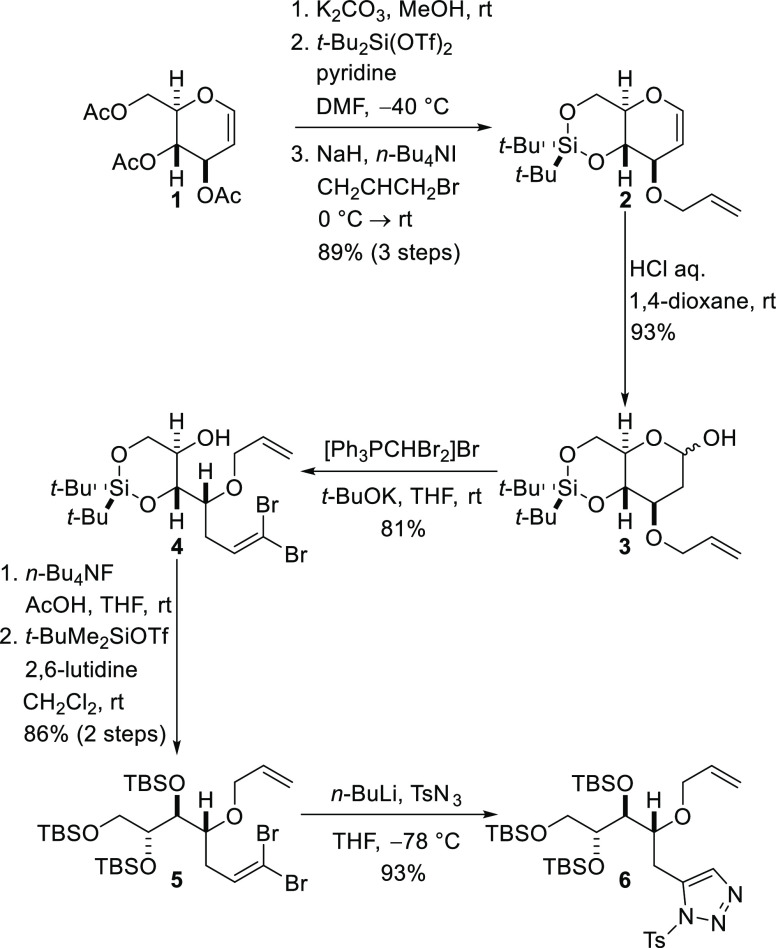
Preparation of the Key Carbenoid Precursor Required for the Synthesis of the C1–C9 Fragment

Synthesis of the C1–C9 fragment of amphidinolide F corresponding to **iii** in the retrosynthetic analysis (Figure 2) began with the high-yielding conversion of commercially available tri-*O*-acetyl-d-glucal (**1**) into allyl ether **2** by sequential ester cleavage, di-*t*-butylsilylene protection of the 1,3-diol, and allylation of the remaining hydroxyl group by deprotonation and *O*-alkylation with allyl bromide (Scheme 1). Acid-mediated hydration of enol ether **2** delivered lactol **3**, and the Ramirez olefination procedure was employed to convert this masked aldehyde into 1,1-dibromoalkene **4**.^21^ Fluoride ion-mediated cleavage of the di-*t*-butylsilylene protecting group followed by *tert*-butyldimethylsilyl (TBS) protection of all three hydroxyl groups of the resulting polar triol intermediate provided the fully protected dibromoalkene **5**. It was essential to buffer the desilylation reaction with acetic acid to avoid decomposition of the dibromoalkene. Treatment of dibromide **5** with *n*-butyllithium resulted in sequential metal–halogen exchange, α-elimination, and rearrangement to produce a lithiated terminal acetylene^22^ that was reacted immediately with tosyl azide to provide the isomerization-prone 1-sulfonyl-1,2,3-triazole **6**,^23^ the precursor required for the key carbenoid reaction, which required rapid purification and careful storage.

**Scheme 2:**
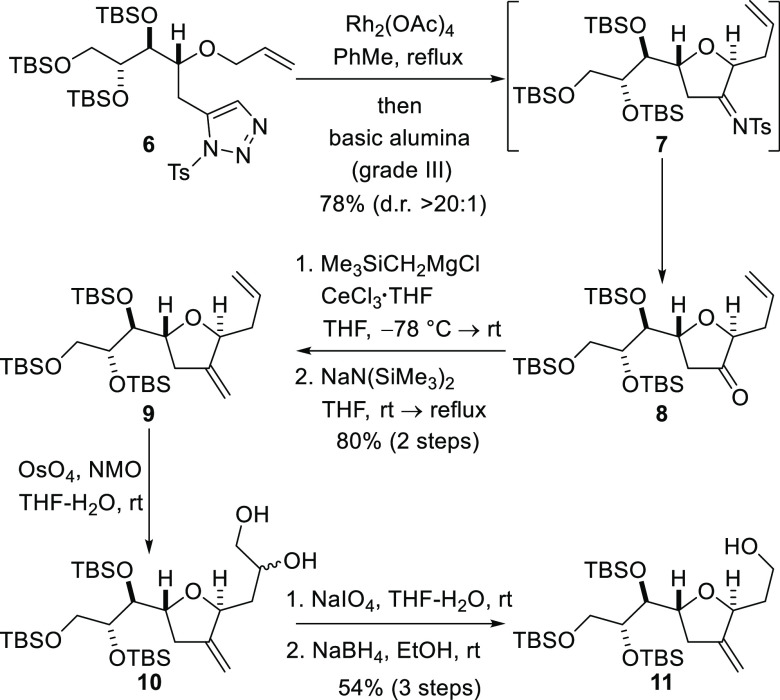
Synthesis of the C1–C9 Fragment by Rearrangement of a Free or Rhodium-Bound Oxonium Ylide

Triazole **6** was converted into dihydrofuranone **8** by reaction with rhodium(II) acetate (1 mol %) in toluene at reflux and treatment of the intermediate product with basic alumina (Brockmann Grade III) according to the procedure devised by Boyer (Scheme 2).^24^ The reaction is presumed to have occurred by rhodium carbenoid generation from the diazo imine formed by Dimroth equilibration of triazole **6**,^25^ followed by oxonium ylide (free or metal-bound) formation and apparent [2,3]-sigmatropic rearrangement. *In situ* hydrolysis of the intermediate imine **7** by exposure to basic alumina afforded ketone **8** in a highly diastereoselective manner (d.r. > 20:1). Ketone **8** was then converted into diene **9** by a Peterson olefination procedure in which the ketone was reacted with the organocerium reagent generated from (trimethylsilyl)methylmagnesium bromide, and the resulting hydroxysilane was treated with sodium bis(trimethylsilyl)amide to effect elimination.^26^ It was necessary to use an organocerium reagent with reduced basicity to avoid epimerization at the site adjacent to the carbonyl group (C3). Selective dihydroxylation of the terminal alkene under standard Upjohn conditions produced 1,2-diol **10** as an inconsequential diastereomeric mixture. Subsequent periodate cleavage of the diol and reduction of the resulting aldehyde provided alcohol **11**.

**Scheme 3:**
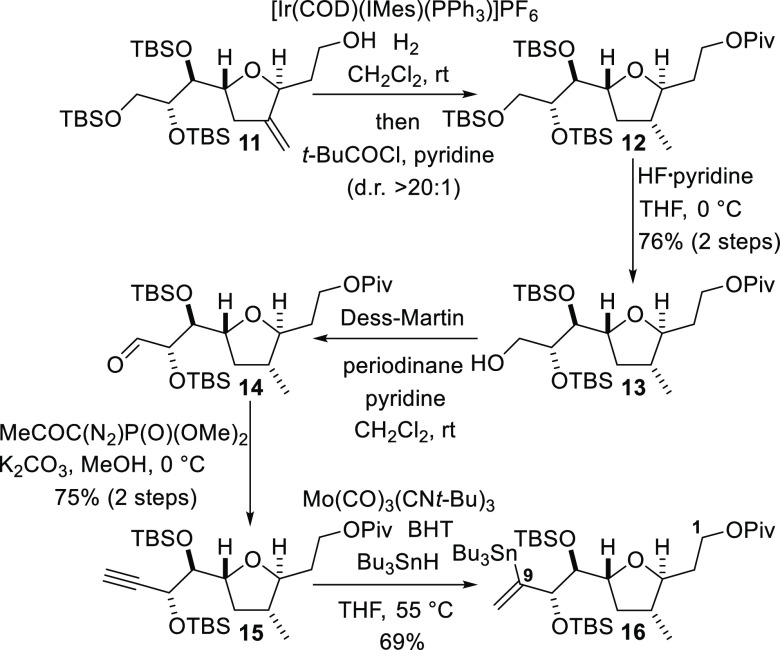
Elaboration of the C1–C9 Fragment to Enable Palladium-Mediated sp^2^–sp^2^ Coupling to the C10–C17 Fragment

Conversion of alcohol **11** into the fully elaborated C1–C9 fragment was accomplished by the reaction sequence shown in Scheme 3. Attempted stereocontrolled conversion of the exocyclic alkene of alcohol **11** into the C4 methyl substituent by hydrogenation in the presence of Crabtree’s catalyst was unsuccessful.^27^ In contrast, rapid and highly diastereoselective directed hydrogenation of the alkene was accomplished when an NHC–iridium(I) complex developed by Kerr and co-workers was employed as the catalyst.^28^ Immediate acylation of the hydroxyl group with pivaloyl chloride afforded ester **12**; hydrogenation and esterification reactions could be performed in a one-pot fashion. Subsequent selective cleavage of a single TBS ether to give primary alcohol **13** was accomplished in good yield by treatment of ester **12** with the hydrogen fluoride pyridine complex at 0 °C. Oxidation of the alcohol to give the corresponding aldehyde **14** was performed by the use of the Dess–Martin protocol, and alkyne **15** was obtained by the use of the Ohira–Bestmann modification^29^ of the Seyferth–Gilbert homologation reaction.^30^ The final step required to complete the C1–C9 fragment was the conversion of alkyne **15** into vinylic stannane **16**. The alkyne hydrostannation protocol developed by Kazmaier and co-workers proved to be uniquely effective for this transformation.^31^ Thus, the treatment of alkyne **15** with tri-*n*-butyltin hydride and a substoichiometric amount (10 mol %) of Mo(CO)_3_(*t*-BuNC)_3_, along with butylated hydroxytoluene (BHT) as a radical inhibitor, at 55 °C in tetrahydrofuran (THF) afforded the required vinylic stannane **16** in a 69% yield as well as a small quantity (16% yield) of the regioisomeric *E*-alkenyl stannane. The isomeric stannanes were readily separable by chromatography on silica gel.

**Scheme 4:**
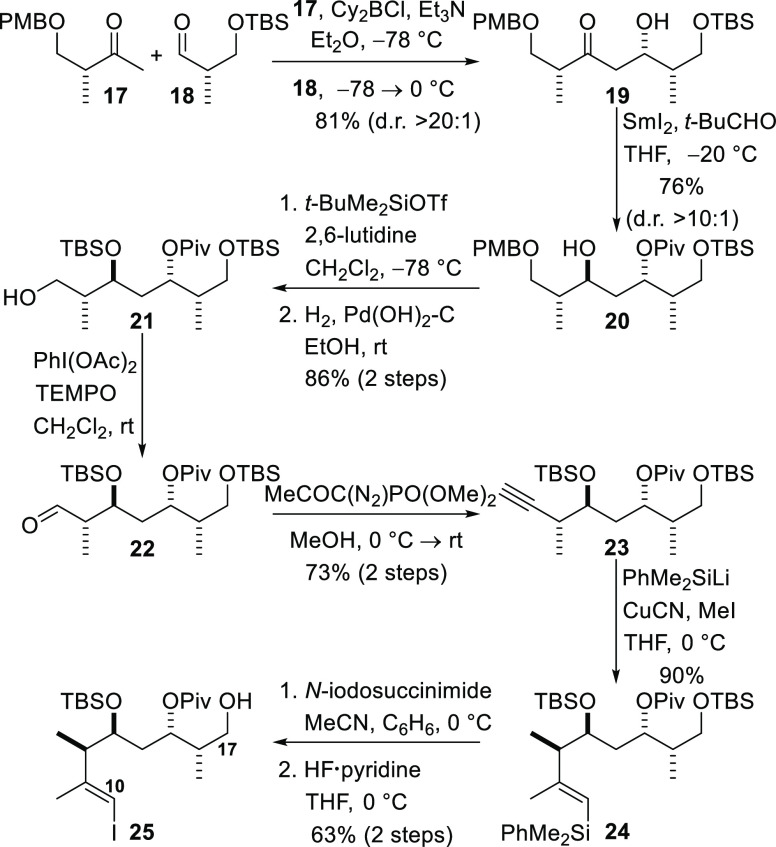
Synthesis of the C10–C17 Fragment by Highly Diastereoselective Aldol Coupling

The synthesis of the C10–C17 fragment corresponding to **iv** in the retrosynthetic analysis (Figure 2) commenced with a diastereoselective aldol reaction between a boron enolate derived from the known methyl ketone **17**(32) and aldehyde **18**, an intermediate that we had used in previous studies concerning the synthesis of the amphidinolides (Scheme 4).^19a^ Thus, the treatment of ketone **17** with dicyclohexylboron chloride and triethylamine produced a boron enolate and subsequent aldol reaction with aldehyde **18** produced β-hydroxyketone **19** in an 81% yield and with >20:1 diastereoselectivity. This result is consistent with the findings of Paterson and co-workers who have reported highly *syn*-selective 1,4-stereoinduction during aldol reactions of enolates generated from the benzyl ether analogue of ketone **17** with aldehydes^33^ and have used a closely related aldol reaction in their synthesis of the marine natural product phorbaside A.^34^ The stereochemical outcome of the reaction and the high level of diastereocontrol can be explained by invoking the model proposed by Paton and Goodman to account for the stereochemical outcome of aldol reactions between aldehydes and boron enolates derived from analogous ketones.^35^

β-Hydroxyketone **19** was then subjected to a highly diastereoselective Evans–Tishchenko reduction reaction with pivaldehyde to produce alcohol **20**.^36^ TBS protection of the free hydroxyl group and hydrogenolytic cleavage of the PMB ether afforded alcohol **21**. Oxidation of the alcohol to give aldehyde **22** was followed by Seyferth–Gilbert homologation according to the Ohira–Bestmann protocol.^29^ The resulting alkyne **23** was converted into alkenylsilane **24** by silylcupration and reaction of the resulting organocopper intermediate with methyl iodide.^37^ Treatment of silane **24** with *N*-iodosuccinimide resulted in the stereoretentive replacement of the silyl group with iodine. Selective fluoride-mediated removal of the TBS group to give a free primary hydroxyl group delivered iodide **25** required for the subsequent Stille coupling to the C1–C9 fragment **16**.

Two fragments corresponding to the C18–C29 unit of amphidinolide F were prepared so that two distinct coupling strategies for construction of the C17–C18 bond could be explored. In previous studies, the tetrahydrofuran had been constructed by the intramolecular reaction of a metal carbenoid with an allyl ether, but for the purposes of the second-generation approach, alternative ring construction methods were explored.

**Scheme 5:**
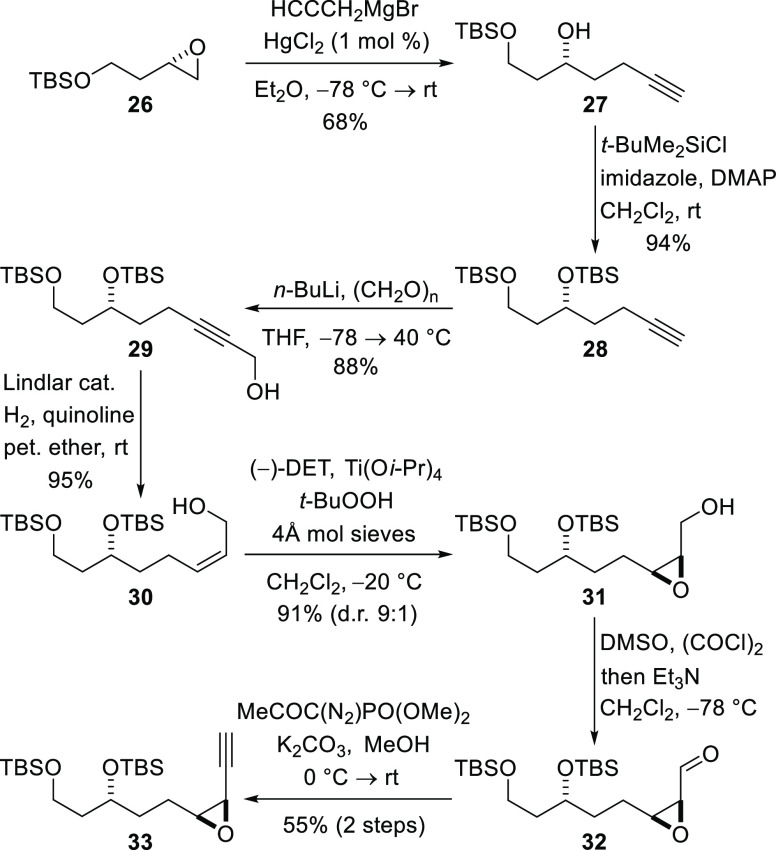
Construction of the Tetrahydrofuran Precursor

The syntheses of both C18–C29 variants commenced with the known epoxide **26**, which can be prepared from d-aspartic acid in three steps.^38^ Epoxide **26** was subjected to nucleophilic ring opening by reaction with propargylmagnesium bromide in the presence of a substoichiometric amount (1 mol %) of mercury(II) chloride to give alcohol **27**,^39^ which was TBS-protected to give alkyne **28** (Scheme 5). Deprotonation of alkyne **28** with *n*-butyllithium and reaction of the resulting anion with formaldehyde afforded propargylic alcohol **29**. Lindlar reduction of the alkyne delivered the *Z*-allylic alcohol **30**, and subsequent Sharpless asymmetric epoxidation, with (−)-diethyl d-tartrate as the ligand,^40^ produced epoxide **31** (d.r. 9:1). Swern oxidation of the alcohol produced aldehyde **32**, and the Ohira–Bestmann protocol was employed immediately to convert this compound into alkyne **33**,^29^ the cyclization precursor.

**Scheme 6:**
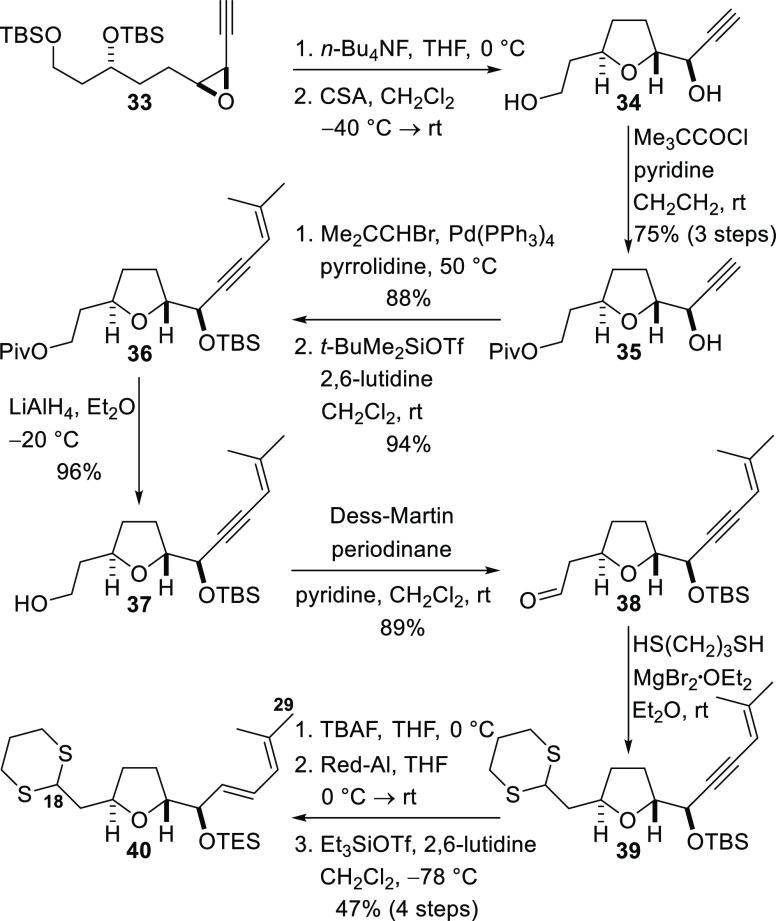
Synthesis of the C18–C29 Dithiane Fragment

Construction of the tetrahydrofuran-containing C18–C29 fragment from epoxide **33** was now investigated (Scheme 6). Fluoride ion-mediated removal of both TBS groups and treatment of the resulting epoxy diol with camphorsulfonic acid in dichloromethane at −40 °C resulted in regioselective intramolecular nucleophilic opening of the epoxide by the secondary alcohol to give the known tetrahydrofuran **34**,^7^ the structure of which was confirmed by comparison of NMR data with that in the literature and by its conversion into the primary *t*-butyldiphenylsilyl ether that had been prepared in our previous studies on the synthesis of amphidinolides C, C2, and C3.^19b^ Immediate acylation of the primary hydroxyl group with pivaloyl chloride then provided the propargylic alcohol **35** in a 75% yield over three steps. A copper-free Sonogashira coupling reaction^41^ was then used to couple the alkyne to 1-bromo-2-methyl-1-propene, and the subsequent TBS protection of the hydroxyl group delivered enyne **36**. Reductive cleavage of the pivaloyl ester, by treatment with lithium aluminum hydride, provided primary alcohol **37**, and this compound was converted into aldehyde **38** by oxidation with the Dess–Martin periodinane. The aldehyde was then treated with 1,3-propanedithiol under Lewis acidic conditions to give dithiane **39**. Sequential cleavage of the TBS ether, stereoselective partial alkyne reduction by treatment with Red-Al to deliver the *E*-allylic alcohol in a highly stereoselective manner, and reprotection of the free hydroxyl group as a triethylsilyl (TES) ether afforded diene **40**, corresponding to C18–C29 of the natural product, in a 47% yield over four steps. This fragment was now ready for coupling to the C1–C17 unit.

**Scheme 7:**
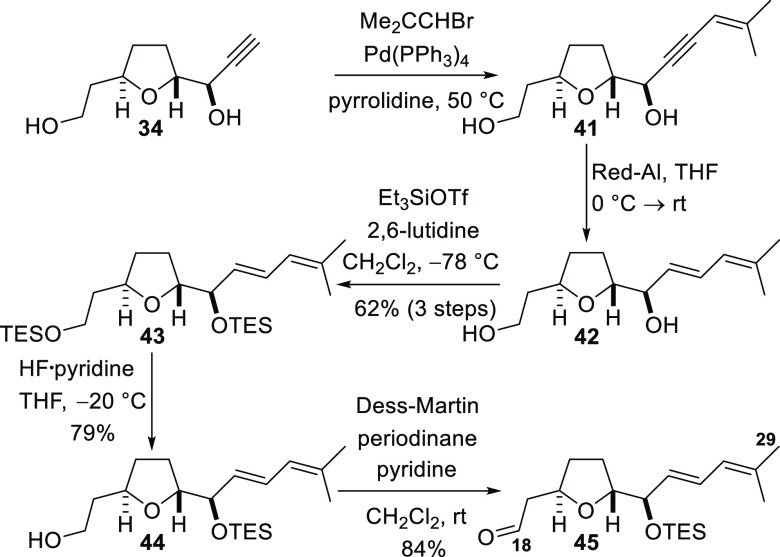
Synthesis of the C18–C29 Aldehyde Fragment

The second C18–C29 fragment was prepared from alkyne **34** by a significantly shorter route than that shown in Scheme 6. Sonogashira coupling of the terminal alkyne to 1-bromo-2-methyl-1-propene afforded enyne **41** (Scheme 7). The propargylic alcohol was then subjected to reduction with Red-Al to deliver *E*-allylic alcohol **42**, and both hydroxyl groups were TES-protected to give diene **43** with an overall yield of 62% over three steps. This diene had been prepared by Kobayashi and co-workers during the synthetic work performed to establish the configuration of stereogenic centers in amphidinolide C, and the data for our sample match those reported in the literature.^4^ Selective cleavage of the primary TES ether to produce alcohol **44** and subsequent oxidation with the Dess–Martin periodinane afforded aldehyde **45**. It should be noted that this aldehyde is the direct TES ether analogue of intermediates prepared by the groups of Armstrong and Ferrié during their studies on the synthesis of amphidinolide F.^5,18^ Direct formation of dithiane **40** by the Lewis acid mediated reaction of aldehyde **45** with 1,3-propanedithiol was attempted to shorten the sequence in Scheme 6, but decomposition of aldehyde **45** was observed.

**Scheme 8:**
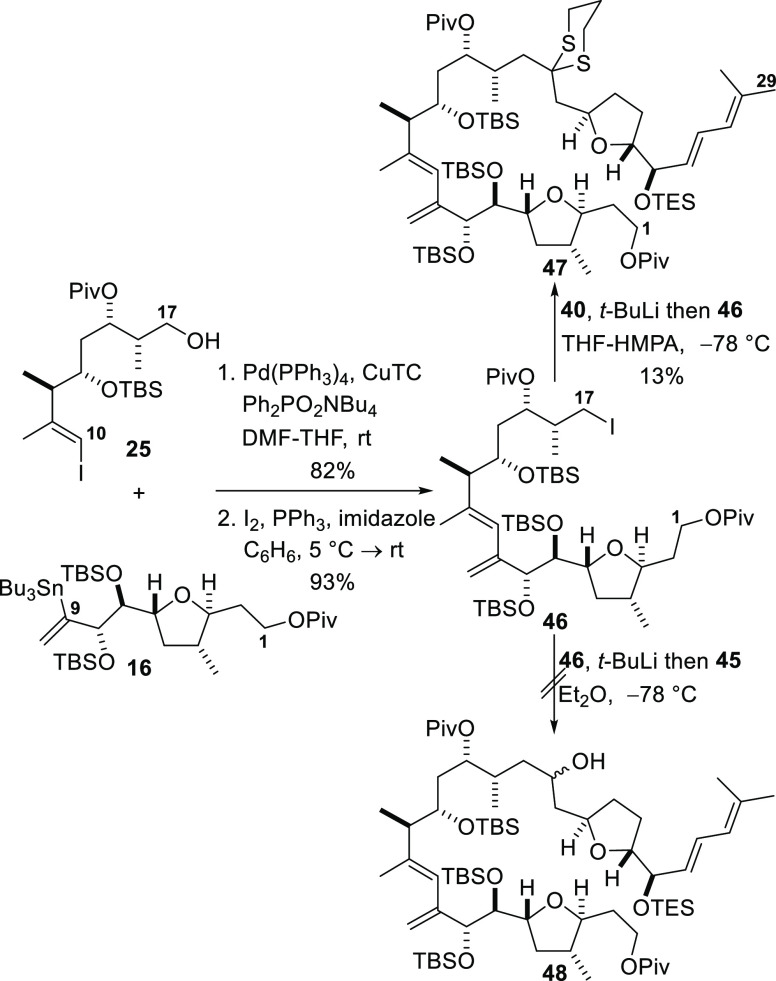
Fragment Coupling to Construct the Complete C1–C29 Framework of Amphidinolide F

The full carbon framework of amphidinolide F was now assembled by coupling of the C1–C9, C10–C17, and C18–C29 fragments (Scheme 8). Alkenyl iodide **25** was first coupled to the vinylic stannane **16** under modified Stille conditions to give the C1–C17 fragment in an 82% yield.^42^ The hydroxyl group at C17 was replaced with iodine under standard iodination conditions to give the iodide **46** in 93% yield. Deprotonation of dithiane **40** with *t*-butyllithium in THF-hexamethylphosphoramide (HMPA) and reaction of the resulting anion with iodide **46** afforded the fully coupled product **47**, a compound that corresponds to the entire C1–C29 framework of amphidinolide F. However, the coupled product was obtained in only 13% yield and significant amounts of both dithiane **40** (42%) and iodide **46** (51%) were recovered from the reaction. Attempts to improve the yield of this coupling reaction were not successful, and the product yield was deemed to be unacceptably low.

To address the issue of incomplete reaction and the resulting low yield obtained when coupling iodide **46** to dithiane **40**, reversal of the polarity of the fragments during the C–C bond-forming reaction was investigated. In this case, aldehyde **45** was reacted with an organolithium reagent generated from iodide **46**. Thus, treatment of iodide **46** with *t*-butyllithium to effect the metal–halogen exchange and addition of the resulting organolithium intermediate to aldehyde **45** was expected to deliver a diastereomeric mixture of alcohols **48**. Unfortunately, treatment of iodide **46** with *t*-butyllithium followed by immediate addition of aldehyde **45** resulted in decomposition of the iodide instead of formation of the required alcohol **48**. Addition of *t*-butyllithium to a mixture of iodide **46** and aldehyde **45** in diethyl ether at −78 °C also failed to deliver the required alcohol **48**.

In summary, the entire carbon framework of amphidinolide F has been assembled by the union of three fragments: stannane **16** (C1–C9), iodide **25** (C10–C17), and dithiane **40** (C18–29). The synthesis of each fragment has been accomplished in a highly stereocontrolled manner. In the case of the C1–C9 fragment, oxonium ylide (free or metal-bound) formation and rearrangement initiated by the generation of a rhodium carbenoid from a 1-sulfonyl-1,2,3-triazole has been used to assemble the tetrahydrofuran with a high level of diastereocontrol at the C3 stereogenic center. The C10–C17 fragment has been assembled by a stereoselective aldol reaction of a boron enolate with 1,4-diastereocontrol followed by an Evans–Tishchenko reduction reaction of the resulting β-hydroxyketone. Efficient construction of the tetrahydrofuran in the C18–C29 fragment has been accomplished by acid-promoted intramolecular 5-*exo* nucleophilic ring opening of an epoxide with a hydroxyl group. Further synthetic studies are required to optimize fragment coupling and complete the synthesis of amphidinolide F. The results of this work will be reported in due course.

## Experimental Section

Reagents were purchased from commercial suppliers and used without purification unless otherwise stated. Air- and moisture-sensitive reactions were performed under an atmosphere of argon in a flame-dried apparatus. Tetrahydrofuran, toluene, acetonitrile, dichloromethane, and diethyl ether were purified using a Pure-SolvTM 500 Solvent Purification System. Petroleum ether used for chromatography was the 40–60 °C fraction. All reactions were monitored by thin-layer chromatography (TLC) using Merck silica gel 60 covered aluminum backed plates F254. TLC plates were visualized under UV light and stained using potassium permanganate solution, acidic ethanolic anisaldehyde solution, or phosphomolybdic acid solution. Flash column chromatography was performed with silica gel (Fluorochem LC60A 35–70 μm or Geduran Si 60 35–70 μm) as solid support. IR spectra were recorded using a Shimadzu FT IR-8400S ATR instrument. The IR spectrum of each compound (solid or liquid) was acquired directly on a thin layer at ambient temperature. ^1^H NMR spectra were recorded on Bruker Avance III 400 and 500 MHz spectrometers at ambient temperature. ^13^C NMR spectra were recorded on Bruker Avance III 400 and 500 MHz spectrometers at 101 and 126 MHz at ambient temperature, respectively. Optical rotations were recorded using an Autopol V polarimeter. High- and low-resolution mass spectra (HRMS) were performed by the use of positive chemical ionization or electron impact ionization on a Jeol MStation JMS-700 instrument or by the use of positive or negative ion electrospray techniques on a Bruker micrOTOF-Q instrument. Elemental analyses were carried out on an Exeter Analytical Elemental Analyser EA 440. Melting points were recorded with an Electrothermal IA 9100 apparatus.

### (4a*R*,8*R*,8a*S*)-2,2-Di-*tert*-butyl-8-(2-propen-1-yloxy)-4,4a,8,8a-tetrahydropyrano[3,2-*d*][1,3,2]dioxasilin (**2**)

To a solution of tri-*O*-acetyl-d-glucal (10.0 g, 36.7 mmol) in MeOH (80 mL) at room temperature (rt) was added potassium carbonate (51 mg, 0.37 mmol). The reaction mixture was stirred at rt for 16 h and then concentrated under reduced pressure to give the crude d-glucal, which was used in the next step without purification. To a solution of d-glucal in *N*,*N*-dimethylformamide (DMF, 88 mL) at −40 °C was added di-*tert*-butylsilyl bis(trifluoromethanesulfonate) (13.1 mL, 40.2 mmol) by a syringe pump for 1 h. After complete addition of the silylating agent, the reaction mixture was stirred at −40 °C for 2 h. The reaction was quenched by the addition of pyridine (8 mL), and the mixture was diluted with diethyl ether (100 mL) and water (100 mL). The phases were separated, and the aqueous phase was extracted with diethyl ether (2 × 100 mL). The combined organic extracts were washed with saturated aqueous sodium bicarbonate (100 mL) and brine (100 mL), dried (anhydrous MgSO_4_), filtered, and concentrated under reduced pressure to deliver the crude allylic alcohol, which was used in the next step without further purification. To a solution of alcohol in DMF (100 mL) at 0 °C was added sodium hydride (2.20 g of a 60% dispersion in mineral oil, 55.0 mmol) portionwise, and the resulting mixture was stirred at 0 °C for 10 min during which time hydrogen evolution ceased. Allyl bromide (16.0 mL, 185 mmol) was added followed by the addition of tetra-*n*-butylammonium iodide (1.36 g, 3.68 mmol). The mixture was then allowed to warm to rt and stirred at this temperature for 18 h. The reaction was quenched by the addition of saturated aqueous ammonium chloride (100 mL) and extracted with diethyl ether (3 × 200 mL). The combined organic extracts were washed with saturated aqueous lithium chloride (100 mL), dried (anhydrous MgSO_4_), filtered, and concentrated under reduced pressure. The residue was purified by flash chromatography on silica gel (pet. ether-diethyl ether, 100:1) to afford allyl ether **2** (10.7 g, 89% over three steps) as a colorless oil. *R_f_* = 0.24 (pet. ether-diethyl ether, 50:1); [α]_D_^31^ −28 (*c* = 2.0, CHCl_3_); ν_max_. 2963, 2934, 2889, 2860, 1647, 869, 826, 768, 653 cm^–1^; ^1^H NMR (500 MHz, CDCl_3_) δ 6.27 (1H, ddd, *J* = 6.1, 1.8, 0.4 Hz), 5.94 (1H, ddt, *J* = 17.2, 10.4, 5.5 Hz), 5.31 (1H, dq, *J* = 17.2, 1.5 Hz), 5.17 (1H, dq, *J* = 10.4, 1.5 Hz), 4.71 (1H, dd, *J* = 6.1, 1.8 Hz), 4.36 (1H, ddt, *J* = 13.1, 5.5, 1.5 Hz), 4.23 (1H, ddt, *J* = 13.1, 5.5, 1.5 Hz), 4.15 (1H, dd, *J* = 10.4, 5.0 Hz), 4.12 (1H, dd, *J* = 10.4, 7.0 Hz), 4.08 (1H, dt, *J* = 7.0, 1.8 Hz), 3.96 (1H, t, *J* = 10.4 Hz), 3.81 (1H, td, *J* = 10.4, 5.0 Hz), 1.07 (9H, s), 1.00 (9H, s); ^13^C{^1^H} NMR (126 MHz, CDCl_3_) δ 144.2, 135.6, 116.8, 102.8, 77.0, 76.7, 72.9, 71.5, 66.2, 27.7, 27.2, 22.9, 20.1; LRMS (CI, isobutane) *m*/*z* (intensity) 326.9 [M + H]^+^ (86), 268.9 (100); HRMS (ESI+) *m*/*z*: [M + Na]^+^ calcd for C_17_H_30_NaO_4_Si 349.1806; found 349.1801. Anal. calcd for C_17_H_30_O_4_Si: C, 62.54%; H, 9.26%, found: C, 62.51%; H, 9.39%.

### (4a*R*,8*R*,8a*S*)-2,2-Di-*tert*-butyl-8-(2-propen-1-yloxy)-4,4a,8,8a-tetrahydropyrano[3,2-*d*][1,3,2]dioxasilin-6-ol (**3**)

To a solution of enol ether **2** (13 g, 40 mmol) in 1,4-dioxane (470 mL) at rt was added 8 M aqueous HCl (99.5 mL), and the reaction mixture was stirred at rt for 3 h. The reaction was diluted with dichloromethane (650 mL) and saturated aqueous sodium bicarbonate (650 mL). The phases were separated, and the aqueous phase was extracted with dichloromethane (2 × 500 mL). The combined organic extracts were washed with brine (500 mL), dried (anhydrous MgSO_4_), filtered, and concentrated under reduced pressure. The residue was purified by flash chromatography on silica gel (pet. ether-ethyl acetate, 9:1) to give lactol **3** (12.8 g of a 3:1 mixture of anomers, 93%) as a colorless solid. *R_f_* = 0.11 (pet. ether-ethyl acetate, 9:1); [α]_D_^30^ +22 (*c* = 1.0, CHCl_3_); mp = 85–86 °C; ^1^H NMR (400 MHz, CDCl_3_) δ 5.99–5.87 (2H, m, 1 major + 1 minor), 5.34–5.25 (3H, m, 2 major + 1 minor), 5.16 (1H, ddt, *J* = 10.4, 1.6, 1.4 Hz, minor), 5.15 (1H, ddt, *J* = 10.4, 1.8, 1.3 Hz, major), 4.85 (1H, ddd, *J* = 9.5, 6.8, 2.1 Hz, minor), 4.40 (1H, ddt, *J* = 13.0, 5.7, 1.4 Hz, major), 4.40 (1H, ddt, *J* = 12.9, 5.4, 1.4 Hz, minor), 4.23 (1H, ddt, *J* = 13.0, 5.7, 1.4 Hz, major), 4.23 (1H, ddt, *J* = 13.0, 5.7, 1.4 Hz, minor), 4.13 (1H, dd, *J* = 10.2, 5.0 Hz, minor), 4.04 (1H, dd, *J* = 9.5, 4.5 Hz, major), 3.98–3.91 (2H, m, 1 major + 1 minor), 3.90–3.68 (4H, m, 3 major + 1 minor), 3.44 (1H, ddd, *J* = 11.6, 8.4, 5.0 Hz, minor), 3.38–3.31 (2H, m, minor), 2.86 (1H, dd, *J* = 2.9, 2.4 Hz, major) 2.27 (1H, ddd, *J* = 12.9, 5.0, 2.1 Hz, minor), 2.14 (1H, ddd, *J* = 13.3, 4.6, 1.2 Hz, major), 1.71–1.61 (1H, m, major), 1.59–1.49 (1H, m, minor), 1.06 (9H, s, major), 1.06 (9H, s, minor), 1.01 (9H, s, major), 0.99 (9H, s, minor); ^13^C{^1^H} NMR (101 MHz, CDCl_3_) δ 135.7 (major), 135.5 (minor), 116.7 (minor), 116.6 (major), 94.7 (minor), 92.6 (major), 80.4 (major), 79.3 (minor), 75.1 (major), 72.8 (major), 72.4 (minor), 71.1 (minor), 67.2 (minor), 67.2 (major), 67.1 (major), 66.7 (minor), 39.2 (minor), 36.5 (major), 27.6 (major), 27.6 (minor), 27.2 (major), 27.2 (minor), 22.8 (major), 22.8 (minor), 20.1 (major), 20.1 (minor); ν_max_. 3407, 2963, 2934, 2888, 2860, 978, 919, 858, 826, 769, 735, 652 cm^–1^; LRMS (EI^+^) *m*/*z* (intensity) 344.0 [M]^+^ (12), 287.0 (44), 257.0 (76), 215.0 (100), 173 (86), 161.0 (42); HRMS (EI^+^) *m*/*z*: [M]^+^ calcd for C_17_H_32_O_5_Si 344.2019; found 344.2018. Anal. calcd for C_17_H_32_O_5_Si: C, 59.27%; H, 9.36%, found: C, 59.17%; H, 9.62%.

### (4*R*,5*R*)-4-[(*R*)-4,4-Dibromo-1-(2-propen-1-yloxy)-but-3-en-1-yl]-2,2-di-*tert*-butyl-1,3-dioxa-2-silacyclohexan-5-ol (**4**)

A solution of potassium *t*-butoxide (7.30 g, 65.1 mmol) in THF (400 mL) was added to a suspension of [Ph_3_PCHBr_2_]Br (35.9 g, 69.7 mmol) in THF (300 mL) at rt. The resulting mixture was stirred at rt for 30 min, during which time the color turned from yellow to brown. A solution of lactol **3** (8.00 g, 23.2 mmol) in THF (100 mL) was added, and the reaction was stirred at rt for 16 h. The reaction was quenched by the addition of saturated aqueous ammonium chloride (800 mL), and the mixture was extracted with diethyl ether (3 × 500 mL). The combined organic extracts were washed with brine (500 mL), dried (anhydrous MgSO_4_), filtered, and concentrated under reduced pressure. The residue was purified by flash chromatography on silica gel (pet. ether-ethyl acetate, 19:1) to afford dibromoalkene **4** (9.41 g, 81%) as a colorless oil. *R_f_* = 0.31 (pet. ether-ethyl acetate, 9:1); [α]_D_^28^ −13 (*c* = 1.9, CHCl_3_); ν_max_. 3429, 2961, 2933, 2891, 2860, 856, 826, 773, 652 cm^–1^; ^1^H NMR (500 MHz, CDCl_3_) δ 6.56 (1H, t, *J* = 7.2 Hz), 5.89 (1H, ddt, *J* = 17.2, 10.5, 5.7 Hz), 5.30 (1H, dq, *J* = 17.2, 1.4 Hz), 5.22 (1H, dq, *J* = 10.5, 1.4 Hz), 4.14 (1H, ddt, *J* = 12.7, 5.7, 1.4 Hz), 4.11 (1H, dd, *J* = 10.5, 4.2 Hz), 4.07 (1H, ddt, *J* = 12.7, 5.7, 1.4 Hz), 3.98–3.90 (2H, m), 3.79 (1H, t, *J* = 10.5 Hz), 3.73 (1H, ddd, *J* = 7.7, 5.0, 2.6 Hz), 2.92 (1H, s), 2.61 (1H, ddd, *J* = 15.0, 7.2, 5.0 Hz), 2.43 (1H, ddd, *J* = 15.0, 7.7, 7.2 Hz), 1.04 (9H, s), 0.99 (9H, s); ^13^C{^1^H} NMR (126 MHz, CDCl_3_) δ 135.4, 134.3, 117.9, 90.3, 78.8, 76.8, 71.9, 68.7, 67.1, 33.4, 27.7, 27.2, 22.9, 20.3; LRMS (CI, isobutane) *m*/*z* (intensity) 501.0 [M + H]^+^ (100), 443.0 (30), 421.0 (33), 341.0 (25), 201.0 (15), 177.0 (13); HRMS (ESI+) *m*/*z*: [M + Na]^+^ calcd for C_18_H_32_NaO_4_SiBr_2_^79^ 521.0329; found 521.0315. Anal. calcd for C_18_H_32_O_4_SiBr_2_: C, 43.21%; H, 6.45%, found: C, 42.66%; H, 6.50%.

### (5*S*,6*R*)-5-[(*R*)-4,4-Dibromo-1-(2-propen-1-yloxy)but-3-en-1-yl]-6-(*tert*-butyldimethylsilyloxy)-2,2,3,3,9,9,10,10-octamethyl-4,8-dioxa-3,9-disilaundecane (**5**)

To a solution of silyl ether **4** (9.01 g, 18.0 mmol) in THF (280 mL) at rt were added sequentially acetic acid (2.06 mL, 36.0 mmol) and tetra-*n*-butylammonium fluoride (72.0 mL of a 1.0 M solution in THF, 72.0 mmol). The reaction mixture was stirred at rt for 14 h and then concentrated under reduced pressure. The residue was filtered through a short pad of silica gel (pet. ether-ethyl acetate, 1:2) to afford the crude triol as a pale yellow solid. The triol was dissolved in dichloromethane (280 mL), and *tert*-butyldimethylsilyl trifluoromethanesulfonate (20.7 mL, 90.1 mmol) and 2,6-lutidine (16.8 mL, 144 mmol) were sequentially added at rt. The mixture was stirred at rt for 3 h, and the reaction was quenched by the addition of saturated aqueous sodium bicarbonate (280 mL). The mixture was extracted with diethyl ether (3 × 400 mL), and the combined organic extracts were washed with saturated aqueous copper(II) sulfate (400 mL), dried (anhydrous MgSO_4_), filtered, and concentrated under reduced pressure. The residue was purified by flash chromatography on silica gel (pet. ether-ethyl acetate, 200:1) to give silyl ether **5** (10.9 g, 86% over two steps) as a colorless oil. *R_f_* = 0.90 (pet. ether-ethyl acetate, 19:1); [α]_D_^26^ −3.4 (*c* = 2.0, CHCl_3_); ν_max_. 2954, 2929, 2886, 2857, 2360, 2341, 831, 774 cm^–1^; ^1^H NMR (400 MHz, CDCl_3_) δ 6.54 (1H, dd, *J* = 7.2, 6.4 Hz), 5.89 (1H, ddt, *J* = 17.2, 10.4, 5.7 Hz), 5.25 (1H, dq, *J* = 17.2, 1.5 Hz), 5.16 (1H, dq, *J* = 10.4, 1.5 Hz), 4.08 (1H, ddt, *J* = 12.5, 5.7, 1.5 Hz), 3.96 (1H, ddt, *J* = 12.5, 5.7, 1.5 Hz), 3.82–3.76 (3H, m), 3.49–3.42 (2H, m), 2.46 (1H, ddd, *J* = 16.0, 6.4, 4.2 Hz), 2.37 (1H, dt, *J* = 16.0, 7.2 Hz), 0.90 (9H, s), 0.90 (9H, s), 0.89 (9H, s), 0.09 (3H, s), 0.09 (3H, s), 0.08 (3H, s), 0.07 (3H, s), 0.05 (6H, s); ^13^C{^1^H} NMR (101 MHz, CDCl_3_) δ 136.0, 135.2, 117.1, 89.5, 79.4, 76.5, 75.4, 71.6, 64.7, 34.8, 26.2, 26.2, 26.2, 18.5, 18.5, 18.4, −4.2, −4.4, −4.5, −4.5, −5.3, −5.3; LRMS (CI, isobutane) *m*/*z* (intensity) 703.0 [M + H]^+^ (100), 289.0 (64); HRMS (ESI+) *m*/*z*: [M + Na]^+^ calcd for C_28_H_58_NaO_4_Si_3_Br_2_^79^ 723.1902; found 723.1876. Anal. calcd for C_28_H_58_O_4_Si_3_Br_2_: C, 47.85%; H, 8.32%, found: C, 48.24%; H, 8.49%.

### 5-[(2*R*,3*S*,4*R*)-2-(Propen-1-yloxy)-3,4,5-tris(*tert*-butyl-dimethylsilyloxy)pentyl]-1-(4-methylbenzene-1-sulfonyl)-1*H*-1,2,3-triazole (**6**)

*n*-Butyllithium (6.83 mL of a 2.5 M solution in hexanes, 17.1 mmol) was added dropwise to a solution of dibromoalkene **5** (6.00 g, 8.54 mmol) in THF (42 mL) at −78 °C. The reaction mixture was stirred at −78 °C for 20 min, and then *p*-toluenesulfonyl azide (4.48 mL of a 2.0 M solution in THF, 8.96 mmol) was added. The resulting mixture was stirred at −78 °C for a further 30 min, and the reaction was quenched by the addition of saturated aqueous ammonium chloride (40 mL). The mixture was warmed to rt and then extracted with diethyl ether (3 × 40 mL). The combined organic extracts were dried (anhydrous MgSO_4_), filtered, and concentrated under reduced pressure. The residue was purified by rapid flash chromatography on silica gel (pet. ether-ethyl acetate, 10:1) to give triazole **6** (5.89 g, 93%) as a colorless oil. *R_f_* = 0.28 (pet. ether-ethyl acetate, 9:1); [α]_D_^27^ +20 (*c* = 2.2, CHCl_3_); ν_max_. 2955, 2929, 2885, 2857, 834, 777, 668 cm^–1^; ^1^H NMR (500 MHz, CDCl_3_) δ 7.95 (2H, d, *J* = 8.3 Hz), 7.54 (1H, s), 7.35 (2H, d, *J* = 8.3 Hz), 5.56 (1H, ddt, *J* = 17.2, 10.5, 5.7 Hz), 5.08 (1H, dq, *J* = 17.2, 1.4 Hz), 5.03 (1H, dq, *J* = 10.5, 1.4 Hz), 4.01 (1H, dd, *J* = 5.7, 1.3 Hz), 3.98 (1H, ddt, *J* = 12.5, 5.7, 1.4 Hz), 3.88 (1H, td, *J* = 6.2, 1.3 Hz), 3.82 (1H, dd, *J* = 10.0, 6.2 Hz), 3.73 (1H, ddt, *J* = 12.5, 5.7, 1.4 Hz), 3.68 (1H, dt, *J* = 12.2, 5.7 Hz), 3.52 (1H, dd, *J* = 10.0, 6.2 Hz), 3.38–3.30 (2H, m), 2.44 (3H, s), 0.92 (9H, s), 0.91 (9H, s), 0.88 (9H, s), 0.13 (3H, s), 0.10 (3H, s), 0.07 (3H, s), 0.06 (3H, s), 0.06 (3H, s), 0.05 (3H, s); ^13^C{^1^H} NMR (126 MHz, CDCl_3_) δ 146.9, 138.2, 134.4, 134.2, 134.0, 130.3, 128.8, 117.3, 79.7, 76.2, 74.7, 71.8, 65.0, 26.2, 26.1, 26.1, 25.8, 21.9, 18.5, 18.4, 18.3, −4.2, −4.5, −4.6, −4.6, −5.3, −5.3; HRMS (ESI+) *m*/*z*: calcd for C_35_H_65_N_3_NaO_6_Si_3_S [M + Na]^+^ 762.3794; found 762.3774.

### (2*S*,5*R*)-5-[(5*S*,6*R*)-6-(*tert*-Butyldimethylsilyloxy)-2,2,3,3,9,9,10,10-octamethyl-4,8-dioxa-3,9-disilaundecan-5-yl]-2-(2-propen-1-yl)dihydrofuran-3(2*H*)-one (**8**)

Rhodium(II) acetate (42.0 mg, 94.6 μmol) was added to a solution of triazole **6** (7.00 g, 9.46 mmol) in toluene (400 mL). The resulting mixture was heated (oil bath) to reflux and stirred at this temperature for 1 h. The mixture was then cooled to rt, and basic alumina (Brockmann activity III, 95 g) was added. The resulting mixture was stirred at rt for a further 30 min and then filtered. The filtrate was concentrated under reduced pressure, and the resulting residue was purified by flash chromatography on silica gel (pet. ether-ethyl acetate, 50:1) to afford dihydrofuranone **8** (4.13 g, 78%, d.r. > 20:1) as a colorless oil. *R_f_* = 0.78 (pet. ether-ethyl acetate, 9:1); [α]_D_^19^ −36 (*c* = 2.5, CHCl_3_); ν_max_. 2953, 2929, 2886, 2858, 1761, 832, 776 cm^–1^; ^1^H NMR (400 MHz, CDCl_3_) δ 5.80 (1H, ddt, *J* = 17.2, 10.2, 7.0 Hz), 5.12 (1H, dq, *J* = 17.2, 1.5 Hz), 5.07 (1H, dq, *J* = 10.2, 1.5 Hz), 4.49 (1H, dt, *J* = 7.5, 6.3 Hz,), 4.03 (1H, dd, *J* = 7.5, 4.5 Hz), 3.83 (1H, dd, *J* = 9.5, 5.5 Hz), 3.79 (1H, td, *J* = 5.5, 2.0 Hz), 3.74 (1H, dd, *J* = 6.3, 2.0 Hz), 3.46 (1H, dd, *J* = 9.5, 5.5 Hz), 2.48 (1H, dd, *J* = 18.2, 7.5 Hz), 2.45–2.38 (1H, m), 2.34 (1H, dd, *J* = 18.2, 6.3 Hz), 2.34–2.21 (1H, m), 0.89 (9H, s), 0.88 (9H, s), 0.87 (9H, s), 0.10 (3H, s), 0.08 (3H, s), 0.07 (6H, s), 0.05 (3H, s), 0.04 (3H, s); ^13^C{^1^H} NMR (101 MHz, CDCl_3_) δ 215.9, 133.4, 118.2, 79.5, 78.9, 76.8, 76.1, 64.6, 39.5, 35.9, 26.2, 26.1, 26.1, 18.5, 18.4, 18.4, −4.1, −4.3, −4.4, −4.6, −5.2, −5.2; HRMS (ESI+) *m*/*z*: [M + Na]^+^ calcd for C_28_H_58_NaO_5_Si_3_ 581.3484; found 581.3467.

### (5*S*,6*R*)-6-(*tert*-Butyldimethylsilyloxy)-5-[(2*R*,5*S*)-4-methylene-5-(2-propen-1-yl)tetrahydrofuran-2-yl]-2,2,3,3,9,9,10,10-octamethyl-4,8-dioxa-3,9-disilaundecane (**9**)

Cerium(III) chloride heptahydrate (6.74 g, 18.1 mmol) was added to a 100 mL round-bottomed flask and dried under vacuum at 120 °C (temperature increased gradually from rt in an oil bath) for 2 h, then at 140 °C for 2 h, and at 160 °C for a further 2 h. The flask was allowed to cool to rt and was purged with argon (10 min). THF (23 mL) was added, and the mixture was stirred at rt for 2 h under argon to give the cerium(III) chloride–THF complex as a white precipitate. A solution of (chloromethyl)trimethylsilane (2.53 mL, 18.1 mmol) in THF (10 mL) was added dropwise to a suspension of magnesium turnings (401 mg, 16.5 mmol) and 1,2-dibromoethane (2 drops) in THF (4 mL). Formation of the Grignard reagent was accomplished by heating (oil bath) the mixture to reflux, followed by the slow addition of (chloromethyl)trimethylsilane to maintain reflux. The Grignard reagent was stirred at rt for 2 h and then added to the cerium(III) chloride–THF complex at −78 °C. The resulting gray mixture was stirred at −78 °C for 30 min, and then a solution of ketone **8** (2.30 g, 4.13 mmol) in THF (4 mL) was added. The reaction mixture was stirred at −78 °C for 1 h, and the flask was then removed from the cold bath. The mixture was stirred at rt for a further period of 18 h and then cooled to 0 °C. Saturated aqueous ammonium chloride (10 mL) was added at 0 °C, and the mixture was stirred for 20 min. Water (40 mL) was added, and the mixture was extracted with diethyl ether (3 × 50 mL). The combined organic extracts were washed with brine (30 mL), dried (anhydrous MgSO_4_), filtered, and concentrated under reduced pressure to give the crude tertiary alcohol as a pale yellow oil. The crude alcohol was dissolved in THF (45 mL) at rt, and sodium bis(trimethylsilyl)amide (8.25 mL of a 2.0 M solution in THF, 16.5 mmol) was added for 30 s. The solution was stirred at rt for 5 min and then heated (oil bath) to reflux for 1.5 h. The mixture was then cooled to rt and quenched by the addition of saturated aqueous ammonium chloride (50 mL). The biphasic mixture was diluted with diethyl ether (50 mL) and water (20 mL), the layers were separated, and the aqueous layer was extracted with diethyl ether (2 × 50 mL). The combined organic extracts were dried (anhydrous MgSO_4_), filtered, and concentrated under reduced pressure. The residue was purified by flash chromatography on silica gel (pet. ether-ethyl acetate, 200:1) to afford diene **9** (1.83 g, 80% over two steps) as a colorless oil. *R_f_* = 0.82 (pet. ether-ethyl acetate, 40:1); [α]_D_^24^ −39 (*c* = 1.1, CHCl_3_); ν_max_. 2953, 2929, 2886, 2857, 831, 774 cm^–1^; ^1^H NMR (400 MHz, CDCl_3_) δ 5.87 (1H, ddt, *J* = 17.2, 10.2, 5.9 Hz), 5.08 (1H, dq, *J* = 17.2, 1.6 Hz), 5.05 (1H, dq, *J* = 10.2, 1.6 Hz), 4.97 (1H, q, *J* = 2.1 Hz,), 4.83 (1H, q, *J* = 2.1 Hz), 4.44–4.39 (1H, m), 4.07 (1H, q, *J* = 7.2 Hz), 3.82 (1H, dd, *J* = 9.9, 5.9 Hz), 3.69 (1H, td, *J* = 5.9, 1.5 Hz), 3.63 (1H, dd, *J* = 7.2, 1.5 Hz), 3.44 (1H, dd, *J* = 9.9, 5.9 Hz), 2.60 (1H, ddt, *J* = 15.5, 7.2, 2.1 Hz), 2.40–2.32 (1H, m), 2.32–2.22 (2H, m), 0.89 (9H, s), 0.89 (9H, s), 0.88 (9H, s), 0.08 (3H, s), 0.07 (3H, s), 0.07 (3H, s), 0.06 (3H, s), 0.04 (6H, s); ^13^C{^1^H} NMR (101 MHz, CDCl_3_) δ 151.5, 135.3, 116.9, 104.8, 79.4, 79.3, 79.0, 75.7, 64.3, 40.0, 36.0, 26.2, 26.2, 26.2, 18.6, 18.5, 18.4, −4.3, −4.4, −4.5, −4.5, −5.3, −5.3; HRMS (ESI+) *m*/*z*: [M + Na]^+^ calcd for C_29_H_60_NaO_4_Si_3_ 579.3692; found 579.3666.

### 2-{(2*S*,5*R*)-5-[(5*S*,6*R*)-6-(*tert*-Butyldimethylsilyloxy)-2,2,3,3,9,9,10,10-octamethyl-4,8-dioxa-3,9-disilaundecan-5-yl]-3-methylenetetrahydrofuran-2-yl}ethanol (**11**)

Osmium tetroxide (730 μL of a 2.5% solution in *t*-butanol, ca. 0.07 mmol) was added to a solution of diene **9** (2.00 g, 3.59 mmol) and *N*-methylmorpholine-*N*-oxide (505 mg, 4.31 mmol) in a 10:1 mixture of THF and water (49.5 mL) at rt. The solution was stirred at rt for 16 h, and then the reaction was quenched by the addition of solid sodium sulfite (1.8 g). The mixture was stirred at rt for 30 min before dichloromethane (80 mL) and water (50 mL) were added. The phases were separated, and the aqueous phase was extracted with dichloromethane (2 × 50 mL). The combined organic extracts were dried (anhydrous MgSO_4_), filtered, and concentrated under reduced pressure to afford the crude diol **10**, which was subjected to oxidative diol cleavage without purification. Sodium periodate (1.54 g, 7.20 mmol) was added to a stirred solution of the crude diol 10 in a mixture of THF and water (4:1, 50 mL) at rt. The mixture was stirred at rt for 1.5 h, diluted with water (40 mL), and extracted with diethyl ether (3 × 40 mL). The combined organic extracts were dried (anhydrous MgSO_4_), filtered, and concentrated under reduced pressure to deliver the crude aldehyde, which was subjected to reduction without purification. The crude aldehyde was dissolved in ethanol (36 mL) at rt, and sodium borohydride (143 mg, 3.78 mmol) was added. The mixture was stirred at rt for 1 h and then concentrated under reduced pressure. Dichloromethane (20 mL) and water (20 mL) were added, and the phases were separated. The aqueous phase was extracted with further dichloromethane (2 × 20 mL), and the combined organic extracts were dried (anhydrous MgSO_4_), filtered, and concentrated under reduced pressure. The residue was purified by flash chromatography on silica gel (pet. ether-ethyl acetate, 19:1) to yield alcohol **11** (1.09 g, 54% over three steps) as a colorless oil. *R_f_* = 0.14 (pet. ether-ethyl acetate, 19:1); [α]_D_^24^ −23 (*c* = 1.0, CHCl_3_); ν_max_. 3409, 2953, 2929, 2886, 2857, 831, 774, 668 cm^–1^; ^1^H NMR (500 MHz, CDCl_3_) δ 4.98 (1H, q, *J* = 2.0 Hz), 4.81 (1H, q, *J* = 2.0 Hz), 4.58 (1H, bdd, *J* = 5.7, 2.0 Hz), 4.13 (1H, q, *J* = 7.4 Hz), 3.84–3.76 (2H, m), 3.77 (1H, dd, *J* = 9.9, 6.3 Hz), 3.68 (1H, td, *J* = 6.3, 1.1 Hz), 3.65 (1H, dd, *J* = 7.4, 1.1 Hz), 3.43 (1H, dd, *J* = 9.9, 6.3 Hz), 2.74 (1H, dd, *J* = 6.6, 4.1 Hz, OH), 2.63 (1H, ddt, *J* = 15.6, 7.4, 2.0 Hz,), 2.37 (1H, ddt, *J* = 15.6, 7.4, 2.0 Hz), 1.83–1.72 (2H, m), 0.88 (9H, s), 0.88 (18H, s), 0.07 (3H, s), 0.07 (3H, s), 0.07 (3H, s), 0.05 (3H, s), 0.03 (6H, s); ^13^C{^1^H} NMR (126 MHz, CDCl_3_) δ 151.4, 104.9, 79.9, 79.0, 78.8, 75.8, 64.2, 61.6, 37.0, 35.9, 26.2, 26.2, 26.1, 18.5, 18.5, 18.4, −4.2, −4.4, −4.4, −4.4, −5.3, −5.3; LRMS (CI, isobutane) *m*/*z* (intensity) 561.4 [M + H]^+^ (100); HRMS (CI, isobutane) *m*/*z*: [M + H]^+^ calcd for C_28_H_61_O_5_Si_3_ 561.3827; found 561.3831.

### 2-{(2*S*,3*R*,5*R*)-5-[(5*S*,6*R*)-6-(Hydroxymethyl)-2,2,3,3,8,8,9,9-octamethyl-4,7-dioxa-3,8-disiladecan-5-yl]-3-methyltetrahydrofuran-2-yl}ethyl 2,2-dimethylpropanoate (**13**)

A solution of [Ir(cod)(IMes)(PPh_3_)]PF_6_ (19.7 mg, 19.4 μmol) and alcohol **11** (1.09 g, 1.94 mmol) in dichloromethane (40 mL) was cooled to −78 °C. The flask was purged three times with hydrogen, and the cooling bath was removed. The solution was stirred under an atmosphere of hydrogen at rt for 1 h, and then the atmosphere was replaced with argon. Pyridine (629 μL, 7.81 mmol) and trimethylacetyl chloride (718 μL, 5.87 mmol) were added sequentially, and the mixture was stirred at rt for 22 h. The reaction was quenched by the addition of 1 M aqueous hydrochloric acid (40 mL), and the mixture was extracted with diethyl ether (3 × 60 mL). The combined organic extracts were washed with 1 M aqueous sodium hydroxide (40 mL) and saturated aqueous copper(II) sulfate (50 mL), dried (anhydrous MgSO_4_), filtered, and concentrated under reduced pressure to afford the crude pivaloyl ester **12**, which was used in the next step without purification. To a solution of crude ester **12** in THF (200 mL) at 0 °C was added a stock solution of HF·pyridine (11.5 mL). The mixture was stirred at 0 °C for 24 h, and the reaction was then quenched by the addition of saturated aqueous sodium bicarbonate (250 mL). The mixture was extracted with diethyl ether (3 × 100 mL), and the combined organic extracts were washed with brine (80 mL), dried (anhydrous MgSO_4_), filtered, and concentrated under reduced pressure. The residue was purified by flash chromatography on silica gel (pet. ether-ethyl acetate, 19:1 to 10:1) to give alcohol **13** (787 mg, 76% over 2 steps) as a colorless oil. *R_f_* = 0.31 (pet. ether-ethyl acetate, 9:1); [α]_D_^23^ −17 (*c* = 0.5, CHCl_3_); ν_max_. 3445, 2958, 2930, 2884, 2857, 1730, 835, 777 cm^–1^; ^1^H NMR (500 MHz, CDCl_3_) δ 4.18 (1H, ddd, *J* = 11.1, 6.9, 5.6 Hz), 4.14–4.05 (2H, m), 4.02 (1H, dt, *J* = 8.3, 5.0 Hz), 3.80–3.73 (2H, m), 3.63 (1H, dd, *J* = 3.6, 1.8 Hz), 3.54–3.47 (1H, m), 3.28–3.24 (1H, m, OH), 2.31–2.24 (1H, m), 2.08 (1H, ddd, *J* = 12.3, 8.8, 7.0 Hz), 1.82–1.70 (2H, m), 1.64 (1H, ddd, *J* = 12.3, 7.0, 2.0 Hz), 1.19 (9H, s), 0.91 (3H, d, *J* = 7.0 Hz), 0.90 (9H, s), 0.90 (9H, s), 0.12 (3H, s), 0.09 (3H, s), 0.08 (3H, s), 0.08 (3H, s); ^13^C{^1^H} NMR (126 MHz, CDCl_3_) δ 178.6, 79.1, 79.0, 78.2, 75.7, 62.9, 62.3, 38.8, 36.5, 36.3, 30.1, 27.3, 26.2, 26.1, 18.6, 18.4, 14.3, −4.0, −4.5, −4.5, −4.8; HRMS (ESI+) *m*/*z*: [M + Na]^+^ calcd for C_27_H_56_NaO_6_Si_2_ 555.3508; found 555.3484.

### 2-{(2*S*,3*R*,5*R*)-5-[(5*S*,6*R*)-6-Ethynyl-2,2,3,3,8,8,9,9-octamethyl-4,7-dioxa-3,8-disiladecan-5-yl]-3-methyl-tetrahydrofuran-2-yl}ethyl 2,2-dimethylpropanoate (**15**)

Pyridine (1.09 mL, 13.5 mmol) and Dess–Martin periodinane (1.90 g, 4.48 mmol) were added sequentially to a solution of alcohol **13** (597 mg, 1.12 mmol) in dichloromethane (18 mL) at rt. The solution was stirred at rt for 3 h, and the reaction was quenched by the addition of saturated aqueous sodium sulfite (40 mL) and saturated aqueous sodium bicarbonate (40 mL). The mixture was extracted with diethyl ether (3 × 40 mL), and the combined organic extracts were washed with saturated aqueous sodium bicarbonate (40 mL) and saturated aqueous copper(II) sulfate (80 mL), then dried (anhydrous MgSO_4_), filtered, and concentrated under reduced pressure to give the crude aldehyde **14**, which was used directly in the next step without purification. Anhydrous potassium carbonate (464 mg, 3.36 mmol) was added to a solution of the Ohira–Bestmann reagent (861 mg, 4.48 mmol) in methanol (18 mL) at 0 °C. The mixture was stirred at this temperature for 1 h, and then a solution of the crude aldehyde in THF (9 mL) was added dropwise. Stirring was continued at 0 °C for 1 h, and the mixture was then warmed to rt and stirred for an additional 15 min. The reaction was quenched with saturated aqueous ammonium chloride (40 mL), and the aqueous phase was extracted with diethyl ether (3 × 40 mL). The combined organic extracts were washed with brine (50 mL), dried (anhydrous MgSO_4_), filtered, and concentrated under reduced pressure. The resulting residue was purified by flash chromatography on silica gel (pet. ether-ethyl acetate, 50:1) to yield alkyne **15** (443 mg, 75% over two steps) as a colorless oil. *R_f_* = 0.50 (pet. ether-ethyl acetate, 20:1); [α]_D_^22^ −30 (*c* = 1.0, CHCl_3_); ν_max_. 2958, 2929, 2886, 2858, 1730, 832, 776 cm^–1^; ^1^H NMR (500 MHz, CDCl_3_) δ 4.38 (1H, dd, *J* = 4.2, 2.2 Hz), 4.20 (1H, ddd, *J* = 11.2, 7.0, 5.7 Hz), 4.15 (1H, ddd, *J* = 8.2, 7.1, 5.6 Hz), 4.11 (1H, ddd, *J* = 11.2, 7.7, 6.8 Hz), 3.94 (1H, dt, *J* = 8.4, 5.0 Hz), 3.57 (1H, dd, *J* = 5.6, 4.2 Hz), 2.36 (1H, d, *J* = 2.2 Hz), 2.29–2.21 (1H, m), 2.11 (1H, ddd, *J* = 12.3, 8.2, 7.2 Hz), 1.78–1.67 (2H, m), 1.63 (1H, ddd, *J* = 12.3, 7.1, 2.6 Hz), 1.19 (9H, s), 0.91 (3H, d, *J* = 7.0 Hz), 0.90 (9H, s), 0.90 (9H, s), 0.13 (3H, s), 0.12 (3H, s), 0.11 (3H, s), 0.09 (3H, s); ^13^C NMR (126 MHz, CDCl_3_) δ 178.7, 84.4, 78.9, 78.0, 77.3, 74.0, 65.7, 62.7, 38.8, 36.4, 36.0, 30.5, 27.4, 26.3, 25.9, 18.6, 18.4, 14.3, −3.9, −4.0, −4.5, −5.0; HRMS (ESI+) *m*/*z*: [M + Na]^+^ calcd for C_28_H_54_NaO_5_Si_2_ 549.3402; found 549.3390.

### 2-{(2*S*,3*R*,5*R*)-3-Methyl-5-{(5*S*,6*S*)-2,2,3,3,8,8,9,9-octamethyl-6-[1-(tributylstannyl)ethen-1-yl]-4,7-dioxa-3,8-disiladecan-5-yl}tetrahydrofuran-2-yl}ethyl 2,2-dimethyl-propanoate (**16**)

To a solution of alkyne **15** (216 mg, 0.410 mmol), 2,6-*tert*-butyl-4-methylphenol (9 mg), and Mo(CO)_3_(CNt-Bu)_3_ (17.6 mg, 0.044 mmol) in THF (1.8 mL) at rt was added tri-*n*-butyltin hydride (551 μL, 2.04 mmol). The mixture was heated (oil bath) to 55 °C and stirred at this temperature for 24 h. Additional Mo(CO)_3_(CNt-Bu)_3_ (17.6, mg, 0.044 mmol) and tri-*n*-butyltin hydride (331 μL, 1.23 mmol) were added, and the solution was stirred at 55 °C for an additional 24 h. The reaction was then concentrated under reduced pressure, and the residue was purified directly by flash chromatography on silica gel (pet. ether-ethyl acetate, 200:1) to afford the 1,1-disubstituted vinyl stannane **16** (231.5 mg, 69%) along with the regioisomeric alkenyl stannane (53.7 mg, 16%). *R_f_* = 0.87 (pet. ether-ethyl acetate, 20:1); [α]_D_^22^ −20 (*c* = 1.1, CHCl_3_); ν_max_. 2956, 2928, 2856, 1733, 832, 776 cm^–1^; ^1^H NMR (500 MHz, CDCl_3_) δ 5.94 (1H, dd, *J* = 2.7, 1.9 Hz, ^3^*J*_SnH_ = 132.4 Hz), 5.23 (1H, dd, *J* = 2.7, 1.9 Hz, ^3^*J*_SnH_ = 63.8 Hz), 4.34 (1H, q, *J* = 1.9 Hz, ^3^*J*_SnH_ = 28.3 Hz), 4.19 (1H, dt, *J* = 11.8, 6.1 Hz), 4.12–4.05 (2H, m), 3.84 (1H, dt, *J* = 7.9, 5.2 Hz), 3.53 (1H, dd, *J* = 7.6, 1.9 Hz), 2.17–2.09 (1H, m), 1.76–1.68 (3H, m), 1.56–1.40 (7H, m), 1.36–1.27 (6H, m), 1.19 (9H, s), 0.96–0.85 (18H, m), 0.91 (9H, s), 0.89 (9H, s), 0.11 (3H, s), 0.09 (3H, s), 0.06 (3H, s), −0.01 (3H, s); ^13^C{^1^H} NMR (126 MHz, CDCl_3_) δ 178.6, 154.0, 125.5, 82.7, 79.3, 78.8, 76.9, 62.6, 38.8, 37.8, 36.4, 30.3, 29.3, 27.6, 27.4, 26.5, 26.3, 18.7, 18.6, 14.1, 13.8, 10.2, −3.7, −4.1, −4.2, −4.3; HRMS (ESI+) *m*/*z*: [M + Na]^+^ calcd for C_40_H_82_NaO_5_Si_2_Sn^120^ 841.4615; found 841.4600.

### (2*R*,5*S*,6*S*)-7-(*tert*-Butyldimethylsilyloxy)-5-hydroxy-1-[(4-methoxybenzyl)oxy]-2,6-dimethylheptan-3-one (**19**)

A solution of methyl ketone **17** (1.40 g, 6.30 mmol) in diethyl ether (4 mL) was added to a solution of dicyclohexylboron chloride (3.35 g, 15.8 mmol) in diethyl ether (24 mL) at 0 °C. Triethylamine was added dropwise, and the resulting suspension was stirred at 0 °C for 1 h. The mixture was cooled to −78 °C, then a solution of aldehyde **18** (1.53 g, 7.56 mmol) in diethyl ether (4 mL) was added, and the resulting mixture was stirred at −78 °C for 2 h. The mixture was then warmed to 0 °C, and the reaction was quenched by the sequential addition of methanol (12 mL), aqueous pH 7 buffer (24 mL), and 30% aqueous hydrogen peroxide (24 mL). The resulting mixture was stirred vigorously at rt for 1 h. The phases were separated, and the aqueous phase was extracted with diethyl ether (2 × 25 mL). The combined organic extracts were washed with brine (40 mL), dried (anhydrous MgSO_4_), filtered, and concentrated under reduced pressure. The residue was purified by flash chromatography on silica gel (pet. ether-ethyl acetate, 19:1 to 9:1) to afford hydroxyketone **19** (2.17 g, 81%, d.r. > 20:1) as a colorless oil. *R_f_* = 0.35 (pet. ether-ethyl acetate, 4:1); [α]_D_^23^ −15 (*c* = 1.5, CHCl_3_); ν_max_. 3510, 2955, 2930, 2857, 1708, 1513, 834, 775 cm^–1^; ^1^H NMR (500 MHz, CDCl_3_) δ 7.20 (2H, d, *J* = 8.6 Hz), 6.86 (2H, d, *J* = 8.6 Hz), 4.42 (1H, d, *J* = 11.7 Hz), 4.38 (1H, d, *J* = 11.7 Hz), 4.30–4.23 (1H, m), 3.79 (3H, s), 3.67 (1H, dd, *J* = 9.9, 4.6 Hz), 3.61 (1H, dd, *J* = 9.9, 5.9 Hz), 3.58 (1H, dd, *J* = 9.0, 8.0 Hz), 3.45 (1H, dd, *J* = 9.0, 5.3 Hz), 3.29 (1H, d, *J* = 2.7 Hz, OH), 2.93–2.85 (1H, m), 2.70 (1H, dd, *J* = 17.0, 9.1 Hz), 2.58 (1H, dd, *J* = 17.0, 3.4 Hz), 1.73–1.64 (1H, m), 1.06 (3H, d, *J* = 7.1 Hz), 0.89 (3H, d, *J* = 6.9 Hz), 0.89 (9H, s), 0.05 (6H, s); ^13^C{^1^H} NMR (126 MHz, CDCl_3_) δ 213.7, 159.4, 130.1, 129.4, 113.9, 73.1, 72.0, 69.3, 66.9, 55.4, 47.0, 46.9, 39.5, 26.0, 18.3, 13.3, 10.9, −5.4, −5.4; HRMS (ESI+) *m*/*z*: [M + Na]^+^ calcd for C_23_H_40_NaO_5_Si 447.2537; found 447.2548.

### (2*S*,3*S*,5*S*,6*R*)-1-(*tert*-Butyldimethylsilyloxy)-5-hydroxy-7-[(4-methoxybenzyl)oxy]-2,6-dimethylheptan-3-yl 2,2-dimethylpropanoate (**20**)

Samarium powder (451 mg, 3.00 mmol) was added to a flame-dried round-bottomed flask. The flask was evacuated and refilled with argon three times before THF (20 mL) was added. Iodine (508 mg, 2.00 mmol) was added, and the resulting brown slurry was heated (oil bath) at 50 °C for 18 h to give a dark blue solution of samarium(II) iodide (approximately 0.1 M). The solution was allowed to cool and settle to rt over 1 h and then used directly in the Evans–Tischenko reaction. To a solution of freshly distilled pivaldehyde (3.81 mL, 35.1 mmol) in THF (8.2 mL) at −30 °C was added samarium(II) iodide (11.7 mL of a 0.1 M solution in THF, 1.17 mmol). The resulting mixture was stirred at −30 °C for 10 min. A solution of hydroxyketone **19** (2.40 g, 5.65 mmol) in THF (8.2 mL) was added, and the reaction mixture was stirred for 3 h maintaining the temperature between −10 and −20 °C. The reaction was quenched by the addition of saturated aqueous sodium bicarbonate (20 mL), and the mixture was extracted with diethyl ether (3 × 20 mL). The combined organic extracts were washed with brine (30 mL), dried (anhydrous MgSO_4_), filtered, and concentrated under reduced pressure. The residue was purified by flash chromatography on silica gel (pet. ether-ethyl acetate, 20:1) to yield alcohol **20** (2.18 g, 76%, d.r. > 10:1) as a colorless oil. *R_f_* = 0.21 (pet. ether-ethyl acetate, 19:1); [α]_D_^23^ −10 (*c* = 0.5, CHCl_3_); ν_max_. 3517, 2957, 2929, 2858, 1707, 1513, 836, 775 cm^–1^; ^1^H NMR (400 MHz, CDCl_3_) δ 7.25–7.21 (2H, m), 6.88–6.84 (2H, m), 5.22 (1H, ddd, *J* = 10.7, 3.3, 2.7 Hz), 4.43 (1H, d, *J* = 11.8 Hz), 4.40 (1H, d, *J* = 11.8 Hz), 3.80 (3H, s), 3.51–3.38 (5H, m), 3.38–3.30 (1H, m), 1.89–1.80 (2H, m), 1.74 (1H, ddd, *J* = 14.0, 10.7, 2.3 Hz), 1.52 (1H, ddd, *J* = 14.0, 10.7, 2.7 Hz), 1.20 (9H, s), 0.93 (3H, d, *J* = 6.9 Hz), 0.92 (3H, d, *J* = 6.9 Hz), 0.88 (9H, s), 0.02 (3H, s), 0.01 (3H, s); ^13^C{^1^H} NMR (101 MHz, CDCl_3_) δ 179.4, 159.3, 130.7, 129.3, 113.9, 73.2, 73.0, 71.2, 69.9, 65.1, 55.4, 40.3, 39.2, 38.9, 37.5, 27.4, 26.0, 18.3, 14.0, 11.5, −5.3; HRMS (ESI+) *m*/*z*: [M + Na]^+^ calcd for C_28_H_50_NaO_6_Si 533.3269; found 533.3271.

### (5*S*,7*S*,8*S*)-5-[(*R*)-1-Hydroxypropan-2-yl]-2,2,3,3,8,11,11,12,12-nonamethyl-4,10-dioxa-3,11-disilatridecan-7-yl 2,2-dimethylpropanoate (**21**)

To a solution of alcohol **20** (2.18 g, 4.27 mmol) in dichloromethane (21 mL) at −78 °C were sequentially added 2,6-lutidine (1.49 mL, 12.9 mmol) and *tert*-butyldimethylsilyl trifluoromethanesulfonate (1.47 mL, 6.40 mmol). The mixture was stirred at −78 °C for 1.5 h, and the reaction was then quenched by the addition of saturated aqueous sodium bicarbonate (20 mL). The resulting mixture was extracted with diethyl ether (3 × 30 mL), and the combined organic extracts were washed with saturated aqueous copper(II) sulfate (30 mL) and brine (30 mL), dried (anhydrous MgSO_4_), filtered, and concentrated under reduced pressure. The residue was filtered through a short pad of silica gel (pet. ether-ethyl acetate, 20:1) to deliver the crude silyl ether, which was used directly in the next step without purification. A mixture of silyl ether and Pearlman’s catalyst (20 wt %, 599 mg, 0.85 mmol) in ethanol at rt was purged three times with hydrogen, and the reaction was stirred under an atmosphere of hydrogen at rt for 2 h. The mixture was filtered to remove the catalyst and concentrated under reduced pressure. The residue was purified by flash chromatography on silica gel (pet. ether-ethyl acetate, 20:1) to afford primary alcohol **21** (1.85 g, 86% over two steps) as a colorless oil. *R_f_* = 0.42 (pet. ether-ethyl acetate, 9:1); [α]_D_^22^ −10 (*c* = 0.9, CHCl_3_); ν_max_. 3510, 2956, 2929, 2858, 1728, 835, 774 cm^–1^; ^1^H NMR (400 MHz, CDCl_3_) δ 4.99 (1H, ddd, *J* = 7.8, 5.5, 3.0 Hz), 3.84 (1H, dt, *J* = 11.1, 3.5 Hz), 3.75 (1H, td, *J* = 6.3, 2.7 Hz), 3.55–3.49 (1H, m), 3.46 (1H, dd, *J* = 9.4, 6.9 Hz), 3.39 (1H, dd, *J* = 9.4, 6.5 Hz), 2.37 (1H, dd, *J* = 7.4, 3.5 Hz), 1.89–1.79 (2H, m), 1.79–1.68 (2H, m), 1.18 (9H, s), 1.01 (3H, d, *J* = 7.1 Hz), 0.93 (3H, d, *J* = 7.0 Hz), 0.89 (9H, s), 0.87 (9H, s), 0.09 (3H, s), 0.07 (3H, s), 0.01 (3H, s), 0.01 (3H, s); ^13^C NMR (101 MHz, CDCl_3_) δ 178.0, 73.7, 71.9, 64.8, 64.7, 39.6, 39.4, 39.1, 36.7, 27.4, 26.0, 18.4, 18.1, 13.9, 11.2, −4.3, −4.5, −5.3, −5.4; HRMS (ESI+) *m*/*z*: [M + Na]^+^ calcd for C_26_H_56_NaO_5_Si_2_ 527.3558; found 527.3539.

### (5*S*,7*S*,8*S*)-5-[(*R*)-But-3-yn-2-yl]-2,2,3,3,8,11,11,12,12-nonamethyl-4,10-dioxa-3,11-disilatridecan-7-yl 2,2-dimethylpropanoate (**23**)

2,2,6,6-Tetramethyl-1-piperidinyloxy (50 mg, 0.32 mmol) and (diacetoxyiodo)benzene (1.13 g, 3.51 mmol) were added sequentially to a solution of alcohol **21** (1.61 g, 3.19 mmol) in dichloromethane (16 mL) at rt. The mixture was stirred at rt for 5 h, and the reaction was quenched by the addition of water (20 mL). The resulting mixture was extracted with diethyl ether (3 × 25 mL), and the combined organic extracts were washed with brine (20 mL), dried (anhydrous MgSO_4_), filtered, and concentrated under reduced pressure. The residue was filtered rapidly through a short pad of silica gel (pet. ether-ethyl acetate, 30:1) to give the crude aldehyde **22**, which was used directly in the subsequent alkyne-forming reaction without purification. Anhydrous potassium (1.32 g, 9.55 mmol) was added to a solution of the Ohira–Bestmann reagent (2.45 g, 12.8 mmol) in methanol (15 mL) at 0 °C. The mixture was stirred for 1 h at this temperature, and then a solution of the crude aldehyde **22** in THF (7.5 mL) was added dropwise. Stirring was continued at 0 °C for 1 h, and the mixture was then warmed to rt and stirred for an additional 15 min. The reaction was quenched by the addition of saturated aqueous ammonium chloride (40 mL), and the resulting mixture was extracted with diethyl ether (3 × 40 mL). The combined organic extracts were washed with brine (50 mL), dried (anhydrous MgSO_4_), filtered, and concentrated under reduced pressure. The resulting residue was purified by flash chromatography on silica gel (pet. ether-ethyl acetate, 100:1) to yield alkyne **23** (1.16 g, 73% over two steps) as a colorless oil. *R_f_* = 0.72 (pet. ether-ethyl acetate, 20:1); [α]_D_^22^ −11 (*c* = 1.1, CHCl_3_); ν_max_. 3315, 2957, 2930, 2886, 2859, 1727, 836, 775 cm^–1^; ^1^H NMR (400 MHz, CDCl_3_) δ 5.02 (1H, ddd, *J* = 9.9, 3.7, 2.2 Hz), 3.71 (1H, ddd, *J* = 9.2, 3.7, 2.0 Hz), 3.55 (1H, dd, *J* = 9.9, 5.5 Hz), 3.40 (1H, dd, *J* = 9.9, 6.9 Hz), 2.63–2.56 (1H, m), 2.05 (1H, d, *J* = 2.5 Hz), 2.03–1.93 (1H, m), 1.92–1.82 (1H, m), 1.59 (1H, ddd, *J* = 14.3, 9.2, 2.2 Hz), 1.20 (9H, s), 1.13 (3H, d, *J* = 7.1 Hz), 0.93 (3H, d, *J* = 7.0 Hz), 0.88 (18H, s), 0.09 (3H, s), 0.03 (3H, s), 0.02 (3H, s), 0.02 (3H, s); ^13^C{^1^H} NMR (101 MHz, CDCl_3_) δ 177.9, 86.2, 72.4, 71.3, 70.1, 64.9, 40.4, 39.1, 35.3, 32.6, 27.5, 26.1, 26.0, 18.4, 18.2, 13.6, 12.0, −4.5, −4.5, −5.3, −5.3; HRMS (ESI+) *m*/*z*: [M + Na]^+^ calcd for C_27_H_54_NaO_4_Si_2_ 521.3453; found 521.3427.

### (5*S*,7*S*,8*S*)-5-[(*R*,*E*)-4-(Dimethylphenylsilyl)-3-methyl-but-3-en-2-yl]-2,2,3,3,8,11,11,12,12-nonamethyl-4,10-dioxa-3,11-disilatridecan-7-yl 2,2-dimethylpropanoate (**24**)

Lithium metal (312 mg, 45.0 mmol) was added to a flame-dried round-bottomed flask. The flask was evacuated and refilled with argon before THF (20 mL) was added. The mixture was cooled to 0 °C, and freshly distilled chloro(dimethyl)-phenylsilane (1.66 mL, 9.89 mmol) was added. The resulting mixture was stirred at 0 °C for 6 h, giving a dark red solution of dimethylphenylsilyllithium (approximate concentration 0.5 M), and the solution was used immediately for the reaction. Copper(I) cyanide (270 mg, 3.01 mmol) was added to a flame-dried round-bottomed flask and dried at 55 °C (oil bath) under high vacuum overnight. The flask was cooled to 0 °C and refilled with argon before THF (6.5 mL) was added. A solution of dimethylphenylsilyllithium (12.0 mL of a 0.5 M solution in THF, 6.01 mmol) was then added in one portion. The resulting blood red solution was stirred at 0 °C for 30 min during which time the color changed from red to purple. A solution of alkyne **23** (1.00 g, 2.00 mmol) in THF (17 mL) was added, and the reaction was stirred at 0 °C for 1 h. Iodomethane (1.25 mL, 20.1 mmol) was added, and stirring was continued at 0 °C for an additional 1 h. The reaction was quenched by the addition of ammonium hydroxide (30% v/v in water, 40 mL) and diethyl ether (25 mL) under vigorous stirring. The biphasic mixture was partitioned between water (80 mL) and diethyl ether (40 mL), and the aqueous layer was extracted with diethyl ether (3 × 50 mL). The combined organic extracts were washed with water (3 × 40 mL) and brine (65 mL), dried (anhydrous MgSO_4_), filtered, and concentrated under reduced pressure. The residue was purified by flash chromatography on silica gel (pet. ether-ethyl acetate, 200:1) to give vinylic silane **24** (1.17 g, 90%) as a colorless oil. *R_f_* = 0.74 (pet. ether-ethyl acetate, 20:1); [α]_D_^25^ −4.8 (*c* = 1.0, CHCl_3_); ν_max_. 2956, 2929, 2885, 2857, 2360, 2337, 1727, 834, 774 cm^–1^; ^1^H NMR (400 MHz, CDCl_3_) δ 7.55–7.52 (2H, m), 7.35–7.32 (3H, m), 5.45 (1H, bs), 5.03 (1H, dt, *J* = 9.7, 2.8 Hz), 3.80 (1H, ddd, *J* = 9.4, 3.5, 2.0 Hz), 3.57 (1H, dd, *J* = 9.9, 5.3 Hz), 3.36 (1H, dd, *J* = 9.9, 7.5 Hz), 2.39 (1H, bqd, *J* = 7.0, 3.5 Hz), 1.90–1.84 (1H, m), 1.73 (3H, s), 1.58 (1H, ddd, *J* = 14.4, 9.7, 2.0 Hz), 1.49 (1H, ddd, *J* = 14.4, 9.4, 2.8 Hz), 1.15 (9H, s), 1.04 (3H, d, *J* = 7.0 Hz), 0.93 (3H, d, *J* = 7.0 Hz), 0.91 (9H, s), 0.89 (9H, s), 0.37 (3H, s), 0.36 (3H, s), 0.09 (3H, s), 0.06 (3H, s), 0.03 (3H, s), 0.03 (3H, s); ^13^C{^1^H} NMR (101 MHz, CDCl_3_) δ 177.7, 157.9, 140.2, 133.9, 128.8, 127.9, 121.6, 72.5, 70.4, 65.0, 50.0, 40.5, 39.0, 33.8, 27.5, 26.1, 22.8, 18.4, 18.2, 12.0, 11.7, −0.7, −0.8, −4.2, −4.5, −5.3; HRMS (ESI+) *m*/*z*: [M + Na]^+^ calcd for C_36_H_68_NaO_4_Si_3_ 671.4318; found 671.4292.

### (2*S*,3*S*,5*S*,6*R*,*E*)-5-(*tert*-Butyldimethylsilyloxy)-1-hydroxy-8-iodo-2,6,7-trimethyloct-7-en-3-yl 2,2-dimethyl-propanoate (**25**)

A solution of freshly recrystallized *N*-iodosuccinimide (700 mg, 3.11 mmol) in acetonitrile (3.6 mL) was added dropwise to a solution of alkenylsilane **24** (400 mg, 0.62 mmol) in a mixture of acetonitrile and benzene (2.5:1; 5 mL) at 0 °C. The bright red mixture was stirred at 0 °C for 4 h, and the reaction was then quenched with saturated aqueous sodium sulfite (8 mL) under vigorous stirring. The resulting colorless mixture was extracted with diethyl ether (3 × 15 mL). The combined organic extracts were washed with water (20 mL) and brine (20 mL), dried (anhydrous MgSO_4_), filtered, and concentrated under reduced pressure. The resulting residue was filtered rapidly through a short pad (ca. 5 cm) of silica gel (from pure pentane to pentane-diethyl ether, 200:1) to afford crude vinylic iodide, which was immediately used in the next step without purification. Pyridine (4 mL) and HF·pyridine (70% HF, 4 mL) were added sequentially to a solution of crude iodide in THF (60 mL) at 0 °C. The resulting solution was stirred at 0 °C until TLC indicated complete consumption of the starting material (24–36 h). The reaction was quenched by the addition of saturated aqueous sodium bicarbonate until gas evolution ceased, and the mixture was extracted with diethyl ether (3 × 50 mL). The combined organic extracts were washed with brine (50 mL), dried (anhydrous MgSO_4_), filtered, and concentrated under reduced pressure. The resulting residue was purified by flash chromatography on silica gel (pet. ether-ethyl acetate, 20:1 to 10:1) to give alcohol **25** (206 mg, 63% over two steps) as a colorless oil. *R_f_* = 0.11 (pet. ether-ethyl acetate, 19:1); [α]_D_^21^ +14 (*c* = 1.0, CHCl_3_); ν_max_. 3491, 2957, 2930, 2883, 2858, 1725, 1708, 836, 806, 775 cm^–1^; ^1^H NMR (400 MHz, CDCl_3_) δ 6.03–6.01 (1H, m), 5.21 (1H, dt, *J* = 9.8, 2.3), 3.73 (1H, ddd, *J* = 9.3, 3.9, 2.3 Hz), 3.40 (1H, ddd, *J* = 13.0, 11.1, 5.4 Hz), 3.13–3.06 (2H, m), 2.54–2.47 (1H, m), 1.85 (3H, d, *J* = 0.8 Hz), 1.84–1.77 (1H, m), 1.65 (1H, ddd, *J* = 14.4, 9.8, 2.3 Hz), 1.40 (1H, ddd, *J* = 14.4, 9.3, 2.3 Hz), 1.21 (9H, s), 1.05 (3H, d, *J* = 7.0 Hz), 0.89 (9H, s), 0.77 (3H, d, *J* = 7.0 Hz), 0.07 (3H, s), 0.05 (3H, s); ^13^C{^1^H} NMR (101 MHz, CDCl_3_) δ 179.9, 148.8, 77.6, 70.6, 70.4, 64.5, 48.7, 40.7, 39.3, 35.6, 27.5, 26.0, 24.6, 18.2, 12.4, 9.8, −4.0,–4.6; LRMS (CI, isobutane) *m*/*z* (intensity) 527.0 [M + H]^+^ (15), 395.0 (15), 113.1 (38), 73.1 (100); HRMS (CI, isobutane) *m*/*z*: [M + H]^+^ calcd for C_22_H_44_O_4_SiI 527.2054; found 527.2052.

### (*R*)-1-(*tert*-Butyldimethylsilyloxy)hept-6-yn-3-ol (**27**)

To a suspension of magnesium turnings (2.19 g, 90.1 mmol) in diethyl ether (45 mL) at rt were added mercury(II) chloride (122 mg, 0.449 mmol, 1 mol %) and iodine (two crystals). The mixture was cooled to 0 °C, and propargyl bromide (80 wt % in toluene, 5.0 mL, 45 mmol) was added dropwise. The mixture was cooled until reflux stabilized. The reaction mixture was then heated (oil bath) at reflux for 1 h, and the resulting yellow solution was then allowed to cool to rt. To a solution of epoxide **26** (1.94 g, 9.59 mmol) in diethyl ether (200 mL) at −78 °C was added a freshly prepared solution of propargylmagnesium bromide (29 mL of a 1.0 M solution in diethyl ether, 29 mmol) dropwise. The resulting mixture was stirred at −78 °C for 30 min and at rt for 1.5 h. The reaction mixture was cooled to 0 °C, and saturated aqueous ammonium chloride (200 mL) was added. The phases were separated, and the aqueous phase was extracted with diethyl ether (4 × 160 mL). The combined organic extracts were washed with brine (400 mL), dried (anhydrous MgSO_4_), filtered, and concentrated. The residue was purified by flash chromatography on silica gel (pet. ether-diethyl ether, 80:20) to deliver alcohol **27** (1.57 g, 68%) as a yellow oil. *R_f_* = 0.37 (pet. ether-diethyl ether, 80:20); [α]_D_^24^ +26.9 (*c* = 1.11, CHCl_3_); ν_max_. 3444, 3315, 2951, 2929, 2884, 2858, 662 cm^–1^; ^1^H NMR (400 MHz, CDCl_3_) δ 4.00–3.93 (1H, m), 3.91 (1H, app dt, *J* = 9.9, 4.7 Hz), 3.87–3.80 (1H, m), 3.51 (1H, d, *J* = 2.3 Hz), 2.34 (2H, ddd, *J* = 7.7, 6.9, 2.7 Hz), 1.95 (1H, t, *J* = 2.7 Hz), 1.76–1.60 (4H, m), 0.90 (9H, s), 0.08 (3H, s), 0.08 (3H, s); ^13^C{^1^H} NMR (101 MHz, CDCl_3_) δ 84.6, 71.0, 68.5, 62.9, 38.3, 36.2, 26.0, 18.3, 14.9, −5.4, −5.4; HRMS (ESI+) *m*/*z*: [M + Na]^+^ calcd for C_13_H_26_NaO_2_Si 265.1594; found 265.1588.

### (*R*)-1,3-Bis(*tert*-butyldimethylsilyloxy)hept-6-yne (**28**)

To a solution of alcohol **27** (2.79 g, 11.5 mmol) in dichloromethane (58 mL) at 0 °C were added imidazole (2.35 g, 34.5 mmol), 4-dimethylaminopyridine (442 mg, 3.62 mmol), and *tert*-butyldimethylsilyl chloride (3.47 g, 13 mmol). The reaction mixture was stirred at rt for 40 h before the addition of saturated aqueous ammonium chloride (58 mL). The phases were separated, and the aqueous phase was extracted with dichloromethane (3 × 50 mL). The combined organic extracts were washed with brine (120 mL), dried (anhydrous MgSO_4_), filtered, and concentrated. The residue was purified by flash chromatography on silica gel (pet. ether-diethyl ether, 99:1 → 98:2 → 95:5) to afford alkyne **28** (3.85 g, 94%) as a colorless oil. *R_f_* = 0.34 (pet. ether-diethyl ether, 98:2); [α]_D_^26^ +6.1 (*c* = 1.0, CHCl_3_); ν_max_. 3316, 2929, 2885, 2858, 661 cm^–1^; ^1^H NMR (500 MHz, CDCl_3_) δ 3.97–3.91 (1H, m), 3.66 (2H, t, *J* = 6.6 Hz), 2.24 (2H, td, *J* = 7.4, 2.7 Hz), 1.92 (1H, t, *J* = 2.7 Hz), 1.75–1.59 (4H, m), 0.89 (9H, s), 0.88 (9H, s), 0.07 (3H, s), 0.06 (3H, s), 0.04 (6H, s); ^13^C{^1^H} NMR (126 MHz, CDCl_3_) δ 84.7, 68.4, 68.2, 59.8, 40.1, 36.1, 26.1, 26.0, 18.4, 18.2, 14.6, −4.4, −4.4, −5.2; HRMS (ESI+) *m*/*z*: [M + Na]^+^ calcd for C_19_H_40_NaO_2_Si_2_ 379.2459; found 379.2441.

### (*R*)-6,8-Bis(*tert*-butyldimethylsilyloxy)oct-2-yn-1-ol (**29**)

To a solution of alkyne **28** (3.85 g, 10.8 mmol) in THF (54 mL) at −78 °C was added *n*-BuLi (5.7 mL of a 2.1 M solution in hexanes, 12 mmol) dropwise. The resulting solution was stirred at −78 °C for 1 h before the addition of paraformaldehyde (1.12 g, 37.3 mmol) in one portion. The reaction mixture was allowed to warm to rt for 10 min and stirred at 40 °C for 45 min. The solution was allowed to cool to rt, and 1 M aqueous sodium hydroxide (50 mL) was added. The biphasic mixture was stirred vigorously at rt for 45 min, and the phases were separated. The organic layer was washed with saturated aqueous ammonium chloride (50 mL), and the phases were separated. The aqueous phase was extracted with diethyl ether (3 × 50 mL), and the combined organic extracts were washed with brine (150 mL), dried (anhydrous MgSO_4_), filtered, and concentrated. The residue was purified by flash chromatography on silica gel (pet. ether-diethyl ether, 80:20) to provide propargylic alcohol **29** (3.69 g, 88%) as a yellow oil. *R_f_* = 0.33 (pet. ether-diethyl ether, 80:20); [α]_D_^22^ +5.5 (*c* = 1.1, CHCl_3_); ν_max_. 3353, 2954, 2929, 2884, 2857 cm^–1^; ^1^H NMR (500 MHz, CDCl_3_) δ 4.24 (2H, dt, *J* = 6.1, 2.1 Hz), 3.94–3.88 (1H, m), 3.66 (2H, t, *J* = 6.6 Hz), 2.27 (2H, tt, *J* = 7.3, 2.1 Hz), 1.74–1.59 (4H, m), 1.49 (1H, t, *J* = 6.1 Hz), 0.89 (9H, s), 0.88 (9H, s), 0.06 (3H, s), 0.06 (3H, s), 0.04 (6H, s); ^13^C{^1^H} NMR (126 MHz, CDCl_3_) δ 86.7, 78.6, 68.3, 59.8, 51.6, 40.1, 36.1, 26.1, 26.0, 18.4, 18.2, 14.9, −4.4, −4.4, −5.2; HRMS (ESI+) *m*/*z*: [M + Na]^+^ calcd for C_20_H_42_NaO_3_Si_2_ 409.2565; found 409.2566.

### (*R*,*Z*)-6,8-Bis(*tert*-butyldimethylsilyloxy)oct-2-en-1-ol (**30**)

To a solution of propargylic alcohol **29** (4.50 g, 11.6 mmol) in pet. ether (58 mL) at rt was added quinoline (1.80 mL, 15.2 mmol) slowly, followed by palladium on calcium carbonate (5 wt %, poisoned with lead, 495 mg, 0.233 mmol, 2 mol %). The reaction mixture was purged with hydrogen three times and then stirred at rt under a hydrogen atmosphere for 1.5 h. The mixture was filtered through a celite pad, and the solids were washed with diethyl ether (5 × 50 mL). The filtrates were concentrated, and the residue was purified by flash chromatography on silica gel (pet. ether-diethyl ether, 80:20) to provide allylic alcohol **30** (4.30 g, 95%) as a light yellow oil. *R_f_* = 0.28 (pet. ether-diethyl ether, 80:20); [α]_D_^20^ −5.4 (*c* = 1.0, CHCl_3_); ν_max_. 3327, 2954, 2929, 2885, 2858, 832, 772 cm^–1^; ^1^H NMR (400 MHz, CDCl_3_) δ 5.64–5.57 (1H, m), 5.57–5.49 (1H, m), 4.25–4.14 (2H, m), 3.84 (1H, app p, *J* = 5.7 Hz), 3.66 (2H, t, *J* = 6.5 Hz), 2.22–2.03 (2H, m), 1.69–1.62 (2H, m), 1.58–1.47 (2H, m), 1.31 (1H, t, *J* = 5.7 Hz), 0.89 (18H, s), 0.05 (3H, s), 0.05 (3H, s), 0.04 (6H, s); ^13^C{^1^H} NMR (126 MHz, CDCl_3_) δ 133.0, 128.6, 69.0, 60.0, 58.7, 40.0, 37.3, 26.1, 26.0, 23.3, 18.4, 18.2, −4.3, −4.4, −5.2, −5.2; HRMS (ESI+) *m*/*z*: [M + Na]^+^ calcd for C_20_H_44_NaO_3_Si_2_ 411.2721, found 411.2714.

### (*R*)-1,3-Bis(*tert*-butyldimethylsilyloxy)-5-[(2*S*,3*R*)-3-(hydroxymethyl)oxiran-2-yl]pentane (**31**)

To a suspension of 4 Å MS (1.0 g) in dichloromethane (21 mL) at −20 °C were added d-(−)-diethyltartrate (0.263 mL, 1.54 mmol), titanium(IV) isopropoxide (0.379 mL, 1.28 mmol), and *t*-butyl hydroperoxide (8.1 mL of a 1.9 M solution in dichloromethane, 15 mmol) sequentially. The resulting mixture was stirred at −20 °C for 30 min before the dropwise addition of allylic alcohol **30** (1.99 g, 5.13 mmol) in dichloromethane (6 mL) for 10 min. The reaction mixture was stirred at −20 °C for 24 h. The reaction was quenched by the addition of water (7.3 mL) at 0 °C, and the mixture was stirred for 45 min while warming to rt. Aqueous sodium hydroxide (30%) saturated with sodium chloride (1.6 mL) was added, and the mixture was stirred vigorously for 45 min. The phases were separated, and the milky, aqueous phase was extracted with dichloromethane (3 × 30 mL). The combined organic extracts were washed with brine (90 mL), dried (anhydrous MgSO_4_), filtered, and concentrated. The residue was purified by flash chromatography on silica gel (pet. ether-ethyl acetate, 80:20) to afford epoxide **31** (1.88 g, 91%) as a colorless oil. *R_f_* = 0.33 (pet. ether-ethyl acetate, 80:20); [α]_D_^20^ −5.1 (*c* = 1.1, CHCl_3_); ν_max_. 3407, 2954, 2929, 2885, 2857, 1254, 832, 773 cm^–1^; ^1^H NMR (400 MHz, CDCl_3_) δ 3.92–3.85 (1H, m), 3.82 (1H, ddd, *J* = 12.0, 7.2, 4.6 Hz), 3.70 (1H, ddd, *J* = 12.0, 6.6, 5.3 Hz), 3.66 (2H, t, *J* = 6.4 Hz), 3.16 (1H, ddd, *J* = 6.6, 4.6, 4.4 Hz), 3.02 (1H, app td, *J* = 6.3, 4.4 Hz), 1.81 (1H, dd, *J* = 7.2, 5.3 Hz), 1.78–1.47 (6H, m), 0.89 (9H, s), 0.88 (9H, s), 0.06 (3H, s), 0.05 (3H, s), 0.04 (6H, s); ^13^C{^1^H} NMR (126 MHz, CDCl_3_) δ 68.9, 60.9, 59.9, 57.4, 56.9, 40.3, 34.0, 26.1, 26.0, 23.8, 18.4, 18.2, −4.3, −4.4, −5.2, −5.2; LRMS (CI+, isobutane) *m*/*z* (intensity) 405.3 (100%), 273.2 (34%); HRMS (CI+, isobutane) *m*/*z*: [M + H]^+^ calcd for C_20_H_45_O_4_Si_2_ 405.2856; found 405.2851.

### (3*R*)-1,3-Bis(*tert*-butyldimethylsilyloxy)-5-[(2*S*,3*R*)-3-ethynyloxiran-2-yl]pentane (**33**)

To a solution of oxalyl chloride (1.60 mL, 18.7 mmol) in dichloromethane (25 mL) at −78 °C was added a solution of dimethyl sulfoxide (2.89 mL, 40.7 mmol) in dichloromethane (4 mL) dropwise. The resulting solution was stirred at −78 °C for 20 min before the slow addition of alcohol **31** (3.43 g, 8.47 mmol) in dichloromethane (13 mL). The reaction mixture was stirred at −78 °C for 1.5 h, and triethylamine (5.91 mL, 42.4 mmol) was added. The mixture was stirred at rt for 1 h, and the reaction was quenched by the addition of saturated aqueous ammonium chloride (40 mL). The phases were separated, and the aqueous phase was extracted with dichloromethane (3 × 40 mL). The combined organic extracts were washed with brine (120 mL), dried (anhydrous MgSO_4_), filtered, and concentrated. The crude aldehyde **32** was used directly in the next step without purification. To a solution of dimethyl(1-diazo-2-oxopropyl)phosphonate (1.79 g, 9.32 mmol) in methanol (38 mL) at 0 °C was added potassium carbonate (1.64 g, 11.9 mmol) in one portion. The mixture was stirred at 0 °C for 1.5 h before the dropwise addition of the crude aldehyde **32** in THF (19 mL). The resulting yellow suspension was stirred at 0 °C for 2 h and at rt for 45 min. The reaction was quenched by the addition of saturated aqueous ammonium chloride (50 mL). The mixture was filtered through a plug of cotton wool, and the phases were separated. The aqueous phase was extracted with diethyl ether (3 × 50 mL), and the combined organic extracts were washed with brine (150 mL), dried (anhydrous MgSO_4_), filtered, and concentrated. The residue was purified by flash chromatography on silica gel (pet. ether-diethyl ether, 95:5) to provide alkyne **33** (1.85 g, 55% over two steps) as a colorless oil. *R_f_* = 0.28 (pet. ether-diethyl ether, 95:5); [α]_D_^24^ −15.6 (*c* = 1.00, CHCl_3_); ν_max_. 3314, 2954, 2929, 2885, 2857, 1253, 773, 662 cm^–1^; ^1^H NMR (400 MHz, CDCl_3_) δ 3.92–3.85 (1H, m), 3.70–3.64 (2H, m), 3.42 (1H, dd, *J* = 4.0, 1.7 Hz), 3.07–3.02 (1H, m), 2.33 (1H, d, *J* = 1.7 Hz), 1.84–1.56 (6H, m), 0.89 (9H, s), 0.88 (9H, s), 0.06 (6H, s), 0.04 (6H, s); ^13^C NMR (126 MHz, CDCl_3_) δ 79.0, 73.6, 69.0, 60.0, 58.0, 45.0, 40.2, 33.3, 26.1, 26.0, 25.4, 18.4, 18.2, −4.3, −4.4, −5.2; LRMS (CI+, isobutane) *m*/*z* (intensity) 399.3 (100%), 267.2 (34%); HRMS (CI+, isobutane) *m*/*z*: [M + H]^+^ calcd for C_21_H_43_O_3_Si_2_ 399.2751; found 399.2756.

### 2-{(2*R*,5*R*)-5-[(*R*)-1-Hydroxyprop-2-yn-1-yl]-tetrahydrofuran-2-yl}ethyl 2,2-dimethylpropanoate (**35**)

To a solution of epoxide **33** (920 mg, 2.31 mmol) in THF (15 mL) at 0 °C was added tetra-*n*-butylammonium fluoride (6.9 mL of a 1.0 M in solution in THF, 6.9 mmol) dropwise. The resulting solution was stirred at rt for 2.5 h. The reaction was quenched by the addition of saturated aqueous ammonium chloride (15 mL). The phases were separated, and the aqueous phase was extracted with ethyl acetate (3 × 15 mL). The combined organic extracts were washed with brine (45 mL), dried (anhydrous MgSO_4_), filtered, and concentrated. The residue was filtered rapidly through a short pad of silica gel (ethyl acetate) to give the crude diol, which was used in the subsequent cyclization reaction without further purification. *R_f_* = 0.41 (EtOAc); ^1^H NMR (500 MHz, CDCl_3_) δ 4.02–3.95 (1H, m), 3.94–3.82 (2H, m), 3.46 (1H, dd, *J* = 4.0, 1.7 Hz), 3.12–3.07 (1H, m), 2.37 (1H, d, *J* = 1.7 Hz), 1.95–1.86 (1H, m), 1.85–1.78 (1H, m), 1.78–1.67 (4H, m); ^13^C{^1^H} NMR (126 MHz, CDCl_3_) δ 78.8, 74.0, 71.6, 62.0, 57.9, 45.0, 38.5, 34.0, 25.6; HRMS (ESI+) *m*/*z*: [M + Na]^+^ calcd for C_9_H_14_NaO_3_ 193.0835; found 193.0830.

To a solution of crude diol (391 mg) in dichloromethane (23 mL) at −40 °C was added (1*S*)-(+)-camphorsulfonic acid (53 mg, 0.23 mmol). The resulting mixture was stirred at −40 °C for 10 min and at rt for 30 min. The reaction was quenched by the addition of triethylamine (96 μL, 0.72 mmol), and the solution was concentrated. The residue was filtered rapidly through a short pad of silica gel (ethyl acetate) to give the crude tetrahydrofuran **34**, which was used directly in the next step without purification. *R_f_* = 0.38 (EtOAc); ^1^H NMR (400 MHz, CDCl_3_) δ 4.25–4.17 (2H, m), 4.16–4.09 (1H, m), 3.79 (2H, app t, *J* = 5.7 Hz), 2.69 (1H, br s), 2.56 (1H, br s), 2.44 (1H, d, *J* = 2.2 Hz), 2.16–2.06 (2H, m), 1.91–1.82 (1H, m), 1.81–1.76 (2H, m), 1.70–1.59 (1H, m); ^13^C{^1^H} NMR (126 MHz, CDCl_3_) δ 82.1, 81.8, 79.7, 73.9, 65.1, 61.3, 37.5, 32.3, 27.9.

To a solution of crude tetrahydrofuran **34** in dichloromethane (12 mL) at 0 °C were added pyridine (0.28 mL, 3.5 mmol) and, after 5 min, trimethylacetyl chloride (305 mg, 2.53 mmol). The resulting solution was stirred at 0 °C for 10 min and at rt for 22 h. The reaction was quenched by the addition of saturated aqueous sodium bicarbonate (9 mL). The phases were separated, and the aqueous phase was extracted with ethyl acetate (3 × 10 mL). The combined organic extracts were washed with brine (30 mL), dried (anhydrous MgSO_4_), filtered, and concentrated. The residue was purified by flash chromatography on silica gel (pet. ether-ethyl acetate, 70:30) to provide pivalate ester **35** (442 mg, 75% over three steps) as a colorless oil. *R_f_* = 0.49 (pet. ether-ethyl acetate, 70:30); [α]_D_^24^ −12.6 (*c* = 1.04, CHCl_3_); ν_max_. 3447, 3284, 2972, 2936, 2910, 2875, 1724, 772, 654 cm^–1^; ^1^H NMR (500 MHz, CDCl_3_) δ 4.23–4.12 (3H, m), 4.09 (1H, app q, *J* = 7.0 Hz), 4.07–4.00 (1H, m), 2.53 (1H, d, *J* = 4.3 Hz), 2.43 (1H, d, *J* = 2.2 Hz), 2.16–2.00 (2H, m), 1.95–1.87 (1H, m), 1.86–1.77 (2H, m), 1.65–1.56 (1H, m), 1.19 (9H, s); ^13^C NMR (126 MHz, CDCl_3_) δ 178.6, 81.9, 81.5, 77.0, 73.7, 65.1, 61.7, 38.7, 34.5, 32.0, 28.0, 27.2; LRMS (CI+, isobutane) *m*/*z* (intensity) 255.19 (100%), 229.18 (17%); HRMS (CI+, isobutane) *m*/*z*: [M + H]^+^ calcd for C_14_H_23_O_4_ 255.1596; found 255.1593.

### 2-{(2*R*,5*R*)-5-[(*R*)-1-Hydroxy-5-methylhex-4-en-2-yn-1-yl]tetrahydrofuran-2-yl}ethyl 2,2-dimethylpropanoate

To a suspension of tetrakis(triphenylphosphine)palladium(0) (25 mg, 22 μmol, 4 mol %) in pyrrolidine (700 μL) at rt was added 1-bromo-2-methyl-1-propene (174 mg, 1.29 mmol) followed, after 5 min, by the dropwise addition of alkyne **35** (109 mg, 0.429 mmol) in pyrrolidine (700 μL). The resulting yellow solution was stirred at 50 °C for 16 h. The reaction mixture was allowed to cool to rt, and then saturated aqueous ammonium chloride (3 mL) was added. The phases were separated, and the aqueous phase was extracted with diethyl ether (3 × 5 mL). The combined organic extracts were dried (anhydrous MgSO_4_), filtered, and concentrated. The residue was purified by flash chromatography on silica gel (pet. ether-ethyl acetate, 80:20) to give the title alcohol (116 mg, 88%) as a light yellow oil. *R_f_* = 0.26 (pet. ether-ethyl acetate, 80:20); [α]_D_^27^ +12.6 (*c* = 2.32, CHCl_3_); ν_max_. 3443, 2969, 2934, 2911, 2874, 2212, 1726, 772 cm^–1^; ^1^H NMR (400 MHz, CDCl_3_) δ 5.28–5.25 (1H, m), 4.37–4.32 (1H, m), 4.22–4.12 (2H, m), 4.12–4.00 (2H, m), 2.49 (1H, d, *J* = 4.0 Hz), 2.18–1.99 (2H, m), 1.96–1.87 (1H, m), 1.88 (3H, br s), 1.87–1.78 (2H, m), 1.80 (3H, br s), 1.65–1.54 (1H, m), 1.19 (9H, s); ^13^C{^1^H} NMR (126 MHz, CDCl_3_) δ 178.7, 149.7, 104.7, 89.0, 83.9, 82.1, 76.9, 66.2, 61.9, 38.9, 34.7, 32.2, 28.3, 27.3, 24.9, 21.2; HRMS (ESI+) *m*/*z*: [M + Na]^+^ calcd for C_18_H_28_NaO_4_ 331.1880; found 331.1870.

### 2-{(2*R*,5*R*)-5-[(*R*)-1-(*tert*-Butyldimethylsilyloxy)-5-methylhex-4-en-2-yn-1-yl]tetrahydrofuran-2-yl}ethyl 2,2-dimethylpropanoate (**36**)

To a solution of alcohol (174 mg g, 0.564 mmol) in dichloromethane (6 mL) at −78 °C were added 2,6-lutidine (170 μL, 1.46 mmol) and *tert*-butyldimethylsilyl trifluoromethanesulfonate (168 μL, 0.732 mmol) sequentially. The resulting solution was stirred at −78 °C for 30 min, and water (2 mL) was added. The biphasic mixture was allowed to warm to rt, and the phases were separated. The aqueous phase was extracted with dichloromethane (3 × 3 mL), and the combined organic extracts were washed with brine (10 mL), dried (anhydrous MgSO_4_), filtered, and concentrated. The residue was purified by flash chromatography on silica gel (pet. ether-ethyl acetate, 95:5) to give enyne **36** (225 mg, 94%) as a colorless oil. *R_f_* = 0.26 (pet. ether-ethyl acetate, 95:5); [α]_D_^25^ −13.1 (*c* = 2.40, CHCl_3_); ν_max_. 2957, 2930, 2907, 2886, 2857, 2211, 1730, 835, 775, 669 cm^–1^; ^1^H NMR (500 MHz, CDCl_3_) δ 5.27–5.23 (1H, m), 4.51 (1H, dd, *J* = 5.8, 1.4 Hz,), 4.20–4.04 (4H, m), 2.11–2.03 (2H, m), 2.01–1.93 (1H, m), 1.93–1.84 (1H, m), 1.87 (3H, br s), 1.83–1.74 (1H, m), 1.79 (3H, br s), 1.56–1.48 (1H, m), 1.18 (9H, s), 0.90 (9H, s), 0.12 (3H, s), 0.11 (3H, s); ^13^C{^1^H} NMR (126 MHz, CDCl_3_) δ 178.6, 148.7, 105.1, 90.6, 83.3, 81.8, 76.8, 67.0, 62.1, 38.8, 34.8, 32.3, 28.0, 27.3, 26.0, 24.9, 21.1, 18.5, −4.5, −4.8; HRMS (ESI+) *m*/*z*: [M + Na]^+^ calcd for C_24_H_42_NaO_4_Si 445.2745; found 445.2734.

### 2-{(2*R*,5*R*)-5-[(*R*)-1-(*tert*-Butyldimethylsilyloxy)-5-methylhex-4-en-2-yn-1-yl]tetrahydrofuran-2-yl}ethanol (**37**)

To a solution of ester **36** (478 mg, 1.13 mmol) in diethyl ether (16 mL) at −20 °C was added lithium aluminum hydride (107 mg, 2.82 mmol) in one portion. The resulting solution was stirred at −20 °C for 20 min before the dropwise addition of water (0.11 mL), 15% aqueous sodium hydroxide (0.11 mL), and water (0.33 mL). The mixture was stirred vigorously at rt for 20 min and then filtered through a cotton plug. The filtrate was concentrated, and the residue was purified by flash chromatography on silica gel (pet. ether-ethyl acetate, 80:20) to give alcohol **37** (369 mg, 96%) as a colorless oil. *R_f_* = 0.30 (pet. ether-ethyl acetate, 80:20); [α]_D_^26^ −17.5 (*c* = 2.00, CHCl_3_); ν_max_. 3410, 2954, 2929, 2883, 2857, 2206, 835, 776, 668 cm^–1^; ^1^H NMR (500 MHz, CDCl_3_) δ 5.27–5.25 (1H, m), 4.48 (1H, dd, *J* = 6.1, 1.7 Hz), 4.23–4.16 (1H, m), 4.10 (1H, ddd, *J* = 7.6, 6.5, 6.1 Hz), 3.82–3.73 (2H, m), 2.90 (1H, dd, *J* = 7.0, 4.2 Hz), 2.11–2.03 (2H, m), 1.98–1.88 (1H, m), 1.87 (3H, br s), 1.79 (3H, br s), 1.78–1.73 (2H, m), 1.62–1.54 (1H, m), 0.90 (9H, s), 0.13 (3H, s), 0.11 (3H, s); ^13^C{^1^H} NMR (126 MHz, CDCl_3_) δ 148.9, 105.0, 90.4, 83.4, 82.3, 80.4, 66.9, 62.0, 37.3, 32.5, 27.8, 25.9, 24.9, 21.1, 18.4, −4.5, −4.8; LRMS (CI+, isobutane) *m*/*z* (intensity) 339.1 (19%), 263.1 (27%), 207.1 (100%), 135.0 (23%); HRMS (CI+, isobutane) *m*/*z*: [M + H]+ calcd for C_19_H_35_O_3_Si 339.2355; found 339.2351.

### 2-{(2*R*,5*R*)-5-[(*R*)-1-(*tert*-Butyldimethylsilyloxy)-5-methylhex-4-en-2-yn-1-yl]tetrahydrofuran-2-yl}-acetaldehyde (**38**)

To a solution of alcohol **37** (269 mg, 0.795 mmol) in dichloromethane (11 mL) at rt were added pyridine (0.39 mL, 4.8 mmol) and Dess–Martin periodinane (674 mg, 1.59 mmol) sequentially. The resulting solution was stirred at rt for 1 h before the addition of saturated aqueous sodium sulfite (10 mL). The layers were separated, and the organic phase was washed with saturated aqueous sodium bicarbonate (10 mL). The aqueous phase was extracted with dichloromethane (3 × 10 mL), and the combined organic extracts were washed with brine (40 mL), dried (anhydrous MgSO_4_), filtered, and concentrated. The residue was purified by flash chromatography on silica gel (pet. ether-ethyl acetate, 90:10) to deliver aldehyde **38** (239 mg, 89%) as a colorless oil. *R_f_* = 0.38 (pet. ether-ethyl acetate, 90:10); ^1^H NMR (400 MHz, CDCl_3_) δ 9.80 (1H, dd, *J* = 2.5, 2.1 Hz), 5.28–5.25 (1H, m), 4.54 (1H, dd, *J* = 5.8, 1.5 Hz), 4.47 (1H, app ddt, *J* = 8.6, 7.2, 5.5 Hz), 4.10 (1H, app td, *J* = 7.1, 5.8 Hz), 2.68 (1H, ddd, *J* = 16.2, 7.2, 2.5 Hz), 2.56 (1H, ddd, *J* = 16.2, 5.5, 2.1 Hz), 2.24–2.16 (1H, m), 2.14–1.98 (2H, m), 1.88 (3H, br s), 1.80 (3H, br s), 1.56 (1H, app ddt, *J* = 12.1, 9.4, 8.6 Hz), 0.90 (9H, s), 0.12 (3H, s), 0.11 (3H, s).

### 2-({(2*R*, 5*R*)-5-[(1*R*, 2*E*)-1-(Triethylsilyloxy)-5-methyl-2,4-hexadien-1-yl] tetrahydro-2-furanyl} methyl)-1,3-dithiane (**40**)

To a suspension of magnesium bromide ethyl etherate (238 mg, 0.922 mmol) in diethyl ether (5 mL) at rt was added 1,3-propanedithiol (86 μL, 0.85 mmol) followed by a solution of aldehyde **38** (239 mg, 0.710 mmol) in diethyl ether (2 mL). The resulting mixture was stirred at rt for 1.5 h, and water (7 mL) was added. The phases were separated, and the aqueous phase was extracted with diethyl ether (3 × 7 mL). The combined organic extracts were washed with brine (20 mL), dried (anhydrous MgSO_4_), filtered, and concentrated. The residue was used directly in the next step without further purification.

To a solution of the crude dithiane **39** (303 mg) in THF (7 mL) at 0 °C was added tetra-*n*-butylammonium fluoride (1.4 mL of a 1.0 M solution in THF, 1.4 mmol) dropwise. The resulting solution was stirred at rt for 1 h, and then water (7 mL) was added. The phases were separated, and the aqueous phase was extracted with diethyl ether (3 × 7 mL). The combined organic extracts were washed with brine (20 mL), dried (anhydrous MgSO_4_), filtered, and concentrated. The residue was used directly in the next step without purification.

To a solution of the crude propargylic alcohol (155 mg) in THF (10 mL) at 0 °C was added sodium bis(2-methoxyethoxy)aluminum hydride (670 μL of a ≥65 wt % in toluene, 2.2 mmol) dropwise. The resulting cloudy mixture was stirred at rt for 30 min and then cooled to 0 °C, and saturated aqueous potassium sodium tartrate (20 mL) was added. The phases were separated, and the aqueous phase was extracted with diethyl ether (3 × 20 mL). The combined organic extracts were washed with brine (50 mL), dried (anhydrous MgSO_4_), filtered, and concentrated. The residue was used directly in the next step without purification.

To a solution of crude allylic alcohol (156 mg) in dichloromethane (10 mL) at −78 °C were added 2,6-lutidine (0.17 mL, 1.5 mmol) and triethylsilyl trifluoromethanesulfonate (0.17 mL, 0.74 mmol) sequentially. The resulting solution was stirred at −78 °C for 30 min, and then water (8 mL) was added. The biphasic mixture was allowed to warm to rt, and the phases were separated. The aqueous phase was extracted with dichloromethane (3 × 8 mL), and the combined organic extracts were washed with brine (35 mL), dried (anhydrous MgSO_4_), filtered, and concentrated. The residue was purified by flash chromatography on silica gel (pet. ether-ethyl acetate, 98:2) to deliver diene **40** (141 mg, 0.33 mmol, 47% over four steps) as a colorless oil. *R_f_* = 0.36 (pet. ether-ethyl acetate, 95:5); [α]_D_^29^ +27.1 (*c* = 1.10, CHCl_3_); ν_max_. 2953, 2930, 2911, 2874, 2859, 1728, 740, 727 cm^–1^; ^1^H NMR (400 MHz, CDCl_3_) δ 6.46 (1H, ddd, *J* = 15.1, 11.0, 1.1 Hz), 5.82 (1H, br d, *J* = 11.0 Hz), 5.52 (1H, dd, *J* = 15.1, 5.8 Hz), 4.21 (1H, dd, *J* = 9.6, 4.9 Hz), 4.24–4.15 (2H, m), 3.93 (1H, ddd, *J* = 8.0, 7.1, 6.3 Hz), 2.94–2.77 (4H, m), 2.16–2.06 (1H, m), 2.03–1.78 (5H, m), 1.77 (3H, br s), 1.75 (3H, br s), 1.68 (1H, app ddt, *J* = 12.6, 9.0, 8.0 Hz), 1.45 (1H, app dtd, *J* = 11.8, 9.0, 8.1 Hz), 0.96 (9H, t, *J* = 7.9 Hz), 0.62 (6H, q, *J* = 7.9 Hz); ^13^C{^1^H} NMR (126 MHz, CDCl_3_) δ 135.2, 129.9, 127.7, 124.9, 82.4, 75.6, 75.5, 44.7, 41.8, 32.3, 30.7, 30.2, 27.4, 26.2, 26.1, 18.4, 7.1, 5.1; HRMS (ESI+) *m*/*z*: [M + Na]^+^ calcd for C_22_H_40_NaO_2_S_2_Si 451.2131; found 451.2110.

### Triethyl((*R*,*E*)-5-methyl-1-{(2*R*,5*R*)-5-[2-(triethylsilyloxy)ethyl]tetrahydrofuran-2-yl}hexa-2,4-dien-1-yl)-oxysilane (**43**)

To a suspension of tetrakis(triphenylphosphine)palladium(0) (120 mg, 0.104 mmol, 5 mol %) in pyrrolidine (2.3 mL) at rt was added 1-bromo-2-methyl-1-propene (842 mg, 6.24 mmol) followed, after 5 min, by the dropwise addition of alkyne **34** (354 mg, 2.08 mmol) in pyrrolidine (2.3 mL). The resulting yellow solution was stirred at 50 °C (oil bath) for 16 h. The reaction mixture was allowed to cool to rt, followed by the addition of saturated aqueous ammonium chloride (10 mL). The phases were separated, and the aqueous phase was extracted with diethyl ether (3 × 10 mL). The combined organic extracts were dried (anhydrous MgSO_4_), filtered, and concentrated. The crude enyne **41** was used directly in the next step without further purification.

To a solution of crude enyne **41** (409 mg) in THF (40 mL) at 0 °C was added sodium bis(2-methoxyethoxy)aluminum hydride (2.46 mL of a ≥65 wt % solution in toluene, ∼7.9 mmol) dropwise. The resulting cloudy mixture was stirred at rt for 30 min and cooled to 0 °C before the dropwise addition of saturated aqueous potassium sodium tartrate solution (40 mL). The phases were separated, and the aqueous phase was extracted with diethyl ether (3 × 40 mL). The combined organic extracts were washed with brine (120 mL), dried (anhydrous MgSO_4_), filtered, and concentrated. The crude diene **42** was used directly in the next step without purification.

To a solution of crude diene **42** (364 mg) in dichloromethane (15 mL) at −78 °C were added 2,6-lutidine (1.12 mL, 9.67 mmol) and triethylsilyl trifluoromethanesulfonate (1.09 mL, 6.02 mmol) sequentially. The resulting solution was stirred at −78 °C for 45 min. Water (15 mL) was added, and the biphasic mixture was allowed to warm to rt. The phases were separated, and the aqueous phase was extracted with dichloromethane (3 × 15 mL). The combined organic extracts were dried (anhydrous MgSO_4_), filtered, and concentrated. The residue was purified by flash chromatography on silica gel (pet. ether-ethyl acetate, 98:2) to deliver diene **43** (590 mg, 62% over three steps) as a colorless oil. *R_f_* = 0.28 (pet. ether-ethyl acetate, 98:2); [α]_D_^24^ +13.5 (*c* = 2.13, CHCl_3_) {Lit.^4^ [α]_D_^20^ +13 (*c* = 1.0, CHCl_3_)}; ν_max_. 2955, 2938, 2911, 2876, 743, 727 cm^–1^; ^1^H NMR (400 MHz, CDCl_3_) δ 6.45 (1H, ddd, *J* = 15.2, 11.0, 1.4 Hz), 5.82 (1H, br d, *J* = 11.0 Hz), 5.54 (1H, dd, *J* = 15.2, 5.9 Hz), 4.18 (1H, app td, *J* = 5.9, 1.4 Hz), 3.97 (1H, app ddt, *J* = 8.2, 7.7, 5.5 Hz), 3.92 (1H, app td, *J* = 7.3, 5.9 Hz), 3.78–3.62 (2H, m), 1.96 (1H, dddd, *J* = 11.7, 8.3, 5.5, 3.2 Hz), 1.91–1.79 (2H, m), 1.77 (3H, br s), 1.75 (3H, br s), 1.73–1.61 (2H, m), 1.46 (1H, app ddt, *J* = 11.7, 9.8, 8.2 Hz), 0.96 (9H, t, *J* = 7.9 Hz), 0.95 (9H, t, *J* = 7.9 Hz), 0.64–0.56 (12H, m); ^13^C{^1^H} NMR (101 MHz, CDCl_3_) δ 135.0, 130.1, 127.6, 124.9, 82.2, 76.8, 75.6, 60.6, 39.2, 32.5, 27.5, 26.1, 18.4, 7.0, 6.9, 5.2, 4.6; HRMS (ESI+) *m*/*z*: [M + Na]^+^ calcd for C_25_H_50_NaO_3_Si_2_ 477.3191; found 477.3198.

### 2-{(2*R*,5*R*)-5-[(*R*,*E*)-5-Methyl-1-(triethylsilyloxy)hexa-2,4-dien-1-yl]tetrahydrofuran-2-yl}ethanol (**44**)

A stock solution of HF·pyridine was prepared by mixing HF·pyridine (1.0 mL of a 70% HF in pyridine solution), pyridine (2.0 mL), and THF (5.0 mL). To a solution of diene **43** (219 mg, 0.48 mmol) in THF (48 mL) at −20 °C was added the stock solution of HF·pyridine (1.75 mL), and the resulting mixture was stirred for 15 h. The reaction was quenched by the dropwise addition of saturated aqueous sodium bicarbonate (150 mL). The biphasic mixture was allowed to warm to rt, and the phases were separated. The aqueous phase was extracted with diethyl ether (3 × 150 mL), and the combined organic extracts were washed with brine (400 mL), dried (anhydrous MgSO_4_), filtered, and concentrated. The residue was purified by flash chromatography on silica gel (pet. ether-ethyl acetate, 85:15) to afford alcohol **44** (130 mg, 79%) as a colorless oil. *R_f_* = 0.21 (pet. ether-ethyl acetate, 85:15); [α]_D_^24^ +11.4 (*c* = 2.08, CHCl_3_); ν_max_. 3410, 2955, 2936, 2911, 2876, 1659, 742 cm^–1^; ^1^H NMR (500 MHz, CDCl_3_) δ 6.44 (1H, ddd, *J* = 15.2, 11.1, 1.3 Hz), 5.81 (1H, dm, *J* = 11.1 Hz), 5.51 (1H, dd, *J* = 15.2, 6.2 Hz), 4.19–4.06 (2H, m), 3.96 (1H, app dt, *J* = 8.0, 6.3 Hz), 3.84–3.71 (2H, m), 3.00 (1H, dd, *J* = 7.0, 4.2 Hz), 2.00 (1H, dddd, *J* = 11.9, 8.3, 5.7, 3.0 Hz), 1.92–1.84 (1H, m), 1.77 (3H, br s), 1.75 (3H, br s), 1.79–1.62 (3H, m), 1.54 (1H, app ddt, *J* = 11.9, 9.9, 8.2 Hz), 0.95 (9H, t, *J* = 8.0 Hz), 0.60 (6H, q, *J* = 8.0 Hz); ^13^C{^1^H} NMR (101 MHz, CDCl_3_) δ 135.5, 129.9, 127.9, 124.7, 82.7, 80.0, 75.7, 62.0, 37.4, 32.6, 27.4, 26.2, 18.4, 7.0, 5.2; LRMS (EI+) m/z (intensity) 340.2 (2%), 225.1 (100%), 115.1 (18%), 87.1 (12%); HRMS (EI+) *m*/*z*: [M]^+^ calcd for C_19_H_36_O_3_Si 340.2434; found 340.2431.

### {(2*R*,5*R*)-5-[(*R*,*E*)-5-Methyl-1-(triethylsilyloxy)hexa-2,4-dien-1-yl]tetrahydrofuran-2-yl}acetaldehyde (**45**)

To a solution of alcohol **44** (129 mg, 0.38 mmol) in dichloromethane (4 mL) at rt were added pyridine (0.14 mL, 1.7 mmol) and Dess–Martin periodinane (241 mg, 0.568 mmol) sequentially. The resulting solution was stirred at rt for 1 h before the addition of saturated aqueous sodium sulfite solution (4 mL). The phases were separated, and the organic layer was washed with saturated sodium bicarbonate (4 mL). The aqueous phase was extracted with dichloromethane (3 × 4 mL), and the combined organic extracts were washed with brine (20 mL), dried (anhydrous MgSO_4_), filtered, and concentrated. The residue was purified by flash chromatography on silica gel (pet. ether-ethyl acetate, 90:10) to deliver aldehyde **45** (108 mg, 84%) as a colorless oil. *R_f_* = 0.37 (pet. ether-ethyl acetate, 90:10); ^1^H NMR (400 MHz, CDCl_3_) δ 9.80 (1H, app t, *J* = 2.2 Hz), 6.45 (1H, ddd, *J* = 15.2, 11.0, 1.3 Hz), 5.82 (1H, dm, *J* = 11.0 Hz), 5.53 (1H, dd, *J* = 15.2, 6.0 Hz), 4.35 (1H, app ddt, *J =* 8.4, 7.3, 5.5 Hz), 4.17 (1H, ddd, *J* = 6.0, 5.8, 1.3 Hz), 3.98 (1H, app td, *J* = 7.3, 5.8 Hz), 2.66 (1H, ddd, *J* = 16.2, 7.3, 2.2 Hz), 2.54 (1H, ddd, *J* = 16.2, 5.5, 2.2 Hz), 2.13–2.05 (1H, m), 1.96–1.86 (1H, m), 1.77 (3H, br s), 1.83–1.73 (1H, m), 1.75 (3H, br s), 1.51 (1H, app ddt, *J* = 12.0, 9.6, 8.4 Hz), 0.94 (9H, t, *J* = 7.9 Hz), 0.59 (6H, q, *J* = 7.9 Hz).

### 2-{(2*S*,3*R*,5*R*)-5-[(1*S*,2*R*,6*R*,7*S*,9*S*,10*S*,*E*)-9-(2,2-Dimethyl-1-oxopropoxy)-11-hydroxy-3-methylene-5,6,10-trimethyl-1,2,7-tris(*tert*-butyldimethylsilyloxy)undec-4-en-1-yl]-3-methyltetrahydrofuran-2-yl}ethyl 2,2-dimethyl-propanoate

Tetrabutylammonium diphenylphosphinate (243 mg, 0.529 mmol) was dried by azeotropic distillation (oil bath) with benzene (1 mL) and then under high vacuum. The flask was then filled with argon, and DMF (450 μL) was added. A solution of stannane **16** (108 mg, 0.132 mmol) in a mixture of DMF and THF (4:1, 1 mL) was added followed by a solution of iodide **25** (69.5 mg, 0.132 mmol) in a mixture of DMF and THF (4:1, 1 mL). Tetrakis(triphenylphosphine)-palladium(0) (46 mg, 39.8 μmol) and copper(I) thiophene-2-carboxylate (75.5 mg, 0.396 mmol) were introduced quickly in solid form, and the resulting mixture was stirred at rt for 2 h. The reaction mixture was diluted with water (10 mL) and extracted with diethyl ether (3 × 10 mL). The combined organic extracts were washed with brine (15 mL), dried (anhydrous MgSO_4_), filtered, and concentrated under reduced pressure. The resulting residue was purified by flash chromatography on silica gel (pet. ether-ethyl acetate, 20:1 to 10:1) to deliver the title 1,3-diene (100 mg, 82%) as a colorless oil. *R_f_* = 0.32 (pet. ether-ethyl acetate, 9:1); [α]_D_^24^ +4.5 (*c* = 1.0, CHCl_3_); ν_max_. 2956, 2929, 2857, 1728, 834, 775 cm^–1^; ^1^H NMR (400 MHz, CDCl_3_) δ 5.73 (1H, br s), 5.29 (1H, dd, *J* = 1.9, 1.2 Hz), 5.24 (1H, dt, *J* = 10.0, 1.7 Hz), 4.91 (1H, br s), 4.19 (1H, ddd, *J* = 11.1, 6.7, 5.6 Hz), 4.13–4.04 (3H, m), 3.86 (1H, dt, *J* = 8.1, 5.1 Hz), 3.79 (1H, ddd, *J* = 9.6, 3.6, 1.7 Hz), 3.51 (1H, dd, *J* = 7.0, 3.2 Hz), 3.39 (1H, ddd, *J* = 11.6, 9.7, 5.5 Hz), 3.18 (1H, dd, *J* = 9.7, 3.6 Hz), 3.08 (1H, td, *J* = 11.6, 3.6 Hz), 2.43–2.33 (1H, m), 2.22–2.12 (1H, m), 1.87–1.77 (2H, m), 1.79 (3H, d, *J* = 0.6 Hz), 1.75–1.68 (3H, m), 1.61 (1H, ddd, *J* = 12.4, 6.7, 2.1 Hz), 1.41–1.33 (1H, m), 1.20 (9H, s), 1.18 (9H, s), 1.04 (3H, d, *J* = 7.0 Hz), 0.90 (9H, s), 0.90 (9H, s), 0.89 (3H, d, *J* = 7.1 Hz), 0.87 (9H, s), 0.75 (3H, d, *J* = 6.9 Hz), 0.08 (3H, s), 0.06 (6H, s), 0.04 (3H, s), 0.01 (6H, s); ^13^C{^1^H} NMR (101 MHz, CDCl_3_) δ 179.9, 178.6, 145.8, 139.9, 124.9, 115.1, 79.0, 78.8, 78.2, 77.2, 71.3, 70.6, 64.5, 62.6, 48.7, 40.8, 39.2, 38.8, 37.0, 36.4, 35.4, 30.2, 27.5, 27.3, 26.3, 26.1, 26.1, 18.6, 18.5, 18.2, 18.2, 14.3, 11.8, 9.9, −3.9, −4.0 (2C), −4.1, −4.6, −4.6, −4.6; HRMS (ESI+) *m*/*z*: [M + Na]^+^ calcd for C_50_H_98_NaO_9_Si_3_ 949.6411; found 949.6366.

### Amphidinolide C1–C17 Iodide (**46**)

The alcohol (78 mg, 0.084 mmol) was dissolved in benzene (2 mL), and the resulting solution was cooled to 5 °C. Triphenylphosphine (66 mg, 0.252 mmol), imidazole (34 mg, 0.50 mmol), and iodine (64 mg, 0.25 mmol) were added sequentially, and the resulting mixture was stirred at 5 °C for 10 min. The reaction mixture was warmed to rt, wrapped in aluminum foil, and stirred at this temperature for a further 2 h. The reaction was quenched by the addition of saturated aqueous sodium sulfite (5 mL), and the mixture was extracted with diethyl ether (3 × 5 mL). The combined organic extracts were dried (anhydrous MgSO_4_), filtered, and concentrated under reduced pressure. The resulting residue was purified quickly by flash chromatography on silica gel (pet. ether-ethyl acetate, 50:1) to afford iodide **46** (81 mg, 93%) as a colorless oil. *R_f_* = 0.86 (pet. ether-ethyl acetate, 9:1); [α]_D_^24^ +5.2 (*c* = 1.0, CHCl_3_); ^1^H NMR (500 MHz, CDCl_3_) δ 5.73 (1H, br s), 5.29 (1H, br s), 5.00 (1H, dt, *J* = 9.7, 2.6 Hz), 4.92 (1H, br s), 4.22–4.17 (1H, m), 4.21–4.06 (3H, m), 3.89–3.85 (1H, m), 3.84 (1H, ddd, *J* = 9.6, 3.5, 1.5 Hz), 3.50 (1H, dd, *J* = 6.8, 3.3 Hz), 3.25 (1H, dd, *J* = 9.7, 4.4 Hz), 2.85 (1H, t, *J* = 9.7 Hz), 2.43–2.34 (1H, m), 2.21–2.16 (1H, m), 2.08–2.00 (1H, m), 1.87–1.68 (3H, m), 1.79 (3H, br s), 1.62 (1H, ddd, *J* = 12.2, 6.4, 1.7 Hz), 1.53 (1H, ddd, *J* = 13.8, 9.7, 1.5 Hz), 1.47–1.40 (1H, m), 1.19 (18H, s), 1.04 (3H, d, *J* = 6.7 Hz), 1.03 (3H, d, *J* = 6.7 Hz), 0.91 (9H, s), 0.90 (9H, s), 0.90 (3H, d, *J* = 7.0 Hz), 0.88 (9H, s), 0.09 (3H, s), 0.08 (3H, s), 0.07 (3H, s), 0.05 (3H, s), 0.01 (3H, s), 0.01 (3H, s); ^13^C{^1^H} NMR (126 MHz, CDCl_3_) δ 178.6, 177.8, 145.8, 139.9, 124.8, 115.2, 79.0, 78.9, 78.2, 77.2, 73.8, 71.0, 62.6, 48.7, 41.2, 39.1, 38.8, 37.0, 36.4, 33.8, 30.3, 27.4, 27.4, 26.3, 26.1, 26.1, 18.6, 18.5, 18.4, 18.2, 15.8, 14.3, 11.6, 9.8, −4.0, −4.0, −4.1, −4.5, −4.5, −4.6; HRMS (ESI+) *m*/*z*: [M + H]^+^ calcd for C_50_H_98_IO_8_Si_3_ 1037.5609; found 1037.5586.

### Amphidinolide C1–C29 Fragment (**47**)

Dithiane **40** (50 mg, 0.117 mmol) was dissolved in a mixture of THF and HMPA (4.5:1, 430 μL), and the resulting solution was cooled to −78 °C. *t*-Butyllithium (47 μL of a 2.5 M solution in hexanes, 0.117 mmol) was added, and the resulting orange/red mixture was stirred at −78 °C for 10 min. A solution of iodide **46** (81 mg, 0.078 mmol) in THF (350 μL) was added, and the reaction was stirred at −78 °C for 1 h. The reaction was quenched by the addition of aqueous pH 7 buffer (2 mL) and extracted with diethyl ether (3 × 2 mL). The combined organic layers were dried (anhydrous MgSO_4_), filtered, and concentrated under reduced pressure. The resulting residue was purified by flash chromatography on silica gel (pet. ether-ethyl acetate, 50:1 to 20:1) to afford the desired coupled product **47** (14 mg, 13%) as a colorless oil along with recovered dithiane **40** (21 mg, 42%) and recovered iodide **46** (40 mg, 50%). *R_f_* = 0.31 (pet. ether-ethyl acetate, 19:1); [α]_D_^28^ +8.7 (*c* = 0.7, CHCl_3_); ν_max_. 2956, 2930, 2878, 2857, 1728, 835, 776 cm^–1^; ^1^H NMR (400 MHz, CDCl_3_) δ 6.45 (1H, ddd, *J* = 15.3, 10.9, 0.9 Hz), 5.86–5.80 (1H, m), 5.72 (1H, bs), 5.55 (1H, dd, *J* = 15.3, 5.8 Hz), 5.28 (1H, dd, *J* = 2.2, 1.3 Hz), 5.03 (1H, dt, *J* = 9.6, 2.5 Hz), 4.91–4.89 (1H, m), 4.23–4.16 (2H, m), 4.16–4.04 (4H, m), 3.94 (1H, dd, *J* = 13.3, 7.1 Hz), 3.86 (1H, dt, *J* = 8.2, 5.1 Hz), 3.79–3.73 (1H, m), 3.50 (1H, dd, *J* = 7.0, 3.0 Hz), 2.93–2.84 (1H, m), 2.81–2.71 (3H, m), 2.40–2.33 (1H, m), 2.27 (1H, dd, *J* = 15.0, 4.5 Hz), 2.22–2.08 (3H, m), 2.06–1.45 (13H, m), 1.77 (6H, bs), 1.75 (3H, d, *J* = 0.6 Hz), 1.19 (9H, s), 1.19 (9H, s), 1.04 (3H, d, *J* = 6.9 Hz), 1.03 (3H, d, *J* = 6.9 Hz), 0.95 (9H, t, *J* = 7.9 Hz), 0.91 (18H, s), 0.90 (3H, d, *J* = 6.2 Hz), 0.88 (9H, s), 0.60 (6H, q, *J* = 7.9 Hz), 0.11 (3H, s), 0.08 (3H, s,), 0.07 (3H, s), 0.05 (3H, s), 0.01 (6H, s); ^13^C{^1^H} NMR (101 MHz, CDCl_3_) δ 178.7, 178.2, 145.8, 140.2, 134.8, 130.0, 27.6, 125.0, 124.7, 114.8, 81.7, 79.0, 78.9, 78.3, 77.0, 76.4, 76.2, 75.3, 71.8, 65.3, 62.7, 48.3, 46.5, 44.2, 42.4, 41.2, 39.1, 38.9, 37.6, 36.4, 34.5, 34.2, 31.0, 30.5, 30.3, 27.6, 27.4, 27.4, 26.4, 26.3, 26.2, 26.2, 26.1, 18.6, 18.5, 18.4, 18.3, 18.0, 16.8, 14.3, 12.9, 7.1, 5.1, −4.1, −4.2, −4.3, −4.5, −4.6; HRMS (ESI+) *m*/*z*: [M + Na]^+^ calcd for C_72_H_136_NaO_10_S_2_Si_4_ 1359.8544; found 1359.8398.

## Supporting Information Available

Copies of ^1^H and ^13^C NMR data for new compounds (PDF)
