# Supplementary material for: Convergent Synthesis of the C1–C29 Framework of Amphidinolide F
Source: J Org Chem. 2022 Jun 8;87(12):8126–41. doi: 10.1021/acs.joc.2c00850 (PMC9207912; doi:10.1021/acs.joc.2c00850)

Convergent Synthesis of the C1–C29 Framework of Amphidinolide F

Filippo Romiti, Ludovic Decultot and J. Stephen Clark*

School of Chemistry, University of Glasgow, Joseph Black Building, University Avenue, Glasgow G12 8QQ, United Kingdom

Supporting Information

^{1}H and ^{13}C NMR Spectra for New Compounds

		Page
^{1}H NMR Spectrum of **2**	(500 MHz, $CDCl_3$)	SI-4
$^{13}C\{^{1}H\}$ NMR Spectrum of **2**	(126 MHz, $CDCl_3$)	SI-5
^{1}H NMR Spectrum of **3**	(400 MHz, $CDCl_3$)	SI-6
$^{13}C\{^{1}H\}$ NMR Spectrum of **3**	(101 MHz, $CDCl_3$)	SI-7
^{1}H NMR Spectrum of **4**	(500 MHz, $CDCl_3$)	SI-8
$^{13}C\{^{1}H\}$ NMR Spectrum of **4**	(126 MHz, $CDCl_3$)	SI-9
^{1}H NMR Spectrum of **5**	(400 MHz, $CDCl_3$)	SI-10
$^{13}C\{^{1}H\}$ NMR Spectrum of **5**	(101 MHz, $CDCl_3$)	SI-11
^{1}H NMR Spectrum of **6**	(500 MHz, $CDCl_3$)	SI-12
$^{13}C\{^{1}H\}$ NMR Spectrum of **6**	(126 MHz, $CDCl_3$)	SI-13
^{1}H NMR Spectrum of **8**	(400 MHz, $CDCl_3$)	SI-14
$^{13}C\{^{1}H\}$ NMR Spectrum of **8**	(101 MHz, $CDCl_3$)	SI-15
^{1}H NMR Spectrum of **9**	(400 MHz, $CDCl_3$)	SI-16
$^{13}C\{^{1}H\}$ NMR Spectrum of **9**	(101 MHz, $CDCl_3$)	SI-17
^{1}H NMR Spectrum of **11**	(500 MHz, $CDCl_3$)	SI-18
$^{13}C\{^{1}H\}$ NMR Spectrum of **11**	(126 MHz, $CDCl_3$)	SI-19
^{1}H NMR Spectrum of **13**	(500 MHz, $CDCl_3$)	SI-20
$^{13}C\{^{1}H\}$ NMR Spectrum of **13**	(126 MHz, $CDCl_3$)	SI-21
^{1}H NMR Spectrum of **15**	(500 MHz, $CDCl_3$)	SI-22
$^{13}C\{^{1}H\}$ NMR Spectrum of **15**	(126 MHz, $CDCl_3$)	SI-23
^{1}H NMR Spectrum of **16**	(500 MHz, $CDCl_3$)	SI-24
$^{13}C\{^{1}H\}$ NMR Spectrum of **16**	(126 MHz, $CDCl_3$)	SI-25
^{1}H NMR Spectrum of **19**	(500 MHz, $CDCl_3$)	SI-26
$^{13}C\{^{1}H\}$ NMR Spectrum of **19**	(126 MHz, $CDCl_3$)	SI-27
^{1}H NMR Spectrum of **20**	(400 MHz, $CDCl_3$)	SI-28
$^{13}C\{^{1}H\}$ NMR Spectrum of **20**	(101 MHz, $CDCl_3$)	SI-29
^{1}H NMR Spectrum of **21**	(400 MHz, $CDCl_3$)	SI-30
$^{13}C\{^{1}H\}$ NMR Spectrum of **21**	(101 MHz, $CDCl_3$)	SI-31
^{1}H NMR Spectrum of **23**	(400 MHz, $CDCl_3$)	SI-32
$^{13}C\{^{1}H\}$ NMR Spectrum of **23**	(101 MHz, $CDCl_3$)	SI-33
^{1}H NMR Spectrum of **24**	(400 MHz, $CDCl_3$)	SI-34
$^{13}C\{^{1}H\}$ NMR Spectrum of **24**	(101 MHz, $CDCl_3$)	SI-35
^{1}H NMR Spectrum of **25**	(400 MHz, $CDCl_3$)	SI-36
$^{13}C\{^{1}H\}$ NMR Spectrum of **25**	(101 MHz, $CDCl_3$)	SI-37
^{1}H NMR Spectrum of **27**	(400 MHz, $CDCl_3$)	SI-38
$^{13}C\{^{1}H\}$ NMR Spectrum of **27**	(101 MHz, $CDCl_3$)	SI-39

^{1}H NMR Spectrum of **28**	(500 MHz, $CDCl_3$)	SI-40
$^{13}C\{^{1}H\}$ NMR Spectrum of **28**	(126 MHz, $CDCl_3$)	SI-41
^{1}H NMR Spectrum of **29**	(500 MHz, $CDCl_3$)	SI-42
$^{13}C\{^{1}H\}$ NMR Spectrum of **29**	(126 MHz, $CDCl_3$)	SI-43
^{1}H NMR Spectrum of **30**	(400 MHz, $CDCl_3$)	SI-44
$^{13}C\{^{1}H\}$ NMR Spectrum of **30**	(126 MHz, $CDCl_3$)	SI-45
^{1}H NMR Spectrum of **31**	(400 MHz, $CDCl_3$)	SI-46
$^{13}C\{^{1}H\}$ NMR Spectrum of **31**	(126 MHz, $CDCl_3$)	SI-47
^{1}H NMR Spectrum of **33**	(400 MHz, $CDCl_3$)	SI-48
$^{13}C\{^{1}H\}$ NMR Spectrum of **33**	(126 MHz, $CDCl_3$)	SI-49
^{1}H NMR Spectrum of **35**	(500 MHz, $CDCl_3$)	SI-50
$^{13}C\{^{1}H\}$ NMR Spectrum of **35**	(126 MHz, $CDCl_3$)	SI-51
^{1}H NMR Spectrum of 2-{(2*R*,5*R*)-5-[(*R*)-1-hydroxy-5-methylhex-4-en-2-yn-1-yl]-tetrahydrofuran-2-yl}ethyl 2,2-dimethylpropanoate (400 MHz, $CDCl_3$)		SI-52
$^{13}C\{^{1}H\}$ NMR Spectrum of 2-{(2*R*,5*R*)-5-[(*R*)-1-hydroxy-5-methylhex-4-en-2-yn-1-yl]-tetrahydrofuran-2-yl}ethyl 2,2-dimethylpropanoate (126 MHz, $CDCl_3$)		SI-53
^{1}H NMR Spectrum of **36**	(500 MHz, $CDCl_3$)	SI-54
$^{13}C\{^{1}H\}$ NMR Spectrum of **36**	(126 MHz, $CDCl_3$)	SI-55
^{1}H NMR Spectrum of **37**	(500 MHz, $CDCl_3$)	SI-56
$^{13}C\{^{1}H\}$ NMR Spectrum of **37**	(126 MHz, $CDCl_3$)	SI-57
^{1}H NMR Spectrum of **38**	(400 MHz, $CDCl_3$)	SI-58
^{1}H NMR Spectrum of **40**	(400 MHz, $CDCl_3$)	SI-59
$^{13}C\{^{1}H\}$ NMR Spectrum of **40**	(126 MHz, $CDCl_3$)	SI-60
^{1}H NMR Spectrum of **43**	(400 MHz, $CDCl_3$)	SI-61
$^{13}C\{^{1}H\}$ NMR Spectrum of **43**	(101 MHz, $CDCl_3$)	SI-62
^{1}H NMR Spectrum of **44**	(500 MHz, $CDCl_3$)	SI-63
$^{13}C\{^{1}H\}$ NMR Spectrum of **44**	(101 MHz, $CDCl_3$)	SI-64
^{1}H NMR Spectrum of **45**	(400 MHz, $CDCl_3$)	SI-65
^{1}H NMR Spectrum of 2-{(2*S*,3*R*,5*R*)-5-[(1*S*,2*R*,6*R*,7*S*,9*S*,10*S*,*E*)-9-(2,2-dimethyl-1-oxopropoxy)-11-hydroxy-3-methylene-5,6,10-trimethyl-1,2,7-tris(*tert*-butyldimethylsilyloxy)undec-4-en-1-yl]-3-methyltetrahydrofuran-2-yl}ethyl 2,2-dimethylpropanoate (400 MHz, $CDCl_3$)		SI-66
$^{13}C\{^{1}H\}$ NMR Spectrum of 2-{(2*S*,3*R*,5*R*)-5-[(1*S*,2*R*,6*R*,7*S*,9*S*,10*S*,*E*)-9-(2,2-dimethyl-1-oxopropoxy)-11-hydroxy-3-methylene-5,6,10-trimethyl-1,2,7-tris(*tert*-butyldimethylsilyloxy)undec-4-en-1-yl]-3-methyltetrahydrofuran-2-yl}ethyl 2,2-dimethyl-propanoate (101 MHz, $CDCl_3$)		SI-67
^{1}H NMR Spectrum of **46**	(500 MHz, $CDCl_3$)	SI-68
$^{13}C\{^{1}H\}$ NMR Spectrum of **46**	(126 MHz, $CDCl_3$)	SI-69
^{1}H NMR Spectrum of **47**	(400 MHz, $CDCl_3$)	SI-70
$^{13}C\{^{1}H\}$ NMR Spectrum of **47**	(101 MHz, $CDCl_3$)	SI-71

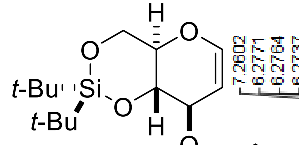

2

^{1}H NMR (500 MHz, $CDCl_3$)

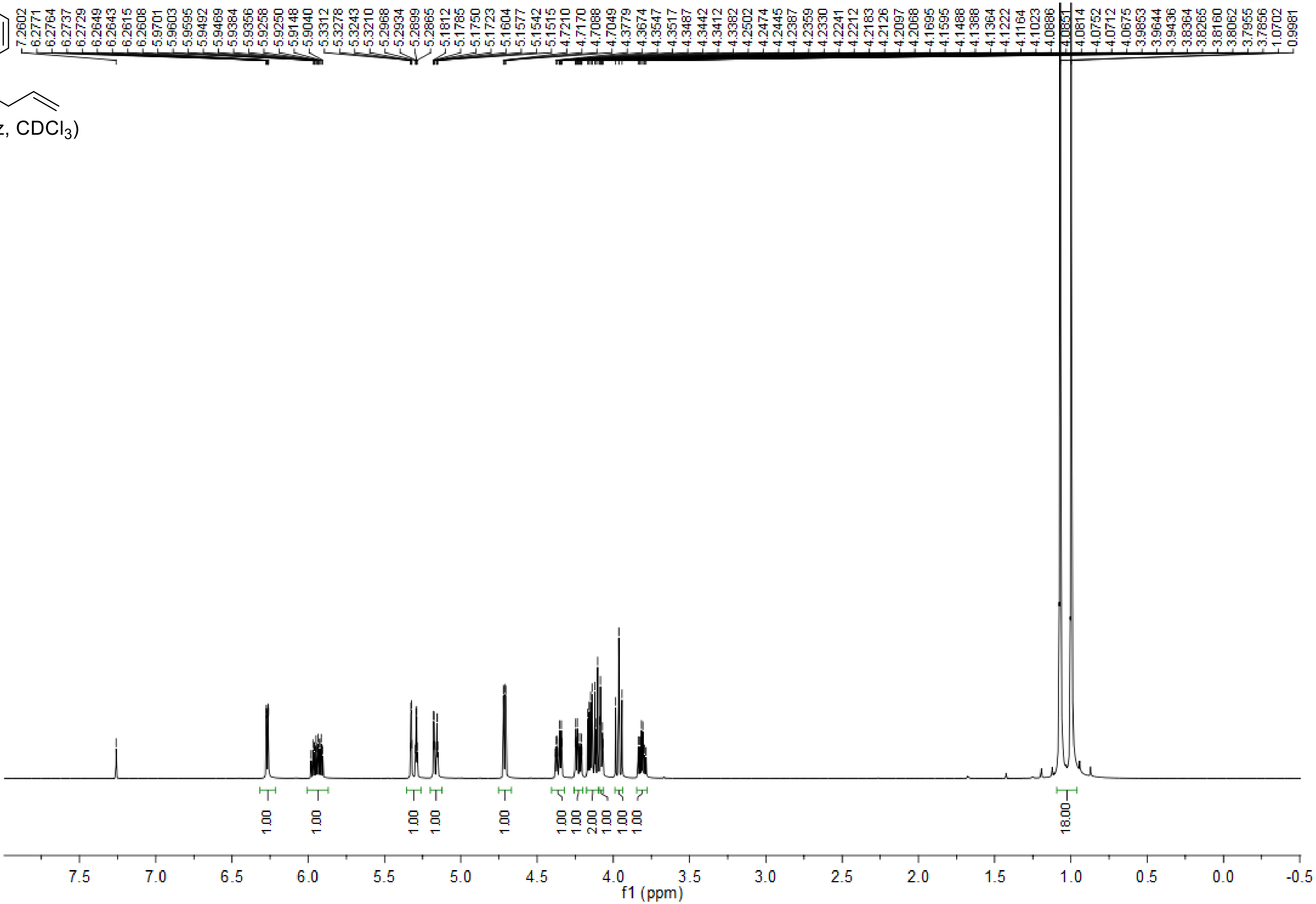

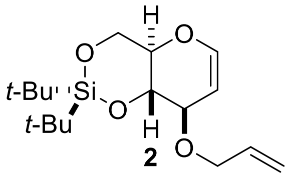

$^{13}C\{^1H\}$ NMR (126 MHz, $CDCl_3$)

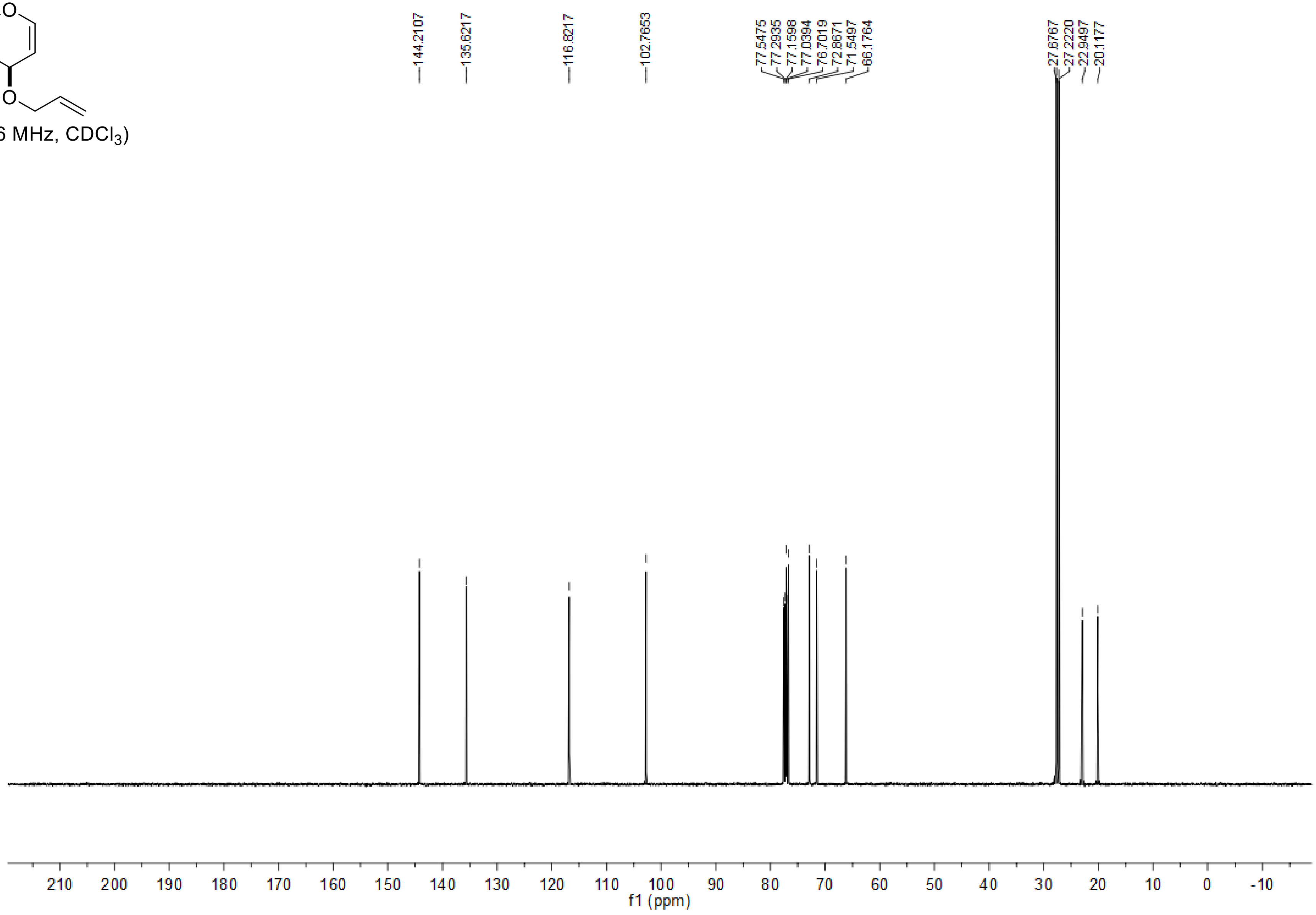

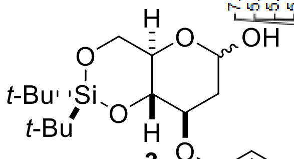

3

^{1}H NMR (400 MHz, $CDCl_3$)

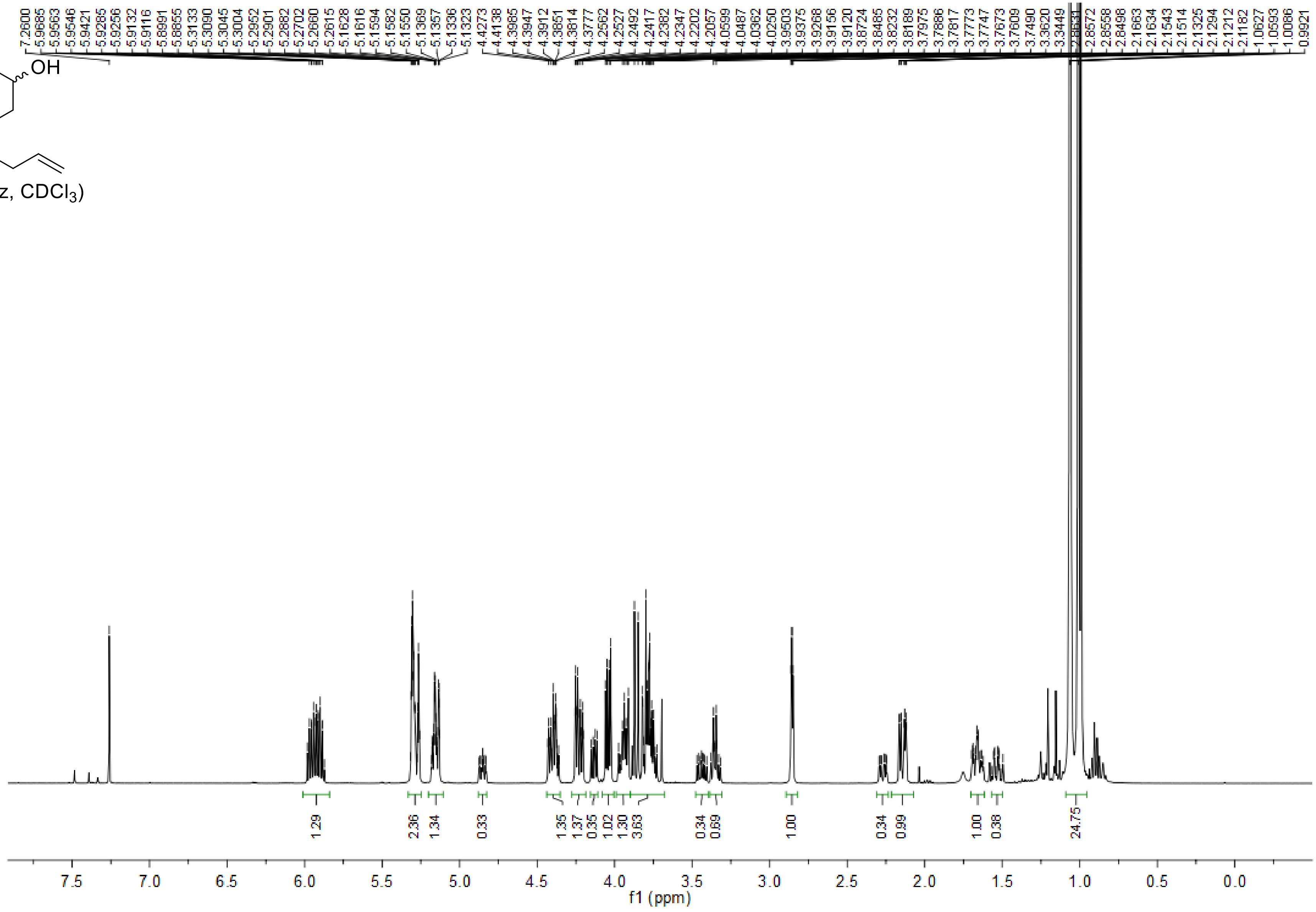

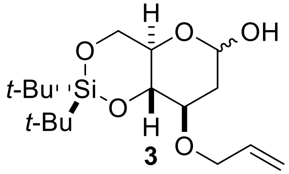

3

$^{13}C\{^{1}H\}$ NMR (101 MHz, $CDCl_3$)

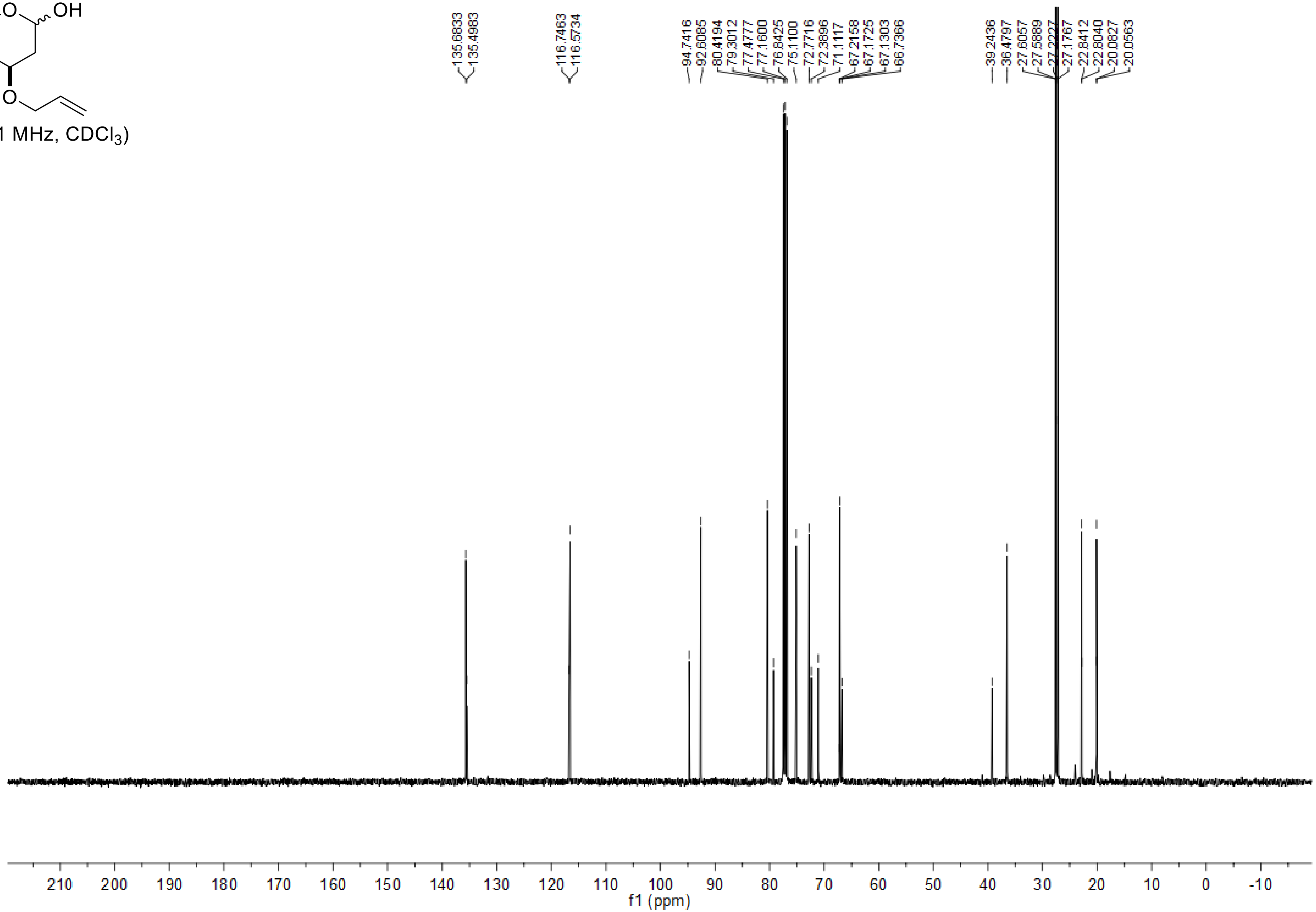

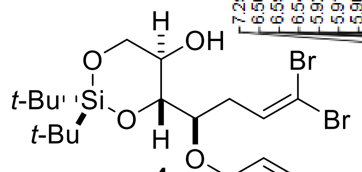

4

^{1}H NMR (500 MHz, $CDCl_3$)

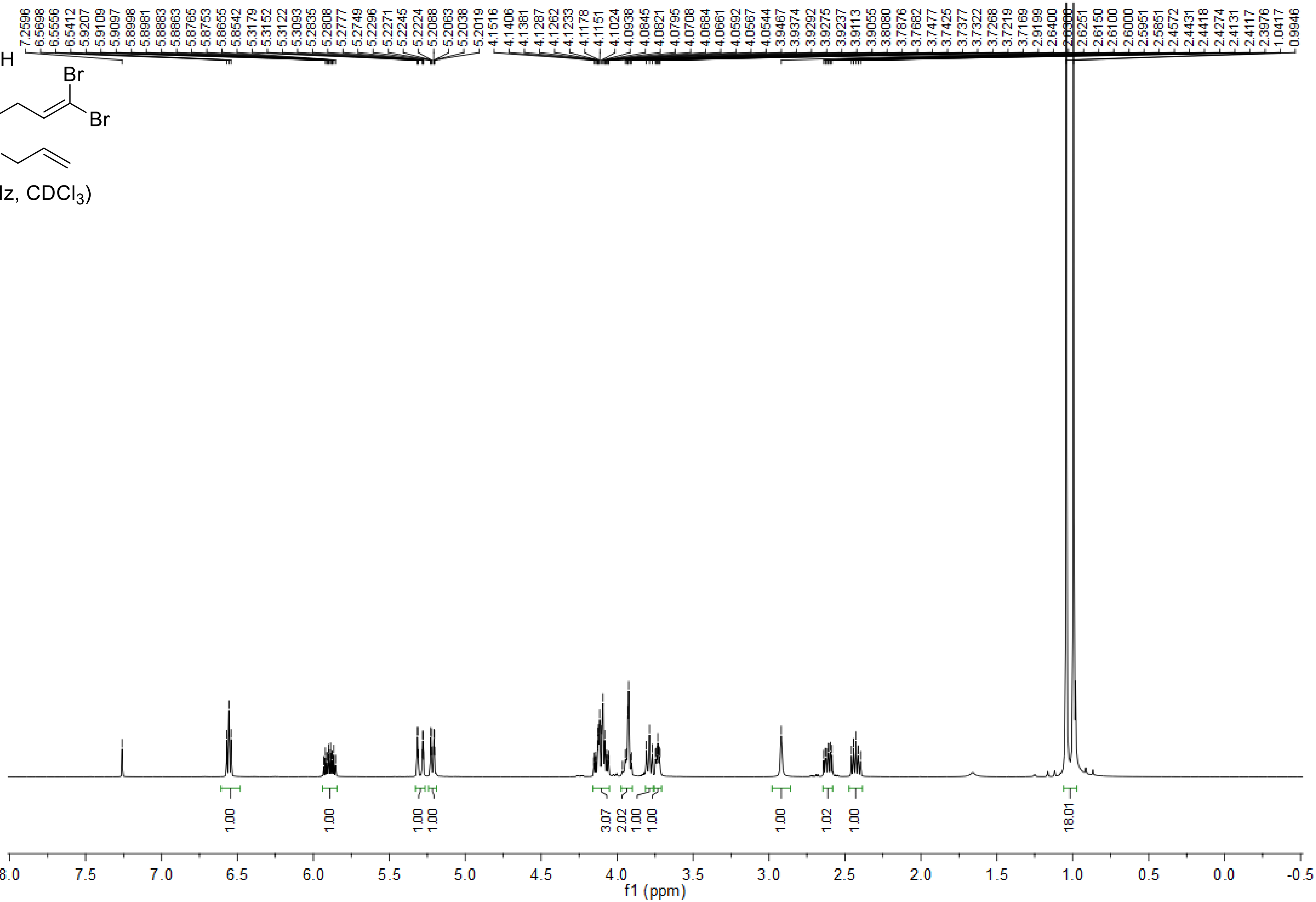

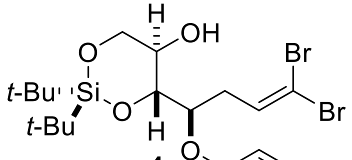

$^{13}C\{^1H\}$ NMR (126 MHz, $CDCl_3$)

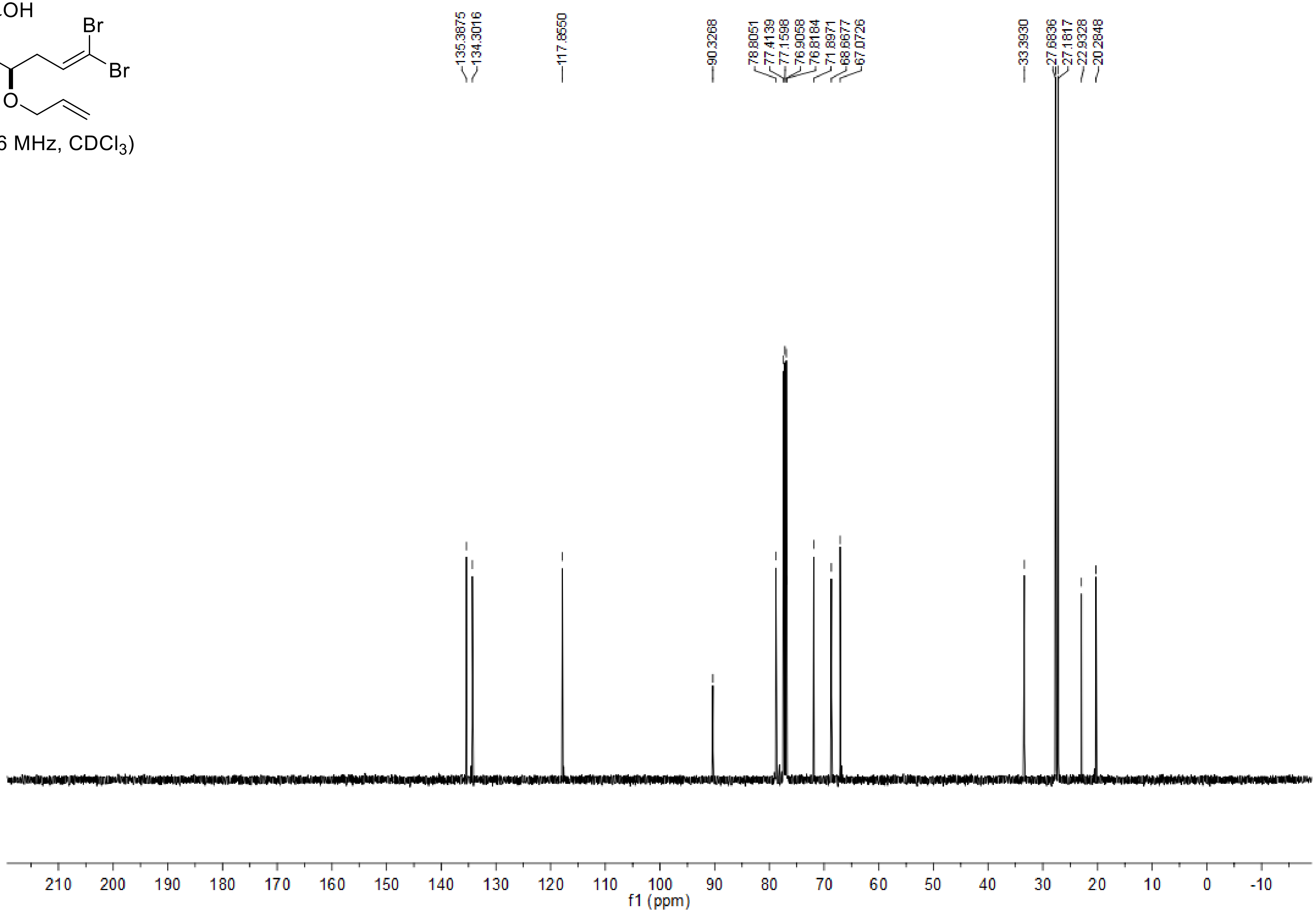

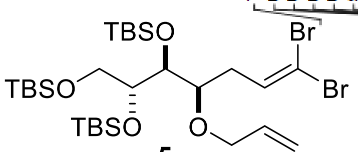

5

^{1}H NMR (400 MHz, $CDCl_3$)

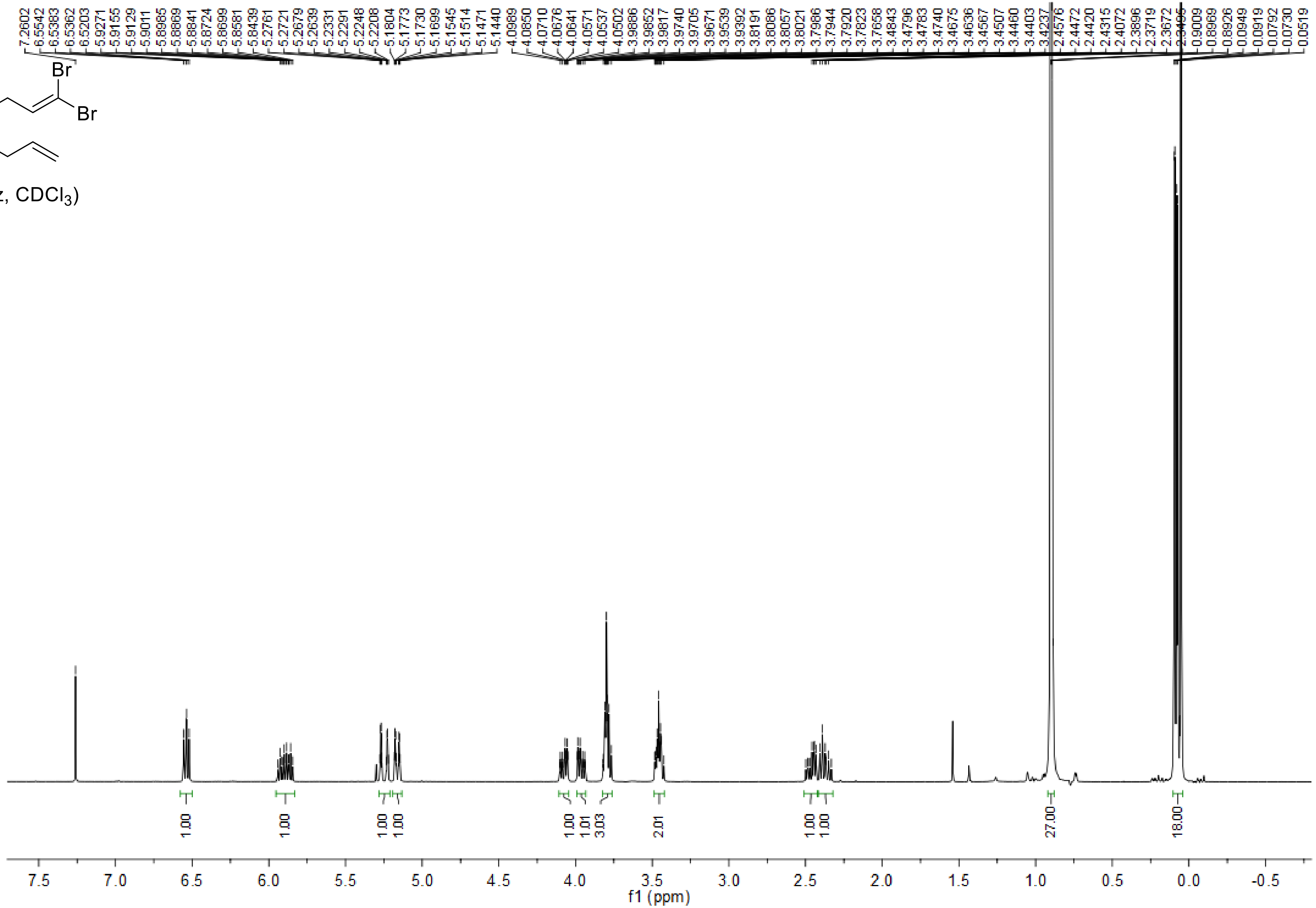

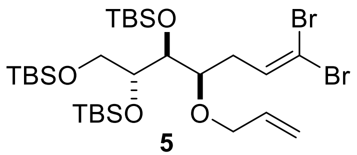

5

$^{13}C\{^{1}H\}$ NMR (101 MHz, $CDCl_3$)

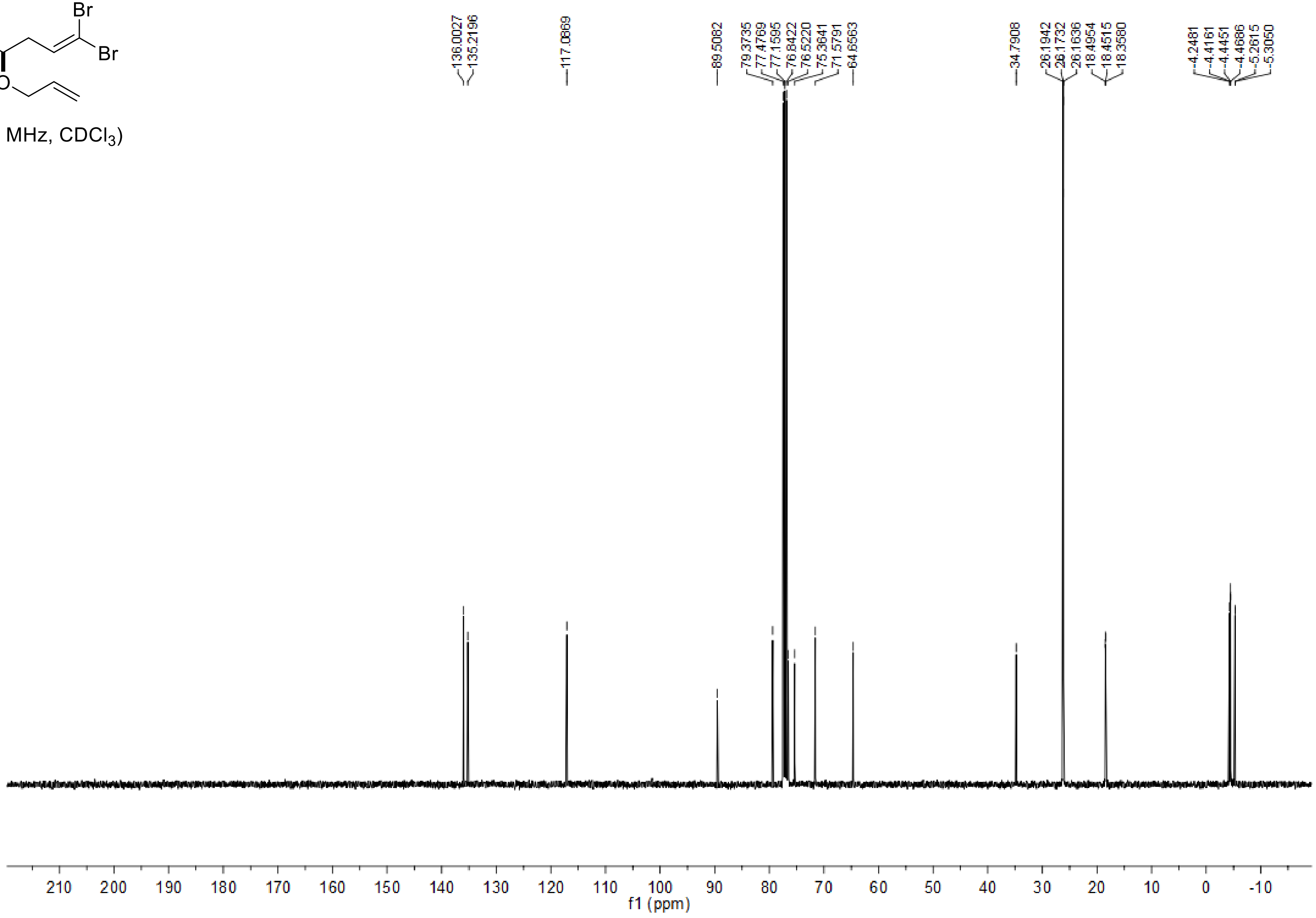

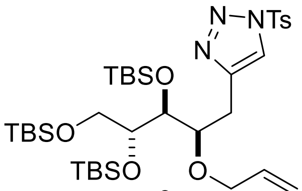

6

^{1}H NMR (500 MHz, $CDCl_3$)

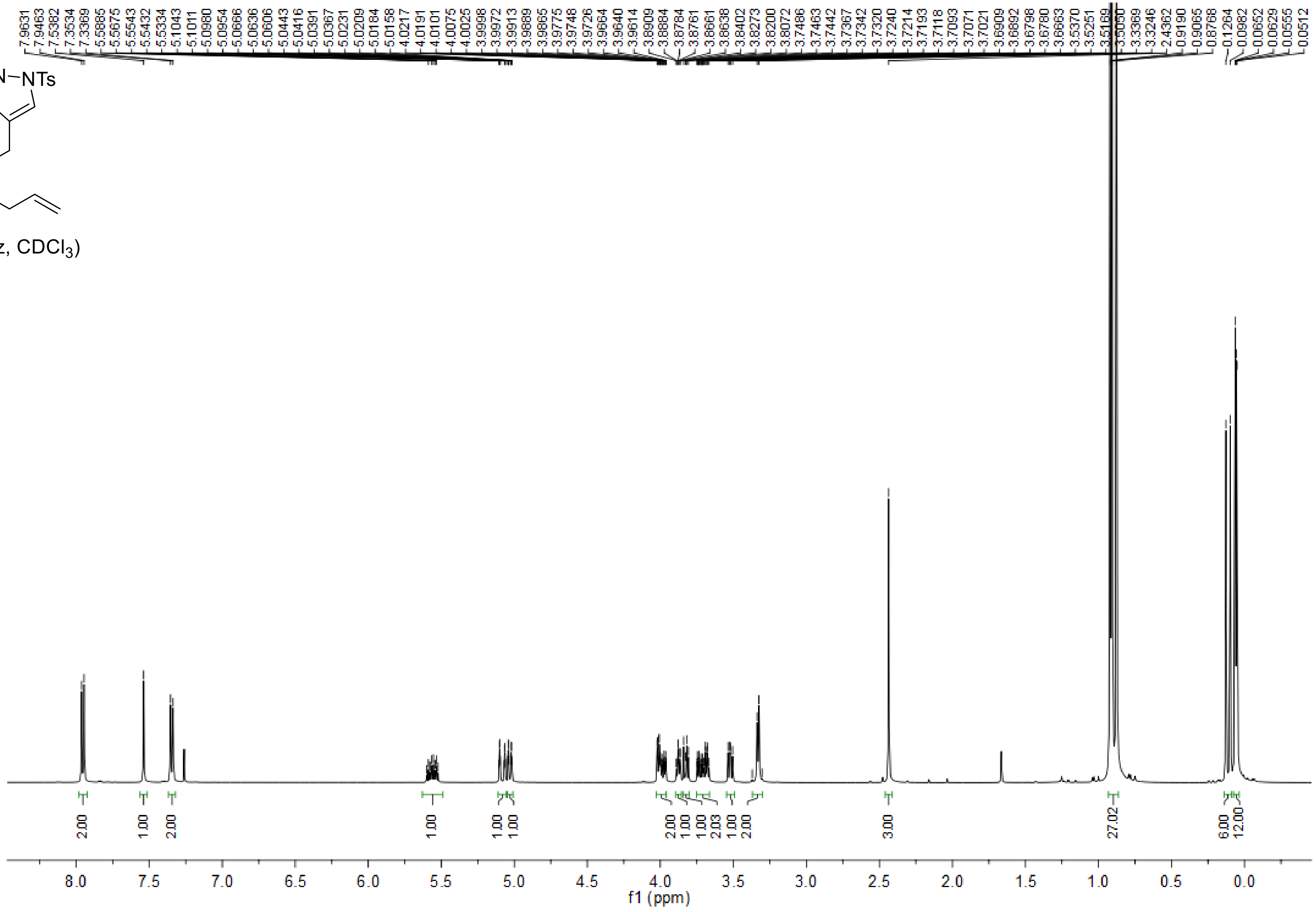

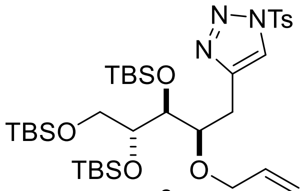

6

$^{13}C\{^{1}H\}$ NMR (126 MHz, $CDCl_3$)

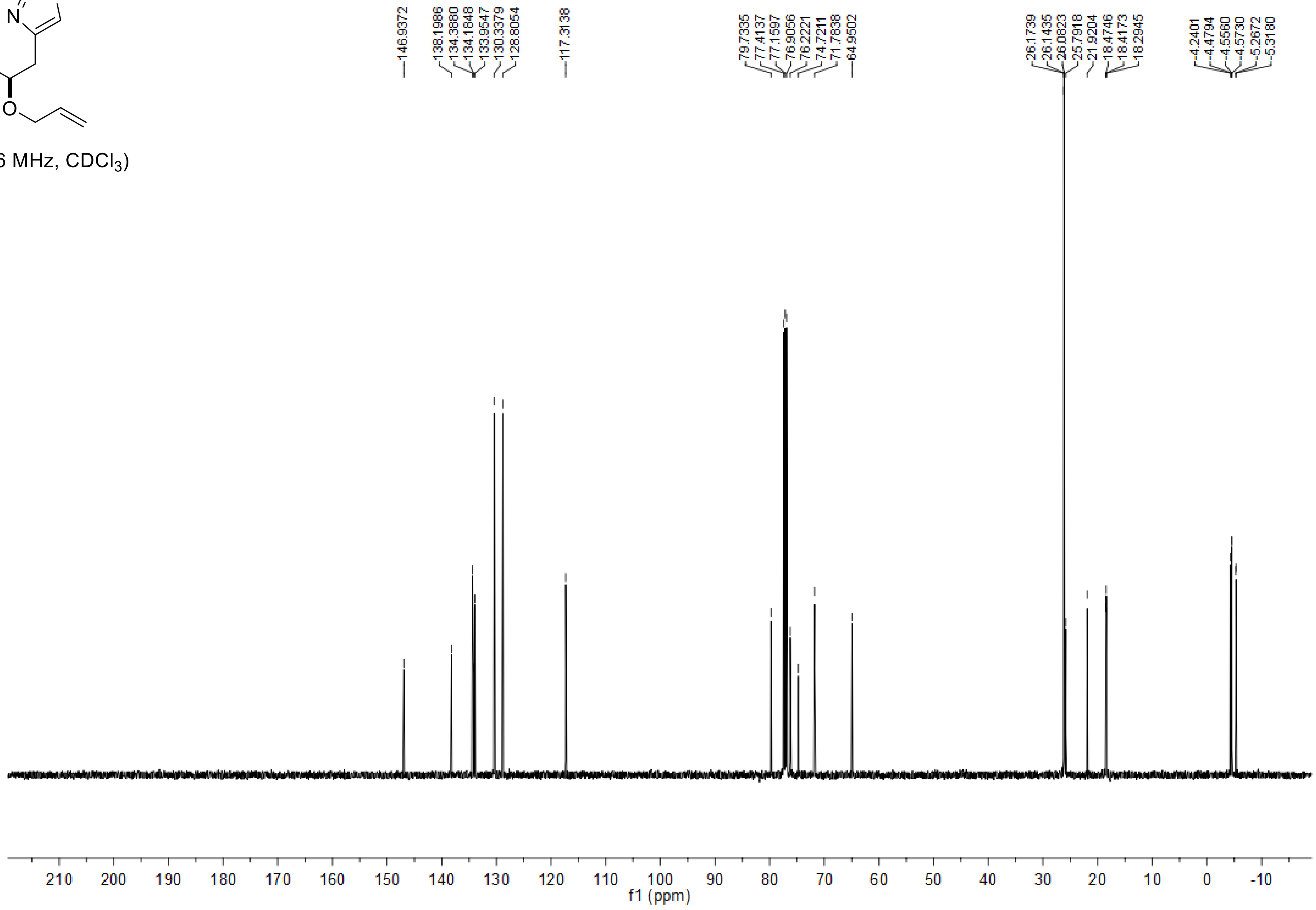

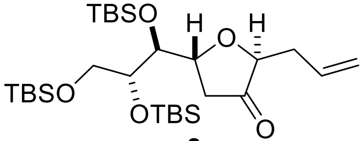

8

^{1}H NMR (400 MHz, $CDCl_3$)

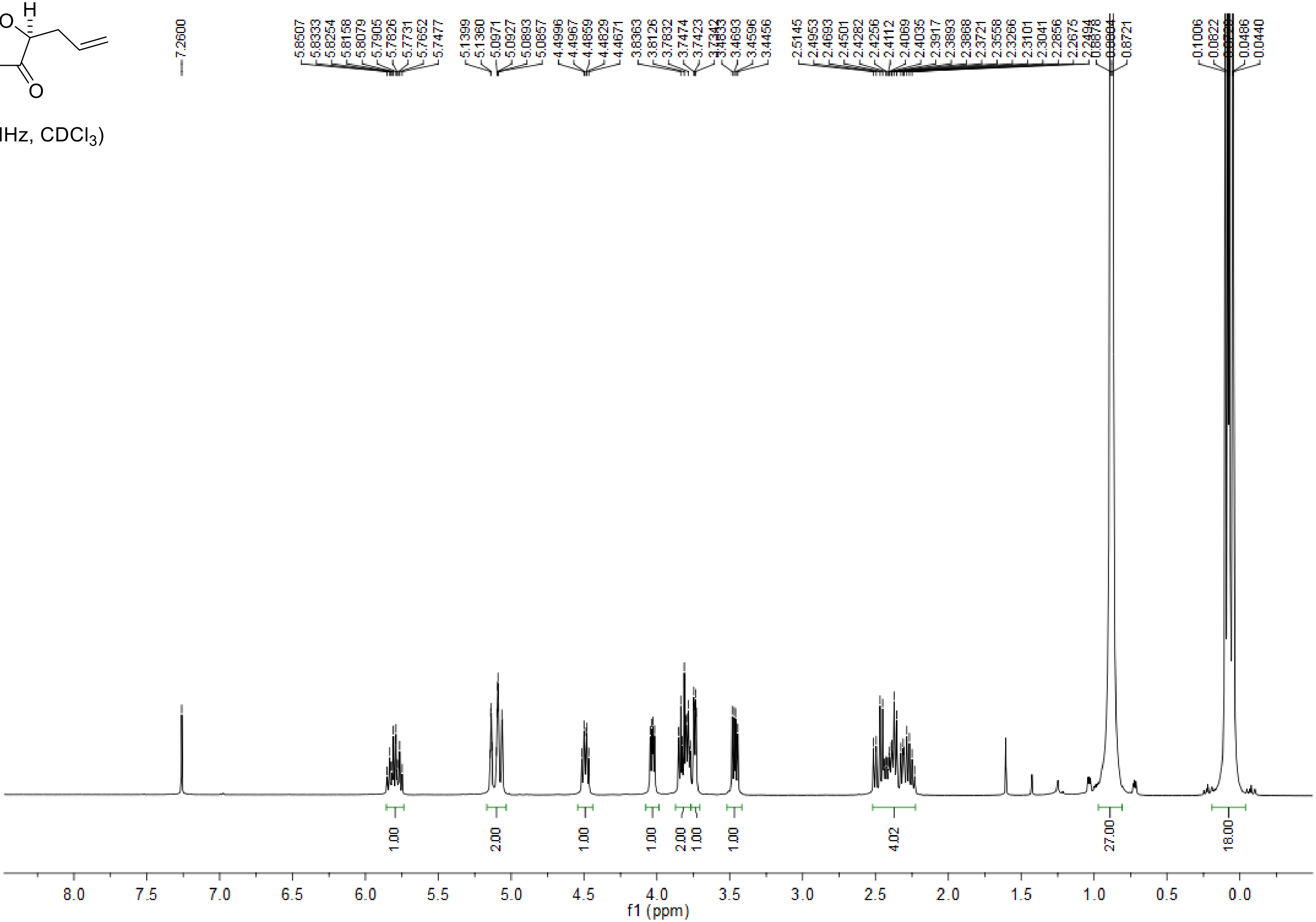

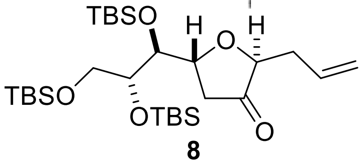

8

$^{13}C\{^1H\}$ NMR (101 MHz, $CDCl_3$)

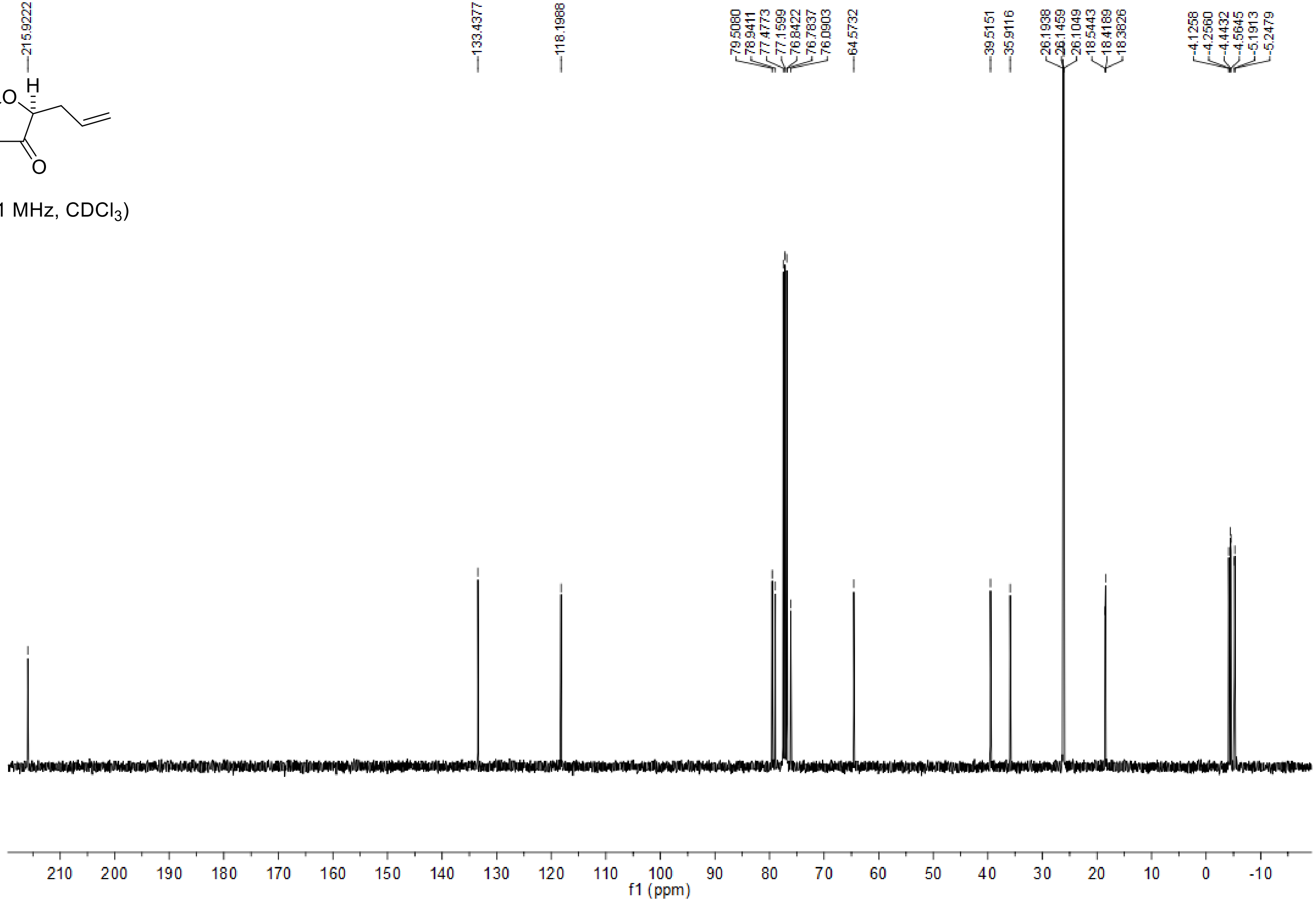

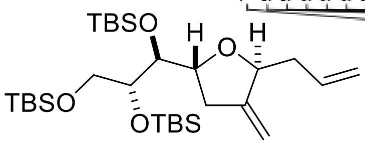

9

^{1}H NMR (400 MHz, $CDCl_3$)

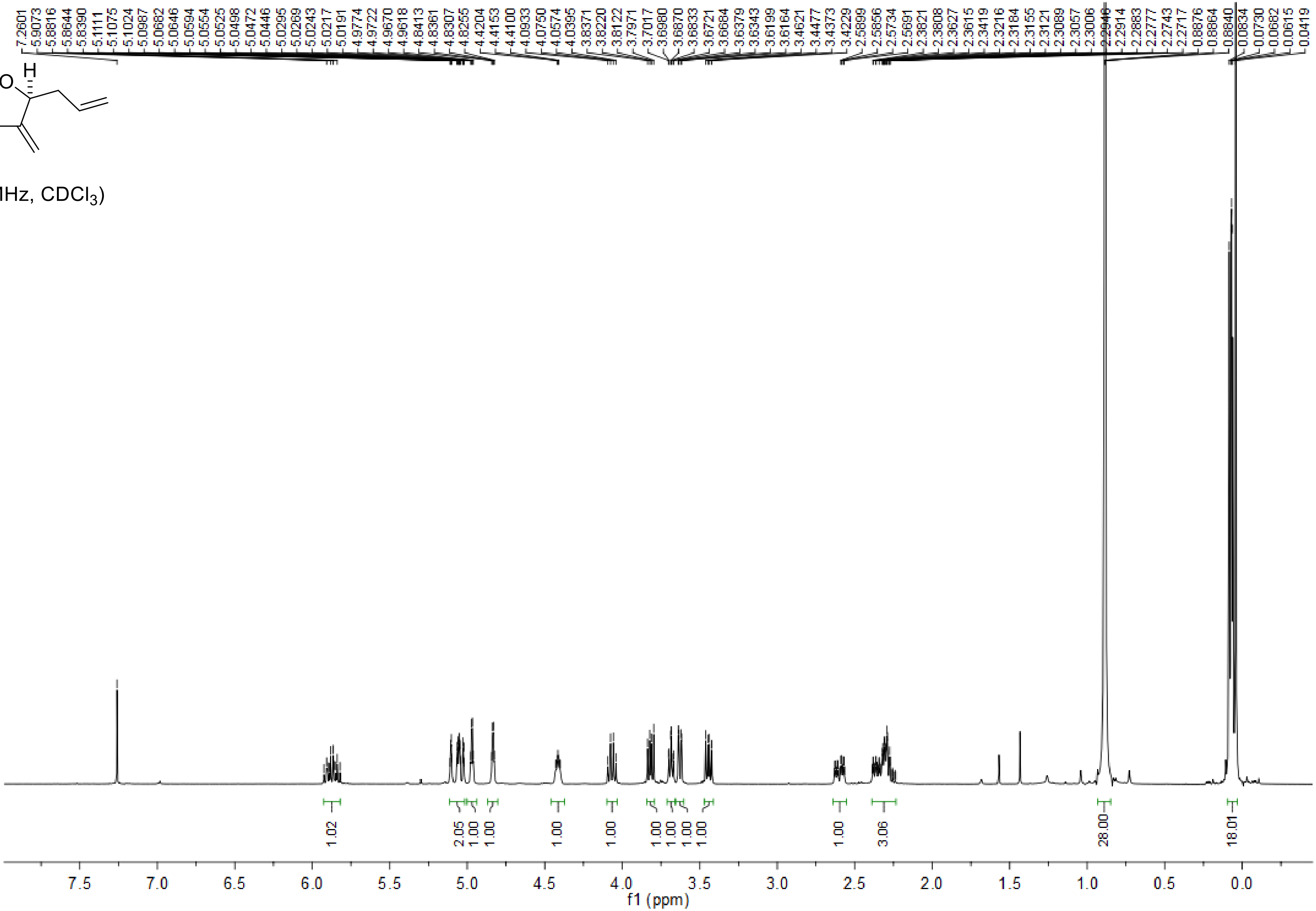

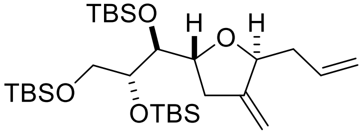

9

$^{13}C\{^{1}H\}$ NMR (101 MHz, $CDCl_3$)

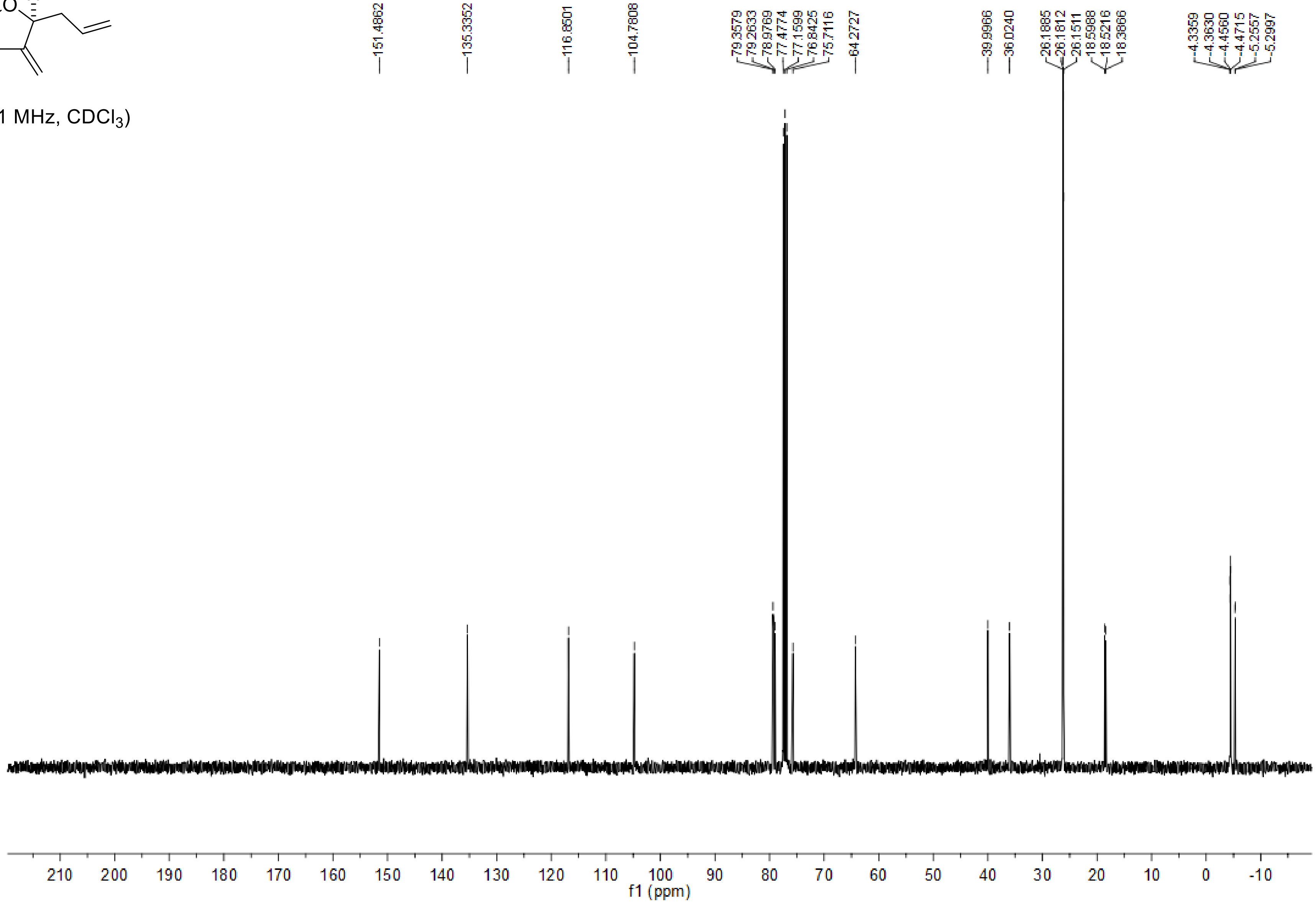

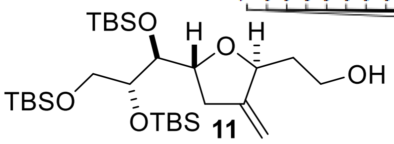

^{1}H NMR (500 MHz, $CDCl_3$)

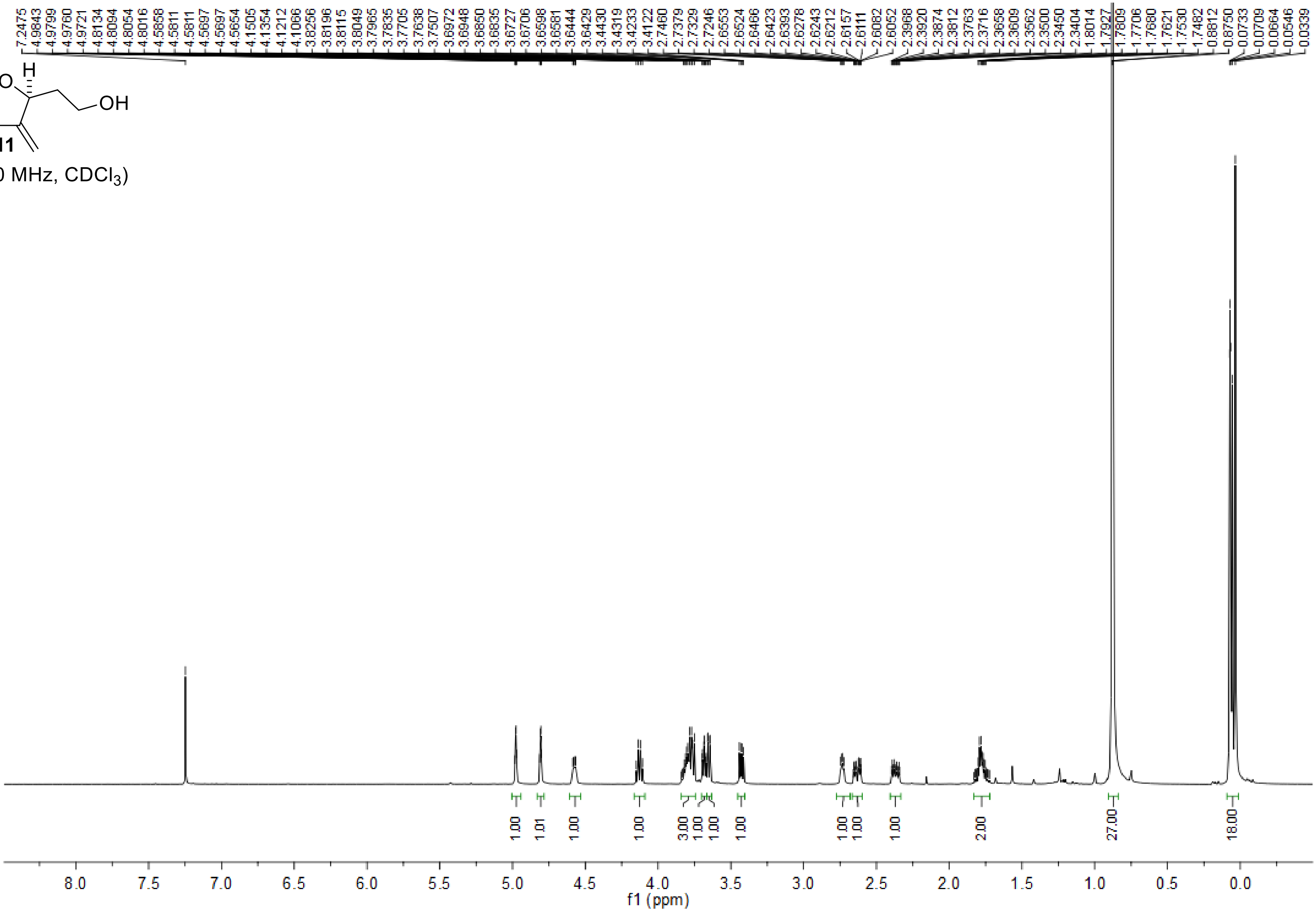

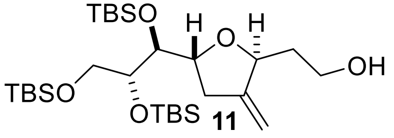

$^{13}C\{^{1}H\}$ NMR (126 MHz, $CDCl_3$)

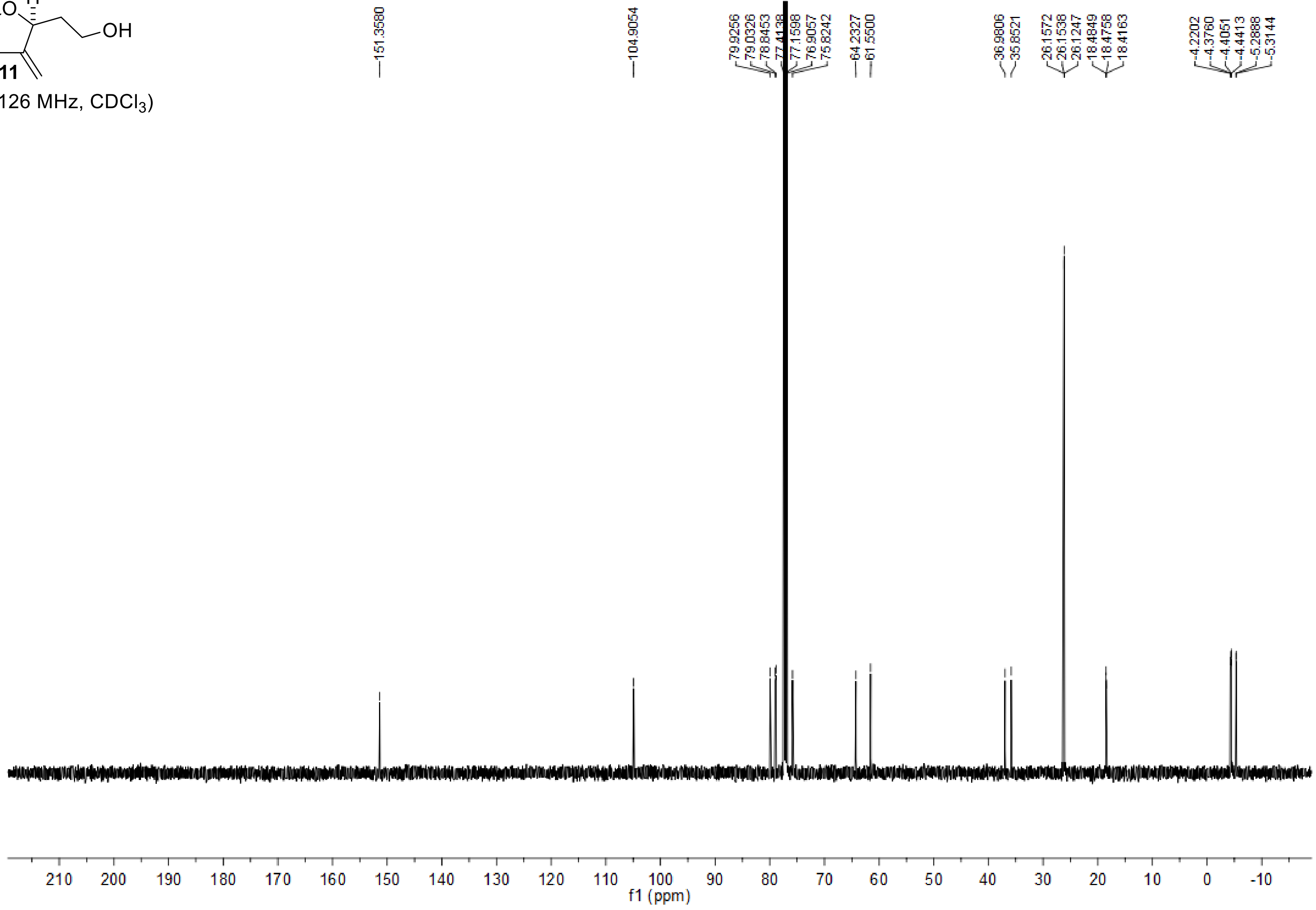

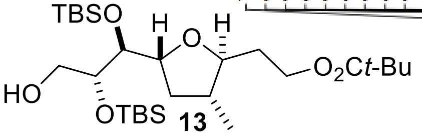

^{1}H NMR (500 MHz, $CDCl_3$)

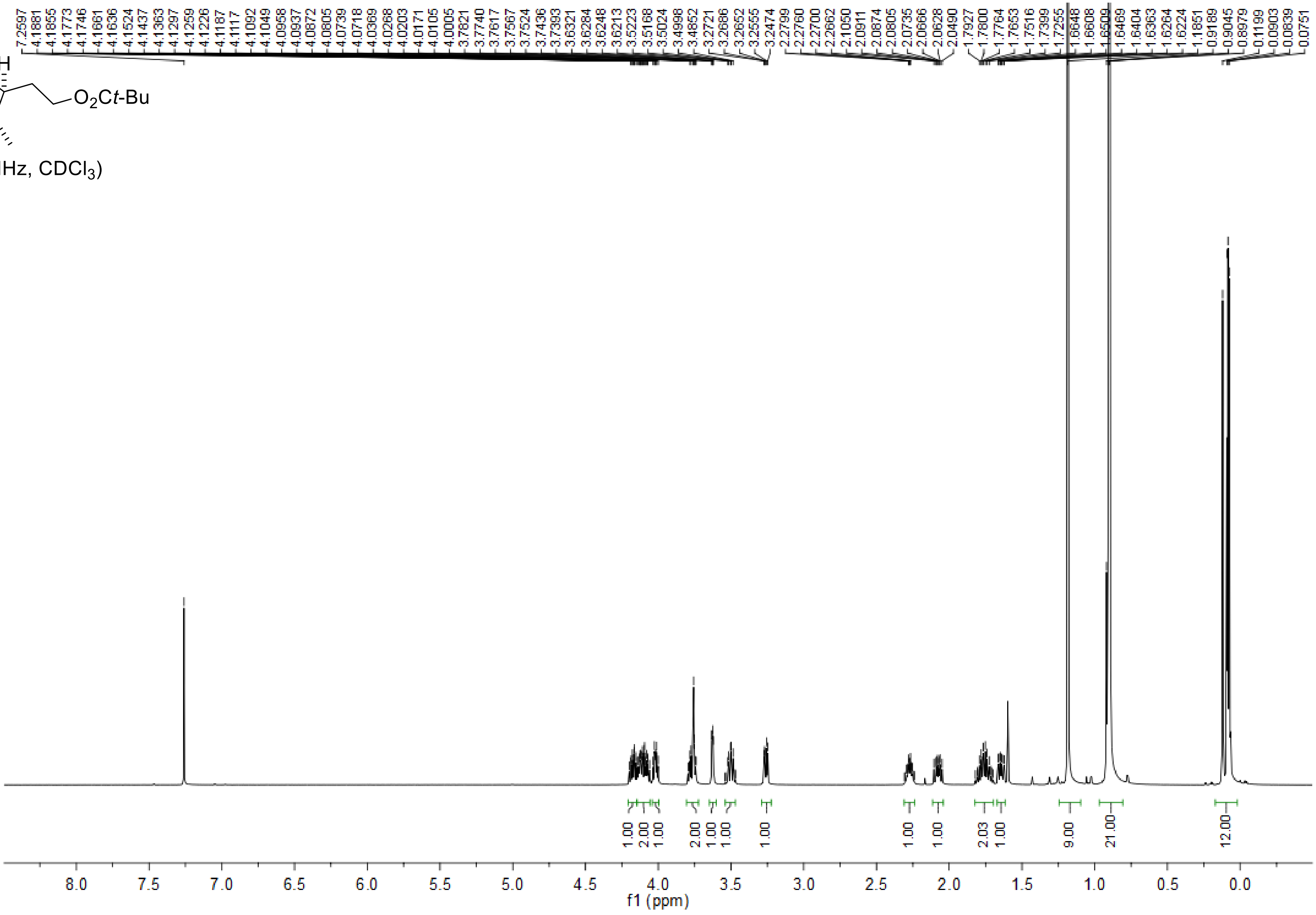

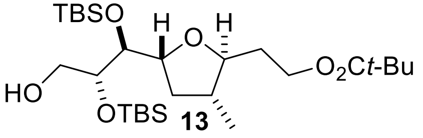

$^{13}C\{^{1}H\}$ NMR (126 MHz, $CDCl_3$)

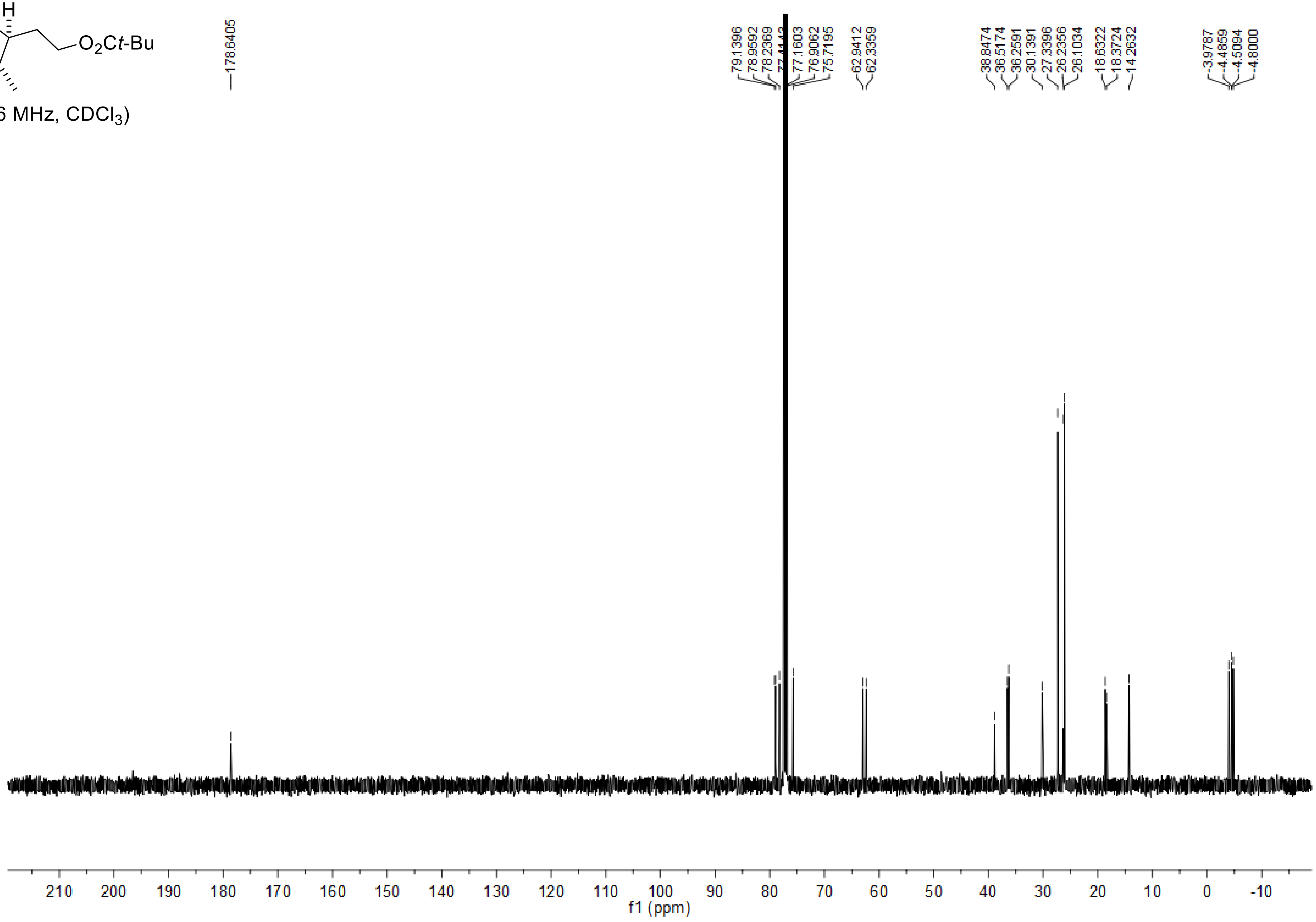

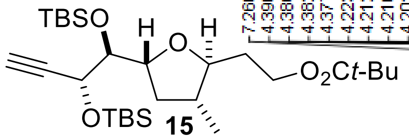

^{1}H NMR (500 MHz, $CDCl_3$)

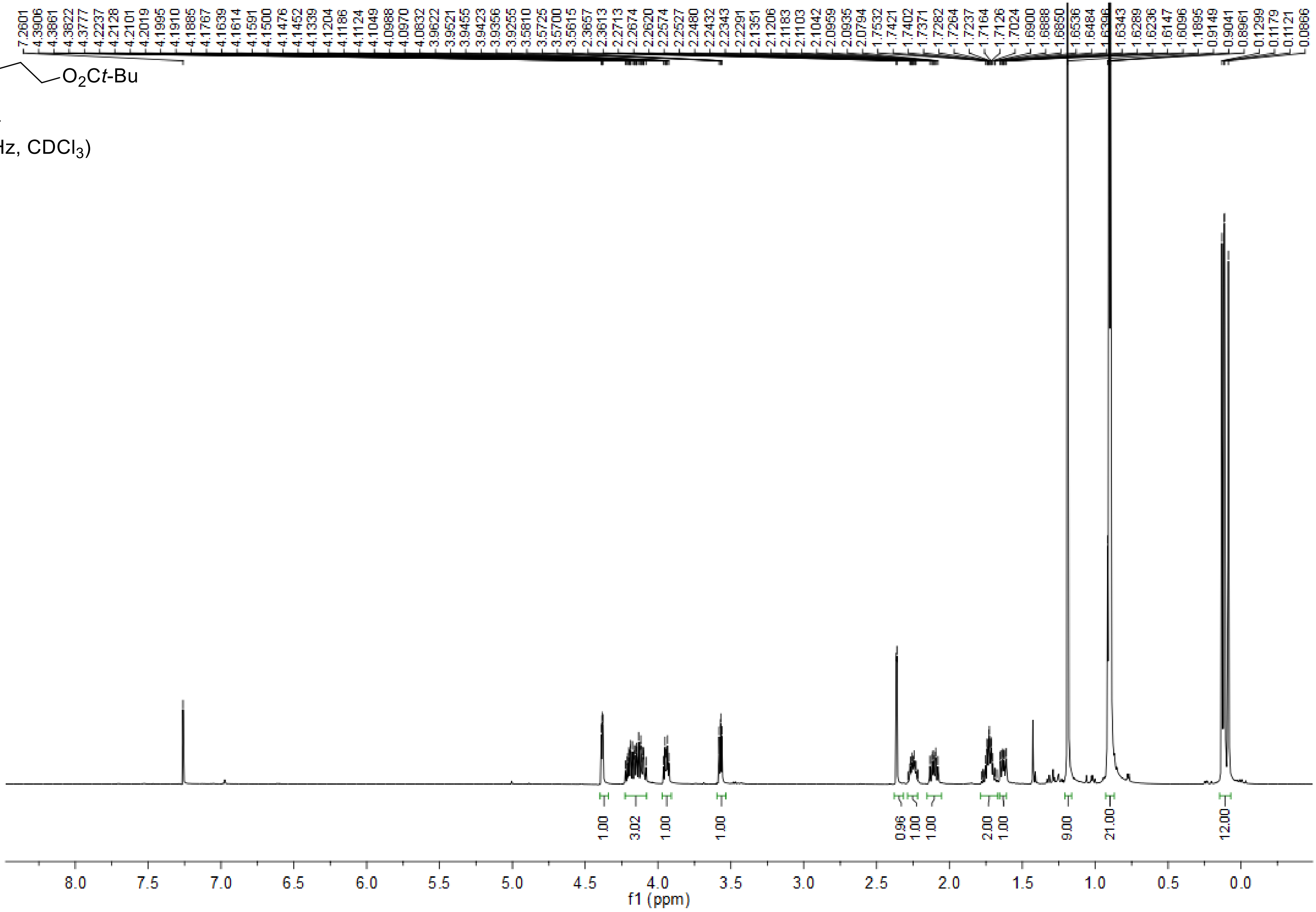

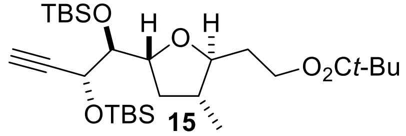

$^{13}C\{^1H\}$ NMR (126 MHz, $CDCl_3$)

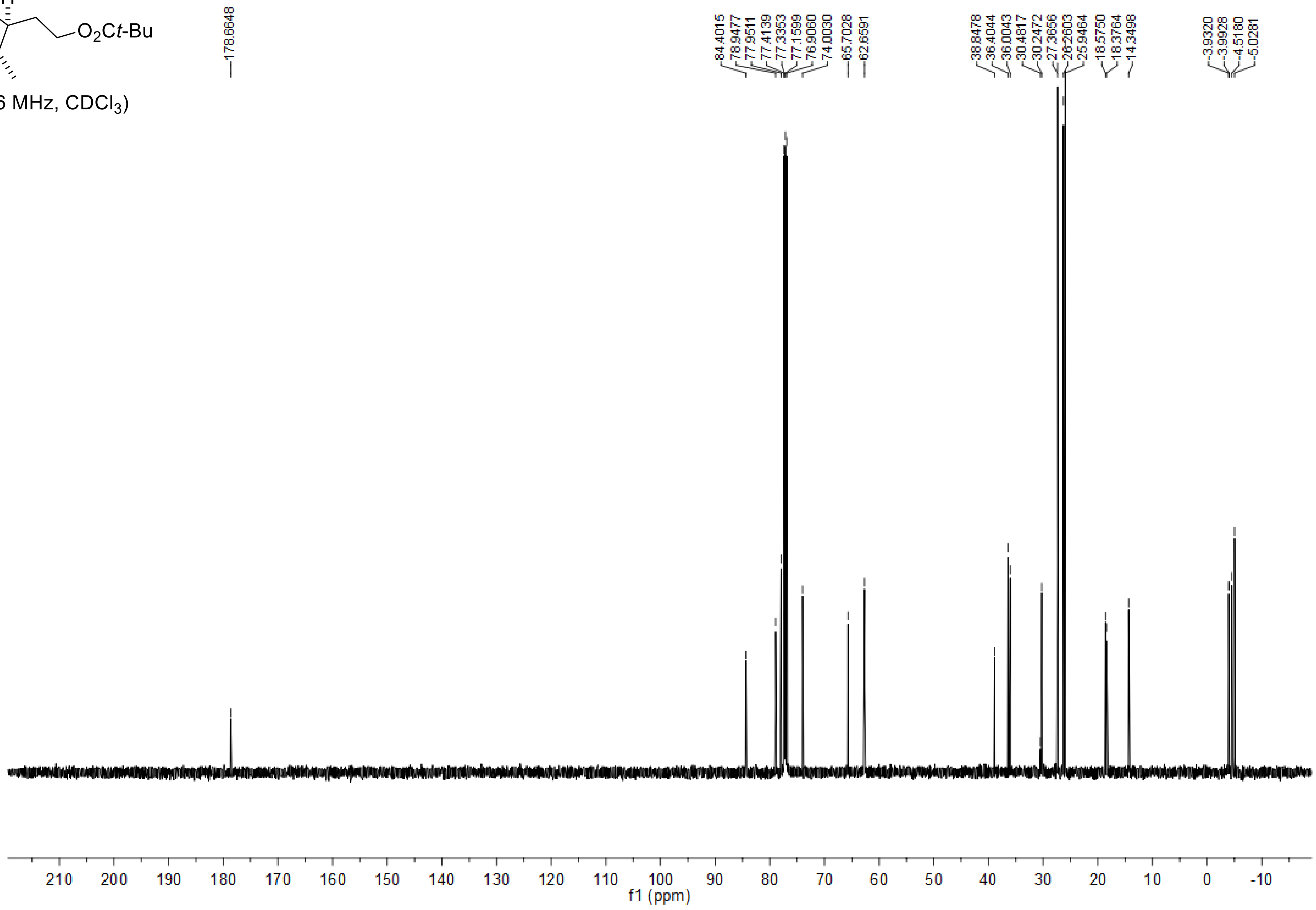

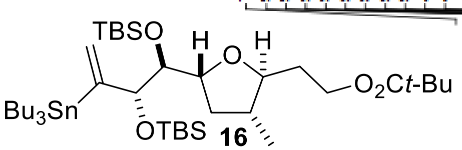

16

^{1}H NMR (500 MHz, $CDCl_3$)

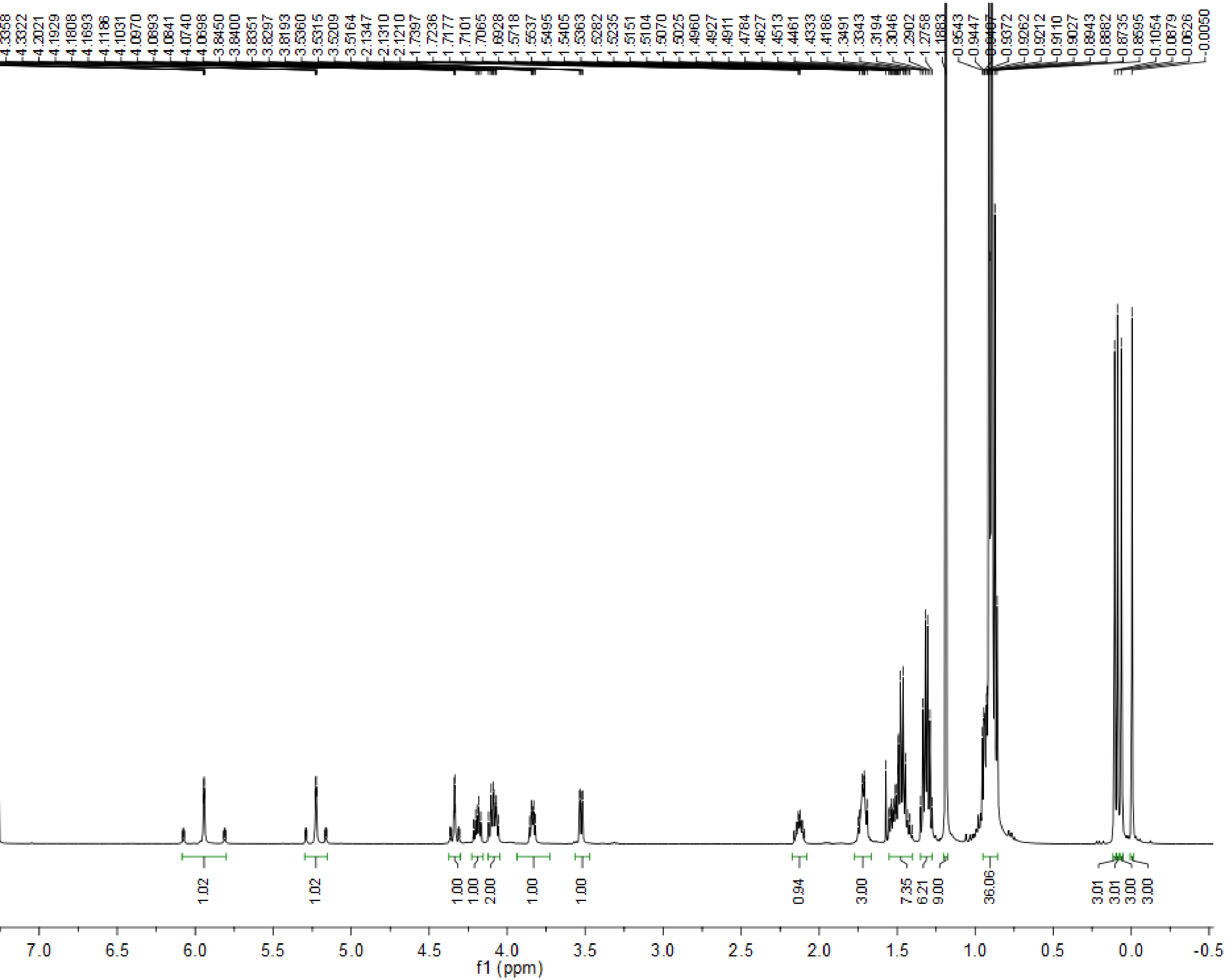

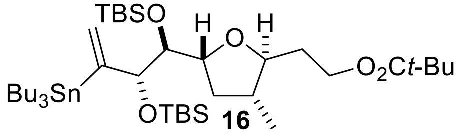

$^{13}C\{^1H\}$ NMR (126 MHz, $CDCl_3$)

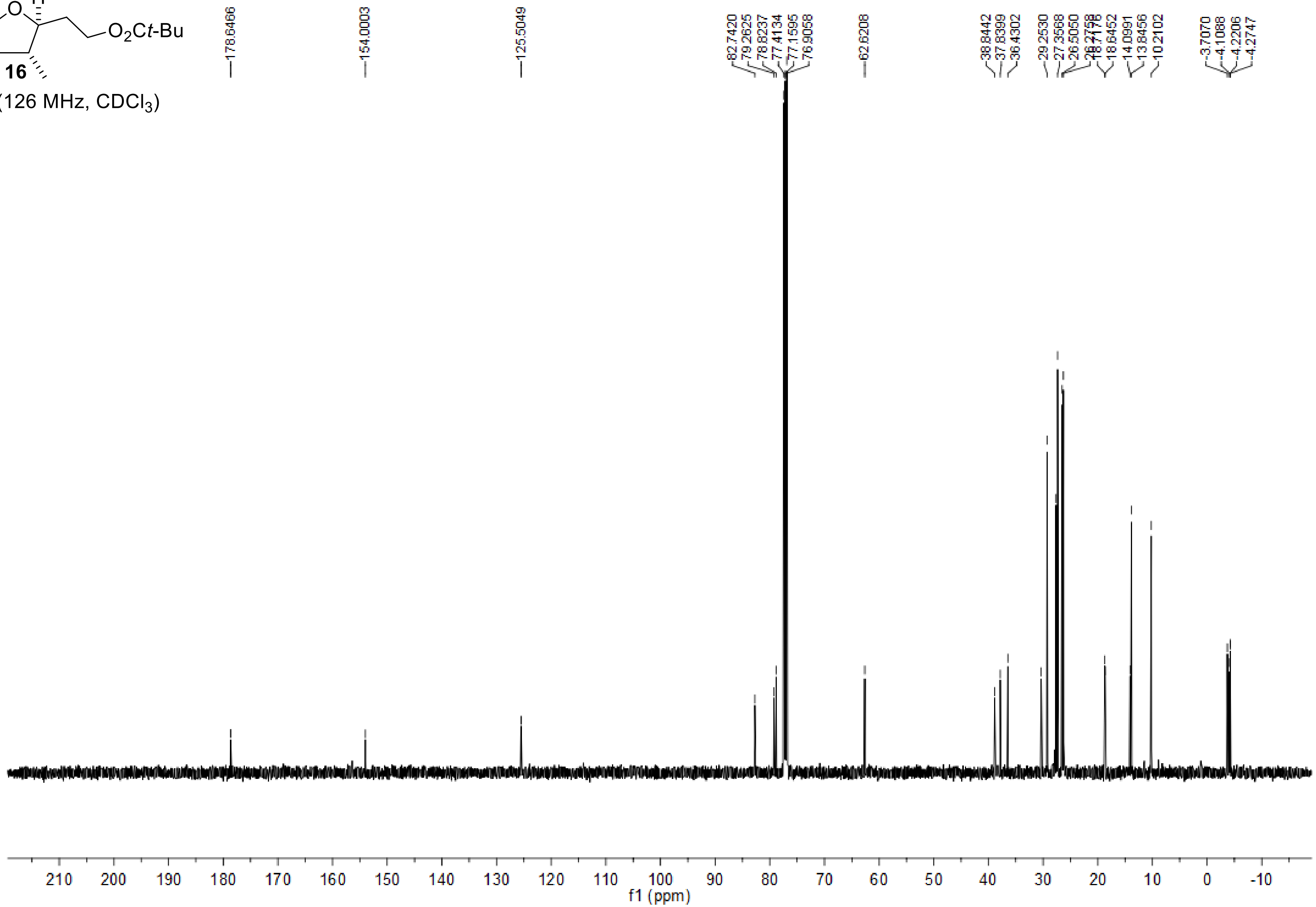

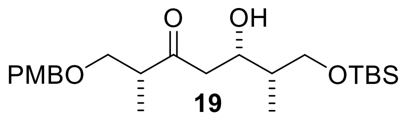

19

^{1}H NMR (500 MHz, $CDCl_3$)

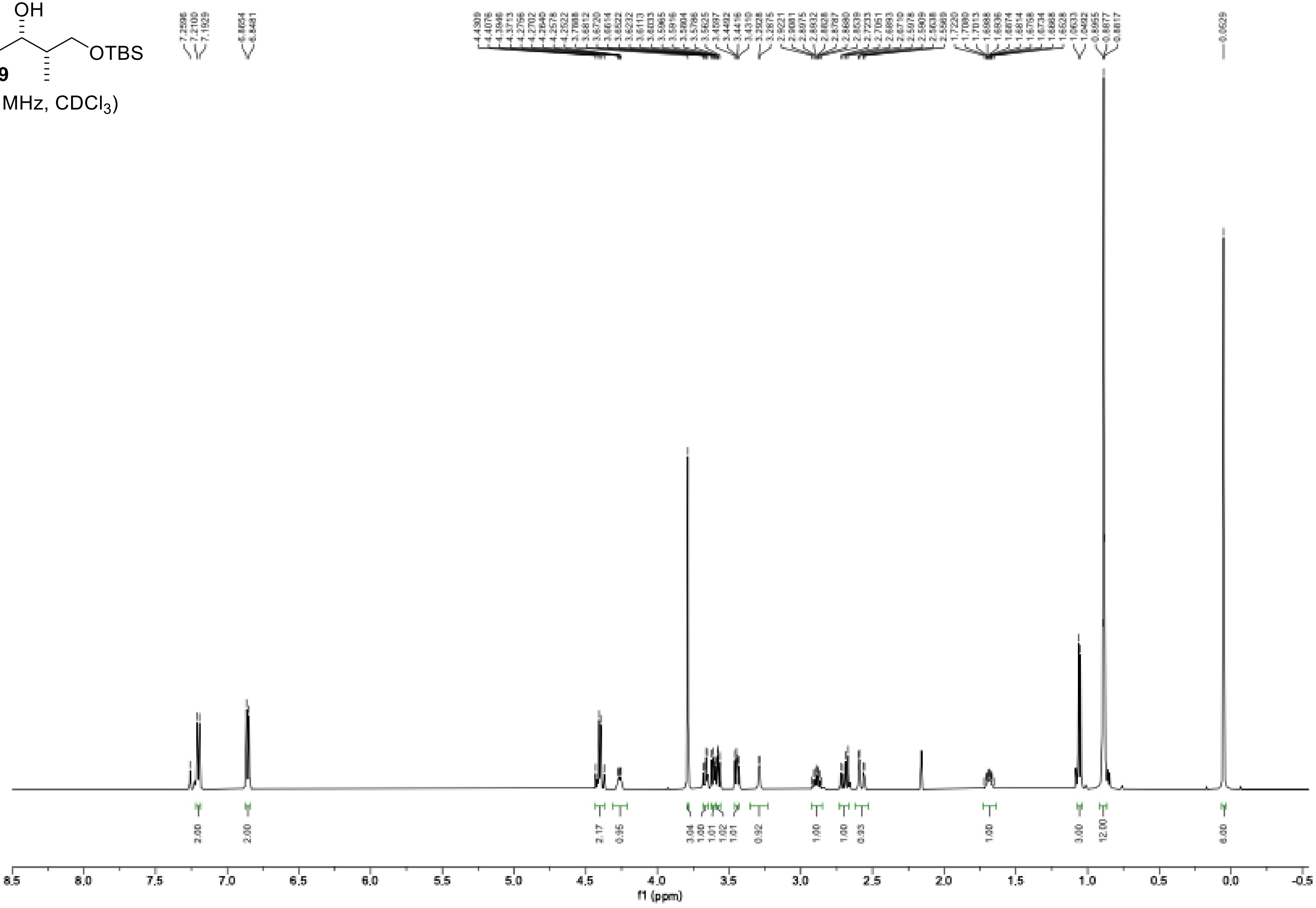

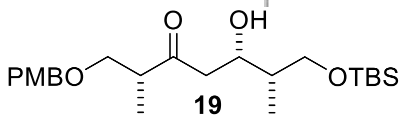

19

$^{13}C\{^{1}H\}$ NMR (126 MHz, $CDCl_3$)

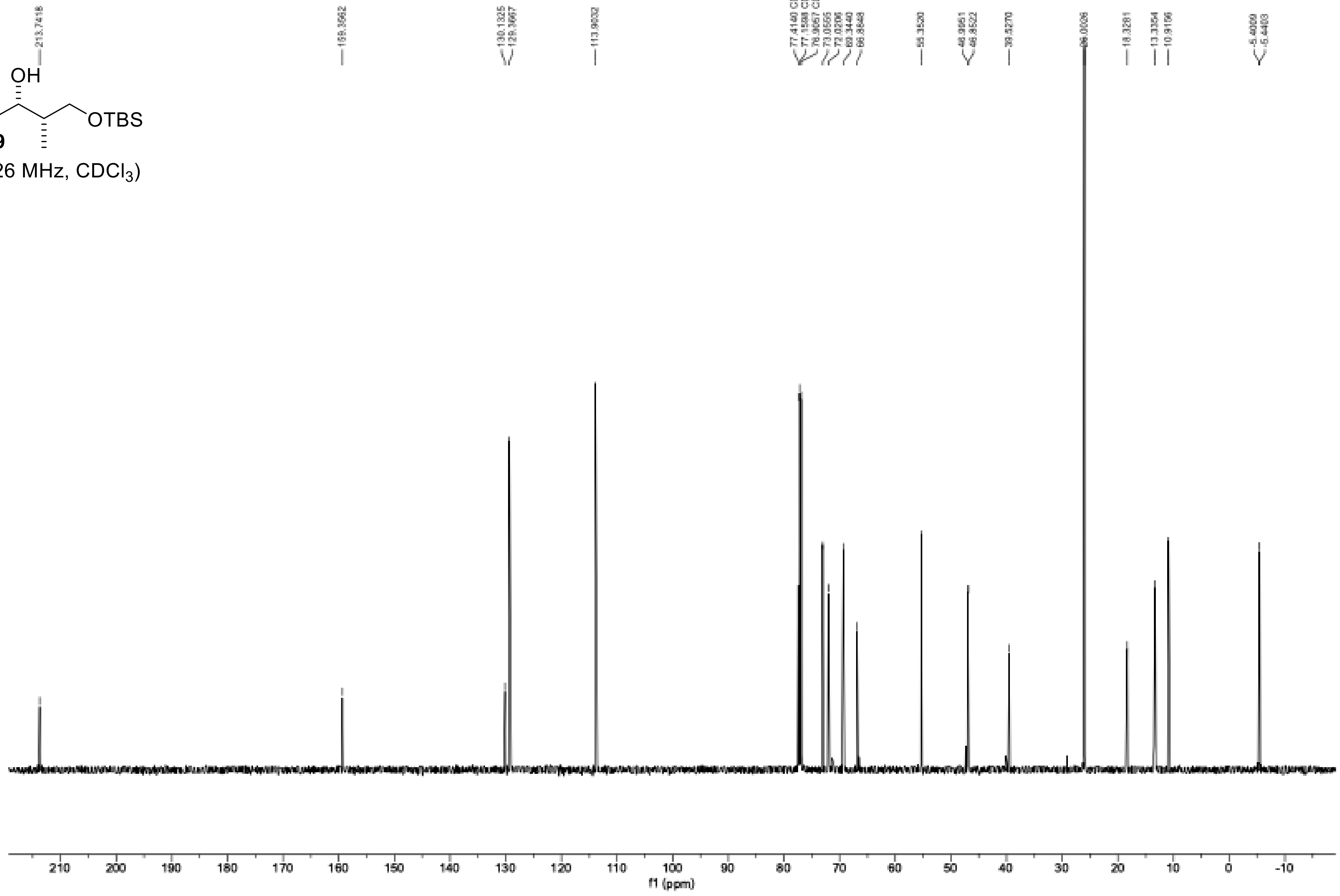

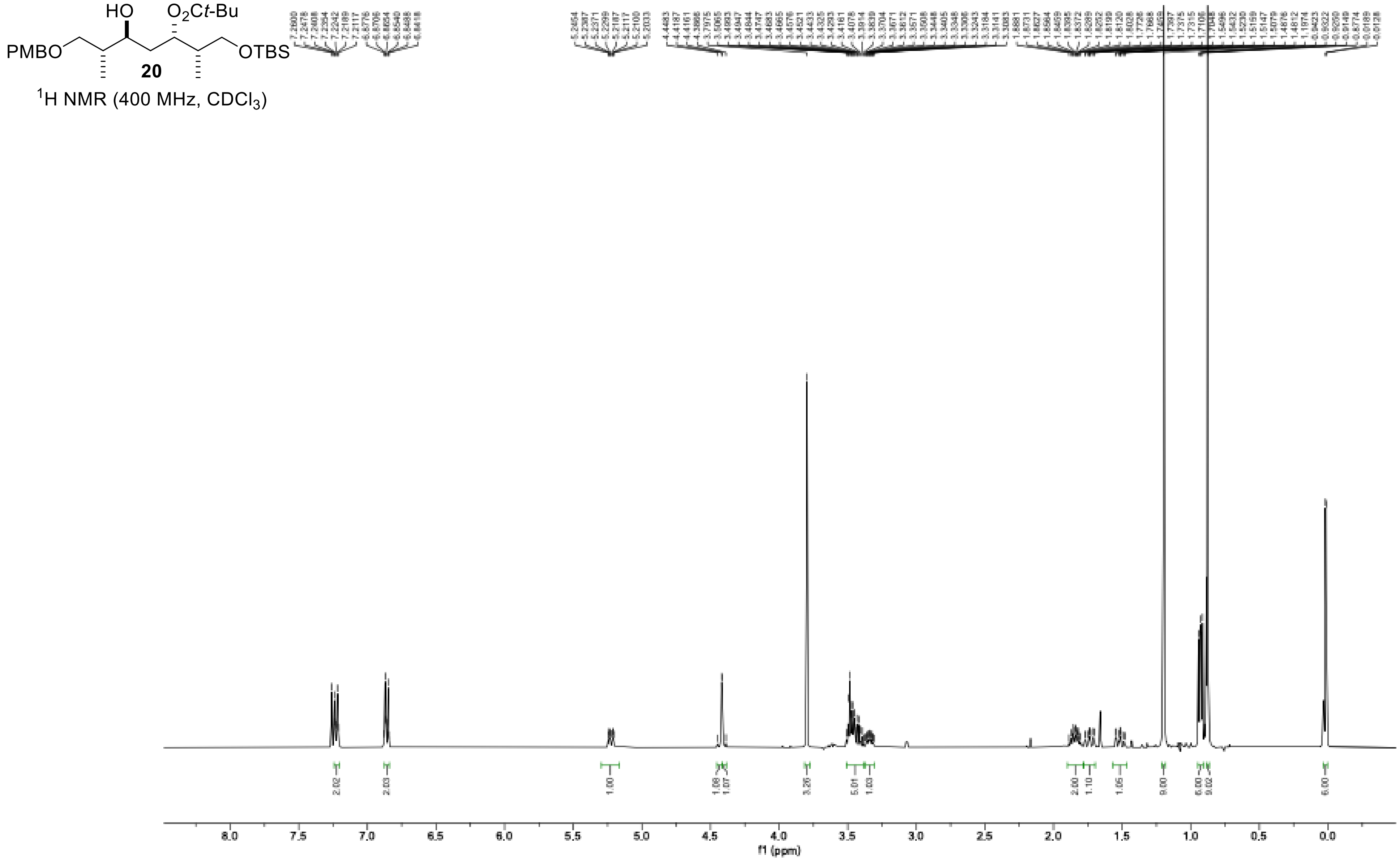
PMBO
HO
O_2Ct-Bu
OTBS
20
^{1}H NMR (400 MHz, $CDCl_3$)
f1 (ppm)

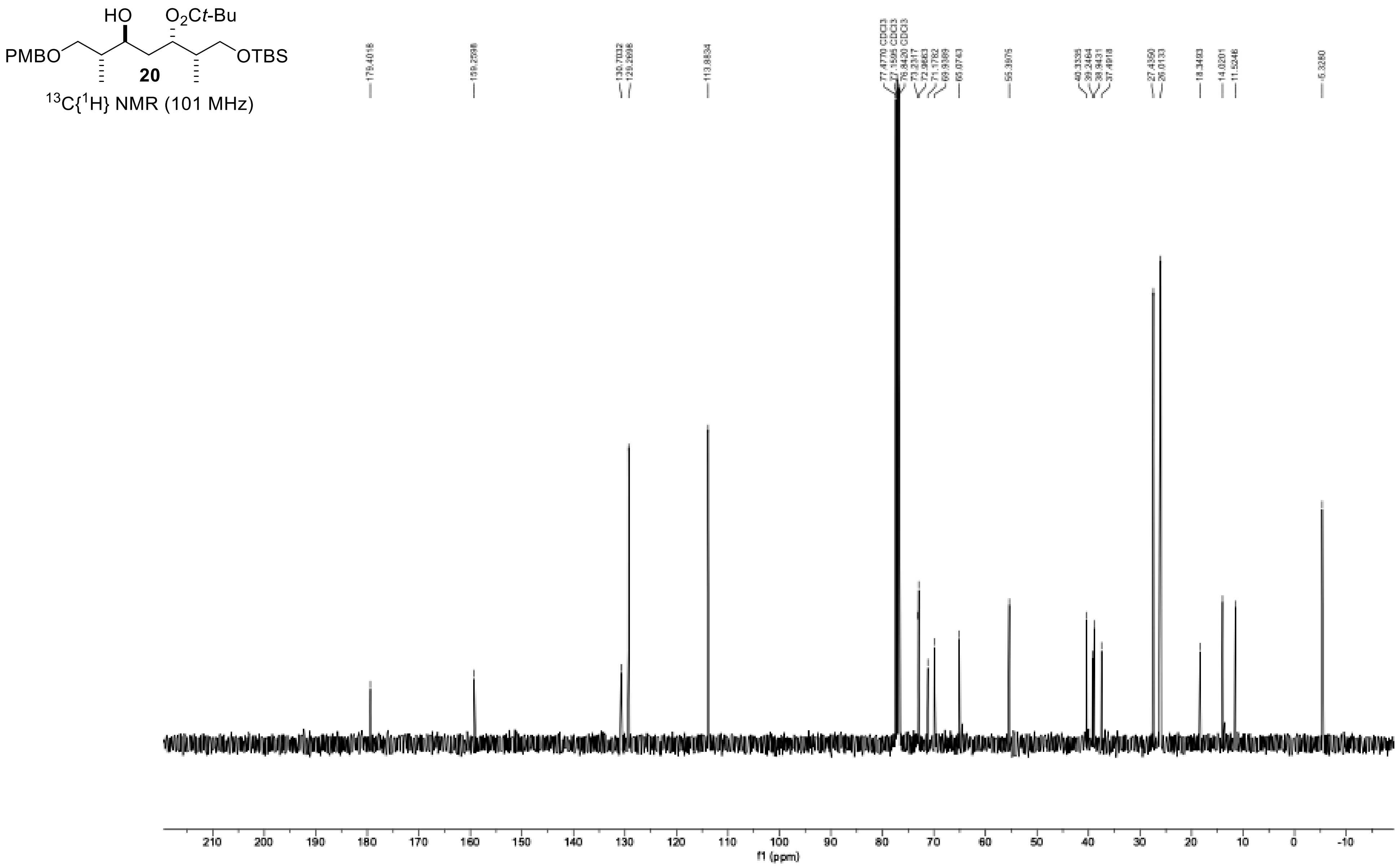
HO
O2Ct-Bu
PMBO
OTBS
20
13C{1H} NMR (101 MHz)
f1 (ppm)

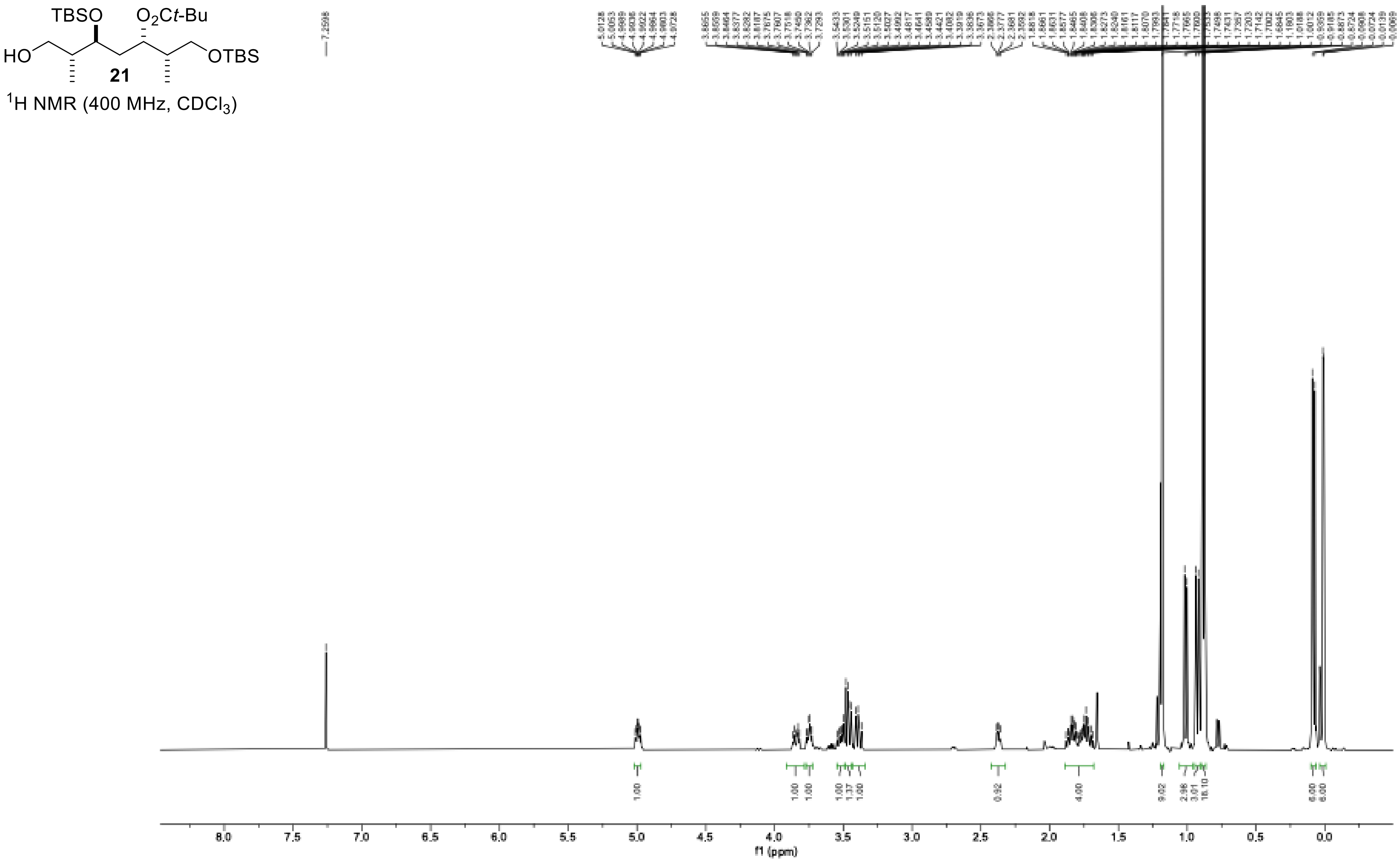
TBSO
O2Ct-Bu
HO
OTBS
21
1H NMR (400 MHz, CDCl3)
f1 (ppm)

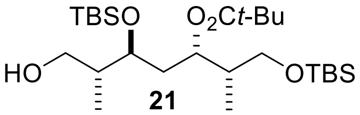

21

$^{13}C\{^1H\}$ NMR (101 MHz, $CDCl_3$)

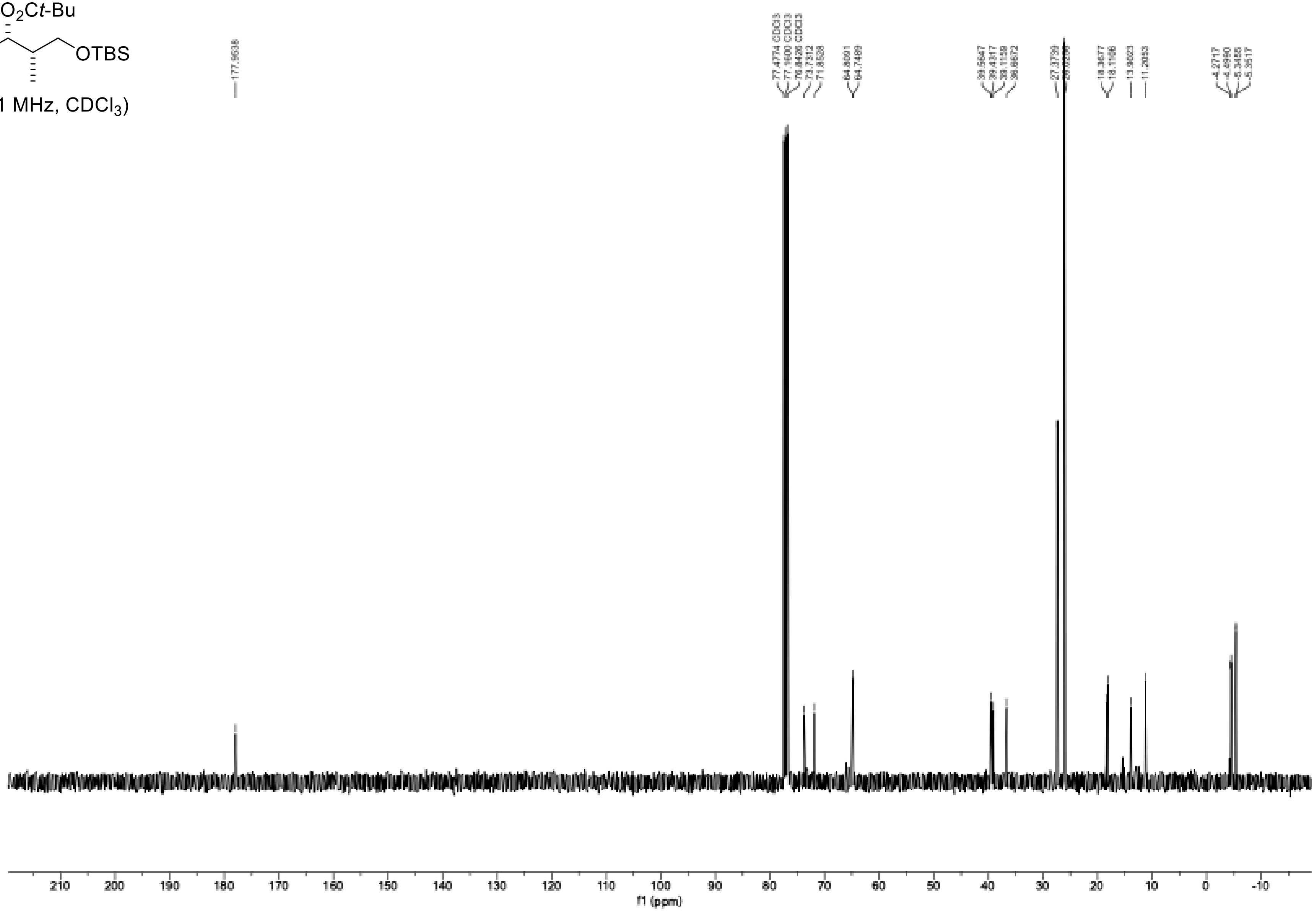

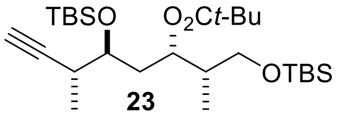

^{1}H NMR (400 MHz, $CDCl_3$)

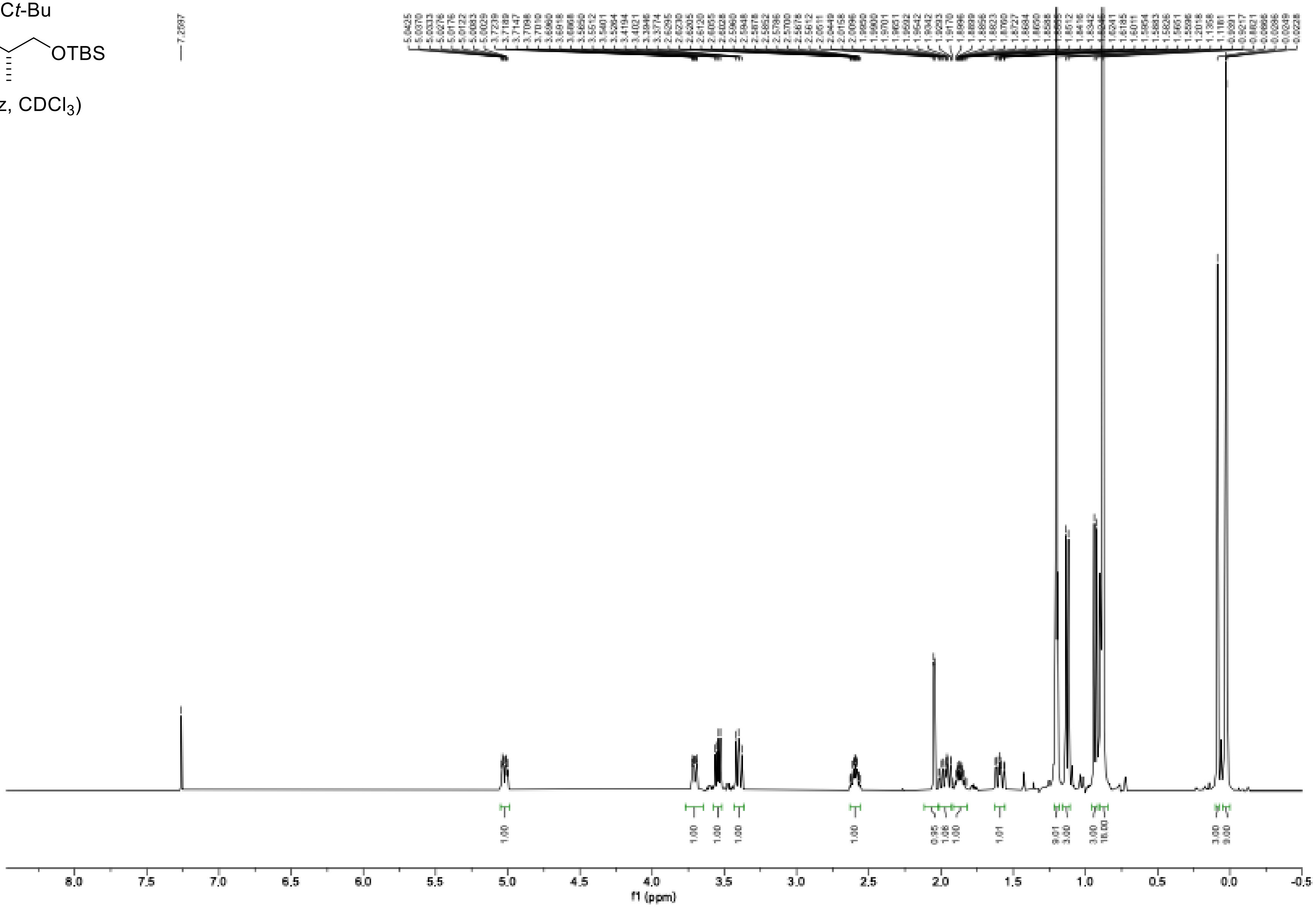

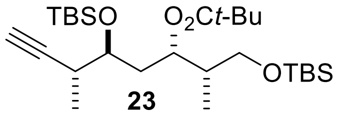

23

$^{13}C\{^1H\}$ NMR (101 MHz, $CDCl_3$)

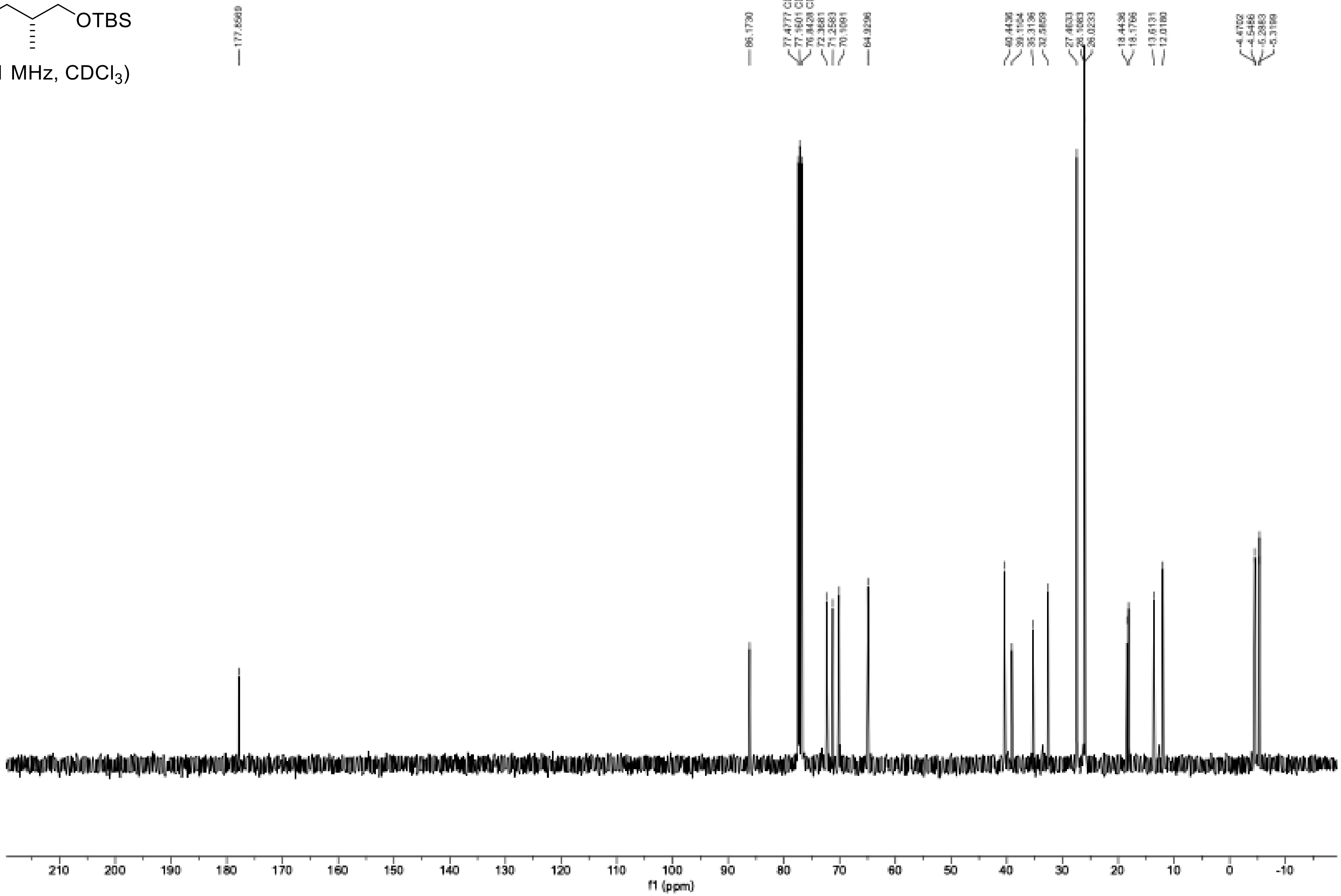

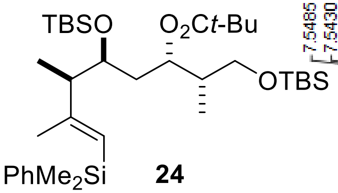

24

^{1}H NMR (400 MHz, $CDCl_3$)

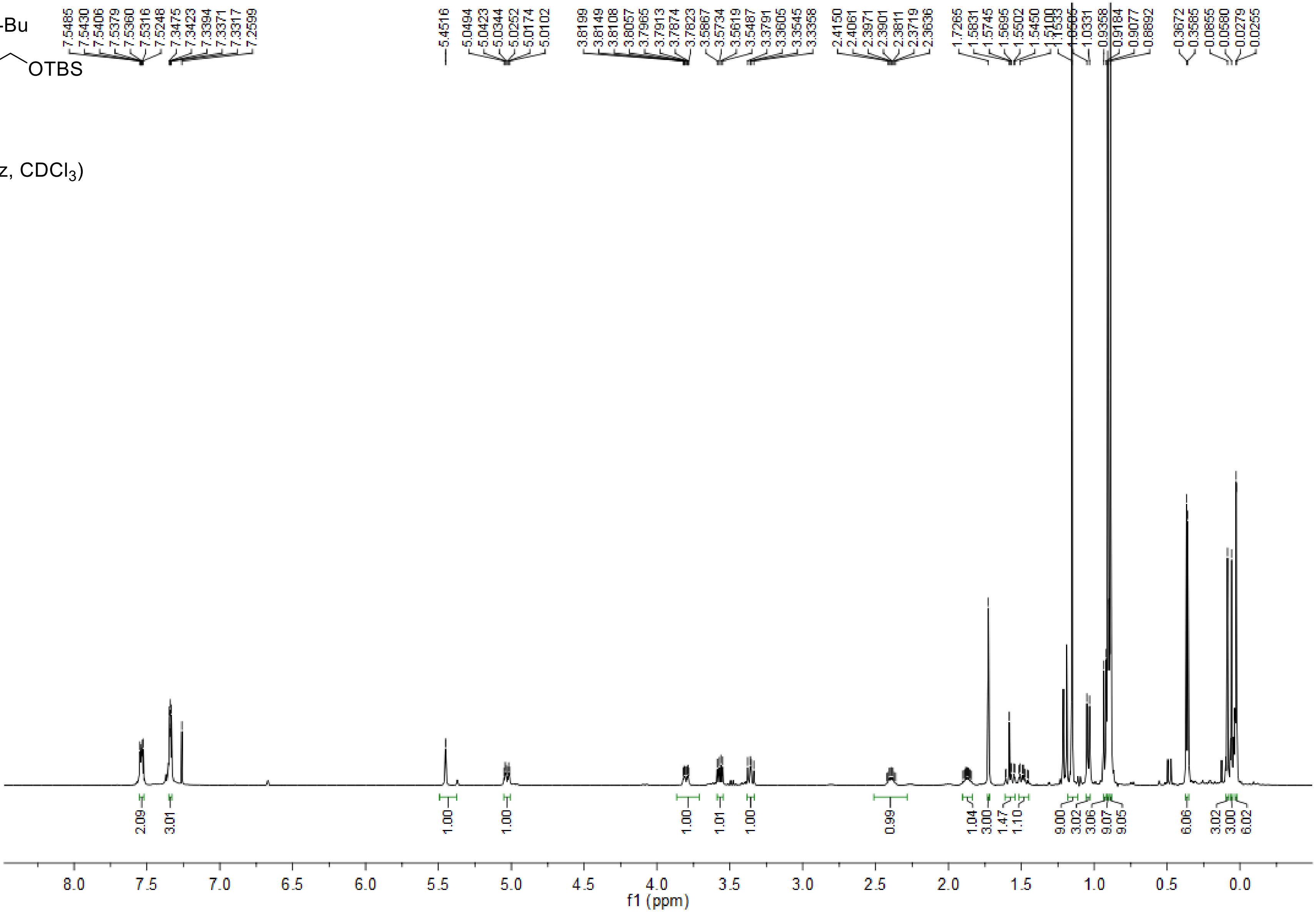

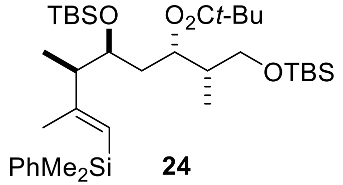

24

$^{13}C\{^{1}H\}$ NMR (101 MHz, $CDCl_3$)

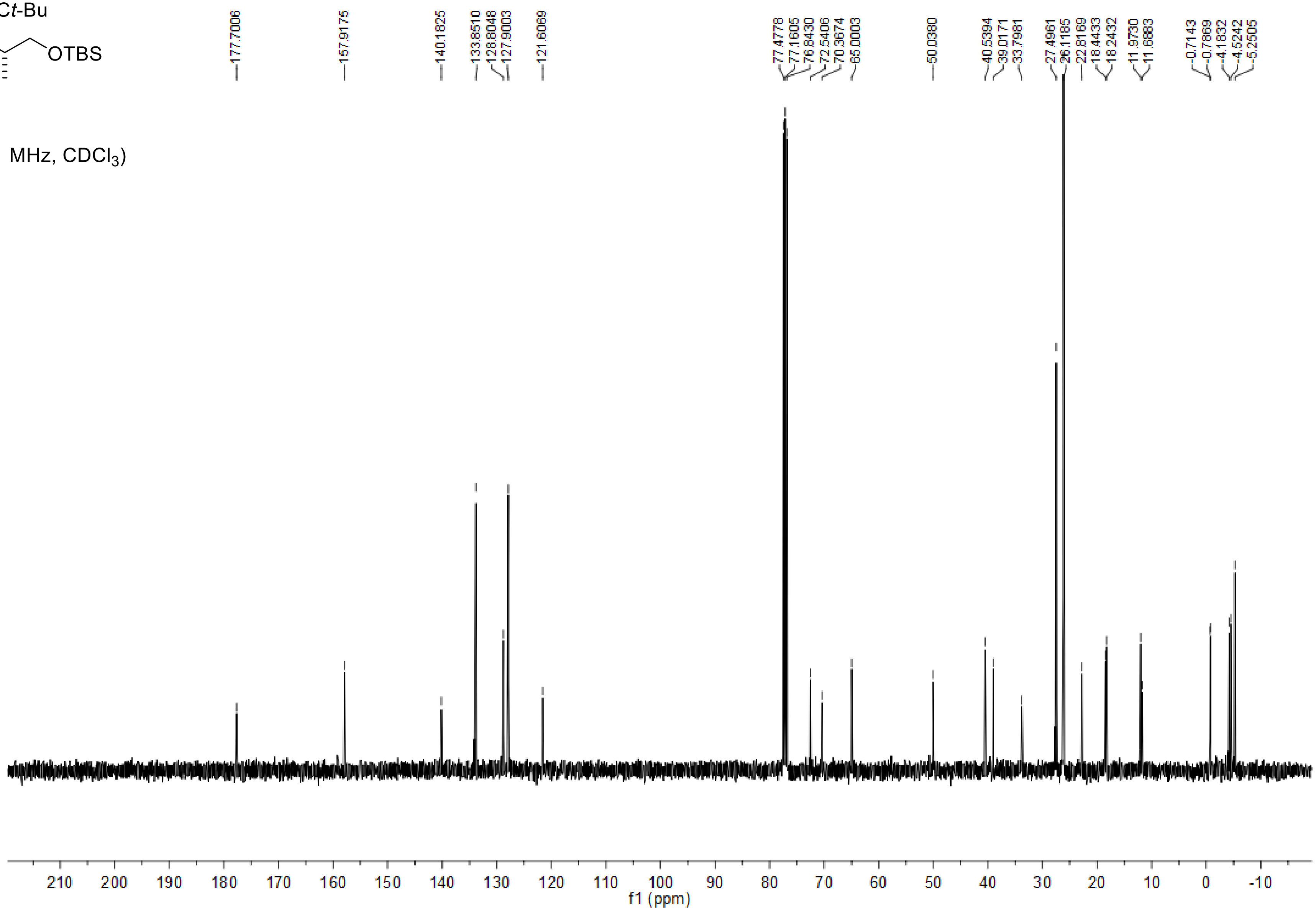

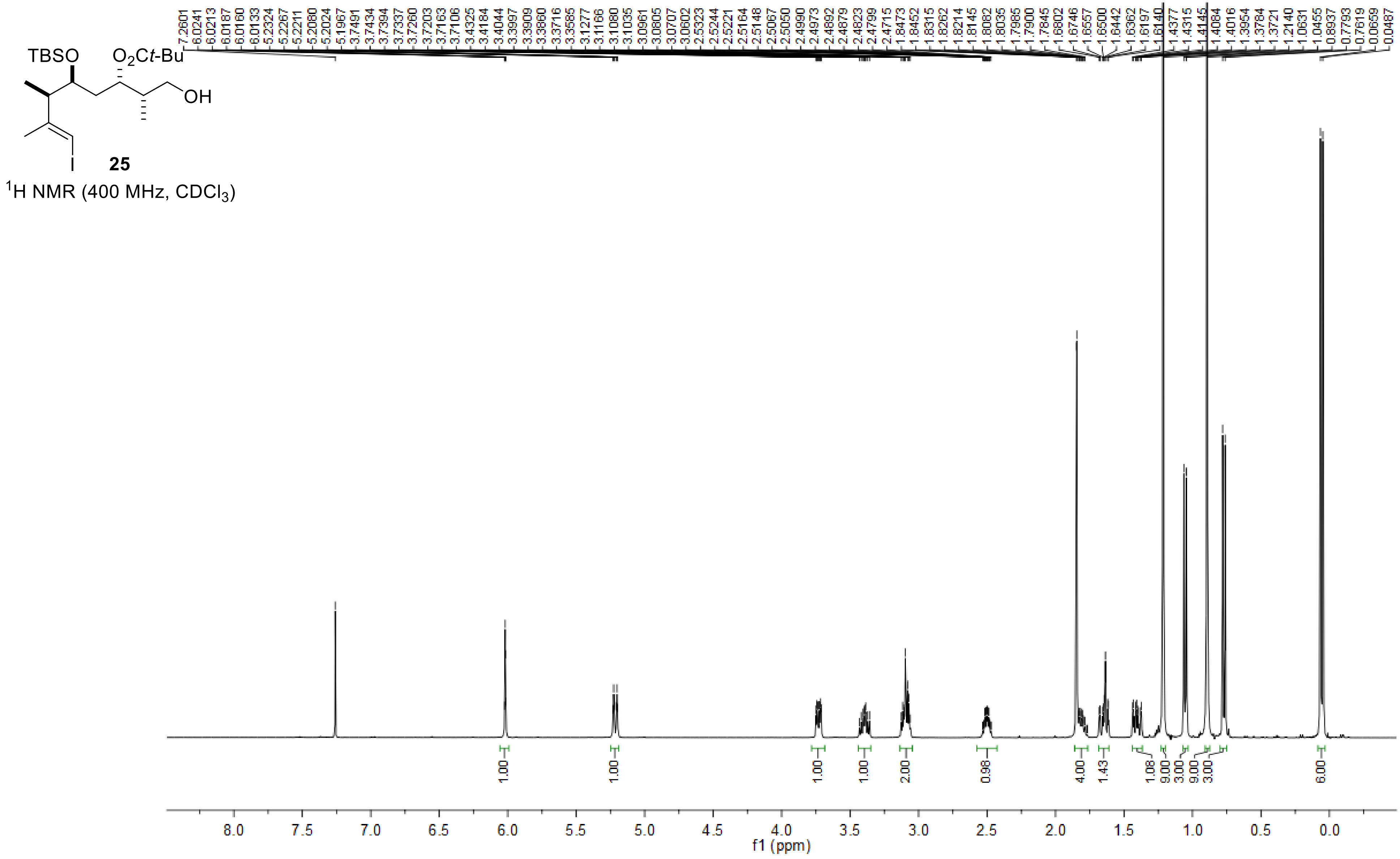

^{1}H NMR (400 MHz, $CDCl_3$)

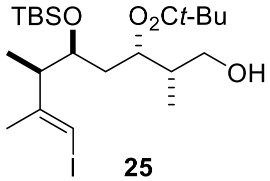

25

$^{13}C\{^{1}H\}$ NMR (101 MHz, $CDCl_3$)

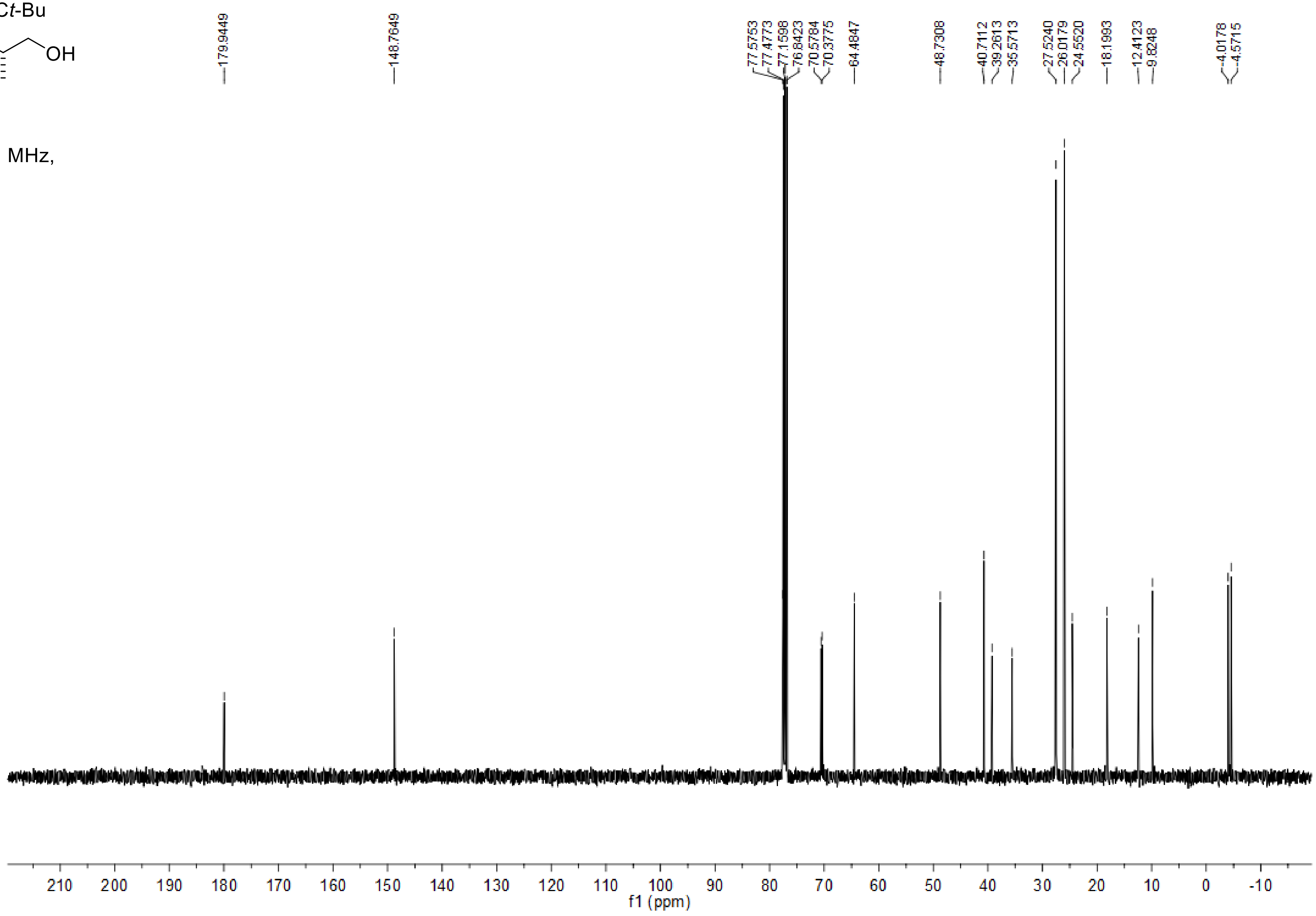

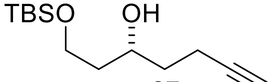

27

^{1}H NMR (400 MHz, $CDCl_3$)

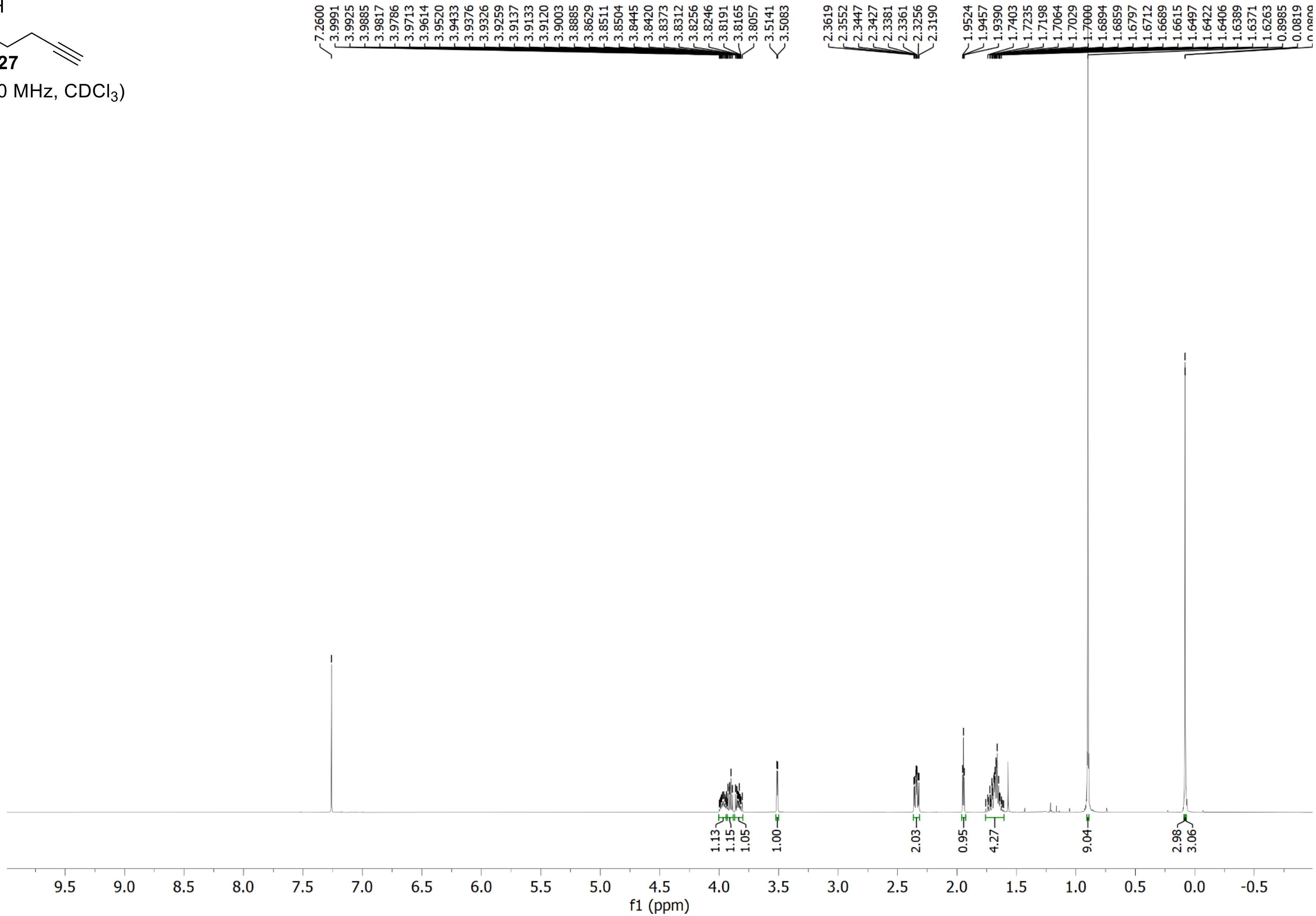

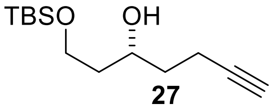

27

$^{13}C\{^{1}H\}$ NMR (101 MHz, $CDCl_3$)

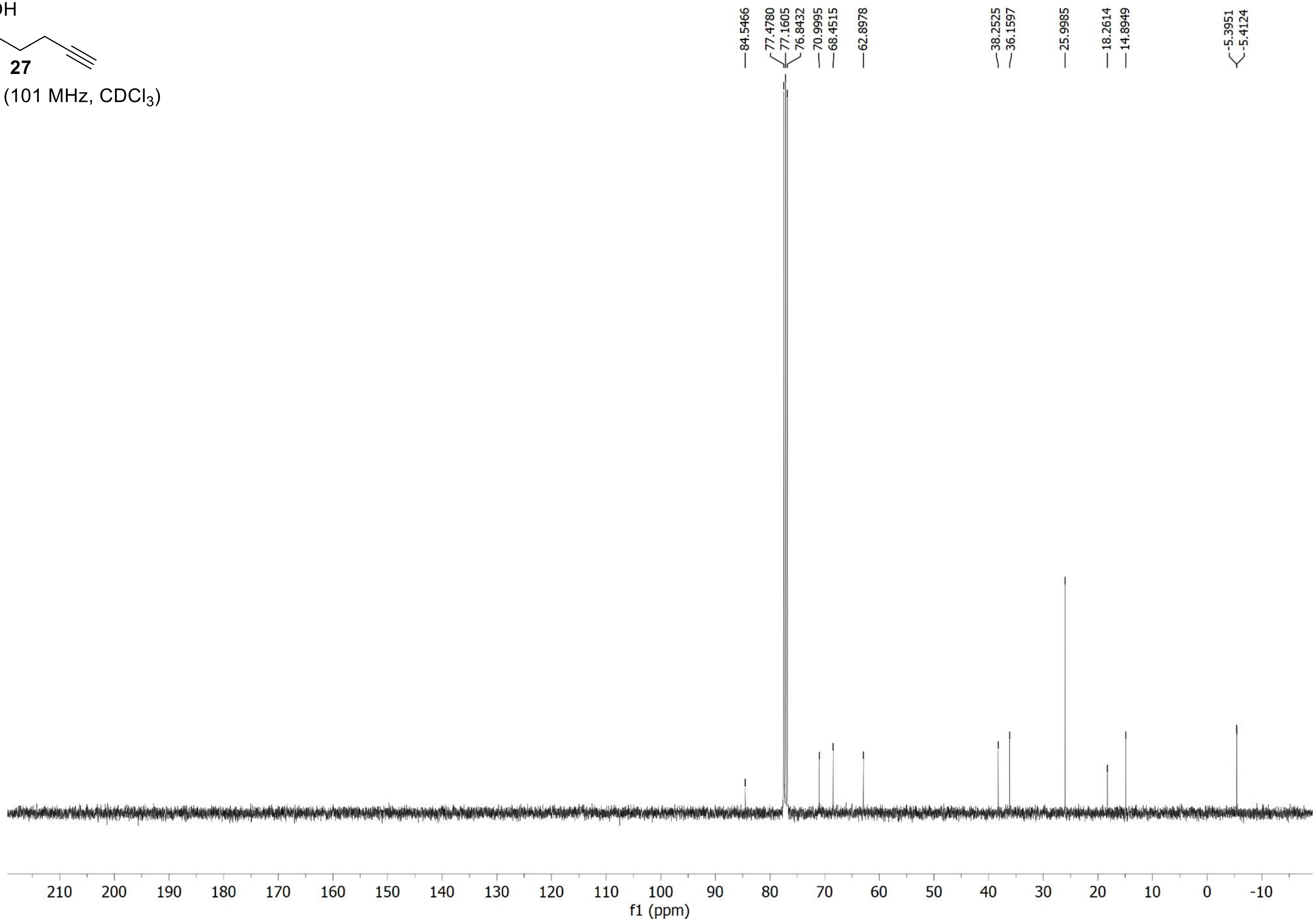

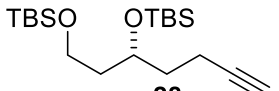

28

^{1}H NMR (500 MHz, $CDCl_3$)

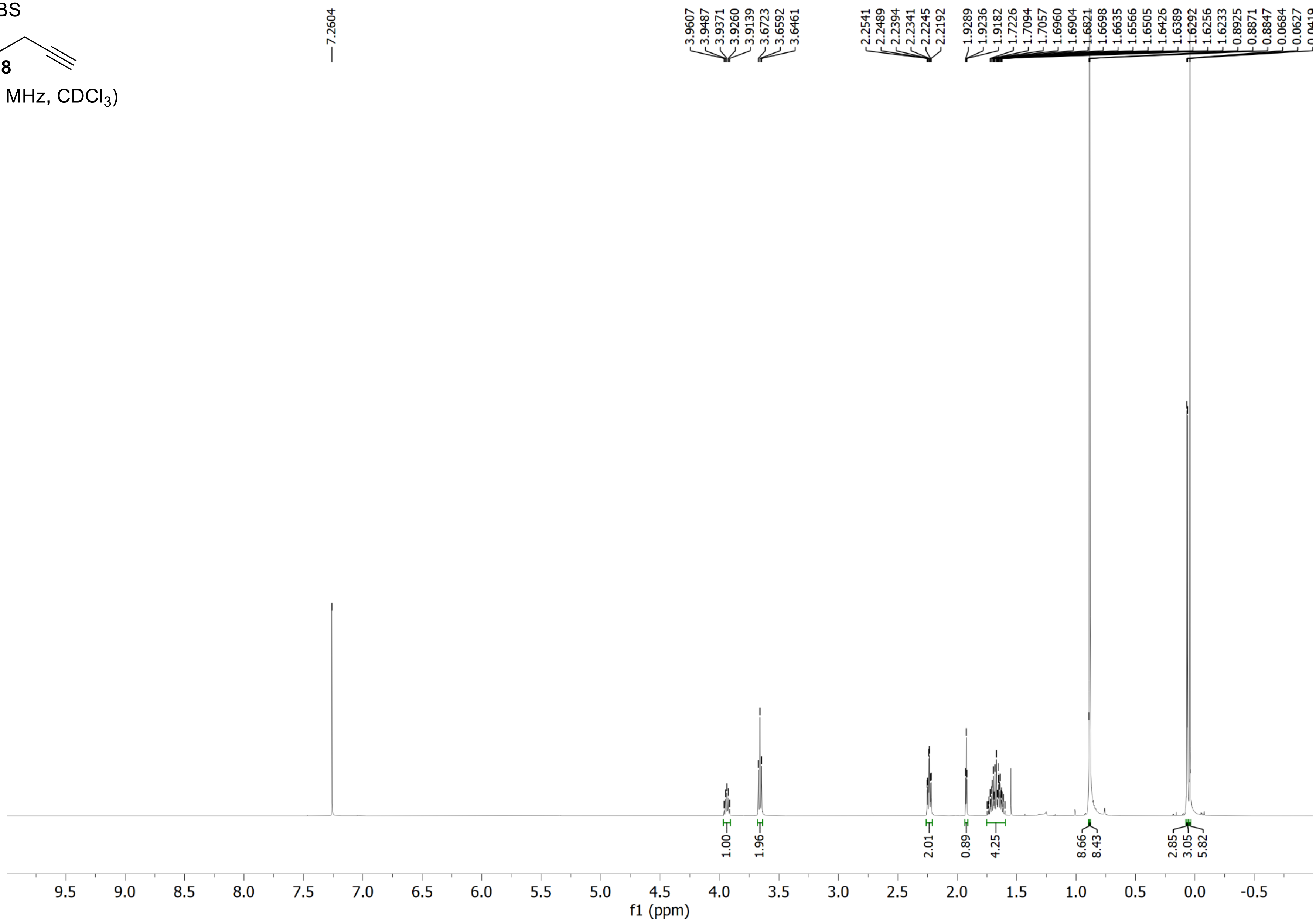

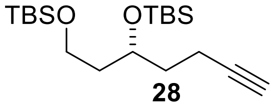

28

$^{13}C\{^{1}H\}$ NMR (126 MHz, $CDCl_3$)

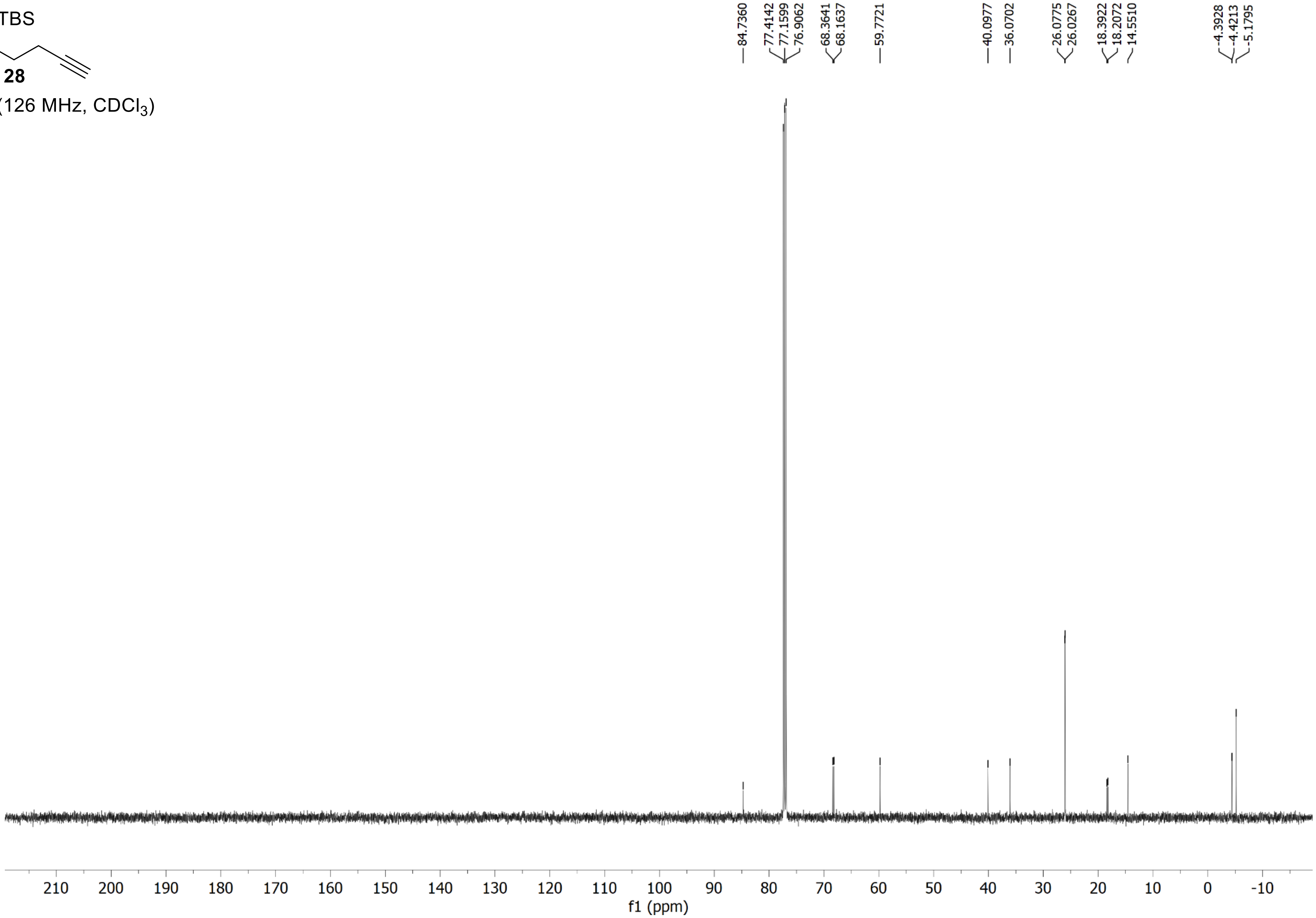

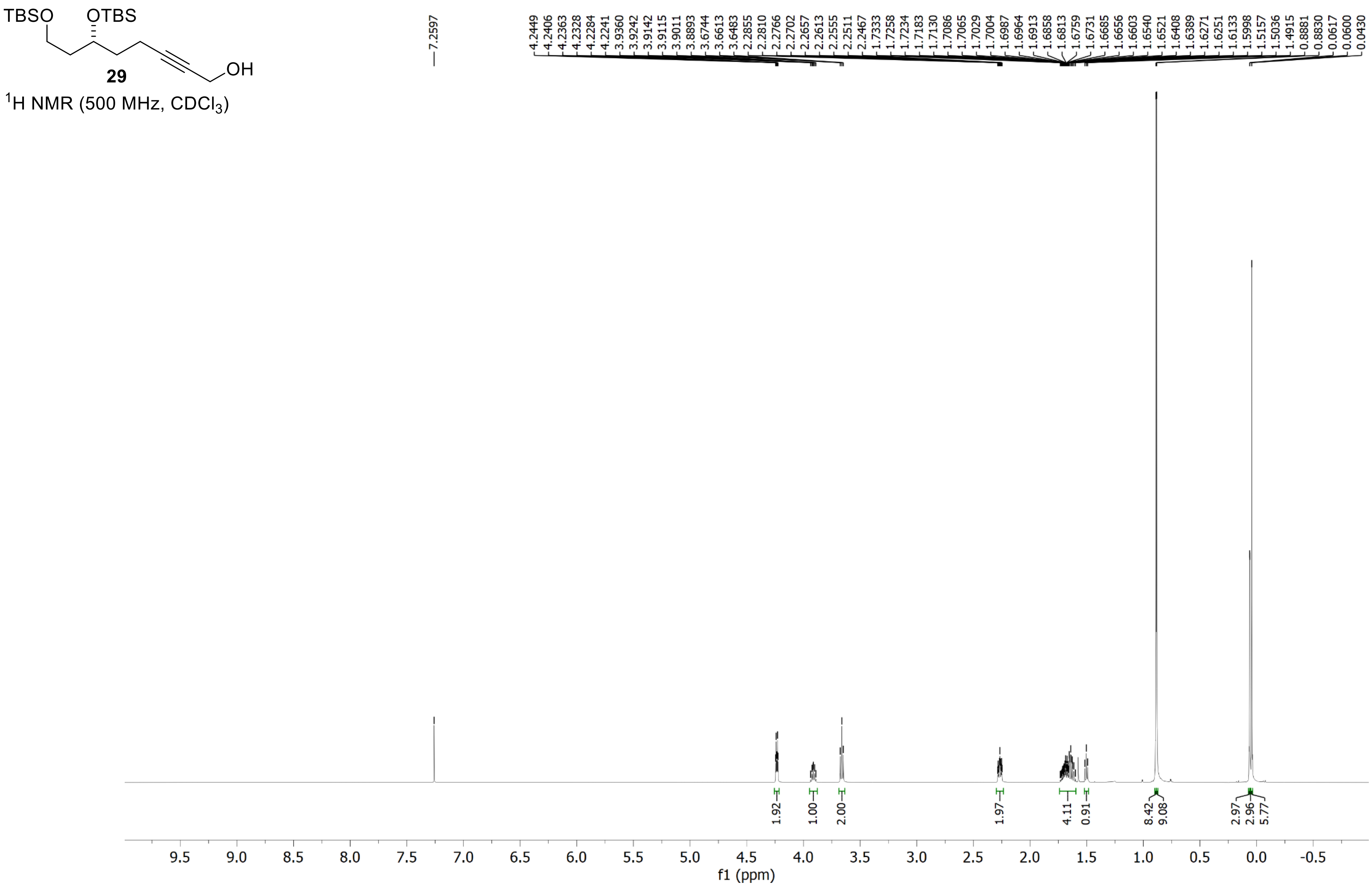

^1H NMR (500 MHz, $CDCl_3$)

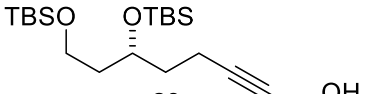

29

$^{13}C\{^{1}H\}$ NMR (126 MHz, $CDCl_3$)

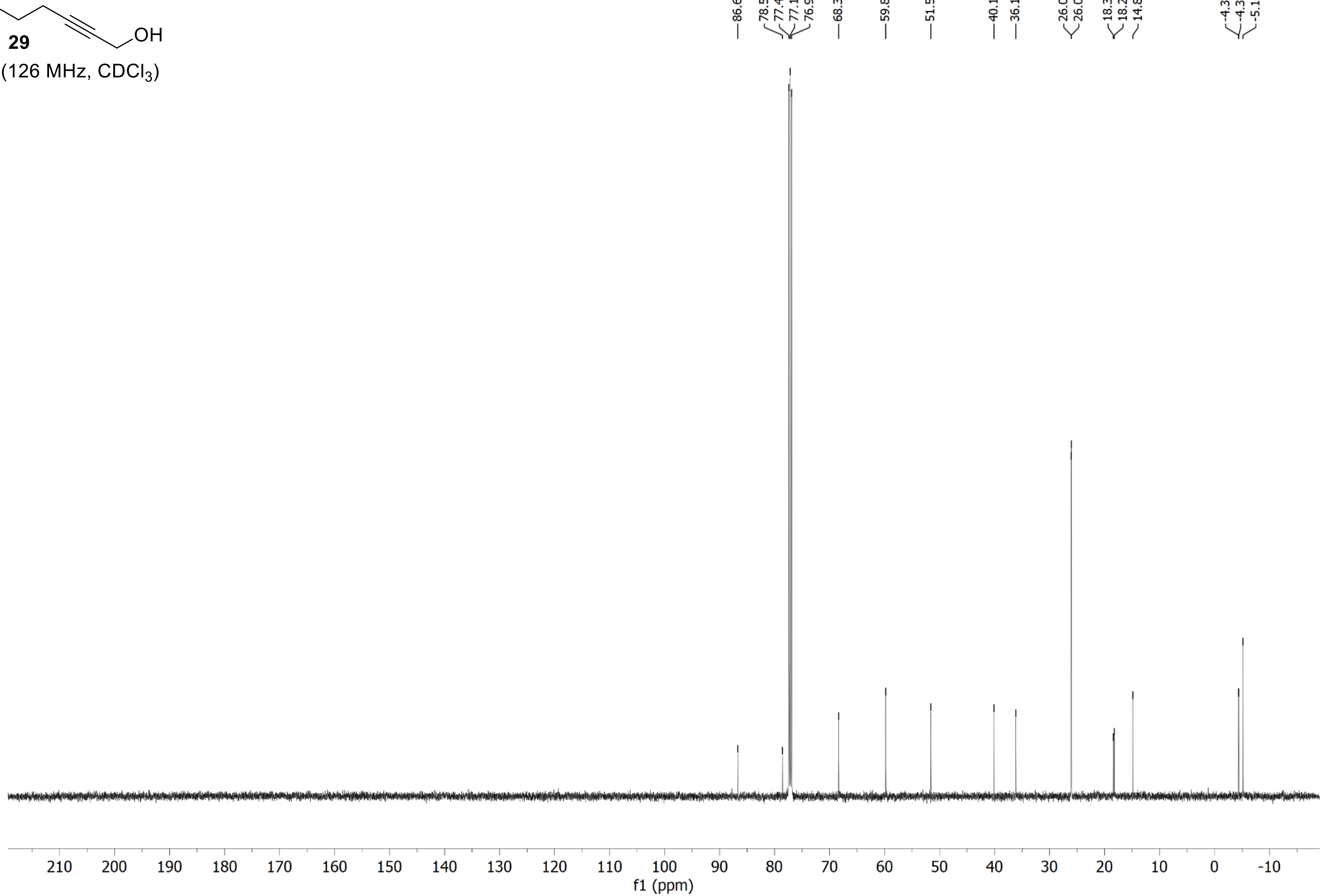

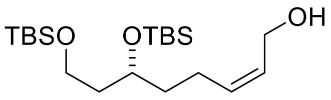

30

^{1}H NMR (400 MHz, $CDCl_3$)

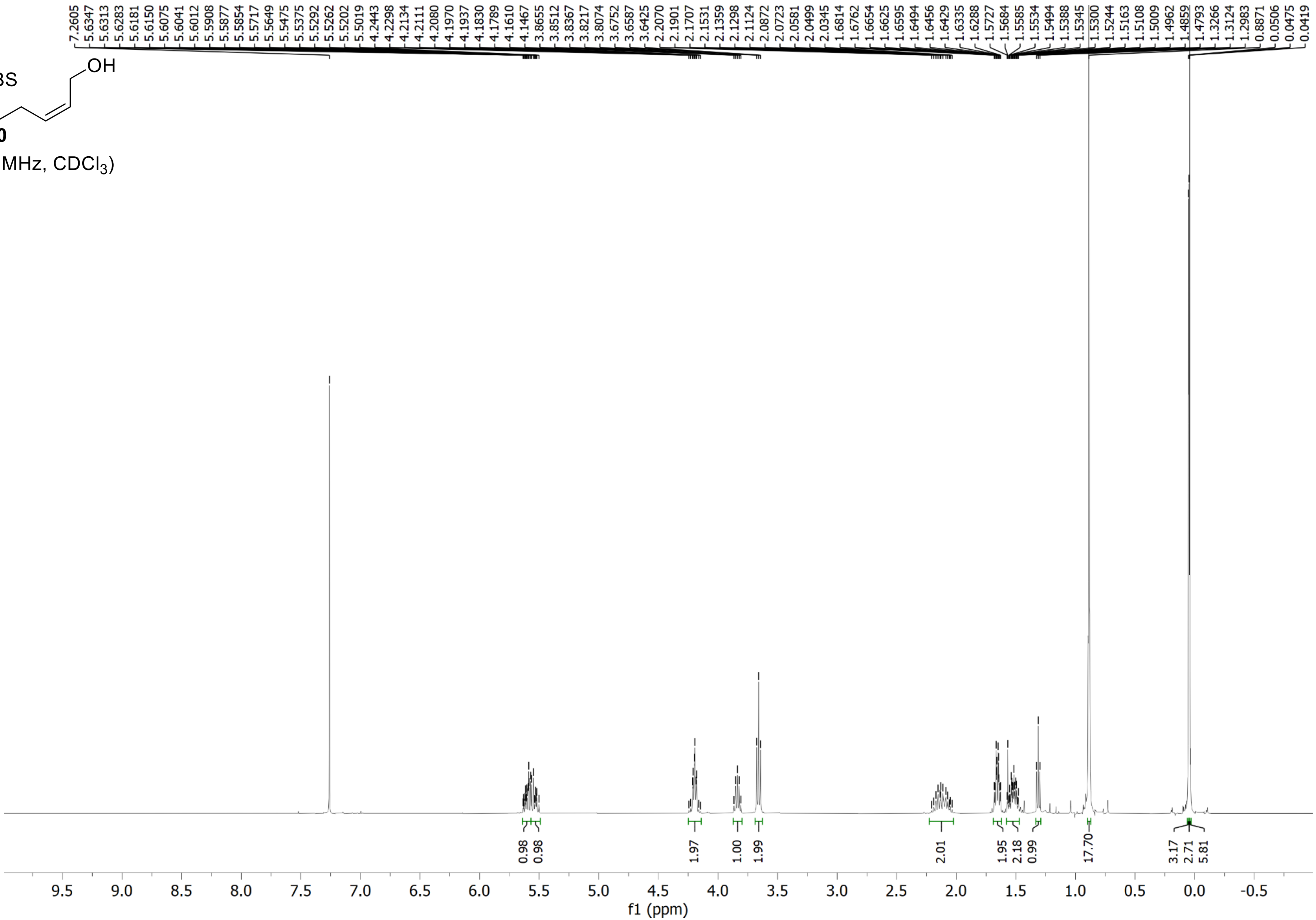

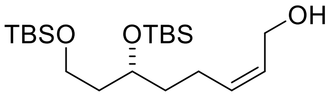

30

$^{13}C\{^1H\}$ NMR (126 MHz, $CDCl_3$)

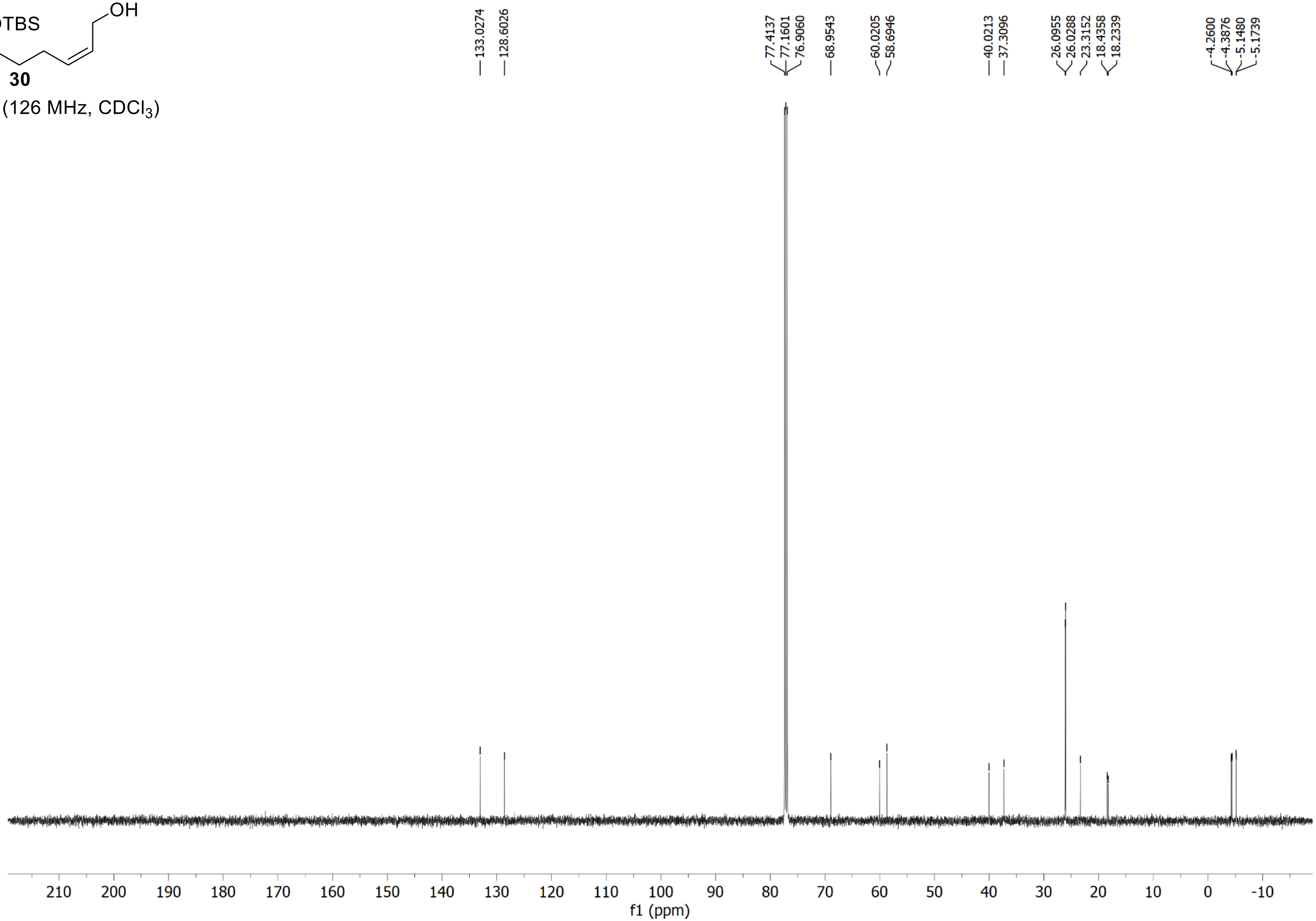

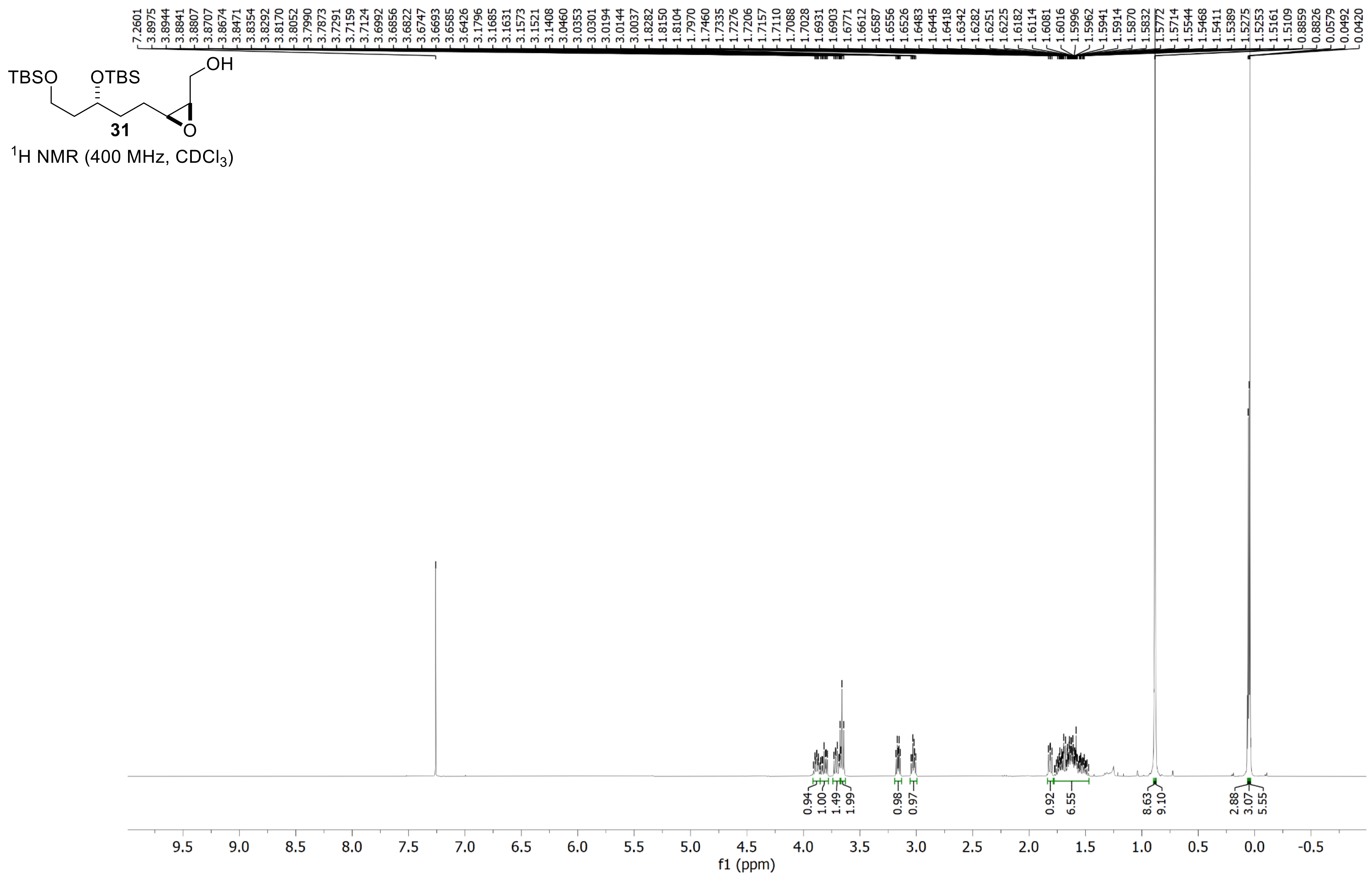
TBSO
OTBS
OH
O
31
^{1}H NMR (400 MHz, CDCl3)
f1 (ppm)

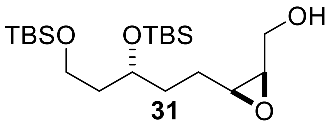

31

$^{13}C\{^1H\}$ NMR (126 MHz, $CDCl_3$)

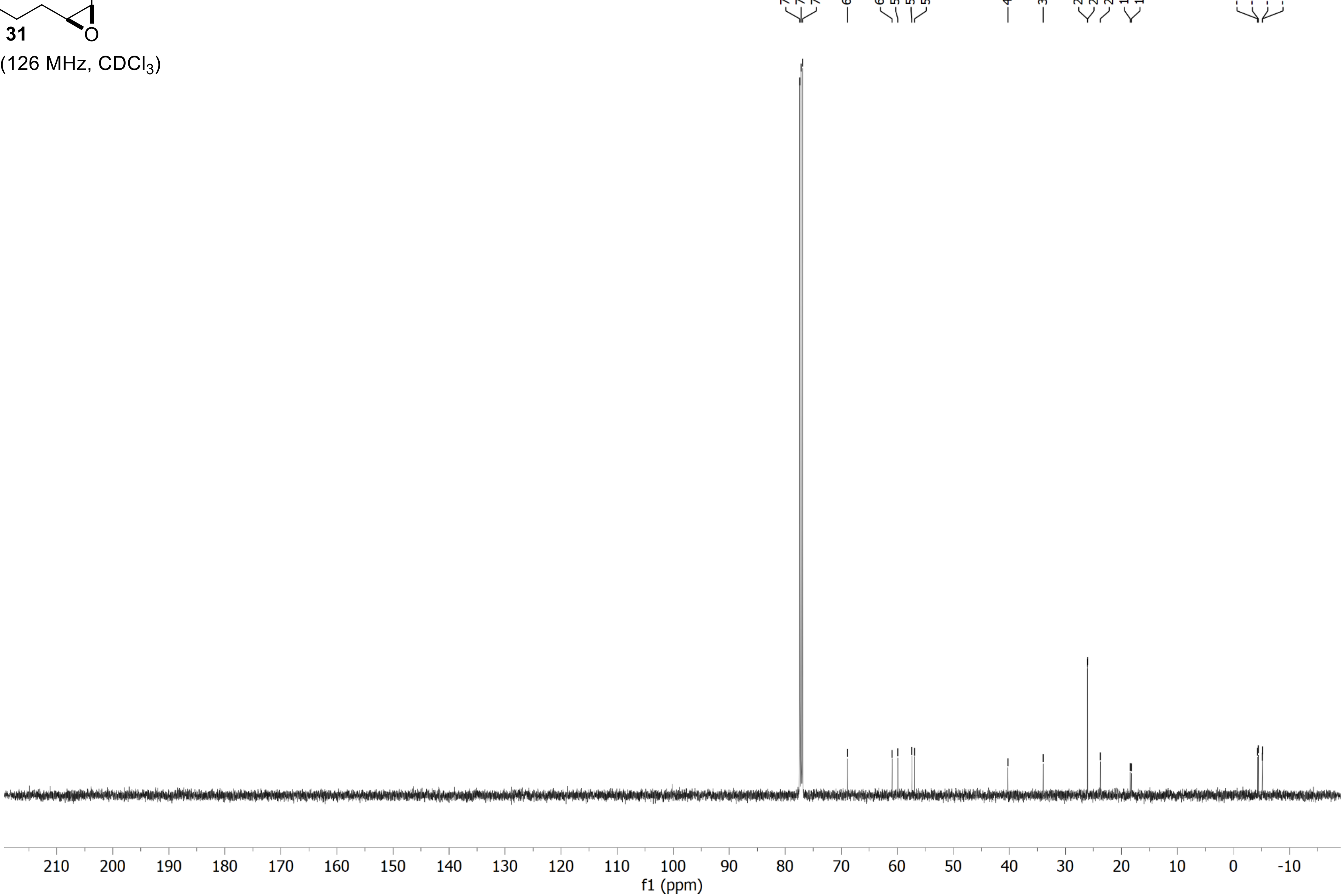

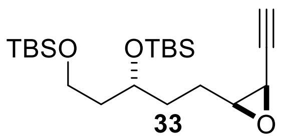

^{1}H NMR (400 MHz, $CDCl_3$)

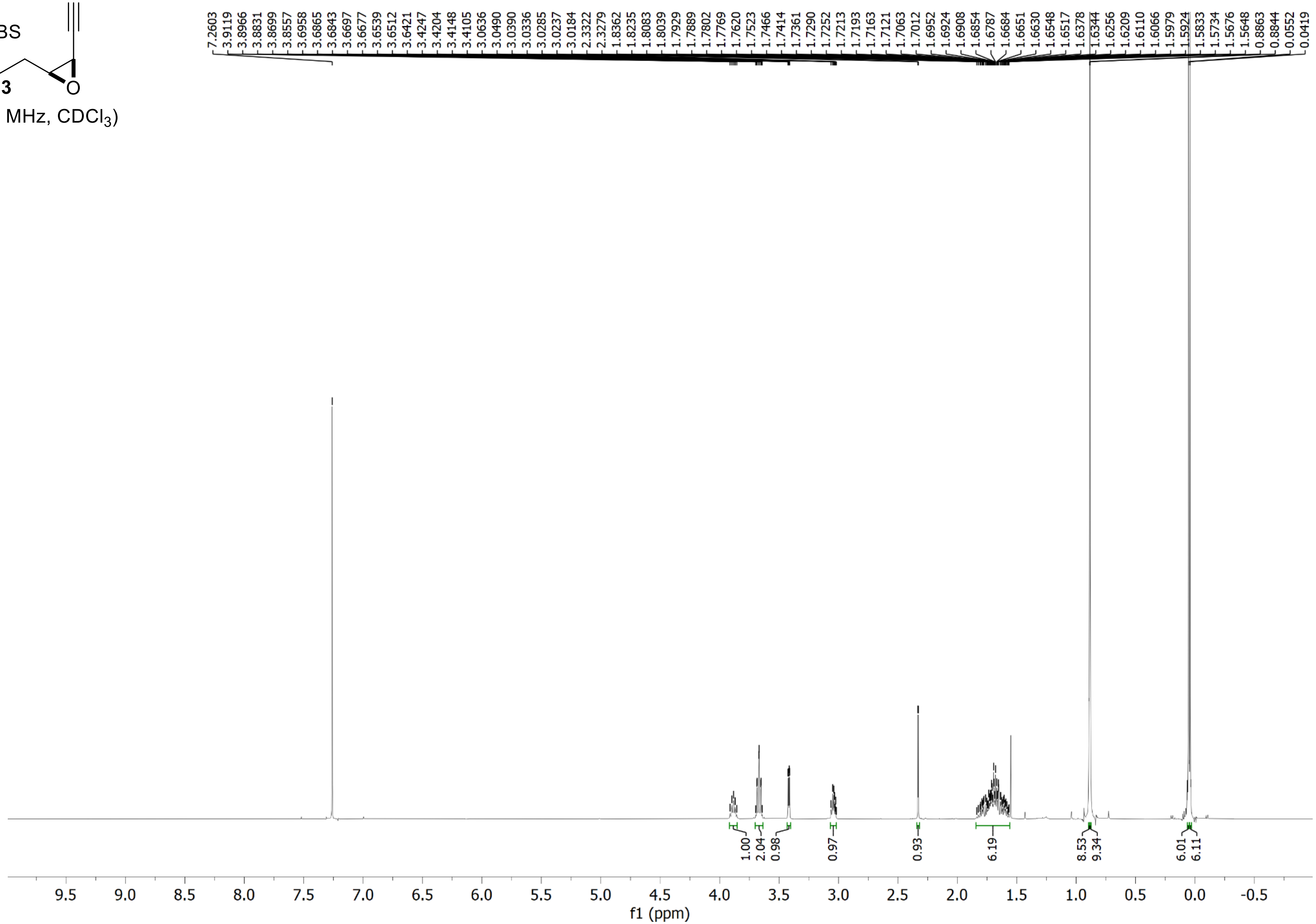

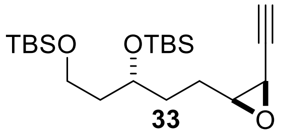

$^{13}C\{^{1}H\}$ NMR (126 MHz, $CDCl_3$)

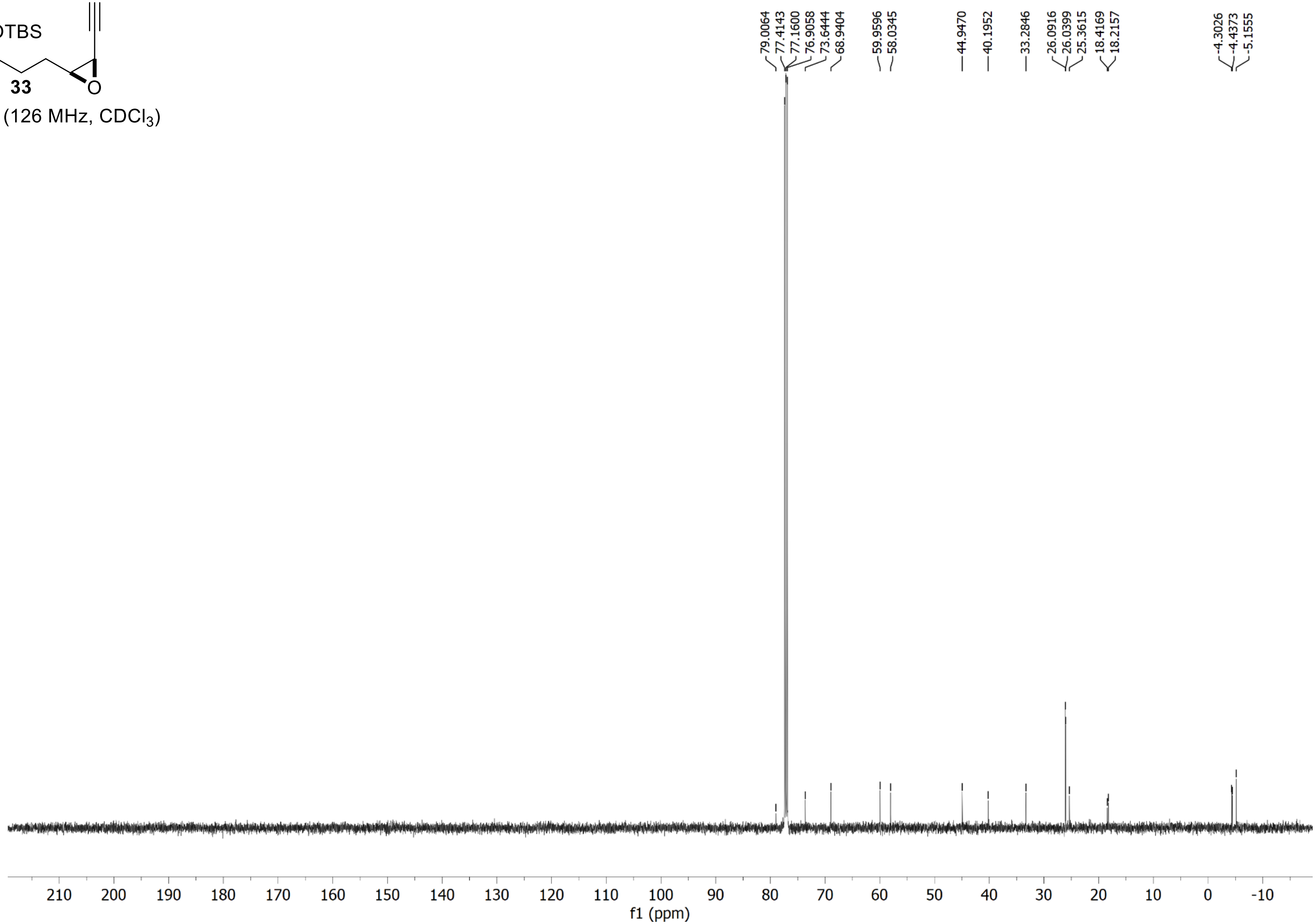

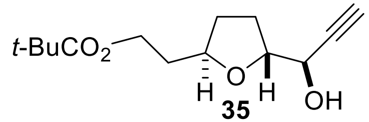

35

^{1}H NMR (500 MHz, $CDCl_3$)

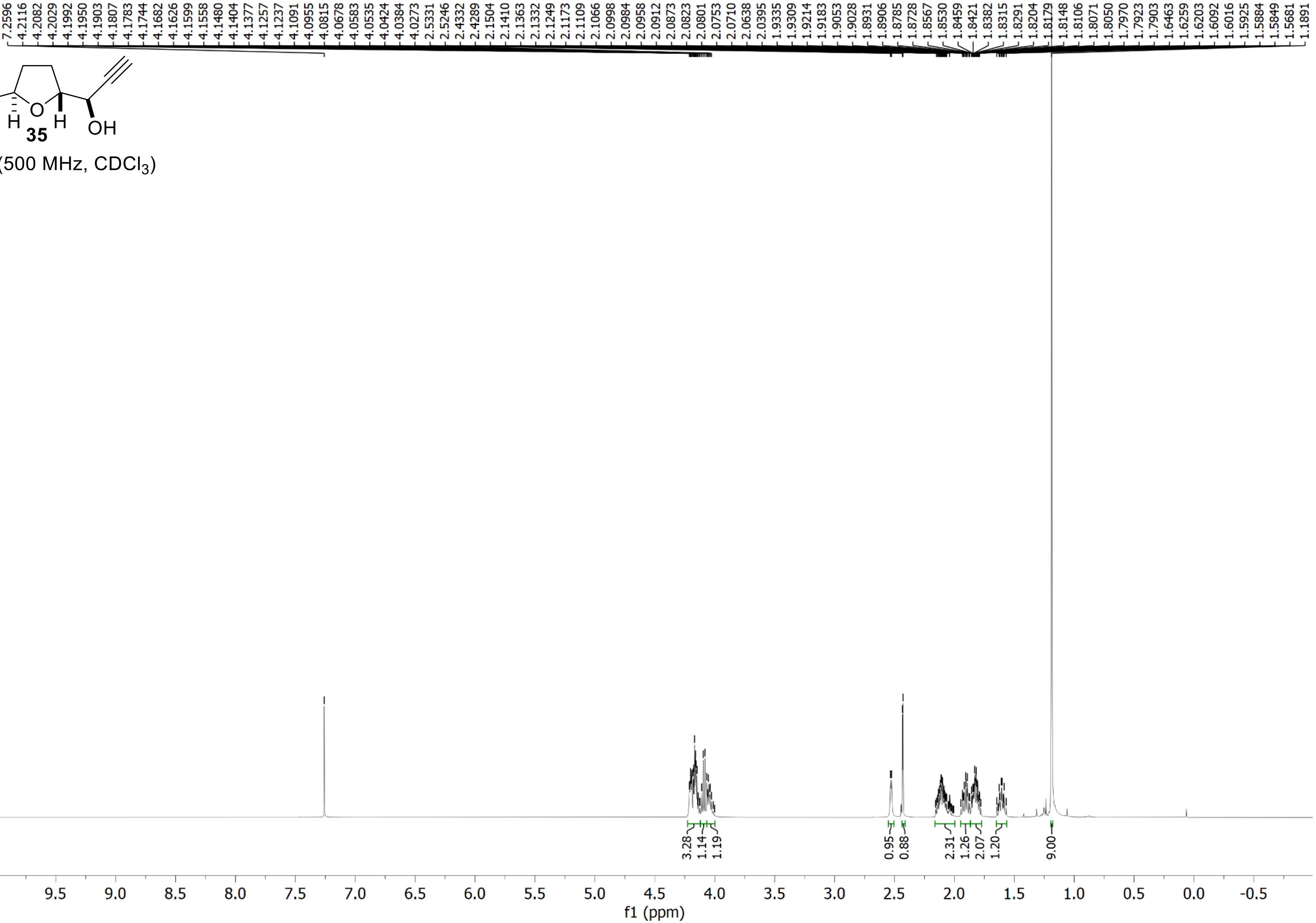

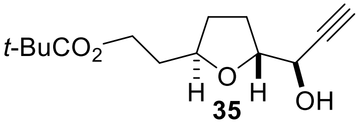

$^{13}C\{^{1}H\}$ NMR (126 MHz, $CDCl_3$)

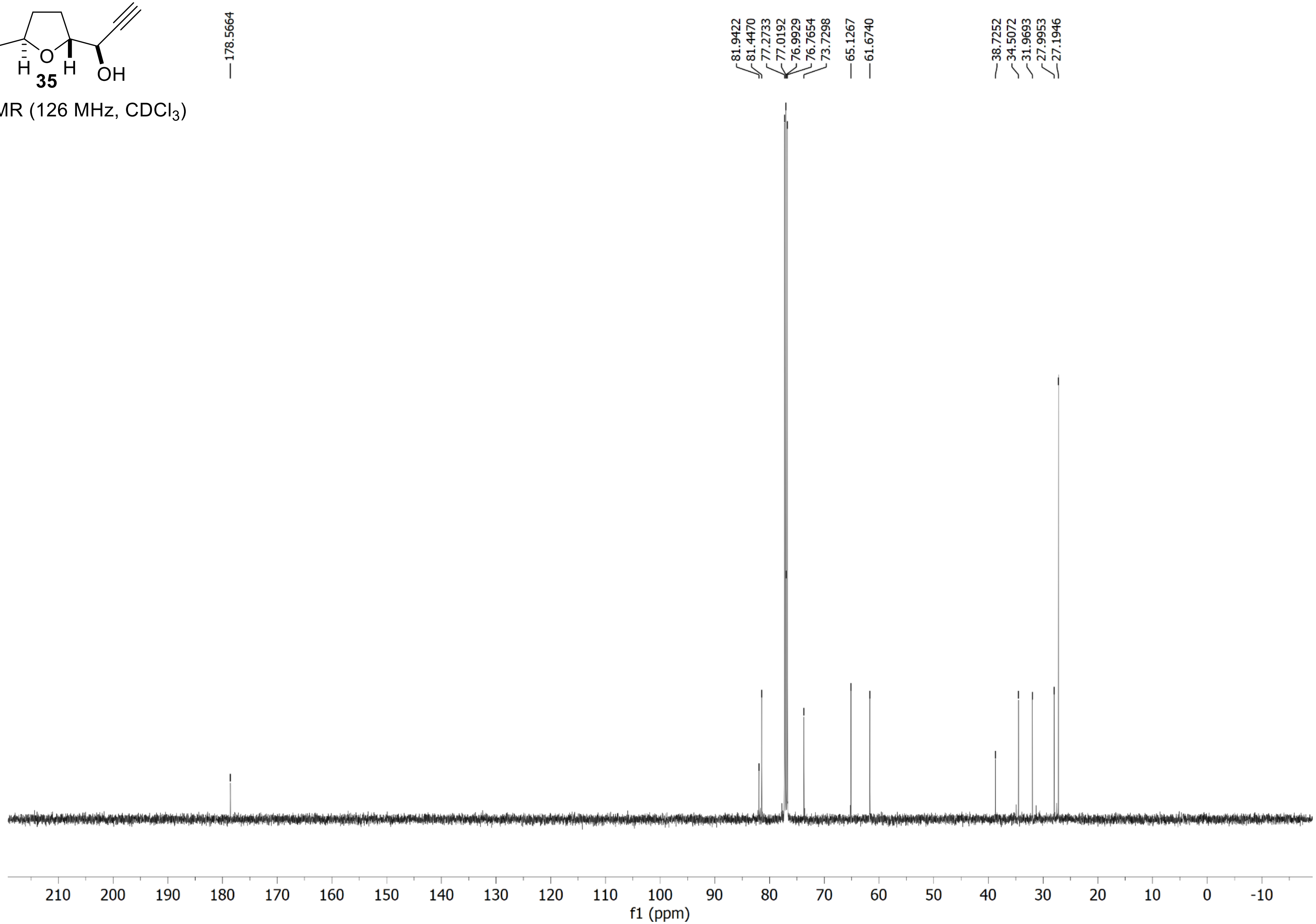

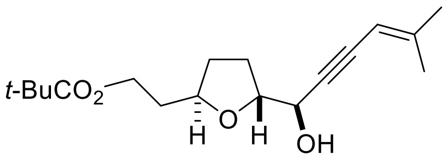

^1H NMR (400 MHz, $CDCl_3$)

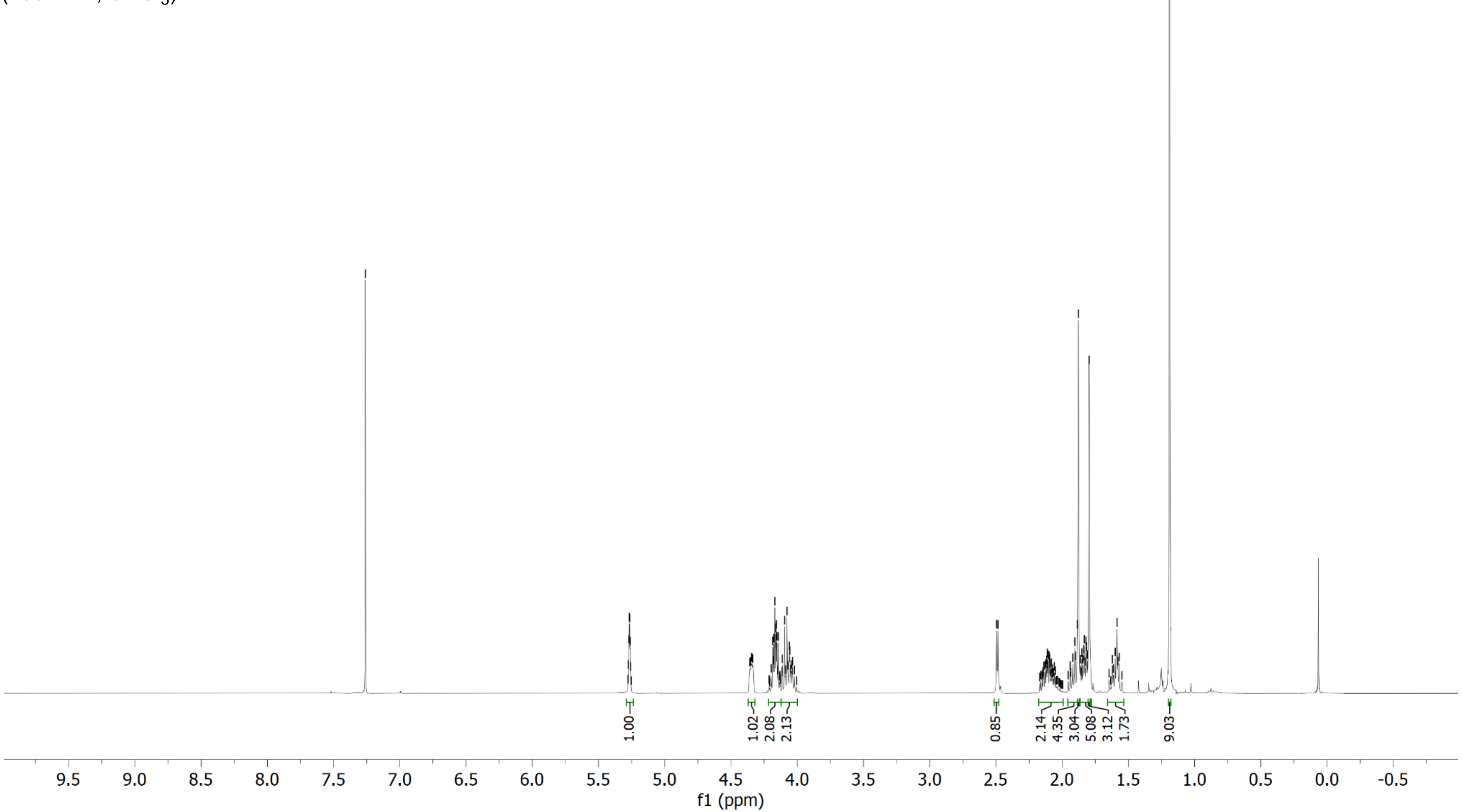

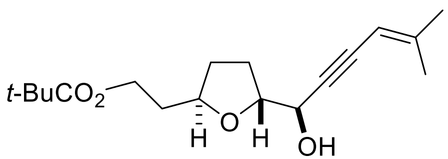

$^{13}C\{^{1}H\}$ NMR (126 MHz, $CDCl_3$)

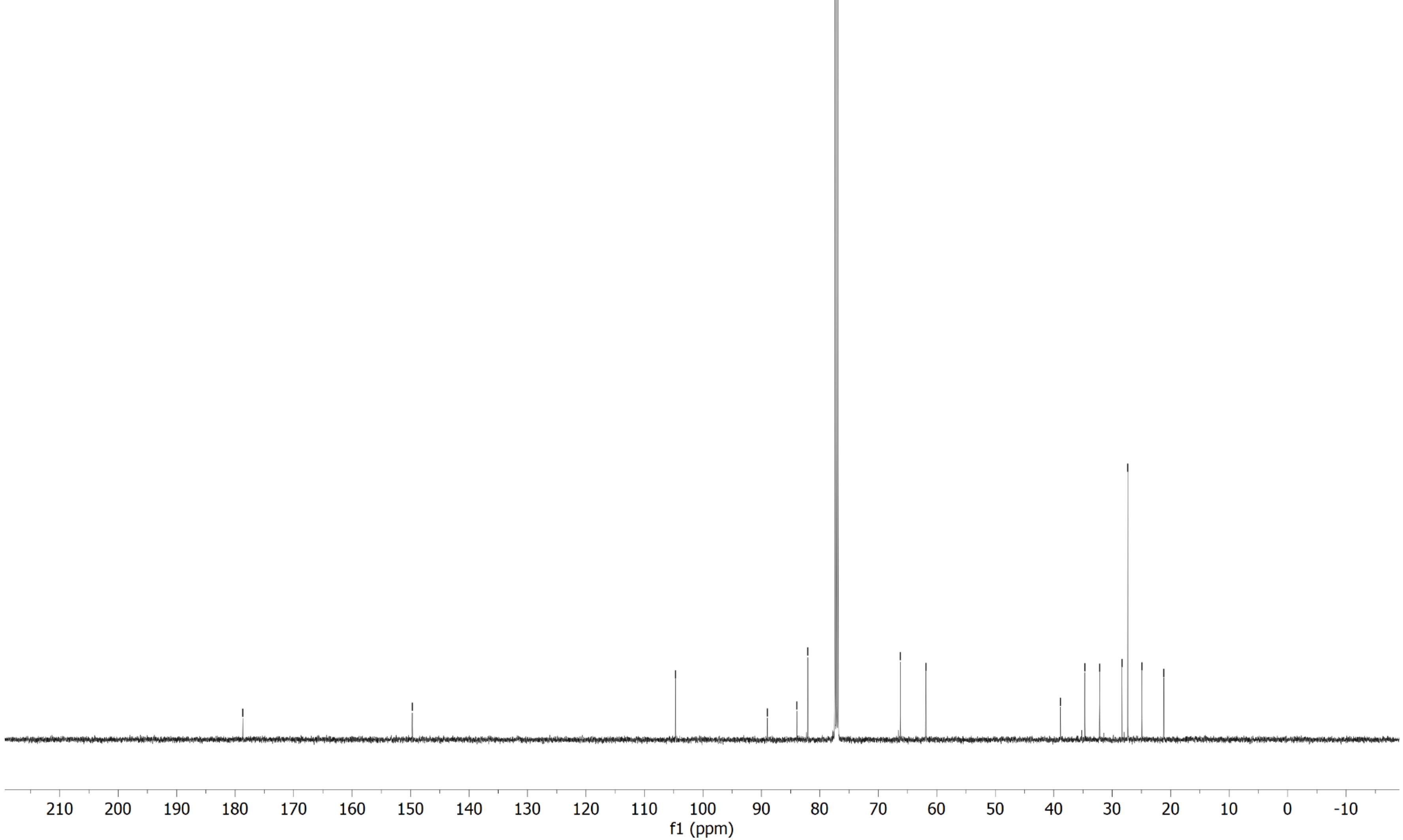

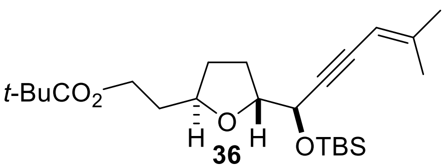

36

^{1}H NMR (500 MHz, $CDCl_3$)

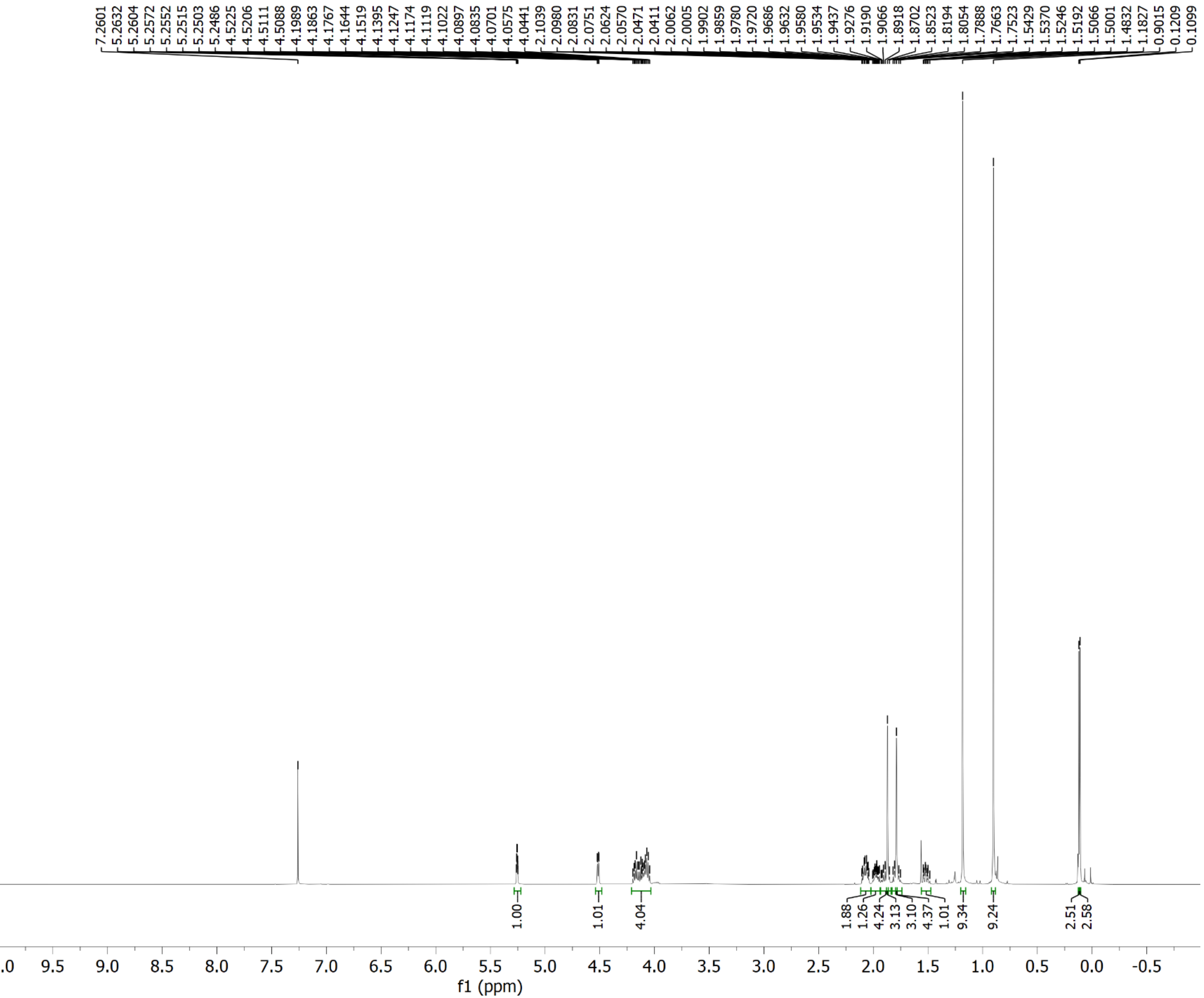

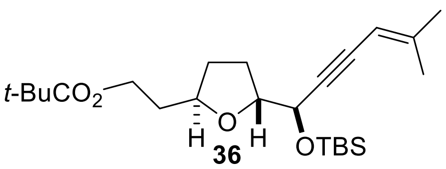

$^{13}C\{^1H\}$ NMR (126 MHz, $CDCl_3$)

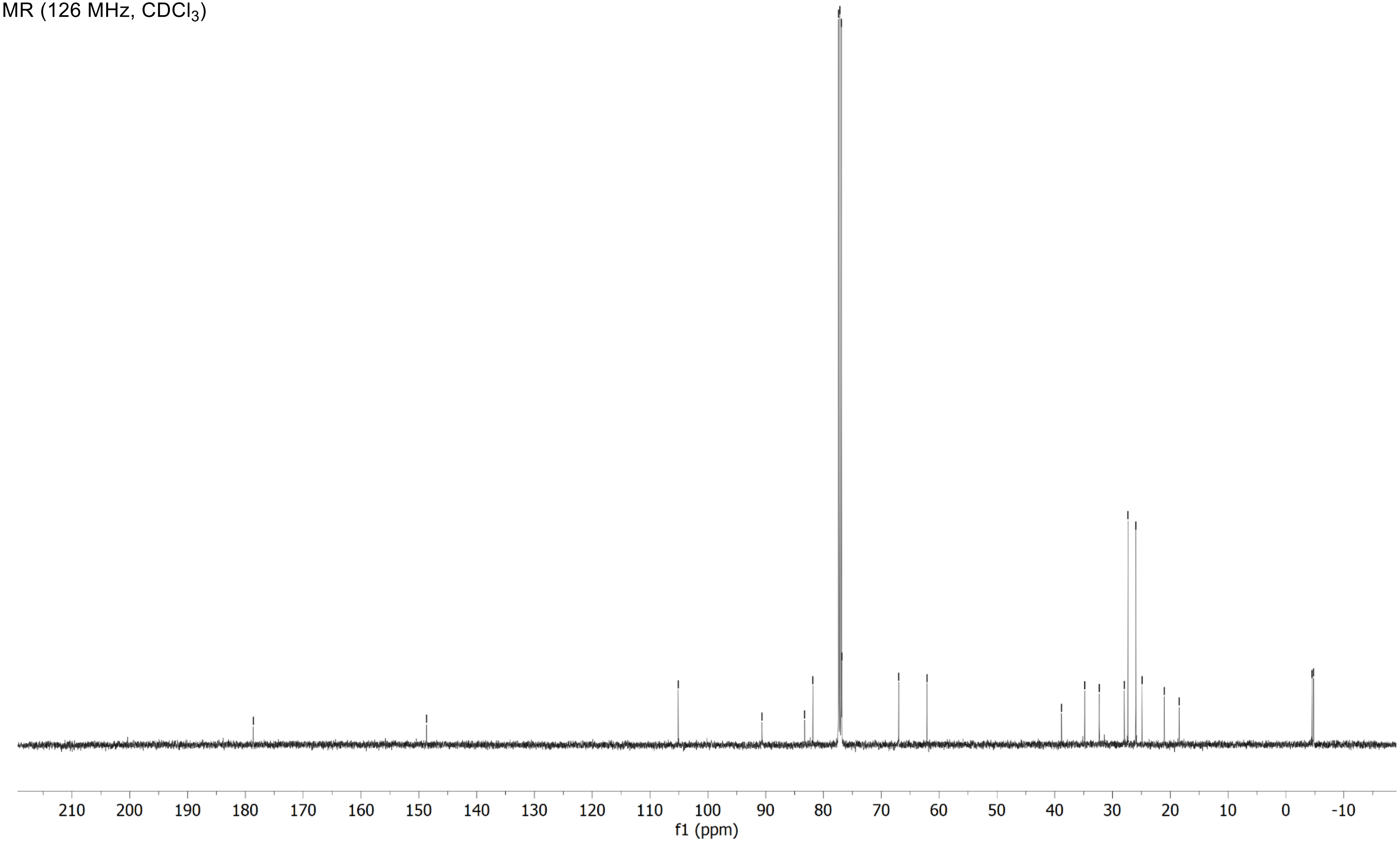

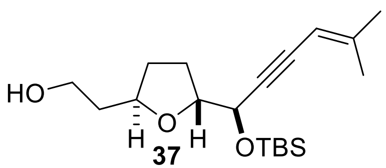

37

^1H NMR (500 MHz, $CDCl_3$)

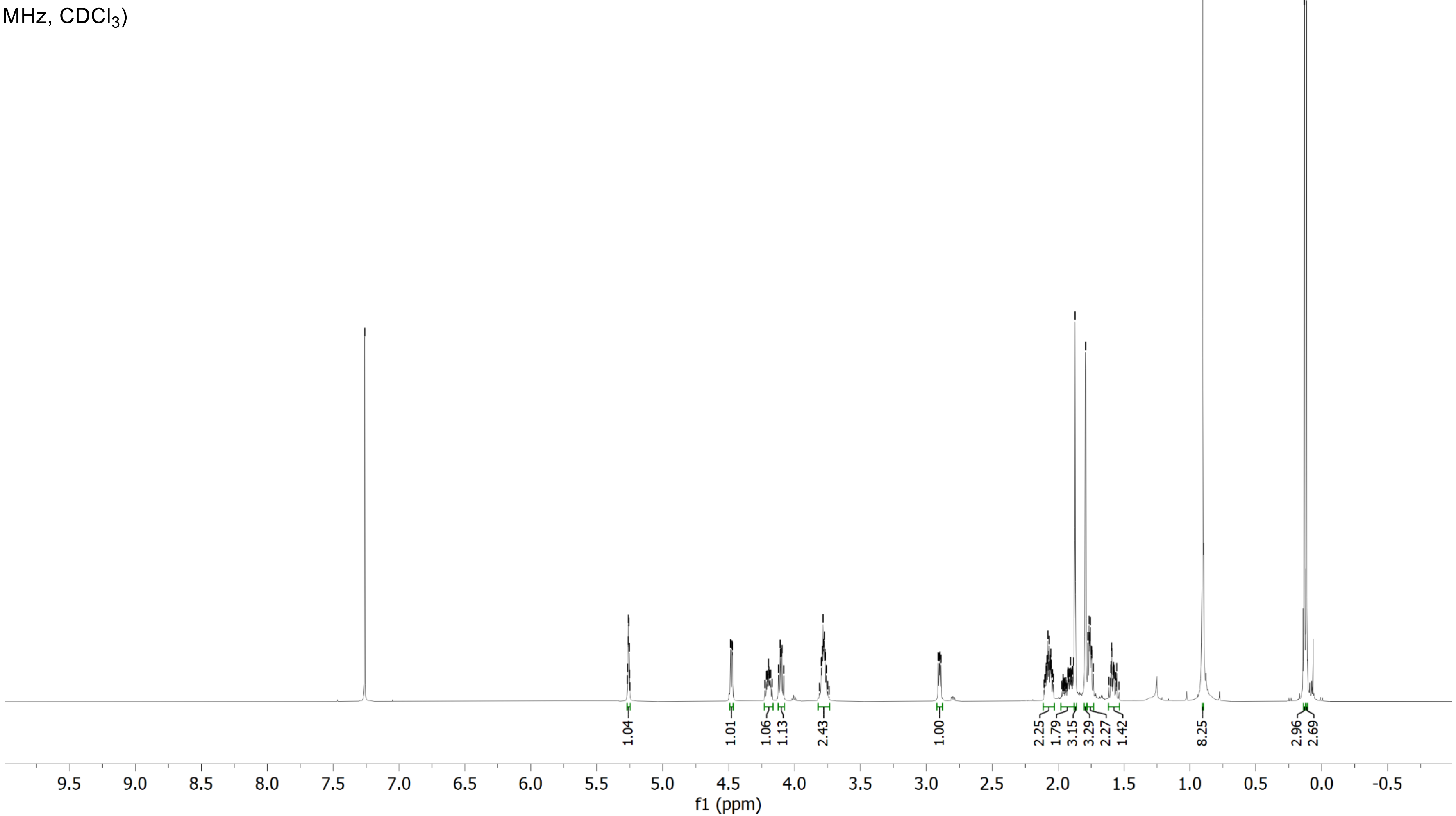

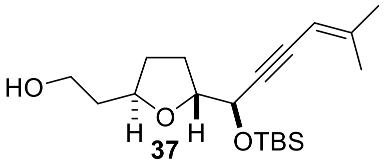

37

$^{13}C\{^{1}H\}$ NMR (126 MHz, $CDCl_3$)

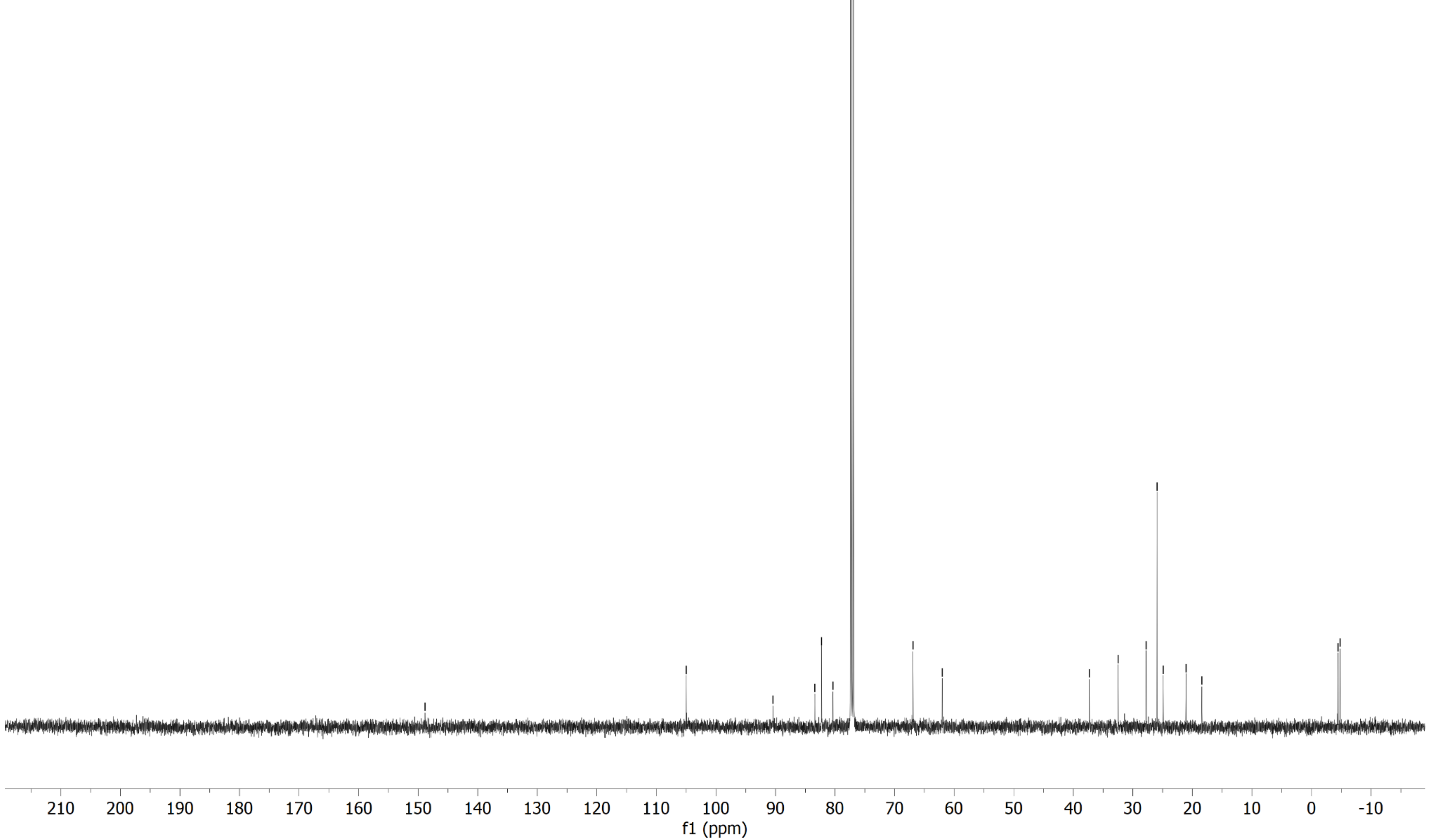

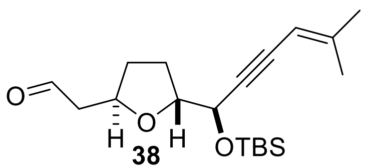

38

^{1}H NMR (400 MHz, $CDCl_3$)

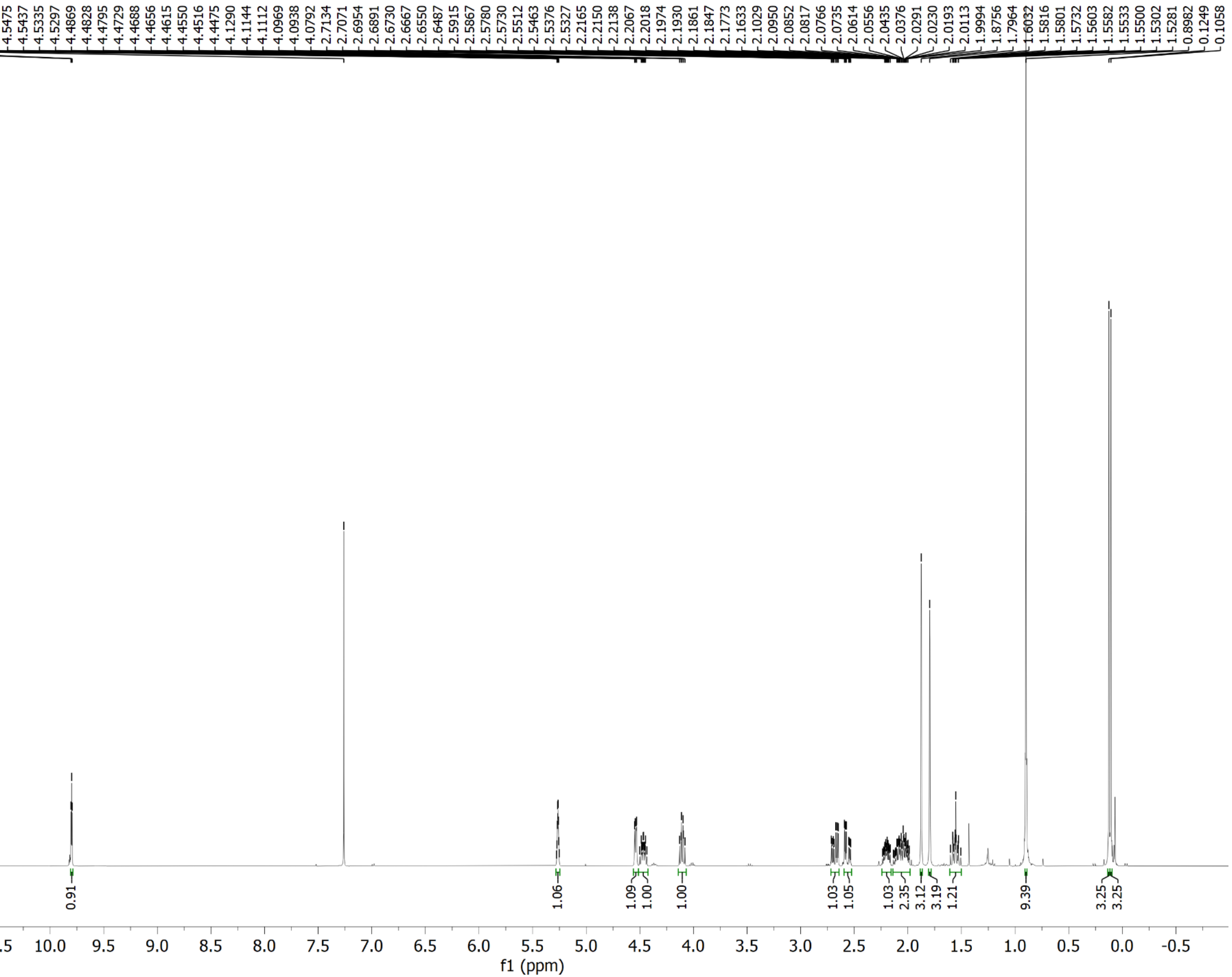

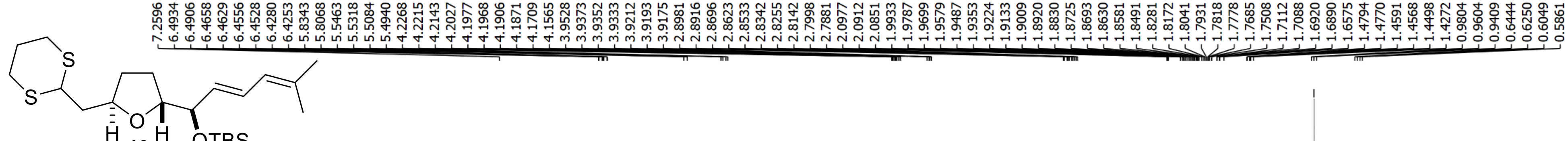

40

^{1}H NMR (400 MHz, $CDCl_3$)

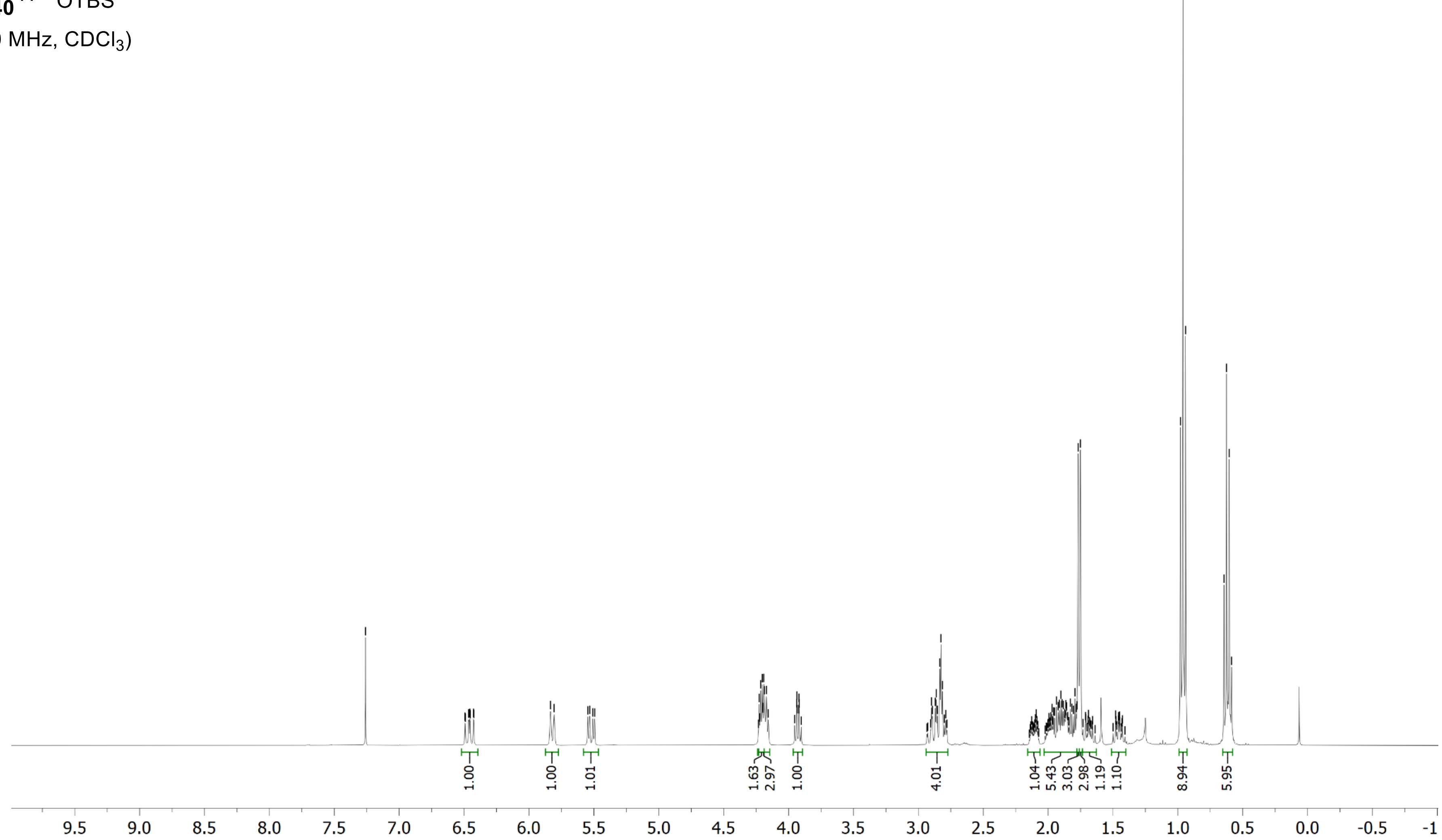

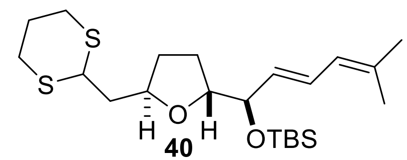

40

$^{13}C\{^{1}H\}$ NMR (126 MHz, $CDCl_3$)

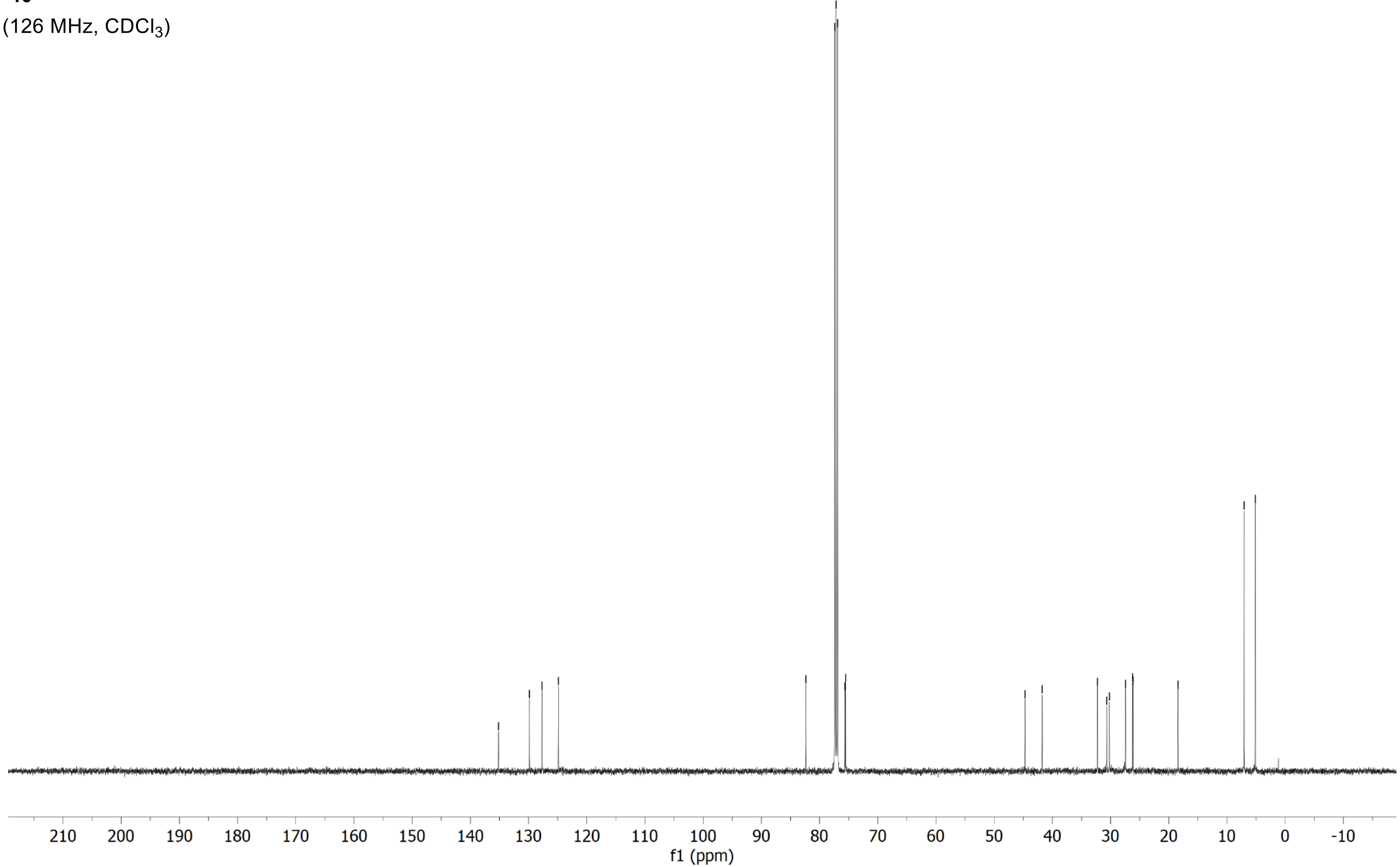

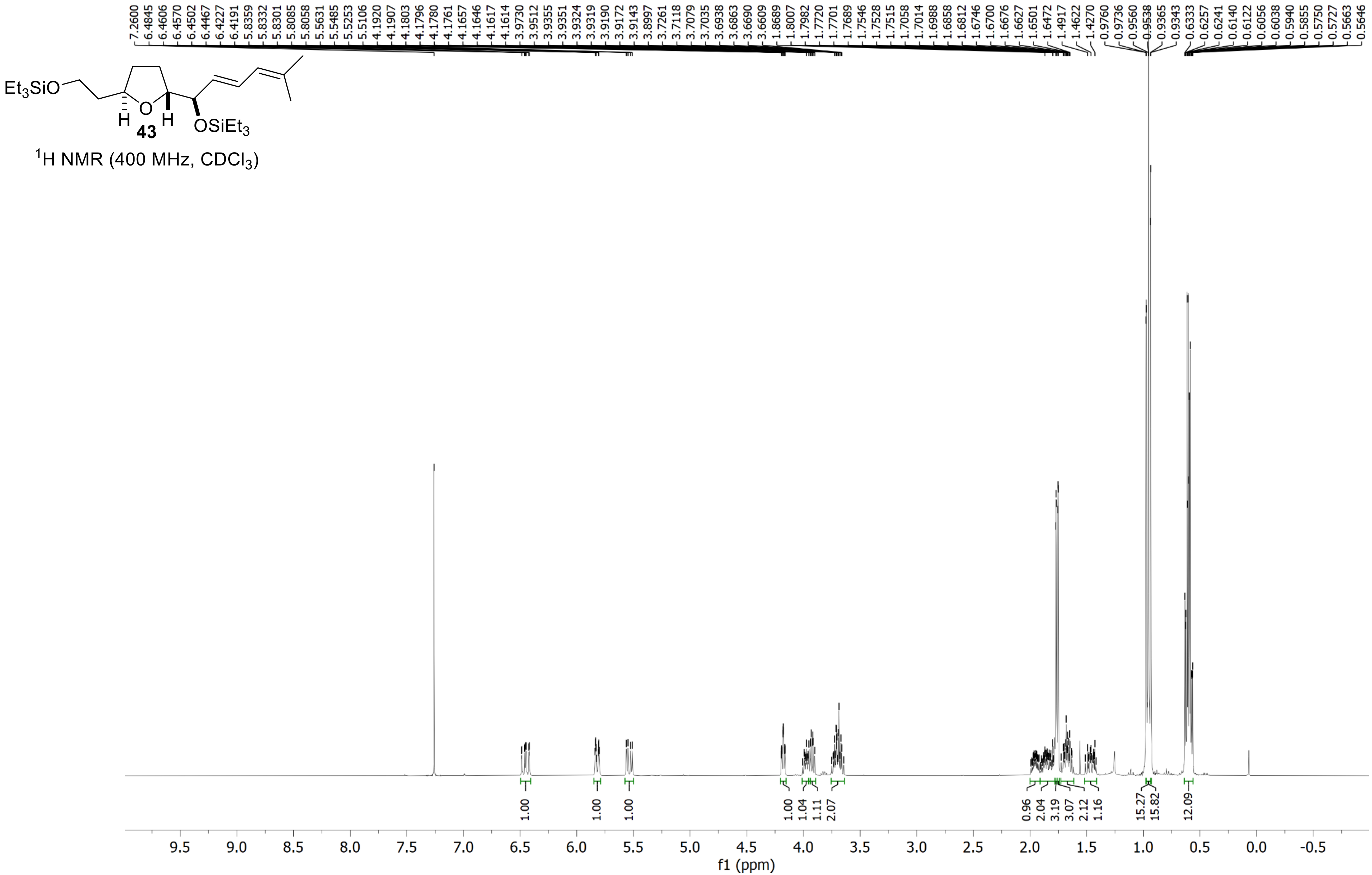

43

^{1}H NMR (400 MHz, $CDCl_3$)

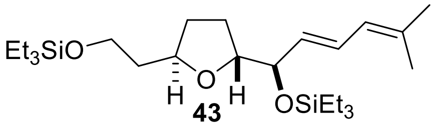

13C{1H} NMR (101 MHz, CDCl3)

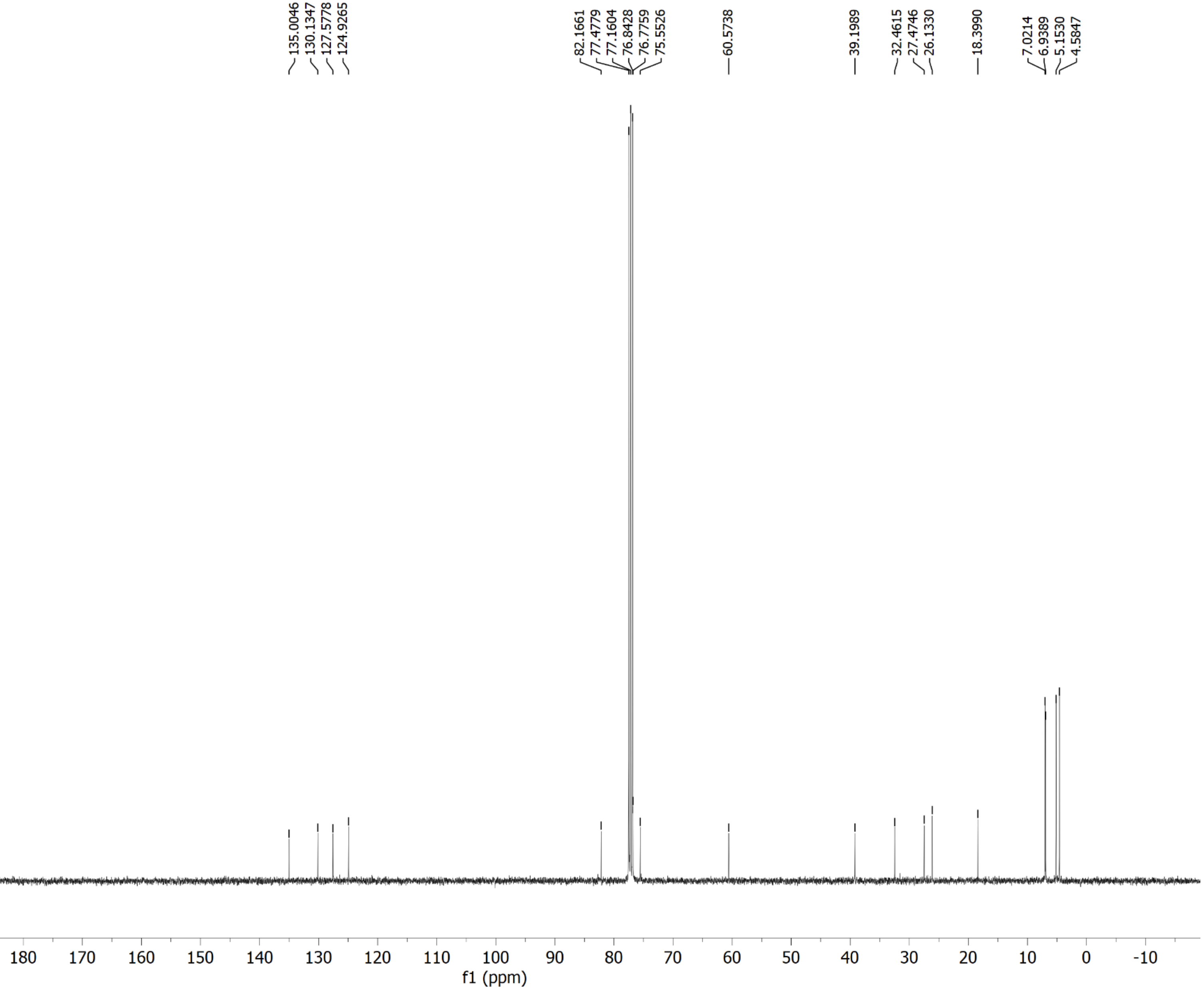

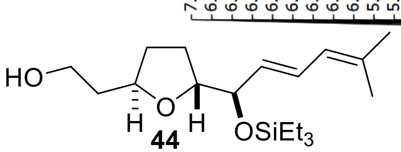

44

^1H NMR (500 MHz, $CDCl_3$)

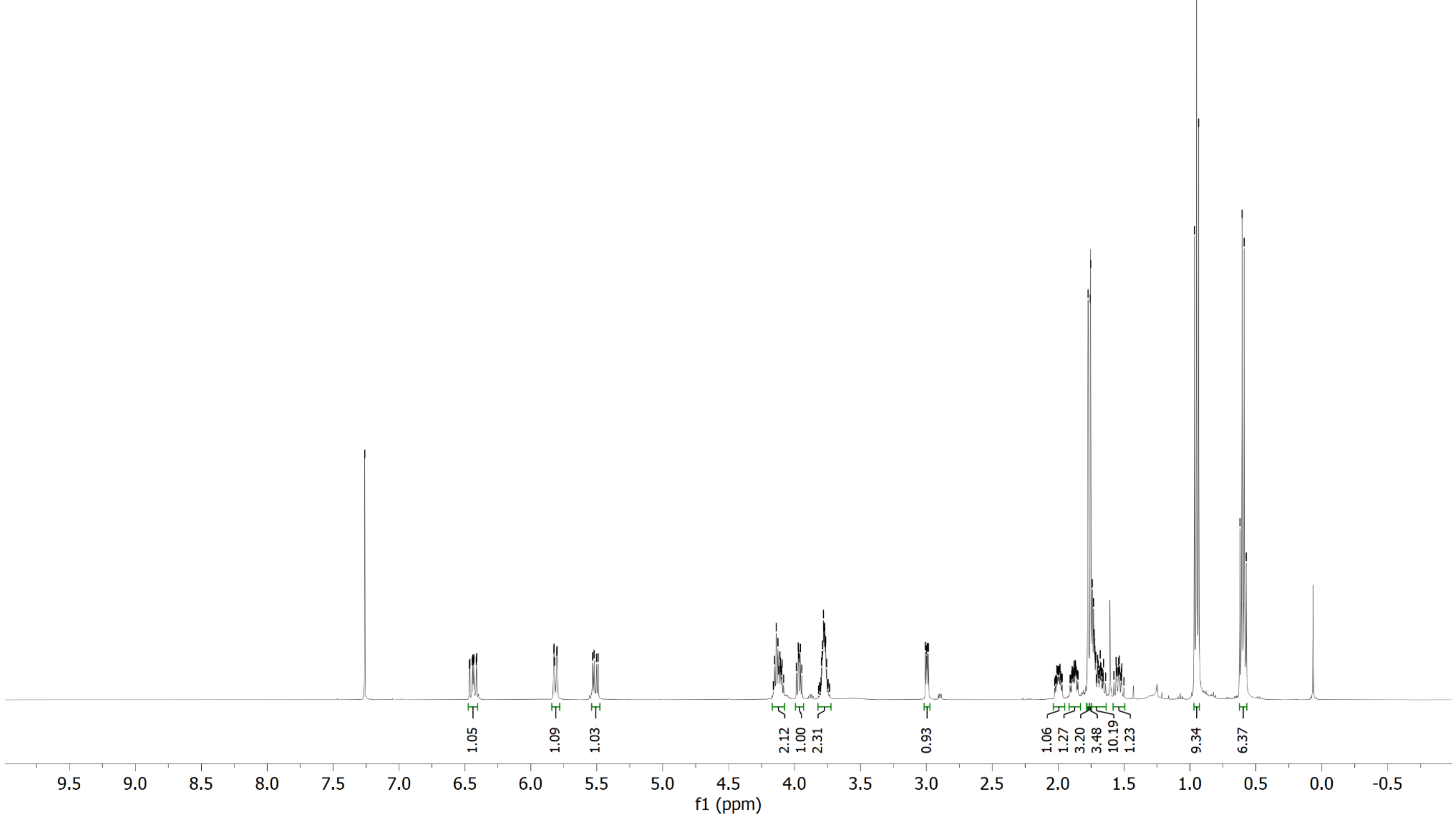

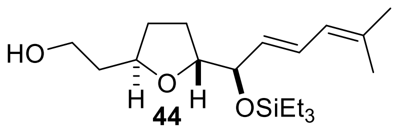

$^{13}C\{^{1}H\}$ NMR (101 MHz, $CDCl_3$)

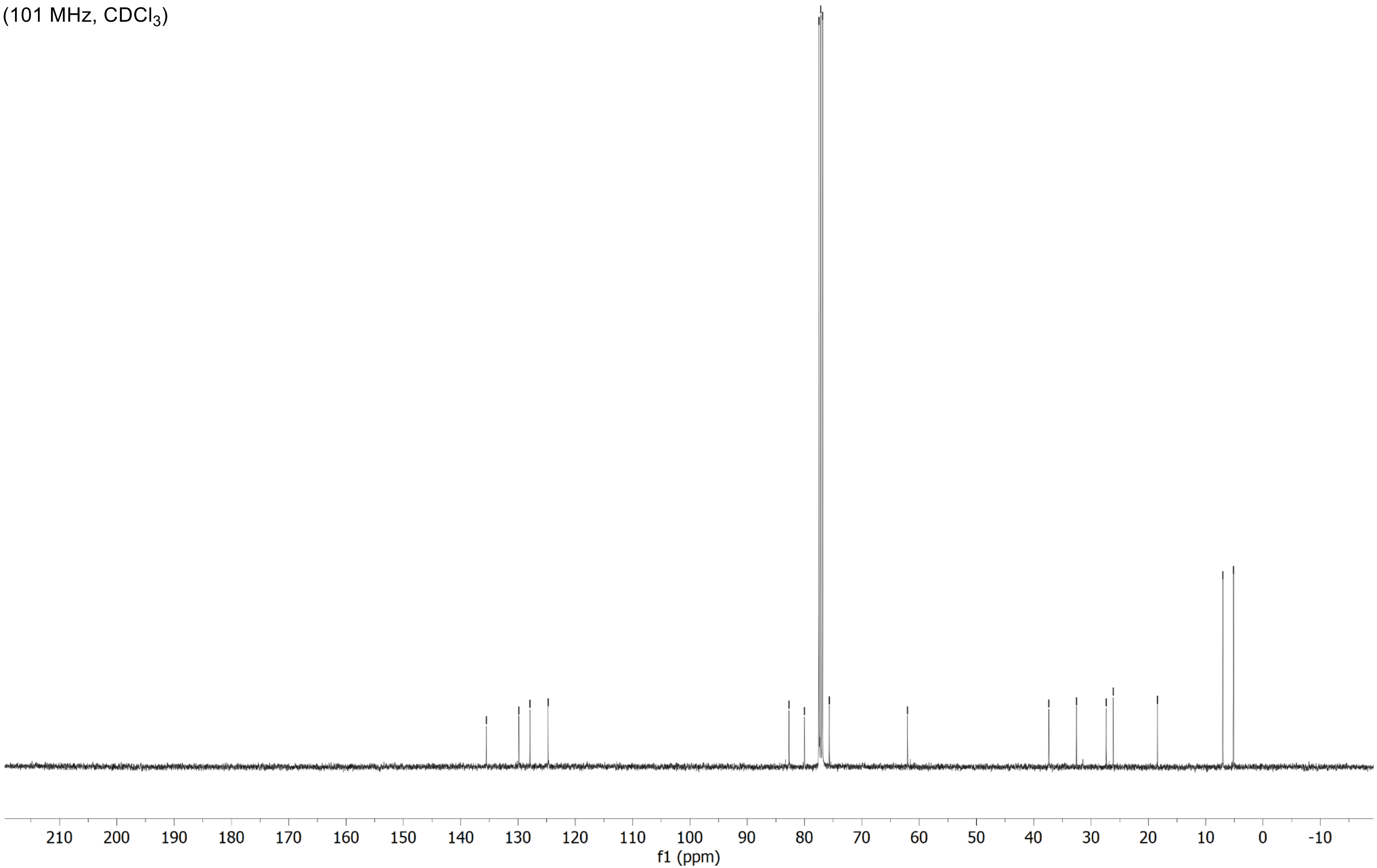

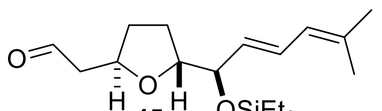

45

^{1}H NMR (400 MHz, $CDCl_3$)

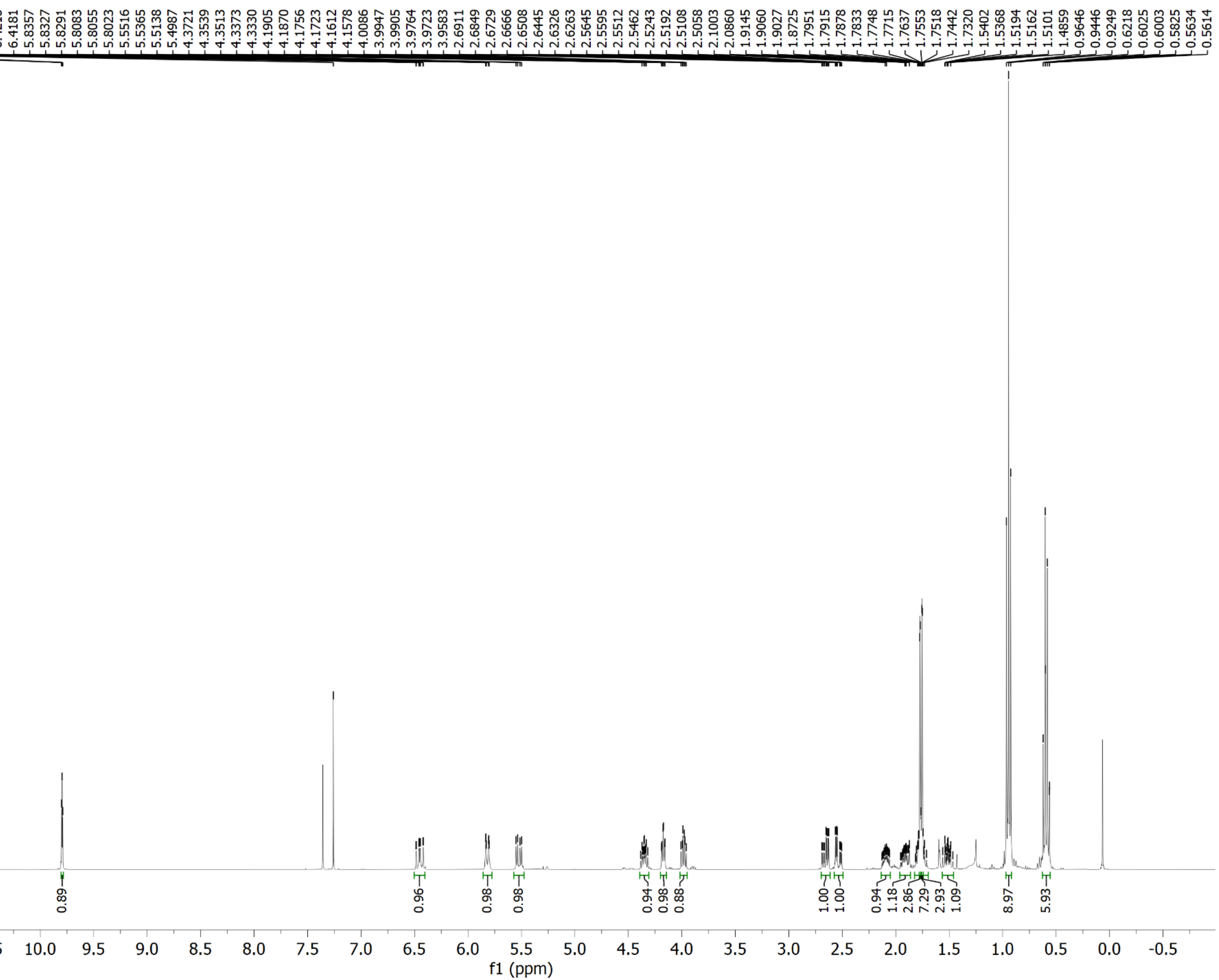

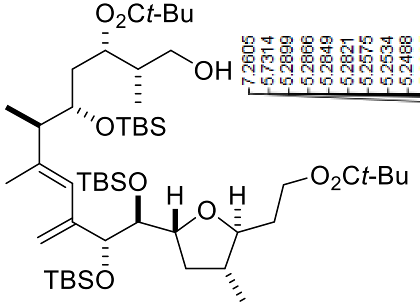

^1H NMR (400 MHz, $CDCl_3$)

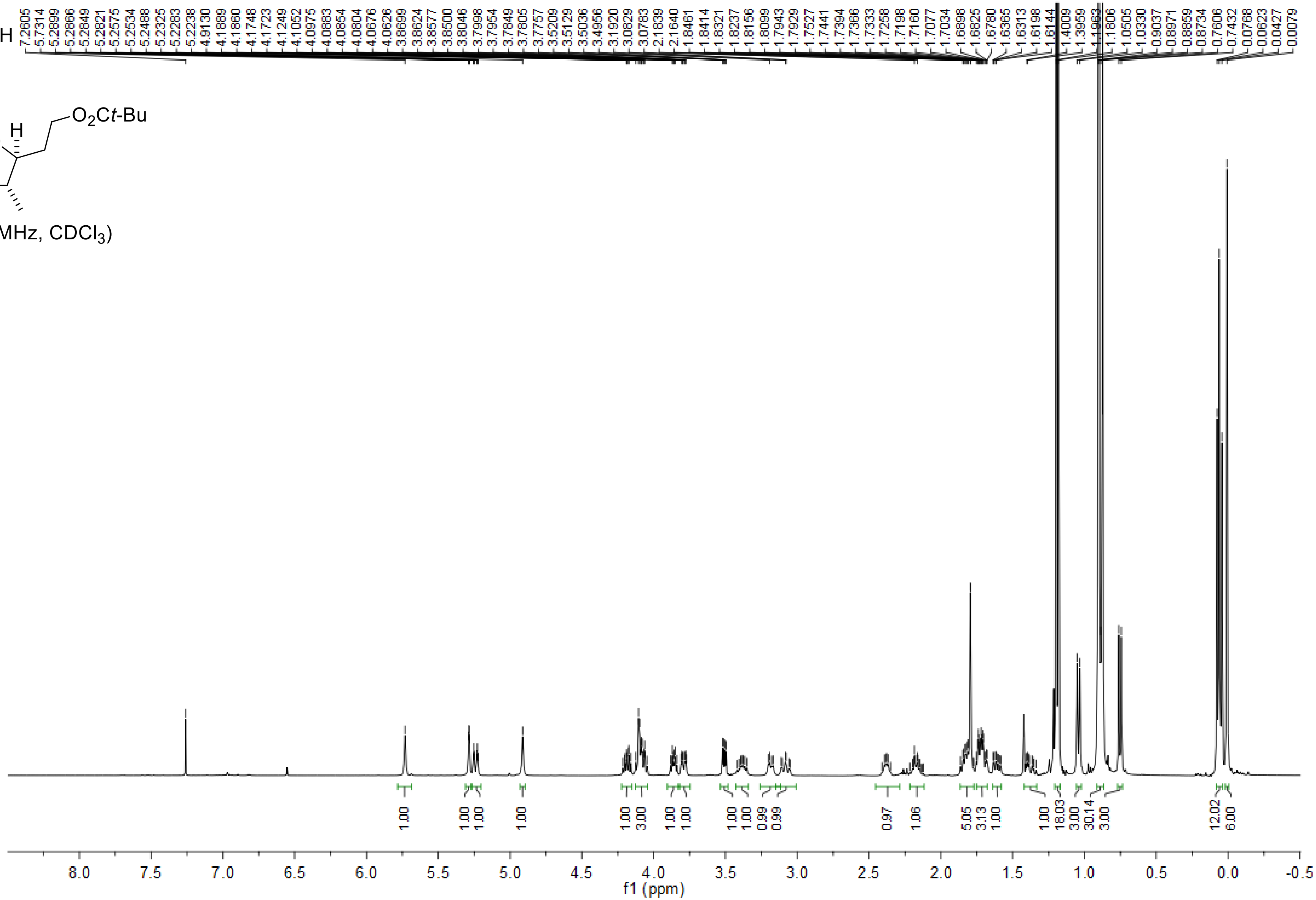

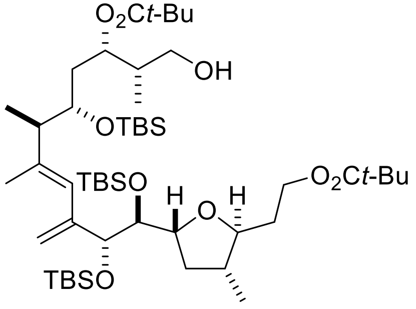

$^{13}C\{^{1}H\}$ NMR (101 MHz, $CDCl_3$)

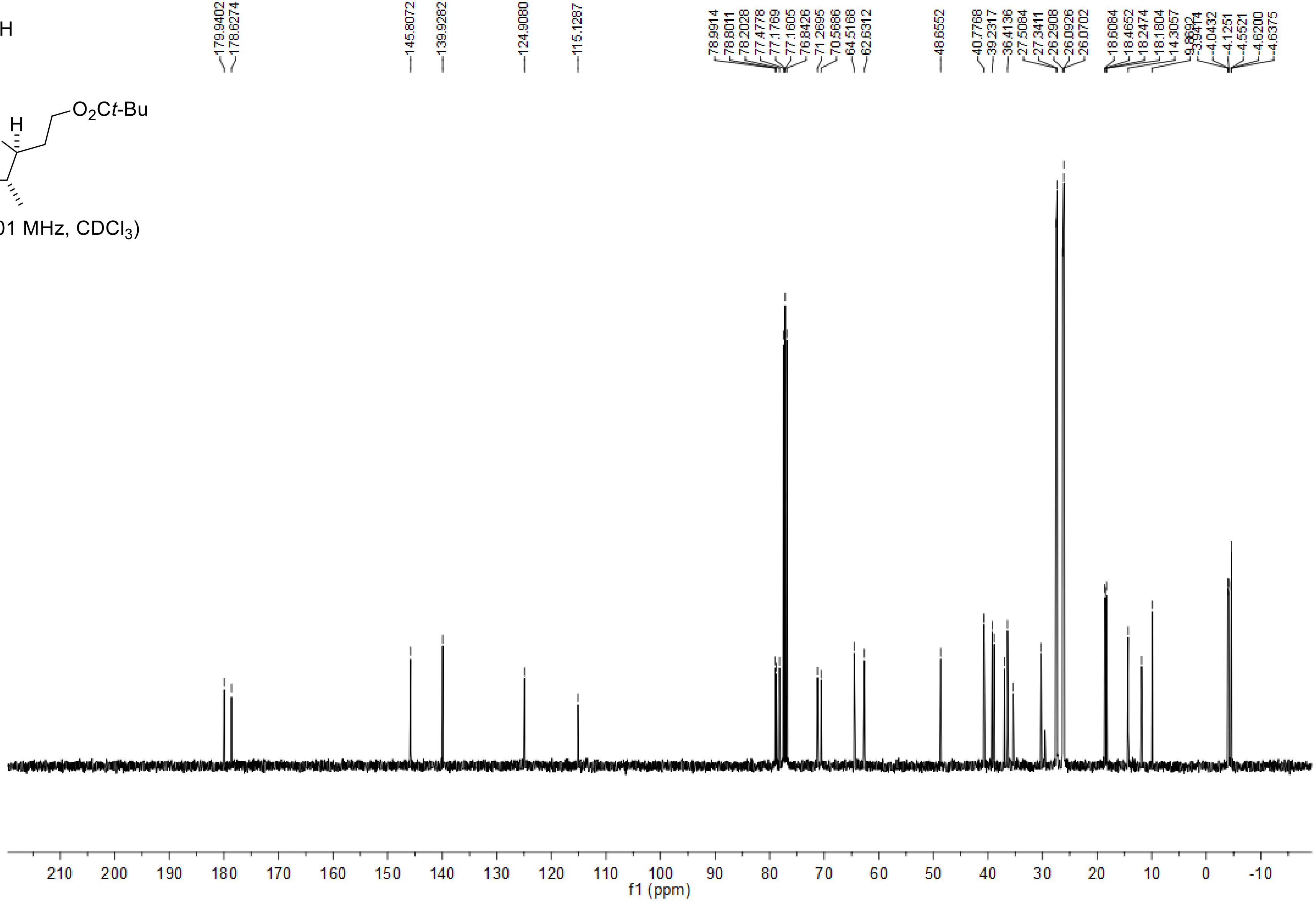

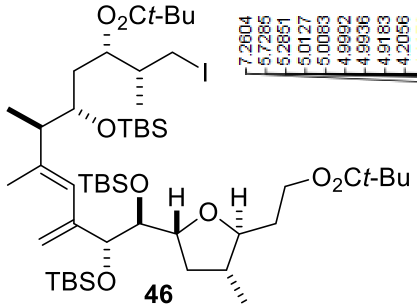

^{1}H NMR (500 MHz, $CDCl_3$)

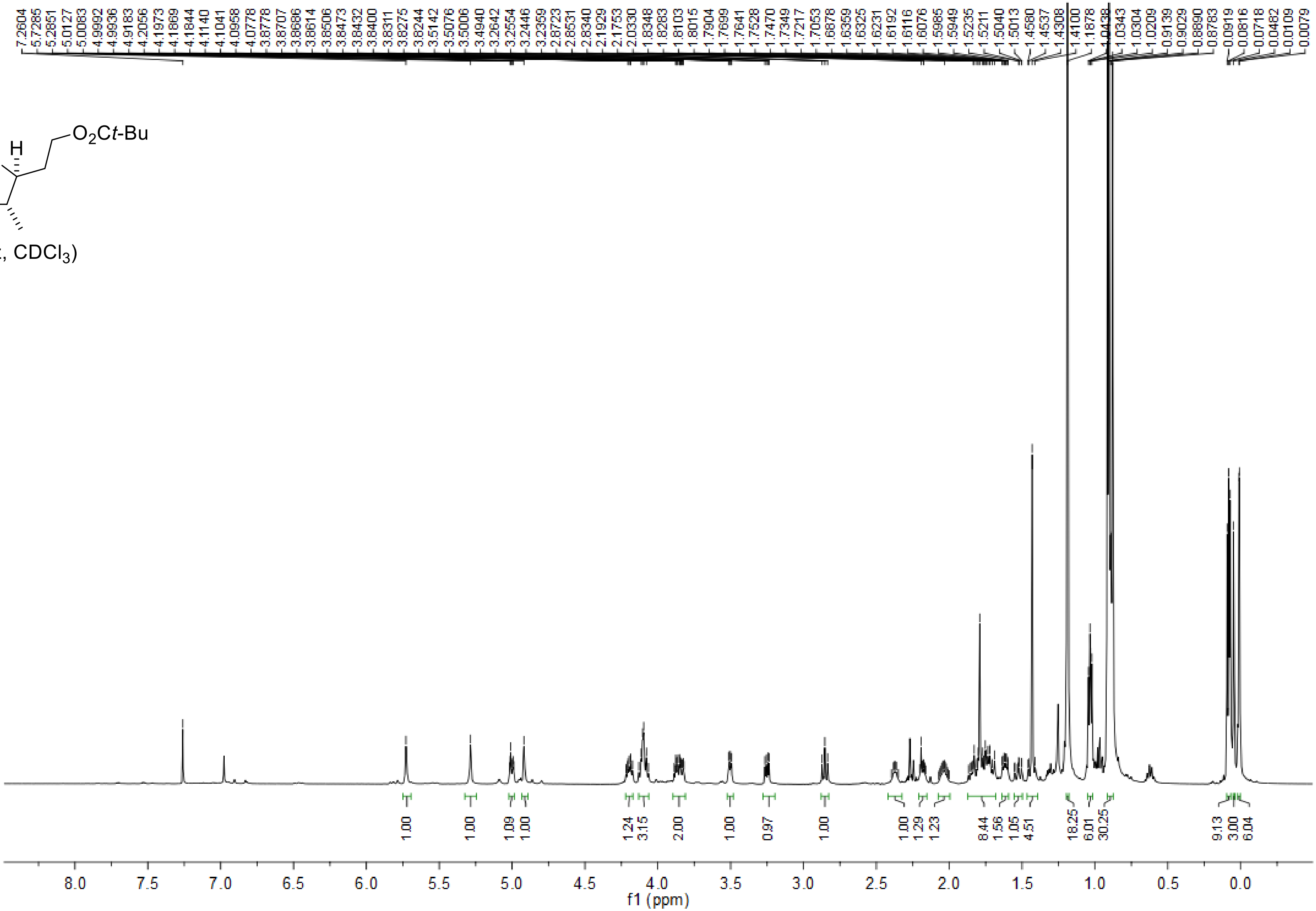

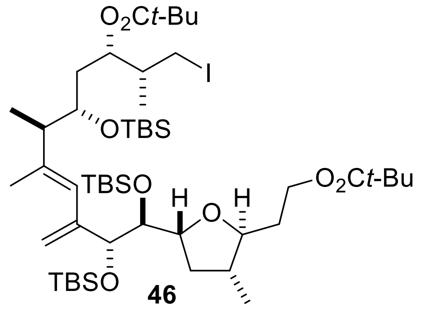

O2Ct-Bu
I
OTBS
TBSO
H
O
H
O2Ct-Bu
TBSO

46

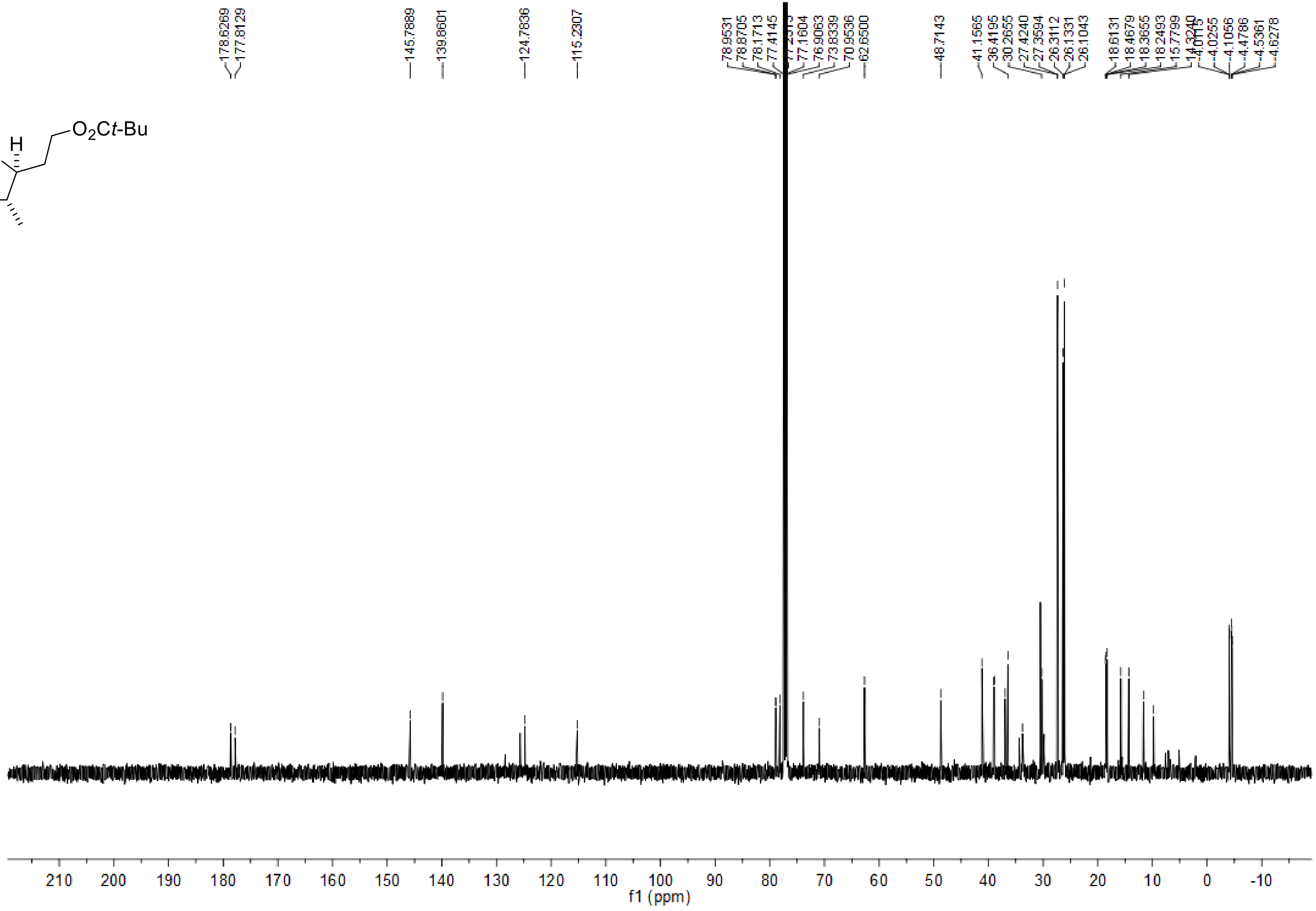

178.6269
177.8129
145.7889
139.8601
124.7836
115.2307
78.9531
78.8705
78.1713
77.4145
77.1604
76.9063
73.8339
70.9536
62.6500
48.7143
41.1565
36.4195
30.2655
27.4240
27.3594
26.3112
26.1331
26.1043
18.6131
18.4679
18.3665
18.2493
15.7799
14.3240
-4.0115
-4.0255
-4.1056
-4.4786
-4.5361
-4.6278
210 200 190 180 170 160 150 140 130 120 110 100 90 80 70 60 50 40 30 20 10 0 -10
f1 (ppm)

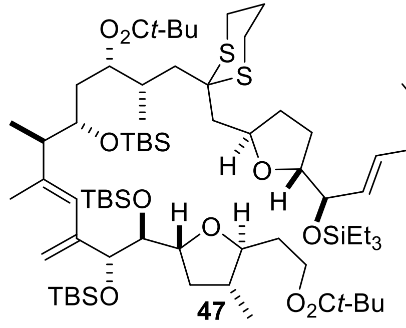

^{1}H NMR (400 MHz, $CDCl_3$)

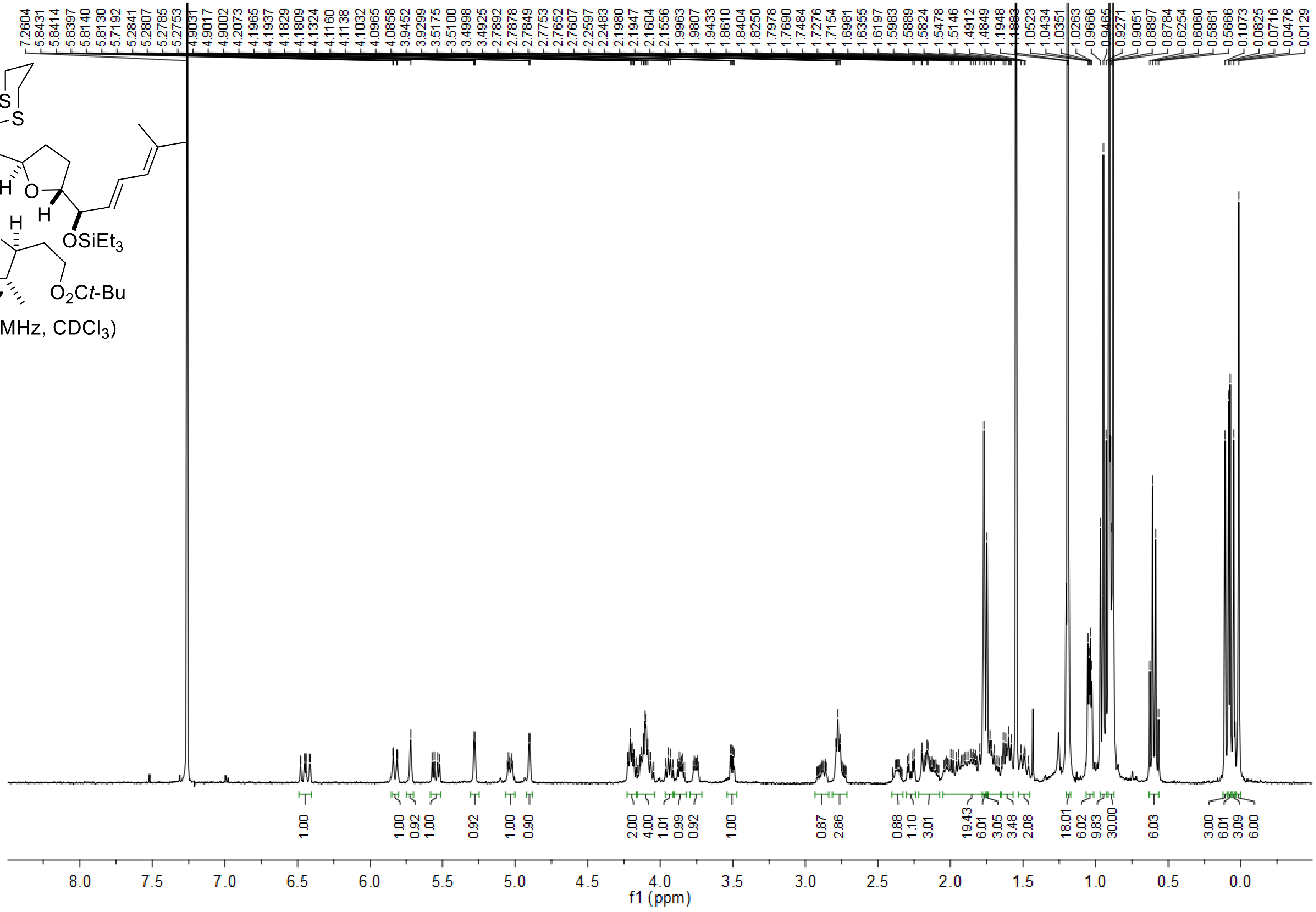

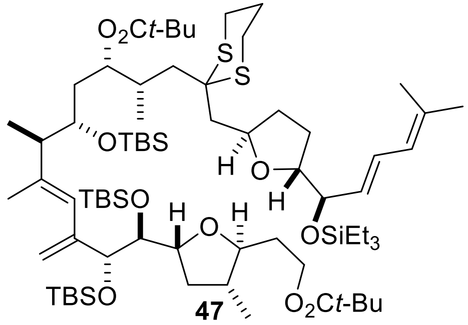

$^{13}C\{^1H\}$ NMR (101 MHz, $CDCl_3$)

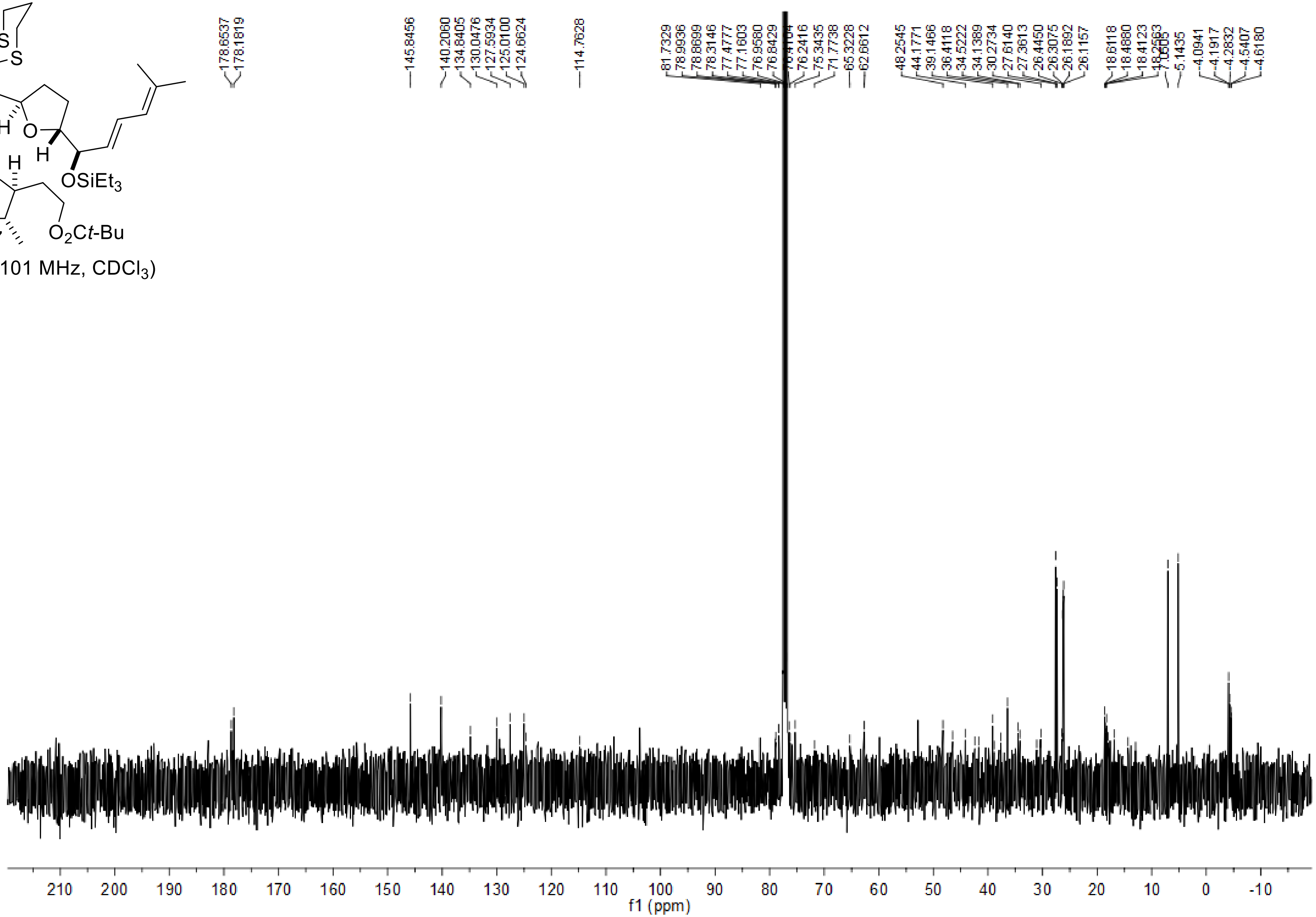

Supplement: Supplementary file 1 — jo2c00850_si_001.pdf [file jo2c00850_si_001.pdf]
